# Integrating proteomic and clinical data to discriminate major psychiatric disorders: Applications for major depressive disorder, bipolar disorder, and schizophrenia

**DOI:** 10.1002/ctm2.929

**Published:** 2022-06-27

**Authors:** Dongyoon Shin, Sang Jin Rhee, Daun Shin, Eun‐Jeong Joo, Hee Yeon Jung, Sungwon Roh, Sang‐Hyuk Lee, Hyeyoung Kim, Minji Bang, Kyu Young Lee, Se Hyun Kim, Jihyeon Lee, Yoseop Kim, Injoon Yeo, Yeongshin Kim, Jaenyeon Kim, Jun Soo Kwon, Kyooseob Ha, Yong Min Ahn, Youngsoo Kim

**Affiliations:** ^1^ Department of Biomedical Sciences Seoul National University College of Medicine Seoul Republic of Korea; ^2^ Institute of Medical and Biological Engineering Medical Research Center Seoul National University College of Medicine Seoul Republic of Korea; ^3^ Department of Psychiatry Seoul National University College of Medicine Seoul Republic of Korea; ^4^ Department of Neuropsychiatry Seoul National University Hospital Seoul Republic of Korea; ^5^ Department of Neuropsychiatry School of Medicine Eulji University Daejeon Republic of Korea; ^6^ Department of Psychiatry Uijeongbu Eulji Medical Center Eulji University Seoul Republic of Korea; ^7^ Department of Psychiatry SMG‐SNU Boramae Medical Center Seoul Republic of Korea; ^8^ Institute of Human Behavioral Medicine Seoul National University Medical Research Center Seoul Republic of Korea; ^9^ Department of Psychiatry Hanyang University Hospital and Hanyang University College of Medicine Seoul Republic of Korea; ^10^ Department of Psychiatry CHA Bundang Medical Center CHA University School of Medicine Seongnam Republic of Korea; ^11^ Department of Psychiatry Inha University Hospital Incheon Republic of Korea; ^12^ Department of Psychiatry Nowon Eulji University Hospital Seoul Republic of Korea; ^13^ Interdisciplinary Program of Bioengineering Seoul National University College of Engineering Seoul Republic of Korea

Dear editor,

We report that integrating proteomic and clinical data enables objective differentiation between major depressive disorder (MDD), bipolar disorder (BD), and schizophrenia (SCZ). These major psychiatric disorders are associated with mortality and life‐long disability.^1^ However, objective discrimination of these disorders remains a formidable challenge. Thus, this study aimed to distinguish MDD, BD, and SCZ by integrating targeted/untargeted proteomic data obtained from liquid chromatography‐mass spectrometry (LC‐MS) and clinical data.

The entire design of the current study is illustrated in Figure S1, and detailed information of the following methods is described in Supporting Information. The study included 675 subjects [171 SCZ, 170 BD, 174 MDD, and 160 healthy controls (HC)], aged 19 to 65 years, and proteomic analyses was performed from each plasma sample. After the final quantifiable 642 peptides for MDD, BD, SCZ, and HC were determined (Figure S2), LC‐multiple reaction monitoring (MRM)‐MS was performed on individual plasma samples, followed by LC‐high resolution MS‐based proteomic profiling on pooled plasma samples (Figure S3A,B). Logarithmic transformation was performed on the LC‐MRM‐MS data for the stable 588 peptides, followed by batch effect correction (Figure S4A,B). The 515 patients were divided into training, validation, and independent test sets (6:2:2). There were significant differences in demographics, medication use, and clinical features between groups (Tables S1–S4). Therefore, peptides that were significant with demographics, medication use, and chronicity of disease/medication, and not with disease types were excluded by ANCOVA, for each pairwise comparisons between groups, in the training sets. Furthermore, peptides with multicollinearity were excluded, resulting in 23, 29, and 30 proteomic candidate features (proteins) for differentiating MDD versus BD, MDD versus SCZ, and BD versus SCZ, respectively (Table S5). These proteins showed consistent expression level patterns across disease types, low inter‐correlation with covariates (Figure S5A–C), and low interdependence between each other (Figure S6A–C).

Multiprotein‐marker (MPM) models were constructed by LASSO (least absolute shrinkage and selection operator) with 100‐repeated 5‐fold cross‐validations, additionally with feature extraction and weighted model averaging,^2^ in the training sets (Table S6 and Figure S7A–C). After evaluating model performances in the validation sets based on selection fractions, the simplest models (selection fraction = 1) were selected, as the performances only mildly increased with selection fraction ≥.8 (Figure 1A–C; Figure S8A–C). The final MPM models for differentiating MDD versus BD, MDD versus SCZ, and BD versus SCZ consisted of 17, 20, and 17 proteins, and the AUROC values were .74, .82, and .78, respectively in the independent test sets (Figure 1A–C). Due to different analytical methods, the corresponding proteins differed with our previous study for discriminating MDD versus BD except for ITIH2.^2^ However, the current models were constructed with larger samples and expanded targets, and validated in an independent set; implying greater reproducibility. For each MPM model, the direction of each average coefficient corresponded to the alteration in expression (fold‐change) (Figure 1A–C). The MPM models had similar performances in differentiating MDD, BD, and SCZ with different subgroups (Figure S9A–F), all of the proteins were less influenced by psychotropic medication (Figure S10), and only few proteins showed associations with specific symptoms (Table S7). Particularly for BD, the proteins were unrelated to depressive or manic symptoms. The mass spectral information of proteins in the MPM models is presented in Table S8, and the alterations in the expression of the proteins is presented in Table S9 and Figure S11. There was no protein that overlapped in all three MPM models.

Symptom checklist‐based (SCLB) models were constructed by generalized linear models (GLMs). The models with the highest discriminatory power considering all combinations of the Symptom Checklist‐90‐Revised (SCL‐90‐R)^3^ dimensions, were selected (Table S10 and Figure S12A–C). Then, ensemble (ES) models were constructed by combining MPM and SCLB models through the stacking ensemble strategy.^4^ At last, clinician rater score‐based (CRSB) models were constructed by GLMs, combining the total scores of the Brief Psychiatric Rating Scale (BPRS),^5^ Hamilton Anxiety Scale (HAM‐A),^6^ Montgomery–Asberg Depression Rating Scale (MADRS),^7^ and Young Mania Rating Scale (YMRS)^8^ (Table S10). The discriminatory and diagnostic performances of the ES and CRSB models were overall comparable (Figure 2A–F and Figure S13A–C).

For 43 proteins from all MPM models, an integrated network comprising up to two networks was predicted (Table S11 and Figure 3). Diseases/functions associated with the network included cellular movement *(p = *7.87 × 10^‐21^–1.61 × 10^‐7^), cell‐to‐cell signalling and interaction (*p *= 9.14 × 10^‐10^–1.61 × 10^‐7^), immune cell trafficking (*p *= 2.3 × 10^‐12^–1.3 × 10^‐7^), neurological disease (*p *= 7.47 × 10^‐12^–8.17 × 10^‐8^), and psychological disorder (*p *= 6.09 × 10^‐12^–3.89 × 10^‐2^). Furthermore, the network was related to significant canonical pathways including complement and coagulation cascade dysregulation, neural signalling, and oxidative and inflammatory pathways, which has been replicated in previous studies (Figure 3).^2^, ^9^ Especially, reelin signalling was a significant canonical pathway, which is known to regulate neuronal migration and synaptogenesis in the brain, and has been linked to MDD, BD, and SCZ.^10^

**FIGURE 1:**
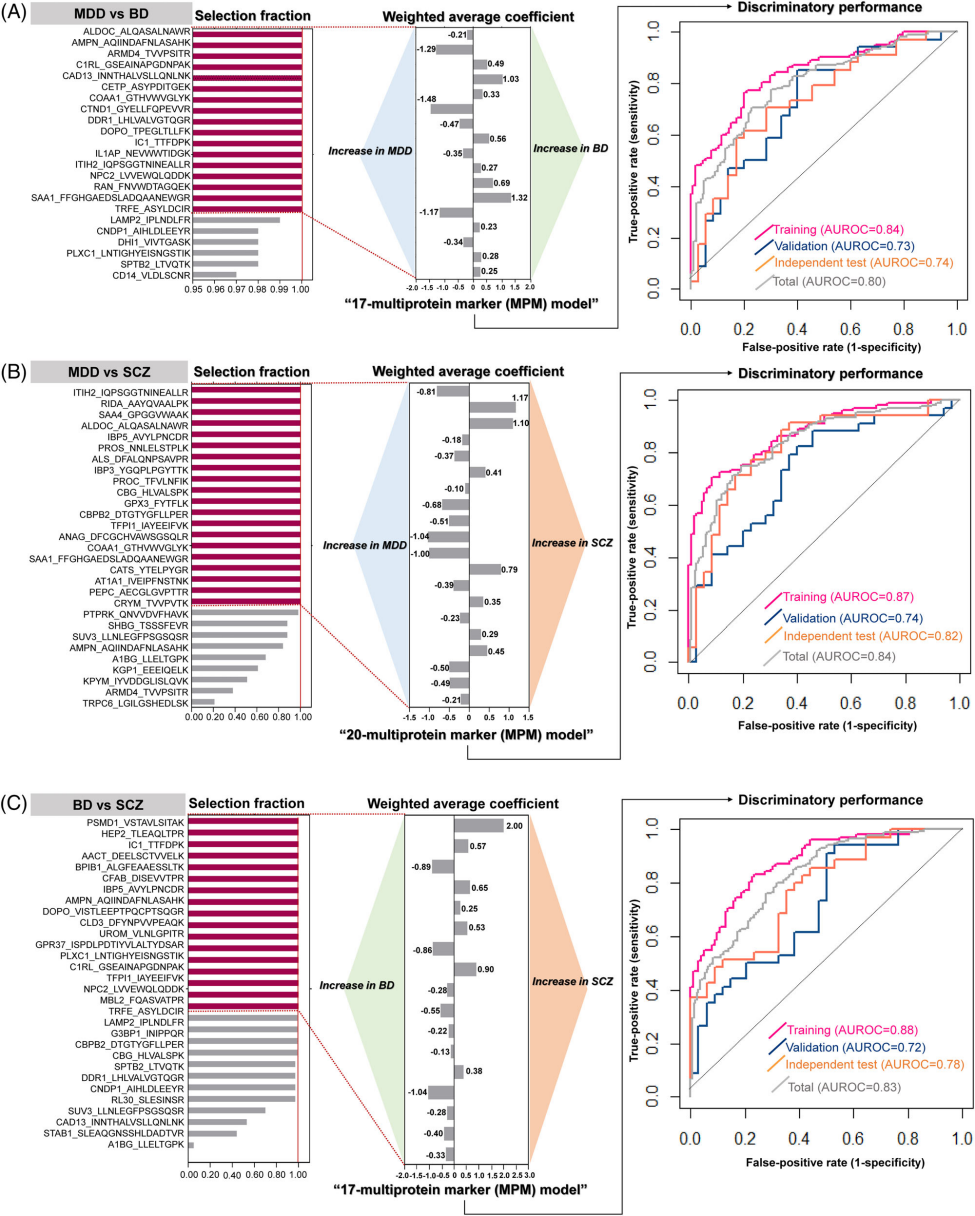
Development of multiprotein marker (MPM) models to discriminate disease types by machine learning. For each pairwise comparison, the selection fraction for proteomic candidate features (proteins), weighted average coefficient, and discriminatory performance are presented. The selected features (selection fraction = 1) in the MPM models are shown as pink bars. Weighted average coefficients corresponding to the selected features and their directions for disease types are presented. Discriminatory performance of each MPM model is presented as AUROC value in the training, validation, independent test, and total sets. Results of MPM models for (A) MDD versus BD, (B) MDD versus SCZ, and (C) BD versus SCZ. MDD, major depressive disorder; BD, bipolar disorder; SCZ, schizophrenia; MPM, multiprotein marker; AUROC, area under the receiver operating characteristics

**FIGURE 2:**
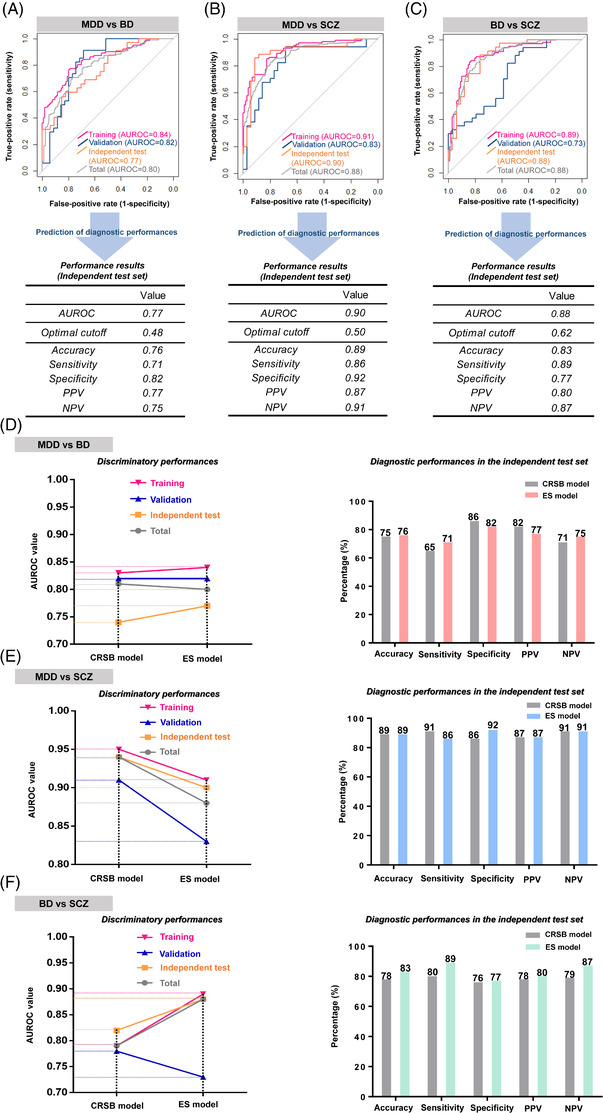
Discriminatory and diagnostic performances of ensemble (ES) models combining MPM and SCLB models and comparison of the performances between ES and CRSB models. For each ES model, discriminatory performance is presented as AUROC value in the training, validation, independent test, and total sets. Diagnostic performance with the independent test sets is presented as accuracy, sensitivity, specificity, PPV, and NPV at optimal cutoff (Youden index). Results for (A) MDD versus BD, (B) MDD versus SCZ, and (C) BD versus SCZ. For each pairwise comparison of groups, patterns of alterations in AUROC values are presented as line charts (left panel). Comparison of diagnostic performance in the independent test sets is presented as bar graphs (right panel). Results for (D) MDD versus BD, (E) MDD versus SCZ, and (F) BD versus SCZ. MDD, major depressive disorder; BD, bipolar disorder; SCZ, schizophrenia; ES, ensemble; MPM, multiprotein marker; SCLB, symptom checklist‐based; CRSB, clinician rater score‐based; AUROC, area under the receiver operating characteristics; PPV, positive predictive value; NPV, negative predictive value

**FIGURE 3:**
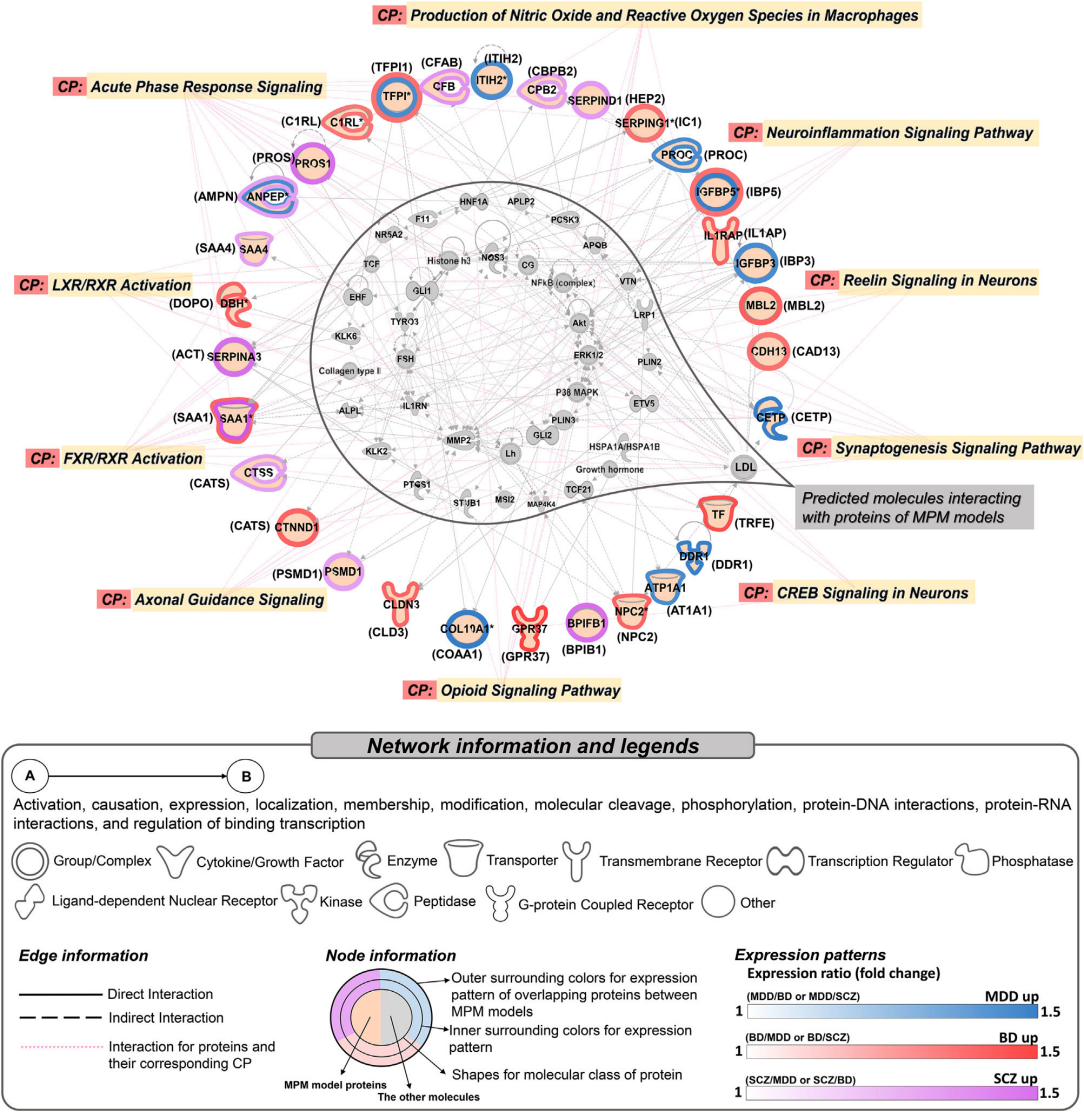
Integrated protein networks and associated canonical pathways for proteins in MPM models. Integrated protein networks and the corresponding canonical pathways were generated. Two networks with network score ≥20 were integrated. For edge information, direct and indirect interactions are presented by solid and dashed lines, respectively. Canonical pathways associated with proteins in the network are presented as dotted lines (light pink). Regarding node information, shapes signify the molecular class of proteins defined in the legend, and colours surrounding the nodes represent expression patterns for each disease type. Overlapping proteins between MPM models are denoted by an asterisk. Each protein is presented as a gene name and the corresponding protein entry in parentheses. Alterations in protein expression are presented as fold‐change for each disease type. MDD, major depressive disorder; BD, bipolar disorder; SCZ, schizophrenia; CP, canonical pathway; MPM, multiprotein marker

Through proteomic profiling, analytically stable plasma proteome (902 quantified proteins) were constructed in each pooled sample for the four groups (Table S12 and Figure S14A–D). Subsequently, 267 differentially expressed proteins (DEPs) with 4 clusters, 347 DEPs with 5 clusters, and 339 DEPs with 4 clusters were determined between MDD versus BD versus HC, MDD versus SCZ versus HC, and BD versus SCZ versus HC, respectively (Table S13). The DEPs that had consistent significance and expression patterns in both targeted proteomics and proteomic profiling were as follows; ITIH2 for the MPM model of MDD versus BD, TFPI1 and ITIH2 for MDD versus SCZ, and C1RL for BD versus SCZ. (Table S14; Figure 4A–C). The overall alterations in abundance of these 3 DEPs in each group is presented in Figure 4D. Further discussion of these key proteins is described in Supporting Information.

**FIGURE 4:**
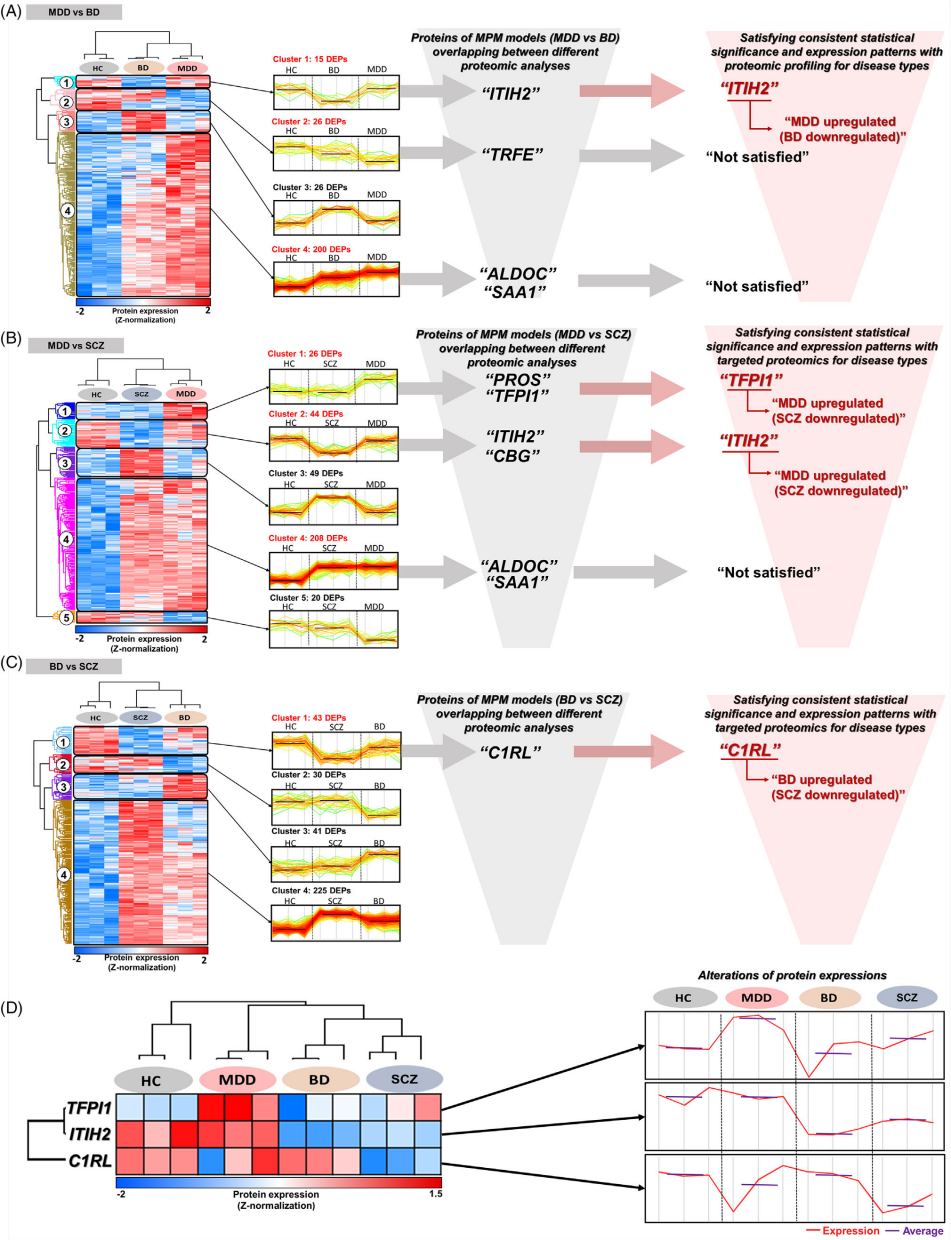
Proteins in MPM models with overlapping and consistent expression patterns between targeted proteomics and proteomic profiling. Clusters originating from DEPs of proteomic profiling analysis and their corresponding expression levels (represented as Z‐score) are presented as heatmaps. For each cluster, alteration in expression and the number of DEPs are presented. Clusters of proteins in the MPM models are indicated in red font. Overlapping proteins in MPM models with consistent expression pattern and information on their corresponding expression patterns are in wine‐coloured font. Results for (A) MDD versus BD, (B) MDD versus SCZ, and (C) BD versus SCZ. (D) Alterations in expressions of three selected proteins, which satisfied consistent statistical significance and expression pattern between targeted proteomics and proteomic profiling, between all disease groups and HCs are presented as heatmap and line graphs. Alterations in protein expression are indicated by a red line, and average protein expression for each group is signified by a purple line. TFPI1 was upregulated in MDD and SCZ but downregulated in BD compared with HCs (MDD > SCZ > HC > BD). ITIH2 showed no difference of expression between MDD and HC but was downregulated in BD and SCZ versus HC (MDD≈HC > SCZ > BD). C1RL showed no difference of expression between BD and HCs but was downregulated in SCZ and MDD compared with HCs (BD≈HC > MDD > SCZ). MDD, major depressive disorder; BD, bipolar disorder; SCZ, schizophrenia; HC, healthy control; MPM, multiprotein marker; DEP, differentially expressed protein

Our study has its limitations regarding sample size, the possibility of other potential confounders and proteomic targets including duration of the current episode, and medication dosage/duration, the cross‐sectional study design, biological interpretations of proteins in peripheral blood, and limited practicalness to clinical practice as a diagnostic tool (Supporting Information). Nevertheless, we demonstrated the viability of integrating proteomic and clinical data in discriminating MDD, BD, and SCZ. We developed MPM and ES models for each pairwise comparison of groups, reporting their potential in differentiating and diagnosing these disorders.

## CONFLICT OF INTEREST

The authors declare that there is no conflict of interest that could be perceived as prejudicing the impartiality of the research reported.

## Supporting information

SUPPORTING INFORMATION

## References

[1] GBD 2019 Mental Disorders Collaborators. Global, regional, and national burden of 12 mental disorders in 204 countries and territories, 1990–2019: a systematic analysis for the global burden of disease study 2019. Lancet Psychiatry. 2022;9(2):137‐150. 10.1016/S2215-0366(21)00395-3 35026139 PMC8776563

[2] Shin D , Rhee SJ , Lee J , et al. Quantitative proteomic approach for discriminating major depressive disorder and bipolar disorder by multiple reaction monitoring‐mass spectrometry. J Proteome Res. 2021;20(6):3188‐3203. 10.1021/acs.jproteome.1c00058 33960196

[3] Derogatis LR . SCL‐90‐R : administration, scoring & procedures manual‐II for the R (evised) version and other instruments of the psychopathology rating scale series. Clinical Psychometric Research. 1992: 1‐16.

[4] Džeroski S , Ženko B . Is combining classifiers with stacking better than selecting the best one?. Machine Learning. 2004;54(3):255‐273.

[5] Hafkenscheid A . Psychometric evaluation of a standardized and expanded brief psychiatric rating scale. Acta Psychiatr Scand. 1991;84(3):294‐300. 10.1111/j.1600-0447.1991.tb03147.x 1950632

[6] Hamilton M . The assessment of anxiety states by rating. Br J Med Psychol. 1959;32(1):50‐55. 10.1111/j.2044-8341.1959.tb00467.x 13638508

[7] Montgomery SA , Asberg M . A new depression scale designed to be sensitive to change. Br J Psychiatry. 1979;134:382‐389. 10.1192/bjp.134.4.382 444788

[8] Young RC , Biggs JT , Ziegler VE , Meyer DA . A rating scale for mania: reliability, validity and sensitivity. Br J Psychiatry. 1978;133:429‐435. 10.1192/bjp.133.5.429 728692

[9] Santa Cruz EC, Zandonadi FDS , Fontes W , Sussulini A . A pilot study indicating the dysregulation of the complement and coagulation cascades in treated schizophrenia and bipolar disorder patients. Biochim Biophys Acta Proteins Proteom. 2021; 1869(8): 140657. 10.1016/j.bbapap.2021.140657 33839315

[10] Jossin Y . Reelin functions, mechanisms of action and signaling pathways during brain development and maturation. Biomolecules. 2020; 10(6):964. 10.3390/biom10060964 PMC7355739 32604886

