# Supplementary material for: Integrating proteomic and clinical data to discriminate major psychiatric disorders: Applications for major depressive disorder, bipolar disorder, and schizophrenia
Source: Clin Transl Med. 2022 Jun 27;12(6):e929. doi: 10.1002/ctm2.929 (PMC9235346; doi:10.1002/ctm2.929)
Supplement: Supplementary file 1 — SUPPORTING INFORMATION [file CTM2-12-e929-s001.pdf]

Supplementary Information

Integrating proteomic and clinical data to discriminate major psychiatric disorders: Applications for major depressive disorder, bipolar disorder, and schizophrenia

Dongyoon Shin[1,¶], Sang Jin Rhee[3,4,¶], Daun Shin[3,4], Eun-Jeong Joo[5,6], Hee Yeon Jung[3,7,8], Sungwon Roh[9], Sang-Hyuk Lee[10], Hyeyoung Kim[11], Minji Bang[10], Kyu Young Lee[5,12], Se Hyun Kim[3], Jihyeon Lee[1], Yoseop Kim[1], Injoon Yeo[2], Yeongshin Kim[13], Jaenyeon Kim[1], Jun Soo Kwon[3,4,8], Kyooseob Ha[3,4,8], Yong Min Ahn[3,4,8,*], and Youngsoo Kim[1,2,*]

[1]Department of Biomedical Sciences, Seoul National University College of Medicine, Seoul, Republic of Korea; [2]Institute of Medical and Biological Engineering Medical Research Center, Seoul National University College of Medicine, Seoul, Republic of Korea; [3]Department of Psychiatry, Seoul National University College of Medicine, Seoul, Republic of Korea; [4]Department of Neuropsychiatry, Seoul National University Hospital, Seoul, Republic of Korea; [5]Department of Neuropsychiatry, School of Medicine, Eulji University, Daejeon, Republic of Korea; [6]Department of Psychiatry, Uijeongbu Eulji Medical Center, Eulji University, Seoul, Republic of Korea; [7]Department of Psychiatry, SMG-SNU Boramae Medical Center, Seoul, Republic of Korea [8]Institute of Human Behavioral Medicine, Seoul National University Medical Research Center, Seoul, Republic of Korea; [9] Department of Psychiatry, Hanyang University Hospital and Hanyang University College of Medicine, Seoul, Republic of Korea;

[10]Department of Psychiatry, CHA Bundang Medical Center, CHA University School of Medicine, Seongnam, Republic of Korea; [11]Department of Psychiatry, Inha University Hospital, Incheon, Republic of Korea [12]Department of Psychiatry, Nowon Eulji University Hospital, Seoul, Republic of Korea [13]Interdisciplinary Program of Bioengineering, Seoul National University College of Engineering, Seoul, Republic of Korea

[¶]These authors contributed equally to this work as co-first authors.

[*]These authors contributed equally to this work as co-corresponding authors.

Corresponding authors

*Youngsoo Kim, Ph.D.

Department of Biomedical Sciences & Institute of Medical and Biological Engineering Medical Research Center, Seoul National University College of Medicine, 103 Daehakro Seoul 30380, Republic of Korea. (Tel) +82-2-740-8073, (Fax) +82-2-741-0253, (E-mail) biolab@snu.ac.kr

*Yong Min Ahn, M.D., Ph.D.

Department of Psychiatry, Seoul National University College of Medicine/Department of Neuropsychiatry, Seoul National University Hospital/Institute of Human Behavioral Medicine, Seoul National University Medical Research Center, 101 Daehakro Seoul 30380, Republic of Korea. (Tel) +82-2-2072-0710, (Fax) +82-2-744-7241, (E-mail) aym@snu.ac.kr

Table of contents

Page S-4 ~ S-20: Supplementary Methods

Page S-21: Supplementary Discussion

Page S-22 ~ S-27: Supplementary References

Page S-28 ~ S-41: Supplementary Figures (Figure S1-S14)

Page S-42 ~ S-100: Supplementary Tables (Table S1-S14)

Supplementary Methods

Clinical samples

The study population comprised 515 patients with major psychiatric disorders (171 SCZ, 170 BD, and 174 MDD) and 160 HCs who were enrolled from August 2018 to December 2020. The ages ranged from 19 to 65 years. The study included 90 BD, 90 MDD and 90 HC patients from our previous study [1], but all proteomic analyses of their plasma samples were newly performed for this study regardless of the previous study. The BD patients consisted of 75 BD-I (Bipolar I disorder), 84 BD-II (Bipolar II disorder), and 11 BD-not otherwise specified (NOS). BD-I, formerly known as manic-depressive disorder, is characterized by episodes of mania and depression. BD-II entails episodes of depression and hypomania that never reach the severity of mania. BD-NOS is known as subthreshold BD or a type that does not fulfill the criteria of BD-I or BD-II.

Patients were recruited from Seoul National University Hospital (SNUH); Nowon Eulji Medical Center, Eulji University; Seoul Metropolitan Government Seoul National University Boramae Medical Center; Hanyang University Seoul Hospital; Inha University Hospital; and Cha University Bundang Medical Center. HCs were recruited from SNUH through an advertisement. The diagnoses of the patients were made per the Diagnostic and Statistical Manual of Mental Disorders, Fifth Edition (DSM-5) and were confirmed using the Mini-International Neuropsychiatric Interview (MINI). Patients had to have a Clinical Global Impression - Severity $\geq$ 3 to participate. HCs had to have no psychiatric diagnosis, based on the MINI, and no known psychiatric family history among second-degree relatives.

Patients and HCs were excluded per the following criteria: use of any anti-

inflammatory analgesic, including nonsteroidal anti-inflammatory drugs (NSAIDs) and steroids (acetaminophen was permitted) in the past 2 weeks; a history of neuromodulation [electroconvulsive therapy (ECT), transcranial magnetic stimulation (TMS), transcranial direct current stimulation (tDCS), or deep brain stimulation (DBS)] or neurosurgery; central nervous system diseases, including epilepsy, stroke, parkinsonism, and meningitis; cancer; tuberculosis; current pregnancy/lactation; a history of substance abuse other than nicotine, caffeine, and alcohol; intensive psychotherapy for the past 2 months; predicted intellectual disability; and difficulty interpreting Korean. Most of the exclusion criteria were based on previous reports of certain conditions and diseases and their associations with altered protein expression [2-10]. Those who had recently received neuromodulation or psychotherapy were excluded to confine the effects of treatment to psychotropic medications.

The study was performed in accordance with the latest version of the Declaration of Helsinki. The study design was reviewed by the institutional review boards of Seoul National University Hospital (IRB no. 1806-106-951) and all hospitals that participated. Informed consent was obtained from each participant.

Plasma samples from each subject were collected in a 6-mL ethylenediaminetetraacetic acid (EDTA) tube (ref 367863, Becton, Dickinson and Company, Trenton, NJ) and centrifuged at 1100−1300g for 10−15 min at room temperature or 4°C. The supernatant was collected and stored in Eppendorf tubes at -70°C or below until use.

Demographics and clinical features

The demographics that we considered for patients and HCs were age, sex, body mass index (BMI), current smoking status, current exercise status, current alcohol use, blood

collection time, and fasting time. Age and BMI were analyzed as continuous variables, and sex (men/women), current smoking status (yes/no), current exercise status (yes/no), current alcohol use (yes/no), blood collection time (AM, PM), and fasting time (< 8 hours, ≥ 8 hours) were analyzed as dichotomous variables. Current exercise status was based on the World Health Organization (WHO) recommendations for moderate-intensity physical activity for at least 30 minutes, once per week [11]. Current alcohol use was defined as at least 1 drink, once per week.

Symptom severity for patients and HCs was assessed using the Brief Psychiatric Rating Scale (BPRS) [12], Young Mania Rating Scale (YMRS) [13], Montgomery-Asberg Depression Rating Scale (MADRS) [14], and Hamilton Anxiety Scale (HAM-A) [15]. The self-reported scale in the analysis was the Symptom Checklist-90-revised (SCL-90-R) [16]. Because bipolar disorder has different mood states, those whose YMRS scores were over 12 points were categorized as having current hypomanic/manic/mixed symptoms[17] for the subgroup analyses on BD.

For patients, medication use was analyzed as a dichotomous variable for each use of antipsychotics (APs), lithium/anticonvulsants (L/ACs), antidepressants (ADs), and benzodiazepines/hypnotics (BZDs/HNTs). The chronicity of the disease or medication was assessed as the duration from first onset (years) (DFO) and duration from first medication (years) (DFM); these parameters were analyzed as continuous variables.

Plasma sample preparation for targeted and proteomic profiling analysis

For the targeted proteomic analysis, plasma samples were thawed on ice and centrifuged at 10,000 *g* for 10 min at 4°C. Supernatants were transferred to fresh tubes and vortexed. For each sample, 44 μL was diluted 1:4 with MARS buffer A (Agilent Technologies,

Santa Clara, CA, USA) and passed through 0.22-μm Spin-X filters (Corning Costar, NY, USA). A volume of 176 μL of buffer A was added to each sample, and each diluted sample was centrifuged through a 0.22-μm filter (12,000 *g*, room temperature). Each plasma sample was depleted of 6 high-abundance human plasma proteins (albumin, IgG, IgA, transferrin, haptoglobin, and antitrypsin) using a multiple affinity removal system (MARS) column (Hu-6HC, 4.6 × 100 mm, Agilent Technologies, Santa Clara, CA, USA) that was loaded onto a high-performance liquid chromatography (HPLC) system (Shimadzu Co, Kyoto, Japan). A total of 200 μL was injected for each sample.

Depleted plasma samples were concentrated by centrifugal filtration for 6 hours at 4°C using a 3000-Da molecular weight cutoff (MWCO) filter (Amicon Ultra-4 3K, Millipore, Burlington, MA, USA). The concentrated proteins of individual samples were then quantified by bicinchoninic acid assay (BCA assay) using the Pierce™ BCA Protein Assay Kit (Thermo Scientific, Rockford, IL, USA). A 6-point standard curve was generated by serially diluting an initial concentration of 2 mg/mL BSA by a factor of 2. Standards and samples were placed on a 96-well plate, and a mixture of copper solution and BCA solution (1:50) was added.

The proteins were digested with RapiGest surfactant and trypsin. Next, 40 μL 0.2% RapiGest, 20 mM dithiothreitol (DTT), and 100 mM ABC buffer, pH 8.0 was added to the 40-μl plasma samples, adjusted with HPLC-grade water to a 100-μg digestion. After 1 hour in 60°C, 20 μL 100 mM iodoacetamide (IAA) was added, and the samples were incubated in the dark for 30 min at room temperature. Next, the samples were incubated for 4 hours at 37°C after trypsin, dissolved in 50 mM ABC, pH 8.0, was added (sequencing-grade modified, Promega, Madison, WI, USA). Then, 10% formic acid was added to the samples to 1% to stop the enzymatic reaction and incubated for 30 min at 37° to hydrolyze RapiGest surfactant in the

acidified samples. After centrifugation at 15,000 rpm at 4°C for 1 hour, the cleaved RapiGest surfactant precipitated, and the supernatant was transferred to a clean tube.

The plasma peptide samples (the transferred supernatant for each sample) were spiked with crude stable isotope-labeled internal standard (SIS) peptide, in which a C-terminal lysine or arginine was heavy-isotope-labeled ($^{13}C_6{}^{15}N_2$ or $^{13}C_6{}^{15}N_4$) [purity: crude (>70%), JPT, Berlin, Germany]. A total of 675 plasma samples were distributed among 5 preparation batches (batches 1–5), because they were collected at various time points. In each preparation batch, the samples were randomly distributed and assigned identification numbers to blind the researchers throughout the sample preparation.

For the proteomic profiling analysis, the remaining depleted individual plasma samples after the targeted proteomic preparation were pooled for each group. In addition, equal amounts of pooled plasma samples for each group were integrated to generate a peptide spectral library. A total of 5 pooled samples were centrifuged at 15,000 rpm for 20 min at 4°C, and 100 µl of the supernatant was placed into a new tube. The supernatant was dissolved in 300 µL lysis buffer (4% SDS; 0.1M TEAB, pH 8.5; 2 mM TCEP). Following sonication, the samples were incubated at 100°C for 30 min. Protein concentrations were measured using a BCA reducing agent-compatible kit (Thermo Fisher Scientific, Waltham, MA, USA).

Protein digestion was performed using a combination of acetone precipitation and filter-aided sample preparation (FASP) [18]. Before the digestion, 300 µg of extracted protein was precipitated with cold acetone at a buffer:acetone ratio of 1:5 and incubated at -20°C for 18 hours. Next, the pellet was washed with 500 µL cold acetone, centrifuged at 15,000 rpm for 15 min, and air-dried for 2 hours. The proteins that had precipitated were dissolved in 35 µL denaturation buffer (4% SDS and 100 mM DTT in 0.1 M TEAB, pH 8.5). After being heated at 100°C for 35 min, the denatured proteins were loaded onto 30-kDa spin filters (Merck

Millipore, Darmstadt, Germany). The buffer was exchanged 3 times with urea solution (8 M urea in 0.1 M TEAB, pH 8.5).

After the SDS was removed, cysteine residues were treated with alkylation buffer (50 mM IAA, 8 M urea in 0.1 M TEAB, pH 8.5) for 1 hour at room temperature in the dark. Urea buffer was exchanged with TEAB buffer (40 mM TEAB, pH 8.5). The proteins were digested with trypsin (enzyme:substrate ratio [w/w] of 1:50) and 4% ACN at 37°C for 18 hours. The digested peptides were eluted by centrifugation, and their concentrations were measured, based on tryptophan fluorescence emission at 350 nm, using an excitation wavelength of 295 nm [19]. Equalized amounts of peptides were then set aside for label-free quantification. The measured peptides were acidified with 10 μL 10% TFA and desalted on homemade C18-StageTip columns [20]. The desalted peptides were then lyophilized on a speed-vacuum centrifuge and stored at -80°C.

To increase the number of identified proteins, each pooled plasma sample was fractionated by modified Stage-tip-based high-pH peptide fractionation [18, 21]. The lyophilized peptides were dissolved in 200 μL loading buffer (15 mM ammonium hydroxide solution, pH 10, and 2% acetonitrile) and separated on a pipette-based C18 RP microcolumn. The column was constructed by plugging the bottom of a 200-μL transparent pipette tip with a C18 Empore disk membrane (3M, Bracknell, UK) and packing the tip with POROS 20 R2 resin. The plugged tip was rinsed 3 times with 100 μL 100% methanol and then 3 times with 100 μL 100% acetonitrile (ACN). The column was then conditioned with 100 μL loading buffer using a syringe. The peptides were loaded onto the column at pH 10. An ACN gradient of 2, 5, 7.5, 10, 12.5, 15, 17.5, 20, 22.5, 25, 27.5, 30, 32.5, 35, 40, 50, 60, 70, 80, and 100% was used to elute 20 fractions, which were collected into 6 tubes discontinuously to distribute eluents of varying

hydrophobicity. These 6 fractions were lyophilized in a speed-vacuum centrifuge and stored at -80℃.

Determination of detectable and quantifiable targets

For the targeted proteomic analysis, integrated protein targets for MDD, BD, SCZ, and HC were selected. Three sources were compiled to generate the list of integrated candidate targets: 1) new targets for psychiatric disorders (MDD, BD, and SCZ), 2) established targets for mood disorders (MDD and BD), and 3) laboratory-established targets.

New candidates for psychiatric disorders were collected from 5 databases on psychiatric disorders (MDD, BD, and SCZ): PsyGeNET (http://www.psygenet.org), Schizophrenia Gene Resource 2 (SZGR2) (https://bioinfo.uth.edu/SZGR/), Laboratory of Neurophenomics (http://www.neurophenomics.info/), Comprehensive Database for Schizophrenia (SZDB2) (www.SPRdb.org), and The Stanley Neuropathology Consortium Integrative Database (SNCID) (http://sncid.stanleyresearch.org) [22-25]. As a result, 8081 genes were integrated as initial targets. The Human Blood Proteins Atlas and Plasma Proteome Database (PPD) were used to filter and select targets that are detectable in blood [26, 27]. In total, 1462 blood-detectable proteins were selected.

To examine targets that had matching MS/MS spectra and unique peptides, 8 MS/MS spectral libraries from various institutes—the Institute for Systems Biology (https://www.systemsbiology.org), National Institute of Standards and Technology (https://www.nist.gov), and the SWATHAtlas database (www. SWATHAtlas.org)—were used. In total, 407 proteins, corresponding to 407 unique peptides with top 10 transitions per each peptide, were selected. The established candidates of mood disorders (ie, MDD and BD) were drawn from our previous study [1]. The laboratory-established targets included proteins that have

been approved by the US Food and Drug Administration (FDA) and designated as laboratory developed tests (LDTs) and proteins that have been developed in our previous research, unrelated to psychiatric disorders. In total, 1667 proteins/2283 peptides were merged as integrated candidate targets.

To examine targets that were detected and quantified in blood samples of psychiatric disorders, LC-MRM-MS analysis was performed on a pooled plasma sample that consisted of 50 HCs, 50 MDD, 50 BD, and 50 SCZ samples. Targets were considered to be detectable and quantifiable if: 1) at least 5 transitions for LC-MRM-MS were observed; 2) they had the same elution patterns within the predicted retention time (RT) (±5 min); 3) the ratio of transition peaks was obtained as in the spectral library (dot product ≥ 0.6); 4) RTs and dot products were equal between light and heavy peptides; and 5) there were transitions that had the highest intensity based on the rank of intensity and that were filtered by AuDIT for selecting interference-free transitions [28].

Regarding the 2283 unique peptides that corresponded to 1667 proteins and the relevant 2283 stable isotope-labeled internal standard (SIS) peptides, LC-MRM-MS analysis was performed to measure the detectability of the integrated candidate targets. A total of 642 target peptides were selected as being quantifiable.

LC-MS analyses for targeted proteomics and proteomic profiling

For our targeted proteomic analysis, target peptides were analyzed by targeted LC-MRM-MS on an Agilent 6490 triple quadrupole (QQQ) mass spectrometer (Agilent Technologies, Santa Clara, CA, USA) that was equipped with a Jetstream electrospray source that was coupled to a 1260 Infinity HPLC system (Agilent Technologies, Santa Clara, CA,

USA). Solvents A and B for the HPLC consisted of 0.1% formic acid/water (v/v) and 0.1% formic acid/acetonitrile (v/v), respectively. Glass vials of the samples in the autosampler were maintained at 4°C.

A total of 40 μl of digested sample was injected into a guard column (2.1 × 15.0 mm, 1.8 μm, 80 Å) (Agilent Technologies, Santa Clara, CA, USA). Online desalting was conducted with the effluent toward waste at 50 μl/min for 10 min in 3% solvent B, consisting of 0.1% formic acid/acetonitrile (v/v), at 40°C. After the position of valve was switched, the desalted sample was transferred to the analytical column (0.5 × 35.0 mm, 3.5 μm, 80 Å) (Agilent Technologies, Santa Clara, CA, USA) in 3% solvent B at a flow rate of 40 μL/min for 5 min. The analytical column was heated and maintained at 40°C by an oven.

The total run time per LC-MRM-MS analysis was 70 min. Approximately 10 μg of digested peptides was injected per LC-MRM-MS run. The peptides were separated on the column and eluted with a linear gradient of 3% to 35% acetonitrile (ACN) with 0.1% formic acid (FA) for 50 min at 40 μL/min. The mass spectra were generated in positive ion mode, based on the following parameters: 2500 V ion spray capillary voltage, 2000 V nozzle voltage, 5 V cell accelerator voltage, 200 V delta EMV, and 380 V fragmented voltage. The drying gas was sprayed at 15 L/min at 250°C, and the sheath gas flow was 12 L/min at 350°C. Collision energy (CE) was optimized by adding the intensities of individual transitions that resulted in the largest peak area. The default value for CE was calculated as follows: CE = 0.031 × (m/z of precursor) + 1 for doubly charged precursor ions and CE = 0.036 × (m/z of precursor) – 4.8 for triply charged ions. Five additional steps of adding or subtracting 2 V on either side of the default CE value were predicted for determining the optimized CE.

Before the analysis of individual plasma samples, SIS peptides that corresponded to the determined targets were pooled and analyzed to evaluate their RTs. The RTs of the SIS peptides

were compared with those of endogenous target peptides by spiking the pooled mixture of SIS peptides (10, 100, or 1000 fmol of heavy peptides corresponding to each target) with 100 fmol of a heavy peptide of beta-galactosidase (β-gal), which was used to address technical variations across LC-MRM-MS runs and between preparations by different researchers. Subsequently, the final targets were quantified in individual blood samples, which were listed randomly in blocked batches with an identification number for each sample. LC-MRM-MS analysis was performed once per sample (1 replicate for each sample).

For the proteomic profiling, the prefractionated peptides were analyzed on an LC-MS system with an Easy-nLC 1000 (Thermo Fisher Scientific, Waltham, MA, USA) that was equipped with a nanoelectrospray ion source (Thermo Fisher Scientific, Waltham, MA, USA) and coupled to a Q-Exactive mass spectrometer (Thermo Fisher Scientific, Waltham, MA, USA), as described in our previous studies [18]. The peptide samples were separated on a 2-column system, comprising a trap column (75 μm I.D. x 2 cm long, 3-μm Acclaim PepMap100 C18 beads) (Thermo Fisher Scientific, Waltham, MA, USA) and an analytical column (75 μm I.D. x 50 cm long, 3-μm ReproSil-Pur C18-AQ beads) (Thermo Fisher Scientific, Waltham, MA, USA). Lyophilized peptide samples were dissolved in Solvent A (0.1% formic acid water and 2% ACN) prior to injection.

The peptides were separated on a 180-min linear gradient, ranging from 6% to 40% Solvent B (100% ACN and 0.1% formic acid) for all peptide samples. The spray voltage was set to 2.2 kV in positive ion mode, and the heated capillary temperature was set to 275°C. Mass spectra were collected in data-dependent acquisition (DDA) mode by top 15 method. Xcaliber (version 2.5) was used to set the mass spectrometer parameters as follows: mass range of 350–1700 m/*z,* resolution of 70,000 at 200 m/z for detected precursor ions, automatic gain control (AGC) at 1 x 10^6, isolation window for MS2 at 1.2 m/z, automatic gain control (AGC) for MS2

at 2 x 10^5, higher-energy collisional dissociation (HCD) scans at a resolution of 17,500, and stepped normalized collision energy (NCE) of 27, 30, and 33. The maximum ion injection time (maximum IT) for the full MS and MS2 scans was 20 ms and 80 ms, respectively. Dynamic exclusion with an exclusion time of 30 s was used. All samples were analyzed in 3 technical replicates.

Processing of targeted proteomic data

The raw data from the LC-MRM-MS analysis were processed in Skyline (version 19.1.0) (MacCoss Lab, Seattle, WA, USA) to calculate the peak area values of the transitions. Peptide-transition peaks were investigated using peak integrations that were performed manually. Peptide quantification was based on the relative abundance of the endogenous and SIS peptide transitions—the relative abundance of the transition pairs (Q1 and Q3) was determined by the ratio of endogenous (Light) to SIS (Heavy) peptide peak areas, reported as the peak area ratio (PAR), or Light/Heavy (L/H) ratio.

After the manual peak integrations, unstable targets were examined across individual samples. A total of 54 unstable targets with low intensity (intensity < 1000), unequal RTs between light and heavy peptide, and skewed peaks were excluded. Subsequently, PAR values of 588 targets across 675 individual samples were generated and normalized by the area of heavy β-gal peptide to reduce the technical variability from the sample preparations between researchers. The normalized PAR values were scaled by $\log_2$ (x+0.001). Potential batch effects between sample preparation batches (batches 1-5) were corrected with the Combat algorithm—a nonparametric adjustment for reducing batch effects using an empirical Bayes framework—using the R package proBatch [29]. Then, the batch-corrected MRM-MS data were distributed into training, validation, and independent test sets for each pairwise comparison of

groups and a reference set of HCs. In addition, the total set was defined as the combination of the training, validation, and independent test sets.

Sequence-based search for plasma proteome

Proteome Discoverer, version 2.2 (Thermo Fisher Scientific, Waltham, MA, USA) was used to analyze the resulting RAW files from Q-Exactive. The full-MS and MS/MS spectra search was conducted using the SEQUEST-HT algorithm against a modified version of the Uniprot human database (December 2014, 88,717 protein entries; http://www.uniprot.org). The database search was performed using the target-decoy strategy.

The search parameters were as follows: a precursor ion mass tolerance value of 20 ppm (monoisotopic mass); a fragment ion mass tolerance value of 0.02 Da (monoisotopic mass); full enzymatic digestion with trypsin (after KR/−) and up to 2 missed cleavages; 57.02 Da for cysteine residues with carbamidomethylation; dynamic modification values of 42.01 Da for protein N-terminal acetylation; and 15.99 Da for methionine oxidation. A false discovery rate (FDR) of less than 1% at the peptide and protein levels was used as the confidence criterion. Peptides were mapped and linked across the inputted multiple MS files w with the "Feature Mapper" node to increase in the number of identified proteins. In this node, the maximum RT shift and mass tolerance of the chromatographic alignment were 30 min and 10 ppm, respectively. Proteins were quantified and normalized by calculating the relative intensities for peptide-spectrum matches (PSMs)—peptides and proteins that were based on precursor quantification—and by using the normalization mode of the total peptide amount in the "Precursor Ions Quantifier" node.

Analysis of demographics and clinical features of study subjects

Demographic and clinical differences between patient groups and HCs were analyzed by one-way analysis of variance (ANOVA), followed by post hoc analysis by Tukey's HSD (honest significant difference) test for continuous variables and chi-squared test for dichotomous variables.

Determination of targeted proteomic candidate features

Initially, targets that represented with the final adjusted PARs corresponding to the range of raw PAR $\leq$ 0.05 and raw PAR $\geq$ 100 in over 5% of individuals in each training set were excluded.

Proteomic features (proteins) that had low relations with the covariates—demographics, medication use, and chronicity of disease and medication—were selected as follows. Initially, independent student's *T*-test for dichotomous covariates and Pearson's correlation analysis for continuous covariates were performed to analyze the association between these covariates and proteomic candidate features (proteins). Significant covariates were further controlled by univariate analysis of covariance (ANCOVA) to determine whether the features correlated with disease type or these covariates. Proteomic candidate features (proteins) with a significant relationship only with the covariates and not disease type were excluded.

Subsequently, proteomic candidate features (proteins) that had a variance inflation factor (VIF) >5 relative to other features were eliminated. AUROC analysis was performed for the determined proteomic feature (protein) candidates, and fold-change values were calculated by subtracting the average batch-corrected PAR value of one group from that of another for

each pairwise comparison of groups. The resulting proteomic candidates (proteins) were used to develop the MPM models.

Determination of clinical candidate features

Clinical candidate features (dimensions of SCL-90-R) were determined by manual selection, based on the optimal combinations of variables with the highest discriminatory performance by generalized linear models (GLMs) for binary data in the training sets for each pairwise comparison of groups. The resulting variables were used to develop symptom checklist-based (SCLB) models. Further, all of the clinician rater total scores (BPRS, MADRS, YMRS, and HAM-A) were used to develop clinician rater score-based (CRSB) models using the same method.

Development of models

For MPM models, the least absolute shrinkage and selection operator (LASSO) was used to decrease overfitting by simultaneous shrinkage of the coefficients and model selection. LASSO regression was conducted using the R package glmnet [30]. Five-fold crossvalidation was used to determine the optimal value of the shrinkage parameter, lambda, which yielded the most regularized model. LASSO with 5-fold crossvalidation (100 repetitions, resulting in 100 models) was applied to the training sets for each pairwise comparison of groups. Feature extraction and model averaging based on previous studies [1, 31] were performed to develop the final MPM model as follows.

For each model generated from 100 repetitions, the bias-corrected version of the AIC (Akaike's information criteria) was calculated. The Akaike weight (w) was calculated with the bias-corrected version of AIC to serve as an indicator for model probability; the probability of

each model being the best model [1, 31]. The proportion of each proteomic candidate feature (protein) that was selected across the 100 models was calculated and defined as the selection fraction, assigned a value from 0 to 1. The selection fraction was used to assess the relative significance of the features. Proteomic features (proteins) that had a selection fraction = 1 or ≥ 0.8 were combined, in each pairwise comparison of groups—feature extraction. Subsequently, the weighted coefficients of the extracted features were averaged across 100 models by weighted model averaging—model averaging. Specifically, the coefficients were calculated by summing the product of the Akaike weight and coefficient of each factor, for each model. More specific methods are described in our early study [1]. The 2 generated MPM models that originated from the 2 conditions (selection fraction = 1 or ≥ 0.8) for each pairwise comparison were evaluated in the validation sets. Then, considering the number of combined features of the models and discriminatory performance in the validation sets between 2 models, the final models were generated, and their performance was validated in independent test sets.

Pearson's correlation analysis was performed to analyze the association between clinician rater scores and self-reported scales with the proteomic candidate features (proteins) of the MPM models. ANCOVA was also performed for significant correlations to determine whether the features were associated with disease type or current symptoms.

The clinically based SCLB and CRSB models were developed using GLMs for binary data in the R package e1071 [32]. The determined candidate features were fitted in the "glm" function. The ES models MPM and SCLB were combined, based on the stacking ensemble strategy [33], generating new prediction values for ES models by combining prediction values (represented as the probability) of the MPM and SCLB models in the training sets.

Discriminatory and diagnostic performance of the models

The discriminatory performance of the models was evaluated by examining their AUROC values using the R package pROC [34]. AUROC values for a model's performance were defined as follows: 0.5–0.6 = fail; 0.6–0.7 = poor; 0.7–0.8 = fair; 0.8–0.9 = good; 0.9–1 = excellent [35]. The optimal cutoff of the ES models for each pairwise comparison of groups was determined per the Youden Index as follows: J = max (Sensitivity + Specificity – 1) [36]. Diagnostic parameters—sensitivity, specificity, accuracy, positive predictive value (PPV), and negative predictive value (NPV)—were calculated at the optimal cutoff.

Alterations in expression of proteins in the MPM models

Changes in expression of proteins in the MPM models were examined in the targeted proteomic data of all study populations (174 MDD, 170 BD, 171 SCZ, and 160 HC). ANOVA was performed for each pairwise comparison—1) MDD versus BD versus HC, 2) MDD versus SCZ versus HC, and 3) BD versus SCZ versus HC. Subsequently, post hoc analysis for each specific pairwise comparison between groups was performed using Tukey's HSD.

Medication effects on the proteins of MPM models

Medication effects of AP, L/ACs, AD, and BZD/HNT on the proteins of MPM models were examined in all patients (174 MDD, 170 BD, and 171 SCZ). Percent variations of the proteins regarding the medications were analyzed using the R package variancePartition [37]. Less than 10% of variation was applied as the criterion of whether the proteins were affected by the medications based on a previous study [38].

Processing of proteomic profiling data

Each of the 4 groups had 3 technical replicates, resulting in 3 normalized protein abundance values per group; normalized values greater than 0 were considered to be valid. Proteins with all 3 valid values within a group and 1 or more unique peptides were determined to be quantified proteins for the following statistical analysis.

After the normalized values were transformed by $\log_2$ (x+0.001), the technical variation was measured by crosscorrelation analysis (Pearson’s correlation), and principal component analysis (PCA) was performed to examine the segregation of the groups. Then, ANOVA was used to identify differentially expressed proteins (DEPs) between patient groups and HCs. Subsequently, post hoc analysis for each pairwise comparison between groups was performed by Tukey's HSD. After z-score normalization of the levels of the DEPs, k-means hierarchical clustering analysis was performed with the default settings. Fold-change values were calculated by subtracting the average normalized amount of protein in 1 group by that of another for each pairwise comparison of groups.

Bioinformatics analysis

The integrated protein network with a score ≥ 20 was examined by Ingenuity Pathway Analysis (IPA, QIAGEN, Hilden, Germany), based on the proteins that were included in the MPM models for each comparison of groups, with matched gene names. Subsequently, diseases/functions and canonical pathways that were associated with the integrated networks were evaluated. The analytical algorithms in IPA use lists of proteins to predict protein

networks and their corresponding diseases/functions and canonical pathways, which were identified by two-tailed Fisher's exact test.

Statistical analysis

The statistical analyses for the clinical and targeted proteomic data and model development were performed using SPSS (version 25.0, IBM, Armonk, NY, USA) and R (version 4.1.0), and Perseus (version 1.5.8.5) was used for the proteomic profiling data. The data in all statistical analyses were deemed to have a normal distribution per the central limit theorem, because the number of samples for each group was over 30, obviating the need for the normality test. The statistical tests were two-tailed, and P-value < 0.05 was considered to be statistically significant. In the bioinformatics analysis, P-value < 0.05 was statistically significant for the integrated network analysis by IPA.

Supplementary Discussion

Compared with our early study, ITIH2 was significantly increased in MDD versus BD and was a key proteomic feature that improved their discrimination [1]. In comparing BD and SCZ, HEP2 was upregulated and was also a significant protein in Santa Cruz et al. (2019), although it decreased in the latter study [39]. Smirnova et al. (2021) discovered AACT in the profiling data on SCZ but not BD, as in our study, in which AACT was higher in SCZ versus BD [40]. The discrepancy with Santa Cruz et al (2019) is likely due to a difference in proteomic approaches, based on alternate study designs, MS-based quantitation methods, data processing methods, and the number of samples and composition of subjects. Thus, integrating profiling data allowed us to focus on more stable and reliable key proteins. TFPI1 differed significantly

between each group. This protein is important in inhibiting the extrinsic pathway of blood coagulation [41]. Its function in cancer has been examined [42], but its ability to discriminate between major psychiatric disorders is novel. ITIH2 was upregulated in MDD and HCs, compared with BD and SCZ. As discussed, compared with our previous study, ITIH2 was a significantly consistent key protein in differentiating MDD and BD [1]. ITIH2 is a serine protease inhibitor with anti-inflammatory functions [43]. An earlier study reported that ITIH2 levels decrease in MDD versus HC, but the study sample was smaller compared with our study [44]. C1RL was upregulated in BD versus SCZ. In a recent report, the level of C1RL at age 12 was higher in those who experienced psychotic symptoms at age 18 compared with HCs [45]. However, C1RL was generally lower in SCZ in our study. The chronicity could explain this discrepancy, because complement pathway activity might differ before and after the onset of psychotic symptoms.

There were several limitations of this study. First, despite the expanded sample size compared with our previous work, it remained a major limitation due to the difficulty in collecting appropriate patients and HC samples. In addition, there were potential confounders that could have affected our results. Specifically, we categorized medication use dichotomously, and specific dosages and durations of medication were not controlled for. This was not enough to refrain from medication effects on plasma proteins, thus requiring analysis of plasma samples of first-episode and drug-naive patients. Moreover, although we attempted to eliminate proteomic features that were related to the covariates, other covariates might have influenced the discriminatory and diagnostic performance. Validation in a larger sample with various covariate values is needed in the future. Second, the interpretation for causality is limited, because the study was cross-sectional. Thus, longitudinal studies are required to observe diagnostic alterations of MDD, BD, and SCZ, and serial quantitation of proteins at various time points in an individual could facilitate discrimination between proteins that are related to the

trait and state of the disorders. Third, because the results of bioinformatics analysis were deduced from peripheral blood proteins, and not the central nervous system (CNS), the results should be interpreted with caution. Additional experimental validation of the proteins of MPM models should be performed to examine whether the plasma proteins are correlated with the CNS. Fourth, although discrimination and diagnostic potentials of the developed models were proposed, they are currently premature to serve as ideal diagnostic tools because of several issues such as cost, time for labor, and usability. Finally, the potential of our LC-MS-based proteomic approaches should be validated using conventional immunoassays such as ELISA in order to evaluate consistency between different analytical platforms.

Supplementary References

1. Shin D, Rhee SJ, Lee J, et al. Quantitative Proteomic Approach for Discriminating Major Depressive Disorder and Bipolar Disorder by Multiple Reaction Monitoring-Mass Spectrometry. *J Proteome Res*. Jun 4 2021;20(6):3188-3203. doi:10.1021/acs.jproteome.1c00058
2. Jabbi M, Arasappan D, Eickhoff SB, Strakowski SM, Nemeroff CB, Hofmann HA. Neuro-transcriptomic signatures for mood disorder morbidity and suicide mortality. *J Psychiatr Res*. Aug 2020;127:62-74. doi:10.1016/j.jpsychires.2020.05.013
3. Najjar S, Pearlman DM, Alper K, Najjar A, Devinsky O. Neuroinflammation and psychiatric illness. *J Neuroinflammation*. Apr 1 2013;10:43. doi:10.1186/1742-2094-10-43
4. Garay-Baquero DJ, White CH, Walker NF, et al. Comprehensive plasma proteomic profiling reveals biomarkers for active tuberculosis. *JCI Insight*. Sep 17 2020;5(18)doi:10.1172/jci.insight.137427
5. Vora N, Kalagiri R, Mallett LH, et al. Proteomics and Metabolomics in Pregnancy-An Overview. *Obstet Gynecol Surv*. Feb 2019;74(2):111-125. doi:10.1097/ogx.0000000000000646
6. Kim Y, Kang UB, Kim S, et al. A Validation Study of a Multiple Reaction Monitoring-Based Proteomic Assay to Diagnose Breast Cancer. *J Breast Cancer*. Dec 2019;22(4):579-586. doi:10.4048/jbc.2019.22.e57
7. Dong W, Qiu C, Gong D, et al. Proteomics and bioinformatics approaches for the identification of plasma biomarkers to detect Parkinson's disease. *Exp Ther Med*. Oct 2019;18(4):2833-2842. doi:10.3892/etm.2019.7888
8. Ryan KM, Glaviano A, O'Donovan SM, et al. Electroconvulsive therapy modulates plasma pigment epithelium-derived factor in depression: a proteomics study. *Transl Psychiatry*. Mar 28 2017;7(3):e1073. doi:10.1038/tp.2017.51
9. Noorbakhsh F, Aminian A, Power C. Application of "Omics" Technologies for Diagnosis and Pathogenesis of Neurological Infections. *Curr Neurol Neurosci Rep*. Sep 2015;15(9):58. doi:10.1007/s11910-015-0580-y
10. Jayanthi S, Buie S, Moore S, et al. Heavy marijuana users show increased serum apolipoprotein C-III levels: evidence from proteomic analyses. *Mol Psychiatry*. Jan 2010;15(1):101-12. doi:10.1038/mp.2008.50
11. Organization WH. *Global Recommendations on Physical Activity for Health*. World Health Organization; 2010.
12. Hafkenscheid A. Psychometric evaluation of a standardized and expanded Brief Psychiatric Rating Scale. *Acta psychiatrica Scandinavica*. Sep 1991;84(3):294-300. doi:10.1111/j.1600-0447.1991.tb03147.x
13. Young RC, Biggs JT, Ziegler VE, Meyer DA. A rating scale for mania: reliability, validity and sensitivity. *The British journal of psychiatry : the journal of mental science*. Nov 1978;133:429-35. doi:10.1192/bjp.133.5.429

14. Montgomery SA, Asberg M. A new depression scale designed to be sensitive to change. *The British journal of psychiatry : the journal of mental science*. Apr 1979;134:382-9. doi:10.1192/bjp.134.4.382
15. Hamilton M. The assessment of anxiety states by rating. *The British journal of medical psychology*. 1959;32(1):50-5. doi:10.1111/j.2044-8341.1959.tb00467.x
16. Derogatis LR. SCL-90-R : Administration, scoring & procedures manual-II for the (revised) version and other instruments of the psychopathology rating scale series. *Clinical Psychometric Research*. 1992 1992:1-16.
17. Sussman N, Mullen J, Paulsson B, Vågerö M. Rates of remission/euthymia with quetiapine in combination with lithium/divalproex for the treatment of acute mania. *J Affect Disord*. 2007;100 Suppl 1:S55-63. doi:10.1016/j.jad.2007.02.008
18. Han D, Moon S, Kim Y, Kim J, Jin J, Kim Y. In-depth proteomic analysis of mouse microglia using a combination of FASP and StageTip-based, high pH, reversed-phase fractionation. *Proteomics*. Oct 2013;13(20):2984-8. doi:10.1002/pmic.201300091
19. Kulak NA, Pichler G, Paron I, Nagaraj N, Mann M. Minimal, encapsulated proteomic-sample processing applied to copy-number estimation in eukaryotic cells. *Nat Methods*. Mar 2014;11(3):319-24. doi:10.1038/nmeth.2834
20. Rappsilber J, Mann M, Ishihama Y. Protocol for micro-purification, enrichment, pre-fractionation and storage of peptides for proteomics using StageTips. *Nat Protoc*. 2007;2(8):1896-906. doi:10.1038/nprot.2007.261
21. Han D, Jin J, Woo J, Min H, Kim Y. Proteomic analysis of mouse astrocytes and their secretome by a combination of FASP and StageTip-based, high pH, reversed-phase fractionation. *Proteomics*. Jul 2014;14(13-14):1604-9. doi:10.1002/pmic.201300495
22. Jia P, Han G, Zhao J, Lu P, Zhao Z. SZGR 2.0: a one-stop shop of schizophrenia candidate genes. *Nucleic Acids Res*. Jan 4 2017;45(D1):D915-D924. doi:10.1093/nar/gkw902
23. Wu Y, Li X, Liu J, Luo XJ, Yao YG. SZDB2.0: an updated comprehensive resource for schizophrenia research. *Hum Genet*. Oct 2020;139(10):1285-1297. doi:10.1007/s00439-020-02171-1
24. Kim S, Webster MJ. The Stanley Neuropathology Consortium Integrative Database (SNCID) for Psychiatric Disorders. *Neurosci Bull*. Apr 2019;35(2):277-282. doi:10.1007/s12264-018-0314-7
25. Gutierrez-Sacristan A, Grosdidier S, Valverde O, et al. PsyGeNET: a knowledge platform on psychiatric disorders and their genes. *Bioinformatics*. Sep 15 2015;31(18):3075-7. doi:10.1093/bioinformatics/btv301
26. Nanjappa V, Thomas JK, Marimuthu A, et al. Plasma Proteome Database as a resource for proteomics research: 2014 update. *Nucleic Acids Res*. Jan 2014;42(Database issue):D959-65. doi:10.1093/nar/gkt1251
27. Ponten F, Schwenk JM, Asplund A, Edqvist PH. The Human Protein Atlas as a proteomic resource for biomarker discovery. *J Intern Med*. Nov 2011;270(5):428-46. doi:10.1111/j.1365-2796.2011.02427.x
28. Abbatiello SE, Mani DR, Keshishian H, Carr SA. Automated detection of inaccurate and

imprecise transitions in peptide quantification by multiple reaction monitoring mass spectrometry. *Clin Chem*. Feb 2010;56(2):291-305. doi:10.1373/clinchem.2009.138420

29. Cuklina J. LCH, Williams E.G., Collins B., Sajic T., Pedrioli P., Rodriguez-Martinez M., Aebersold, R. Computational challenges in biomarker discovery from high-throughput proteomic data. 2018;doi:https://doi.org/10.3929/ethz-b-000307772

30. Friedman J, Hastie T, Tibshirani R. Regularization Paths for Generalized Linear Models via Coordinate Descent. *J Stat Softw*. 2010;33(1):1-22.

31. Burnham KP, Anderson DR. Multimodel inference: understanding AIC and BIC in model selection. *Sociological methods & research*. 2004;33(2):261-304.

32. David Meyer ED, Kurt Hornik, Andreas Weingessel, and Friedrich Leisch. e1071: Misc Functions of the Department of Statistics. 2021;

33. Džeroski S, Ženko B. Is combining classifiers with stacking better than selecting the best one? *Machine learning*. 2004;54(3):255-273.

34. Robin X, Turck N, Hainard A, et al. pROC: an open-source package for R and S+ to analyze and compare ROC curves. *BMC Bioinformatics*. Mar 17 2011;12:77. doi:10.1186/1471-2105-12-77

35. Hanley JA, McNeil BJ. The meaning and use of the area under a receiver operating characteristic (ROC) curve. *Radiology*. Apr 1982;143(1):29-36. doi:10.1148/radiology.143.1.7063747

36. Youden WJ. Index for rating diagnostic tests. *Cancer*. Jan 1950;3(1):32-5. doi:10.1002/1097-0142(1950)3:1<32::aid-cncr2820030106>3.0.co;2-3

37. Hoffman GE, Schadt EE. variancePartition: interpreting drivers of variation in complex gene expression studies. *BMC bioinformatics*. 2016;17(1):1-13.

38. Alnafisah RS, Reigle J, Eladawi MA, et al. Assessing the effects of antipsychotic medications on schizophrenia functional analysis: a postmortem proteome study. *Neuropsychopharmacology*. 2022:1-9.

39. Santa Cruz EC, Zandonadi FDS, Fontes W, Sussulini A. A pilot study indicating the dysregulation of the complement and coagulation cascades in treated schizophrenia and bipolar disorder patients. *Biochim Biophys Acta Proteins Proteom*. Aug 2021;1869(8):140657. doi:10.1016/j.bbapap.2021.140657

40. Smirnova L, Seregin A, Boksha I, et al. The difference in serum proteomes in schizophrenia and bipolar disorder. *BMC Genomics*. Jul 11 2019;20(Suppl 7):535. doi:10.1186/s12864-019-5848-1

41. Mast AE. Tissue Factor Pathway Inhibitor: Multiple Anticoagulant Activities for a Single Protein. *Arterioscler Thromb Vasc Biol*. Jan 2016;36(1):9-14. doi:10.1161/ATVBAHA.115.305996

42. Fei X, Wang H, Yuan W, Wo M, Jiang L. Tissue Factor Pathway Inhibitor-1 Is a Valuable Marker for the Prediction of Deep Venous Thrombosis and Tumor Metastasis in Patients with Lung Cancer. *Biomed Res Int*. 2017;2017:8983763. doi:10.1155/2017/8983763

43. Zhuo L, Hascall VC, Kimata K. Inter-alpha-trypsin inhibitor, a covalent protein-glycosaminoglycan-protein complex. *J Biol Chem*. Sep 10 2004;279(37):38079-82. doi:10.1074/jbc.R300039200

44. Wang Q, Su X, Jiang X, et al. iTRAQ technology-based identification of human peripheral

serum proteins associated with depression. *Neuroscience*. Aug 25 2016;330:291-325. doi:10.1016/j.neuroscience.2016.05.055
45. Focking M, Sabherwal S, Cates HM, et al. Complement pathway changes at age 12 are associated with psychotic experiences at age 18 in a longitudinal population-based study: evidence for a role of stress. *Mol Psychiatry*. Feb 2021;26(2):524-533. doi:10.1038/s41380-018-0306-z

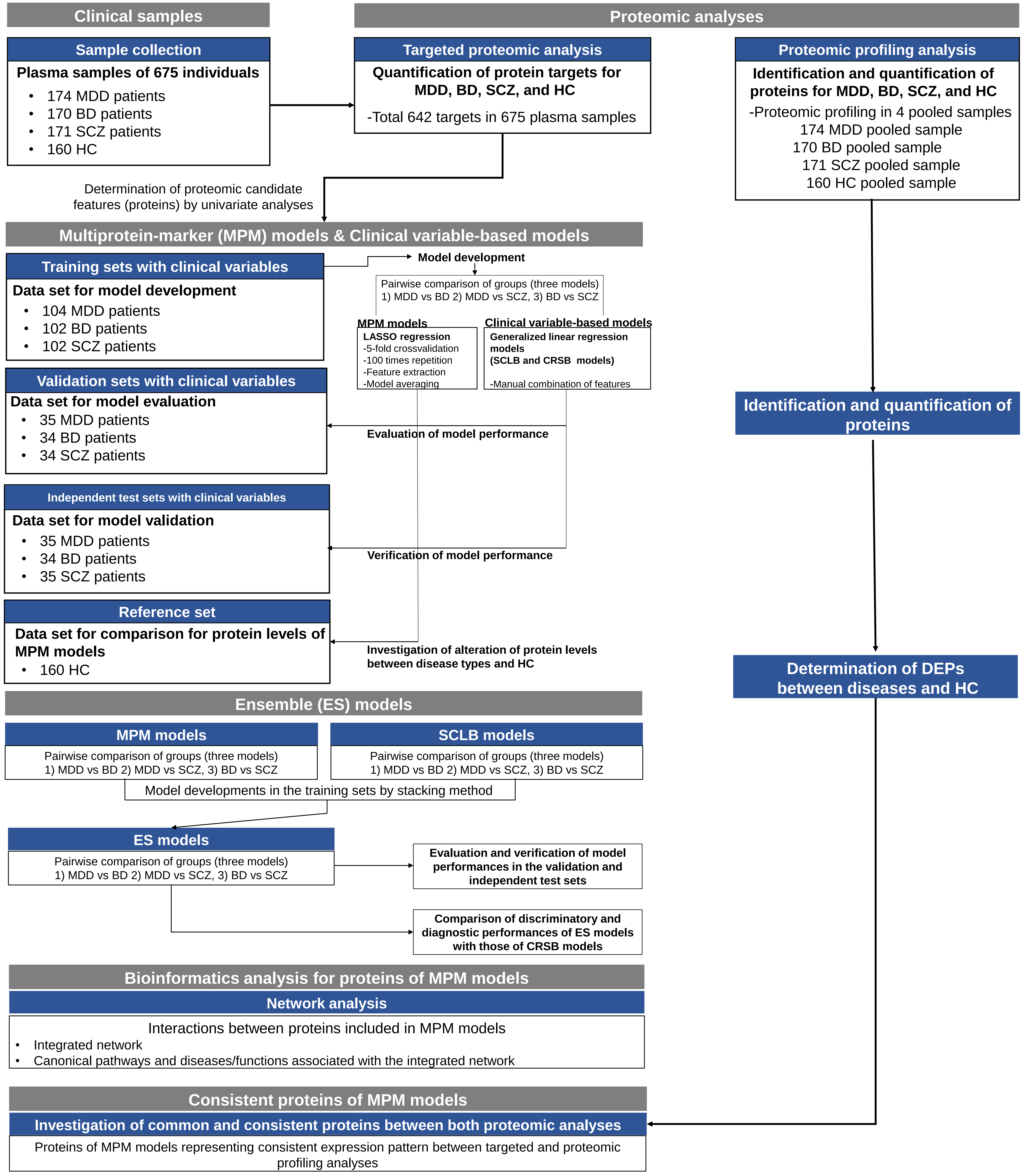

Figure S1. Overall scheme of the study. The study consists of "Clinical samples," "Proteomic analyses," "Multiprotein-marker (MPM) models & Clinical variable-based models," "Ensemble (ES) models," "Bioinformatics analysis of proteins in MPM models," and "Consistent proteins in MPM models." MDD, major depressive disorder; BD, bipolar disorder; SCZ, schizophrenia; HC, healthy control; MPM, multiprotein marker; SCLB, symptom checklist-based; CRSB, clinician rater score-based; ES ensemble; DEP, differentially expressed protein.

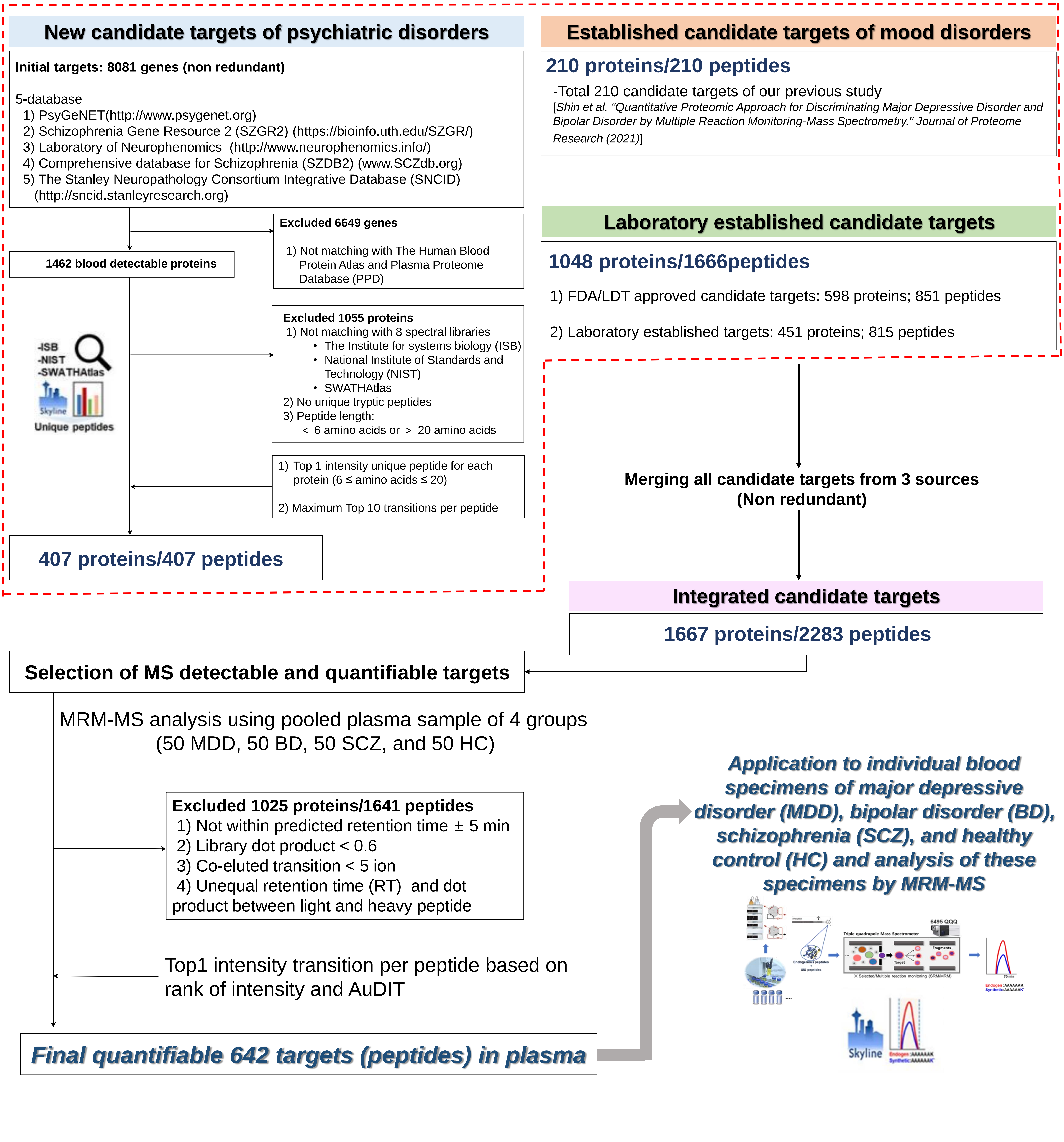

Figure S2. Overall workflow for determining quantifiable targets. Overall process, including integrated protein candidate targets, selection of MS-detectable targets, and determination of final quantifiable targets. The number of selected targets for each step is presented. The final quantifiable targets were applied to MRM-MS analysis of individual blood samples of MDD, BD, SCZ patients and HCs. MS, mass spectrometry; MRM, multiple reaction monitoring; MDD, major depressive disorder; BD, bipolar disorder; SCZ, schizophrenia; HC, healthy control; FDA, US Food and Drug Administration; LDT, laboratory-developed test; AuDIT, automated detection of inaccurate and imprecise transition.

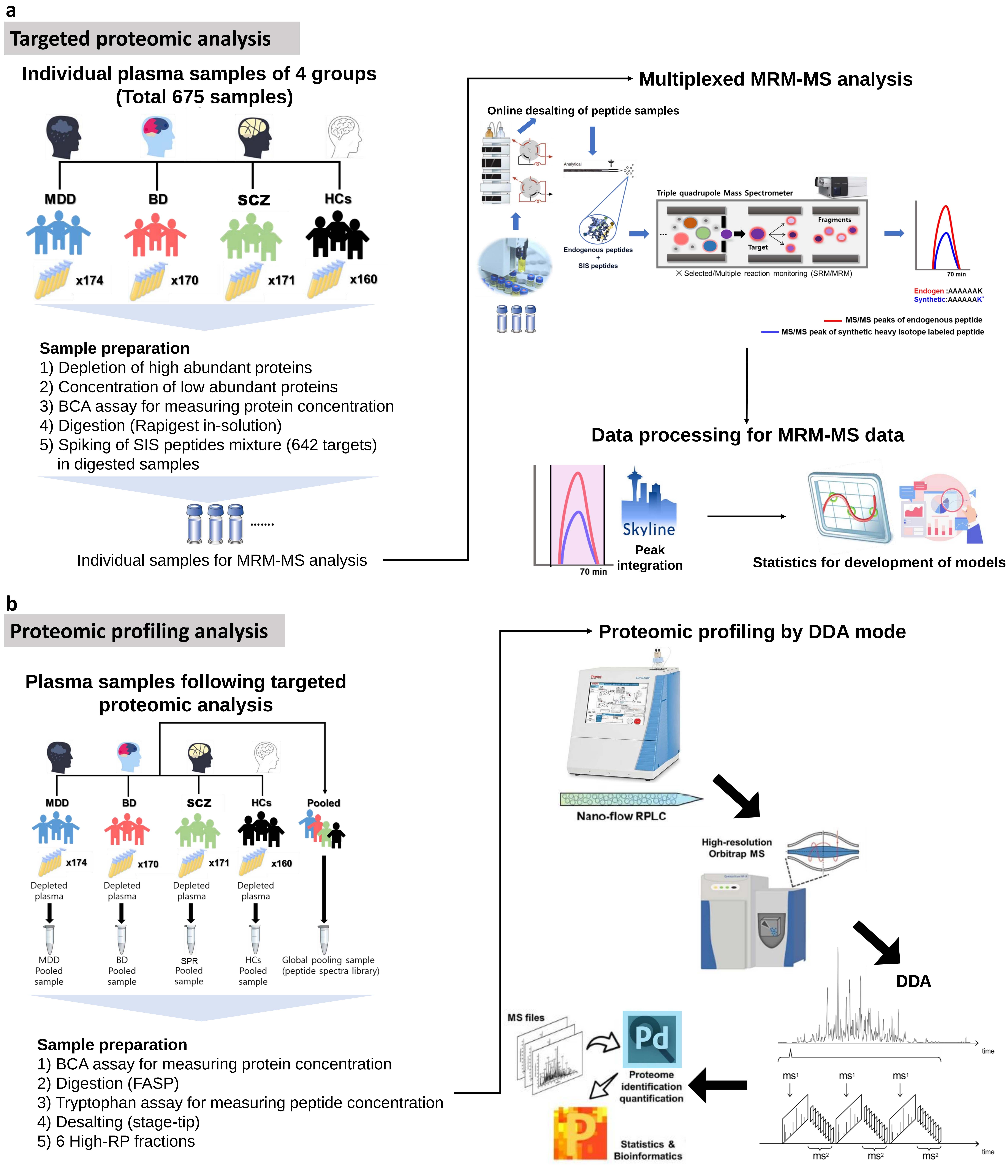

Figure S3. Workflows of experiments and MS-based analysis for targeted proteomic and proteomic profiling analysis. Overall workflow for (a) targeted proteomic analysis and (b) proteomic profiling analysis. SIS, stable-isotope synthetic; MRM, multiple reaction monitoring; MS, mass spectrometry; FASP, filter-aided sample preparation; RP, reverse-phase; DDA; data-dependent acquisition; MDD, major depressive disorder; BD, bipolar disorder; SCZ, schizophrenia; HC, healthy control.

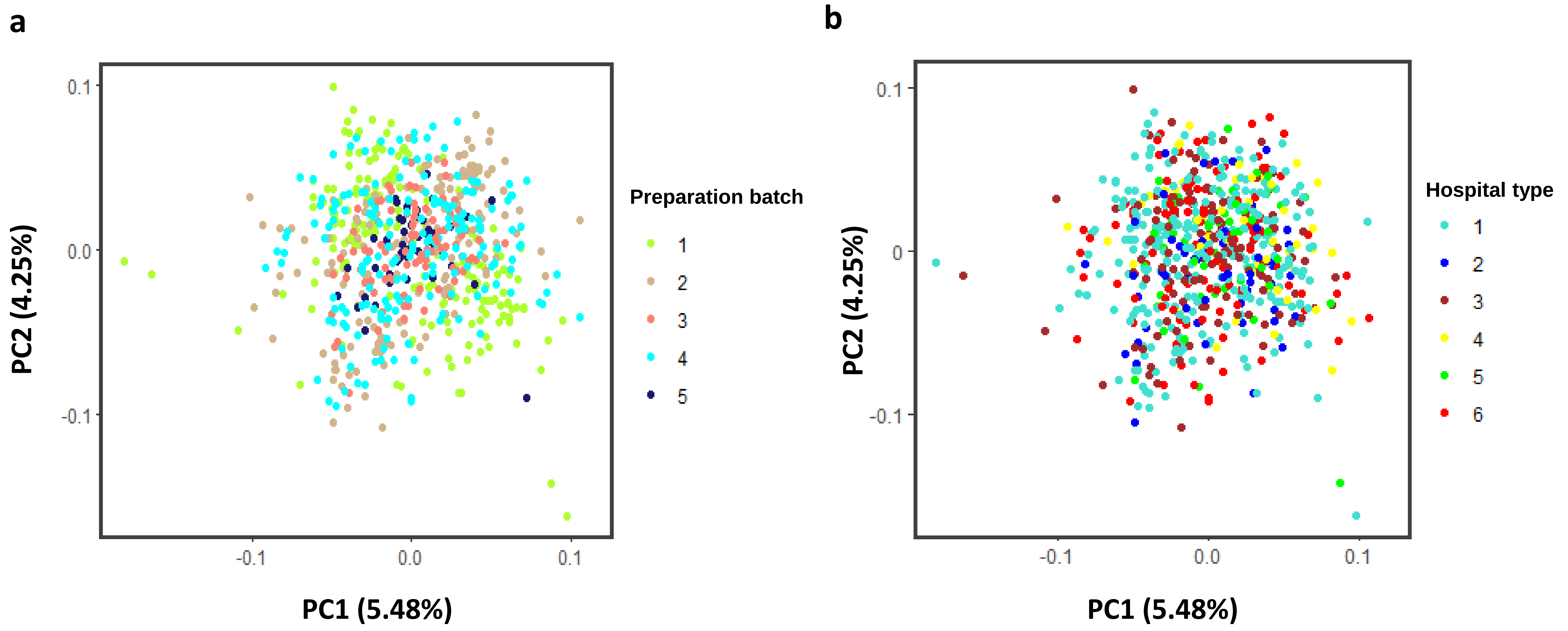

Figure S4. Principal component analysis of clinical samples (171 SCZ, 170 BD, 174 MDD, and 160 HCs) based on adjusted peak area ratio of MRM-MS targets after batch effect correction. The first 2 principal components are plotted with the percentage of variation accounted for by each principal component in the axis labels. (a) PCA plot of clinical samples for sample preparation batches, analyzed at various time points—1: batch 1, 2: batch 2, 3: batch 3, 4: batch 4, and 5: batch 5. (b) PCA plot of clinical samples for hospital type—1: Seoul National University Hospital 2: Inha University Hospital, 3: Nowon Eulji Medical Center, Eulji University, 4: Cha University Bundang Medical Center, 5: Hanyang University Seoul Hospital, and 6: Seoul Metropolitan Government Seoul National University Boramae Medical Center. PCA, principal component analysis; MDD, major depressive disorder; BD, bipolar disorder; SCZ, schizophrenia; HC, healthy control; MRM-MS, multiple reaction monitoring-mass spectrometry.

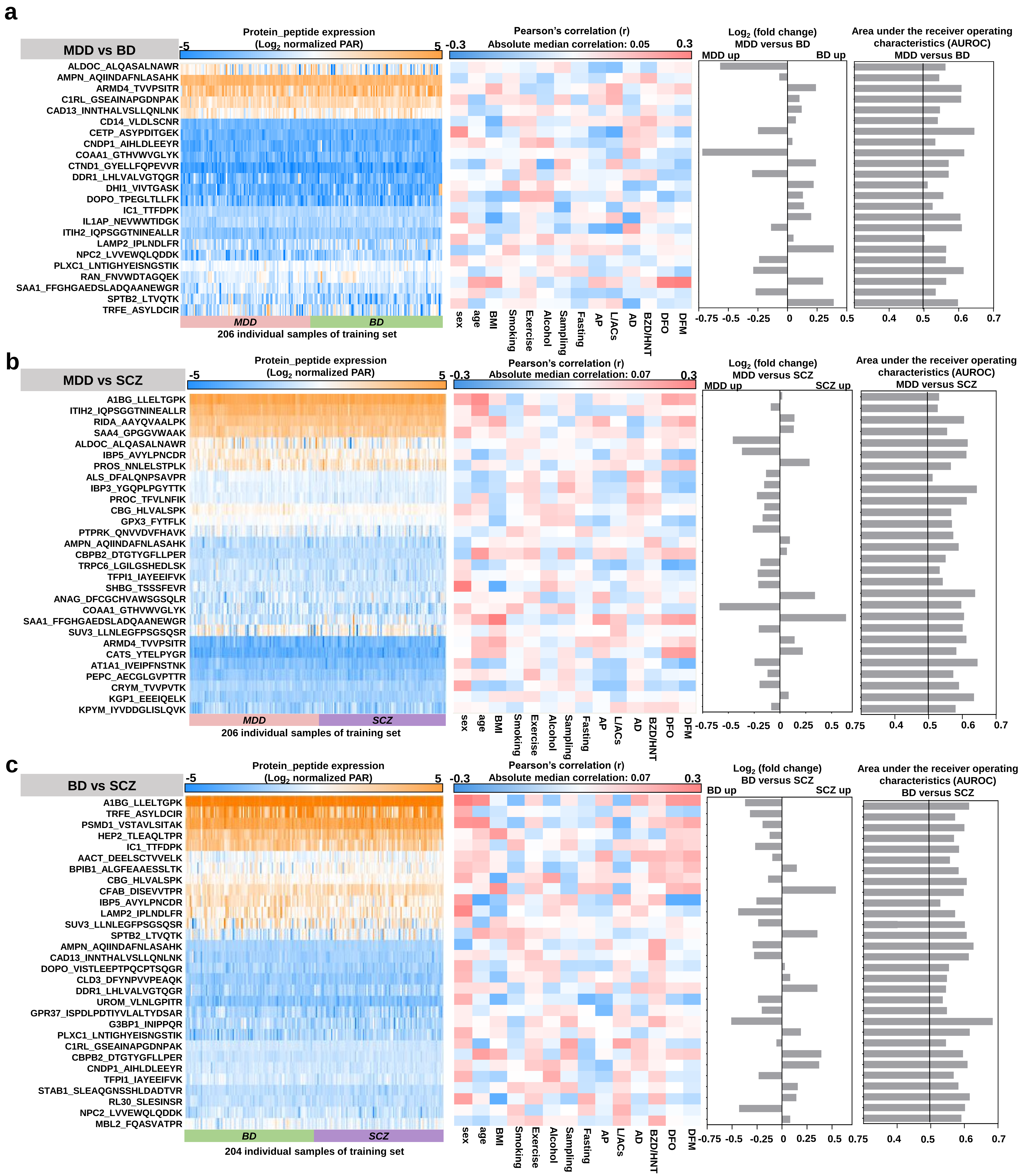

Figure S5. Univariate analysis to determine proteomic candidate features (proteins). Heatmaps of expression of proteomic candidate features (proteins) for each pairwise comparison of groups are presented as log_2-normalized PAR. Correlation plots for the features and demographic variables are presented by Pearson's correlation (r). Alterations in features are presented as log_2-fold-change. AUROC was used to determine the ability of features to discriminate disease types. Univariate analysis results for (a) MDD versus BD, (b) MDD versus SCZ, and (c) BD versus SCZ. MDD, major depressive disorder; BD, bipolar disorder; SCZ, schizophrenia; PAR, peak area ratio; AUROC, area under the receiver operating characteristics; AP, antipsychotics; L/ACs, lithium/anticonvulsants; AD, antidepressant; BZDs/HNTs, benzodiazepines/hypnotics; DFO, duration from first onset; DFM, duration from first medication.

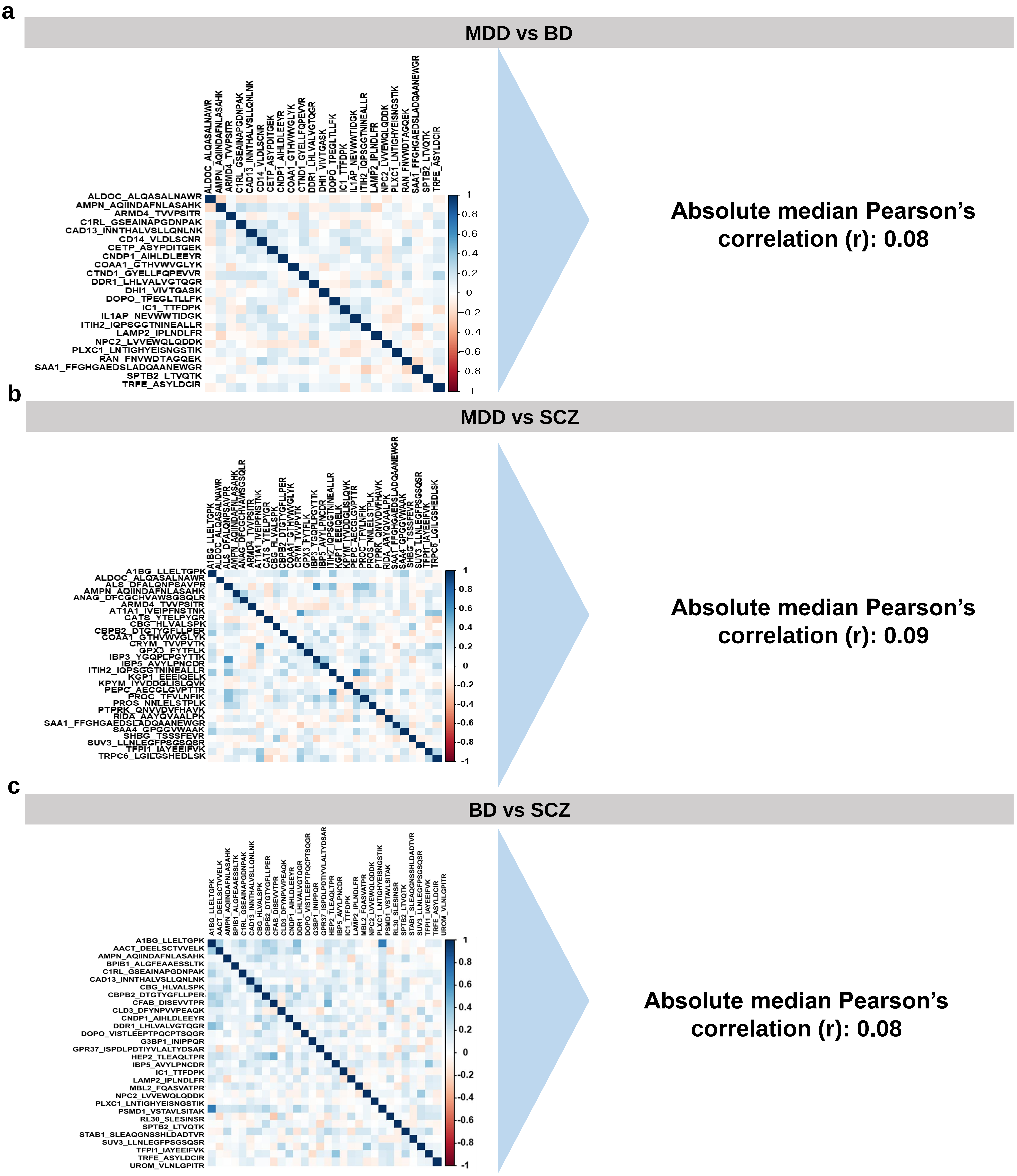

Figure S6. Intercorrelation between proteomic candidate features (proteins) by univariate analysis. Correlation matrix of expression of proteomic candidate features (proteins) determined by univariate analysis for each pairwise comparison of groups. Correlation values were calculated using Pearson's correlation coefficient (r). Absolute median values of each correlation matrix are presented. Results for (a) MDD vs BD, (b) MDD vs SCZ, and (c) BD vs SCZ. MDD, major depressive disorder; BD, bipolar disorder; SCZ, schizophrenia.

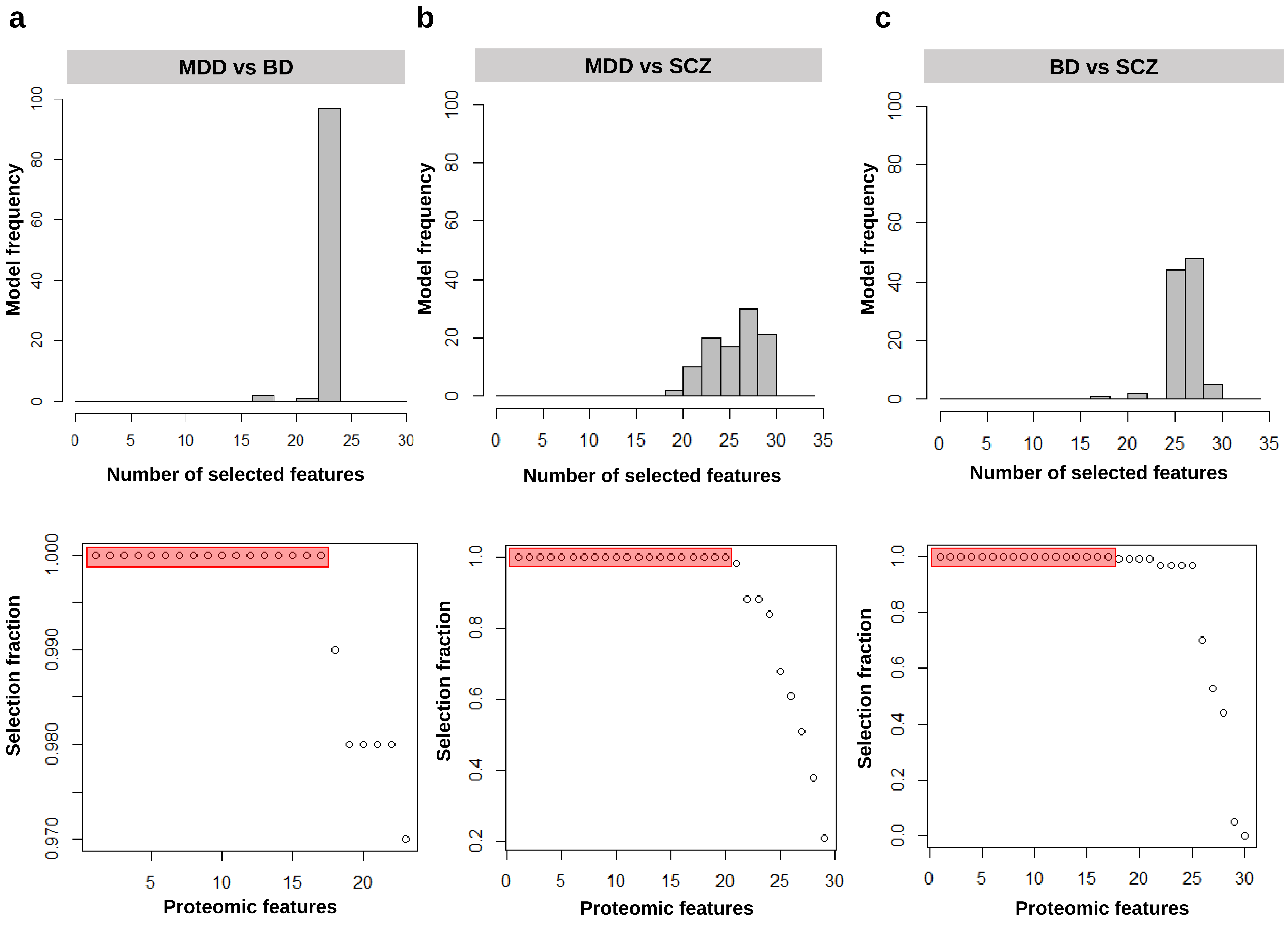

Figure S7. Feature extraction across 100 models generated by repeated LASSO regression with 5-fold crossvalidation on the training sets of each pairwise comparison of groups. Frequency of selected features for the 100 generated models for each pairwise comparison of groups (the top panel). Selection fraction for proteomic features (proteins) used to develop MPM models (bottom panel). Proteomic features (proteins) satisfying selection fraction=1 are included in red boxes. Results for (a) MDD vs BD, (b) MDD vs SCZ, and (c) BD vs SCZ. ; LASSO, the least absolute shrinkage and selection operator, MPM, multiprotein marker; MDD, major depressive disorder; BD, bipolar disorder; SCZ, schizophrenia.

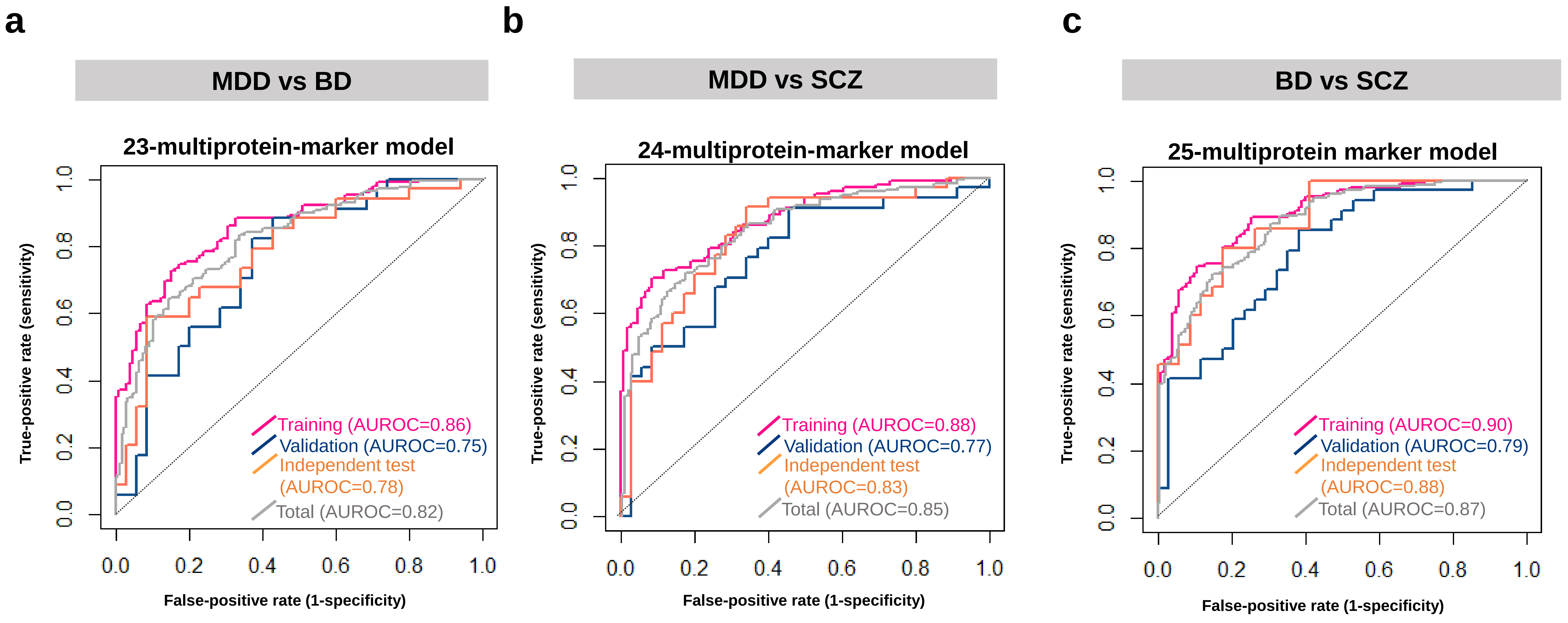

Figure S8. Discriminatory performance of MPM models combining additional proteomic features (proteins). To correlate the increase in performance with that of the number of combined proteomic features (proteins), the performance of MPM models with additional proteomic features (proteins) (satisfying selection fraction ≥ 0.8) was examined. Discriminatory performance is presented as AUROC values in the training, validation, independent test, and total sets. Results of altered discriminatory performance for (a) MDD vs BD, (b) MDD vs SCZ, and (c) BD vs SCZ. MPM, multiprotein marker; MDD, major depressive disorder; BD, bipolar disorder; SCZ, schizophrenia; AUROC, area under the receiver operating characteristics.

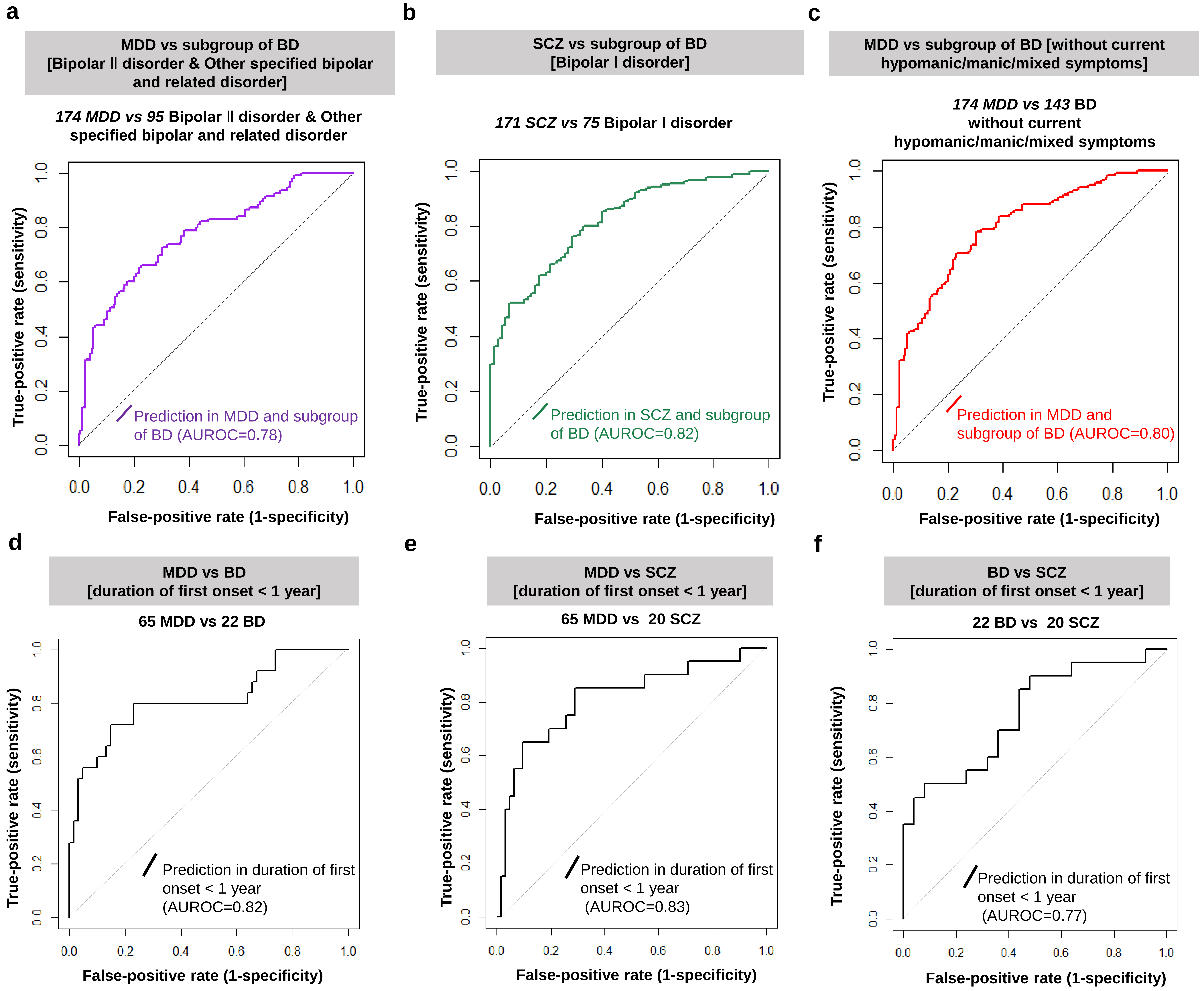

Figure S9. Prediction of discriminatory performance of MPM models in different subgroups. MPM models for MDD vs BD and BD vs SCZ were applied to predict discriminatory performance in subgroups of BD: (a) 95 Bipolar II disorder & Other specified bipolar and related disorder, (b) 75 Bipolar I disorder, and (c) 143 without current hypomanic/manic/mixed symptoms. In addition, MPM models for MDD vs BD, MDD vs SCZ, and BD vs SCZ were applied to predict discriminatory performance in subgroups of duration of first onset <1 year. Discriminatory performance is presented as AUROC values. MPM, multiprotein marker; MDD, major depressive disorder; BD, bipolar disorder; SCZ, schizophrenia; AUROC, area under the receiver operating characteristics.

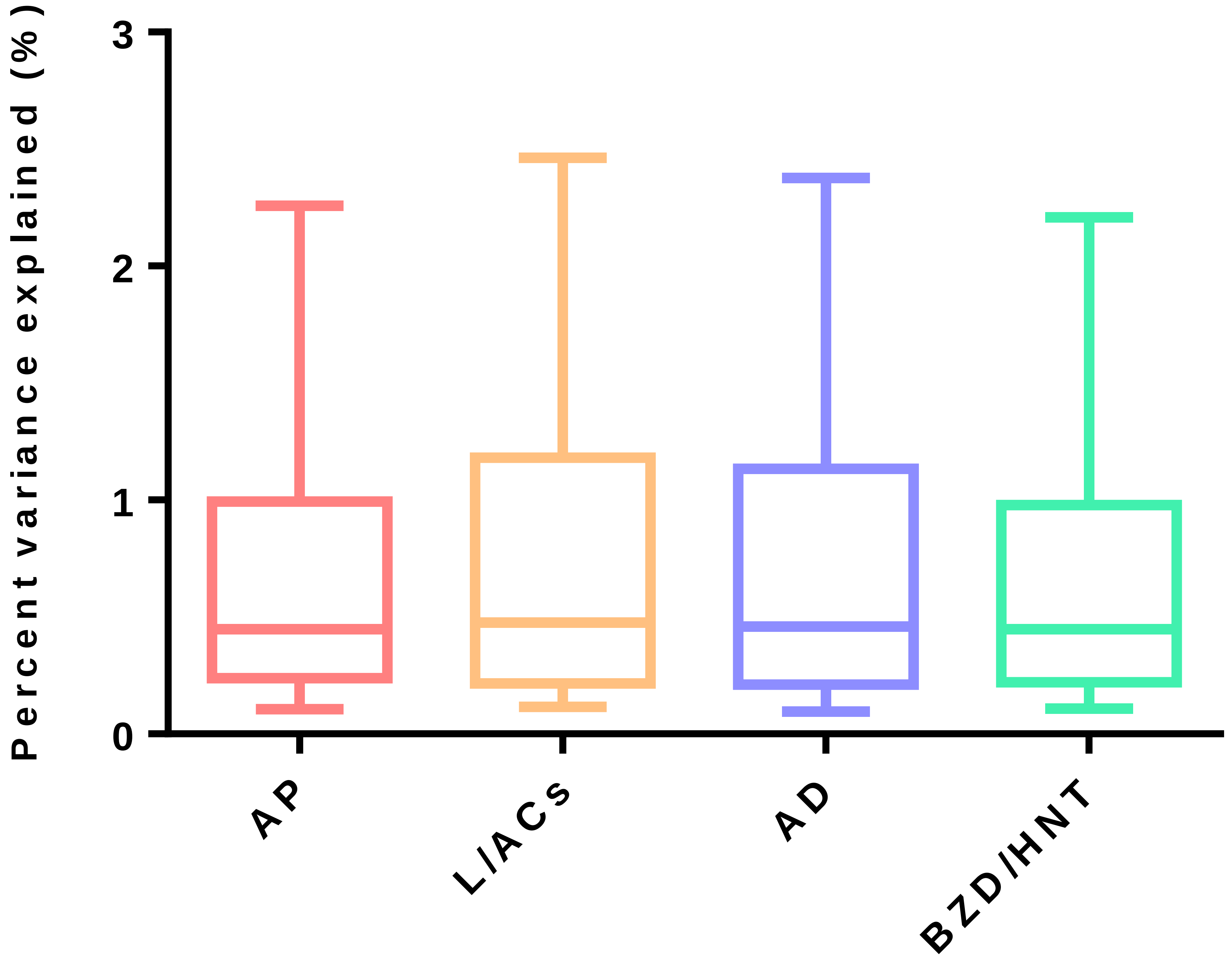

Figure S10. Effect of medications on the proteins of MPM models. Box plots of percent variance for examination of medication effects on the proteins of MPM models across all patients. AP, antipsychotics; L/ACs, lithium/anticonvulsants; AD, antidepressant; BZD/HNT, benzodiazepines/hypnotics.

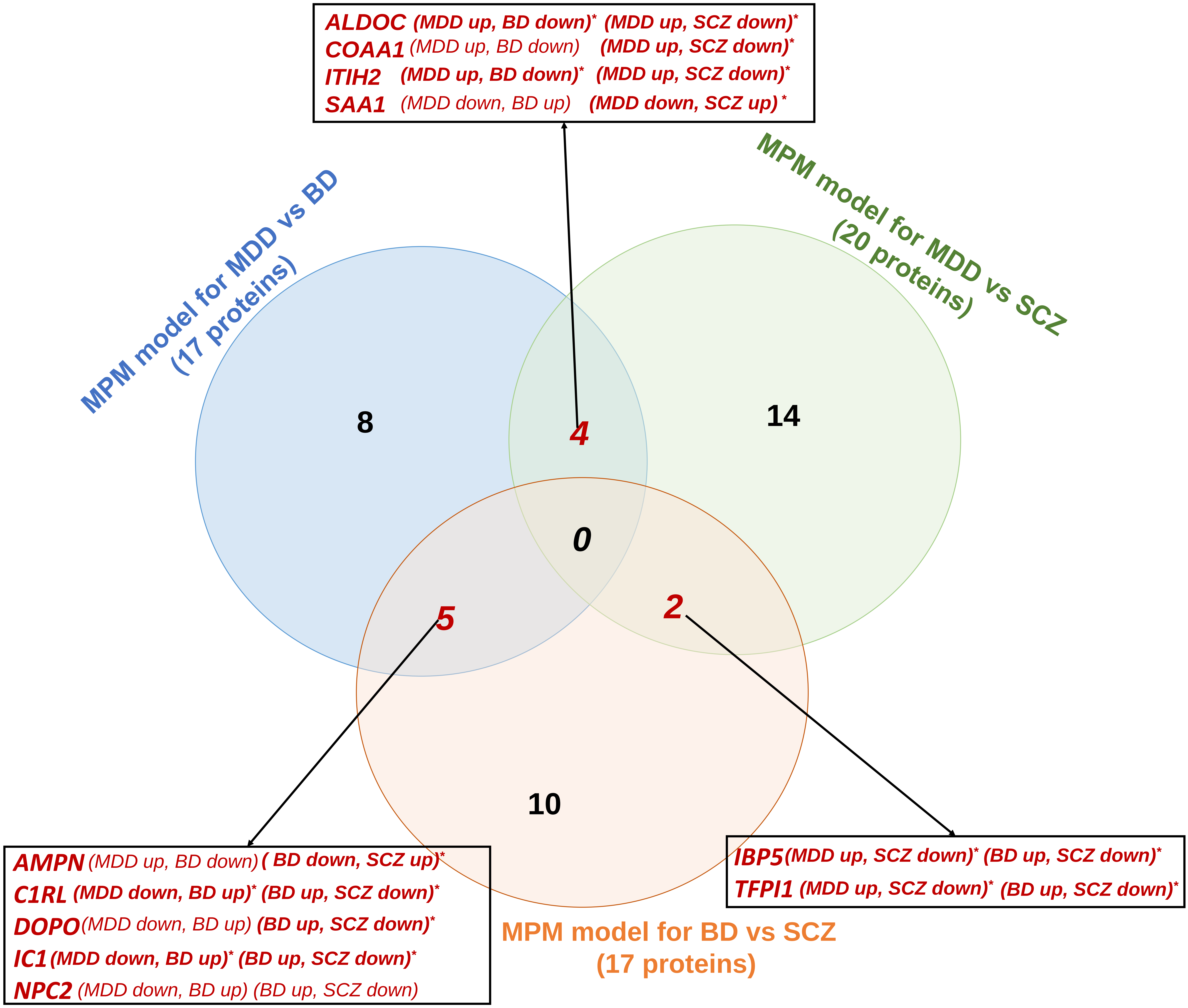

Figure S11. Distribution of proteins in MPM models. Venn diagram of proteins in MPM models. Overlapping proteins (represented as protein entry) between MPM models are shown in wine-colored font, and their expression pattern is indicated—up: upregulated and down: downregulated. Proteins with significant differences between disease types are signified by bold font with asterisk. MPM, multiprotein marker; MDD, major depressive disorder; BD, bipolar disorder; SCZ, schizophrenia.

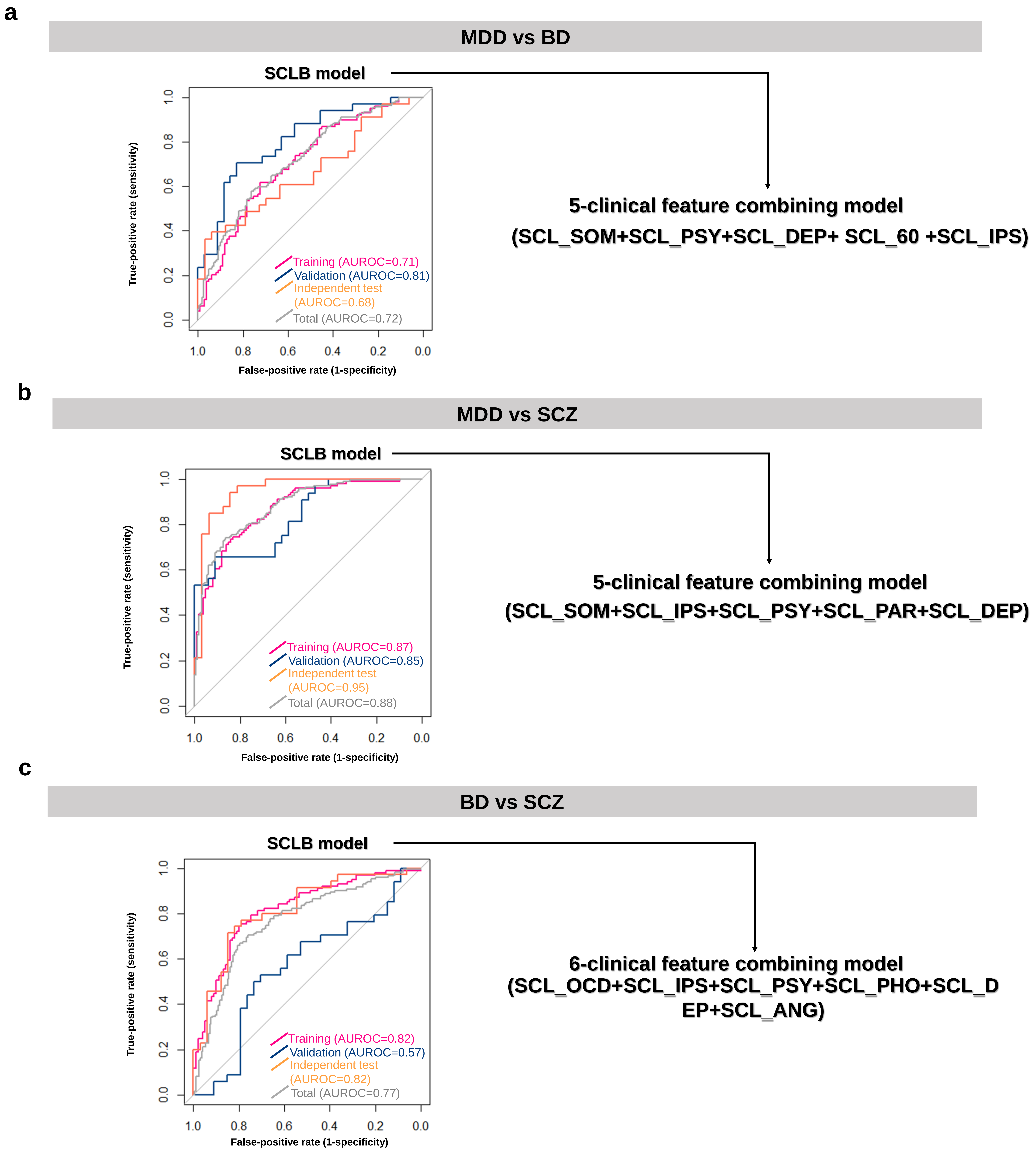

Figure S12. Discriminatory performance of symptom checklist-based models for each pairwise comparison of groups. Discriminatory performance of SCLB models presented as AUROC values in the training, validation, independent test, and total sets. Results for (a) MDD vs BD, (b) MDD vs SCZ, and (c) BD vs SCZ. MDD, major depressive disorder; BD, bipolar disorder; SCZ, schizophrenia; SCL, Symptom Checklist-90-Revised; SCLB, symptom checklist-based; OCD, obsessive-compulsive dimension; SOM, somatization dimension; IPS, interpersonal sensitivity dimension; ANG, hostility dimension; PHO, phobic anxiety dimension; PAR, paranoid ideation dimension; PSY, psychoticism dimension; SCL_60, overeating item; AUROC, area under the receiver operating characteristics.

a

MDD vs BD

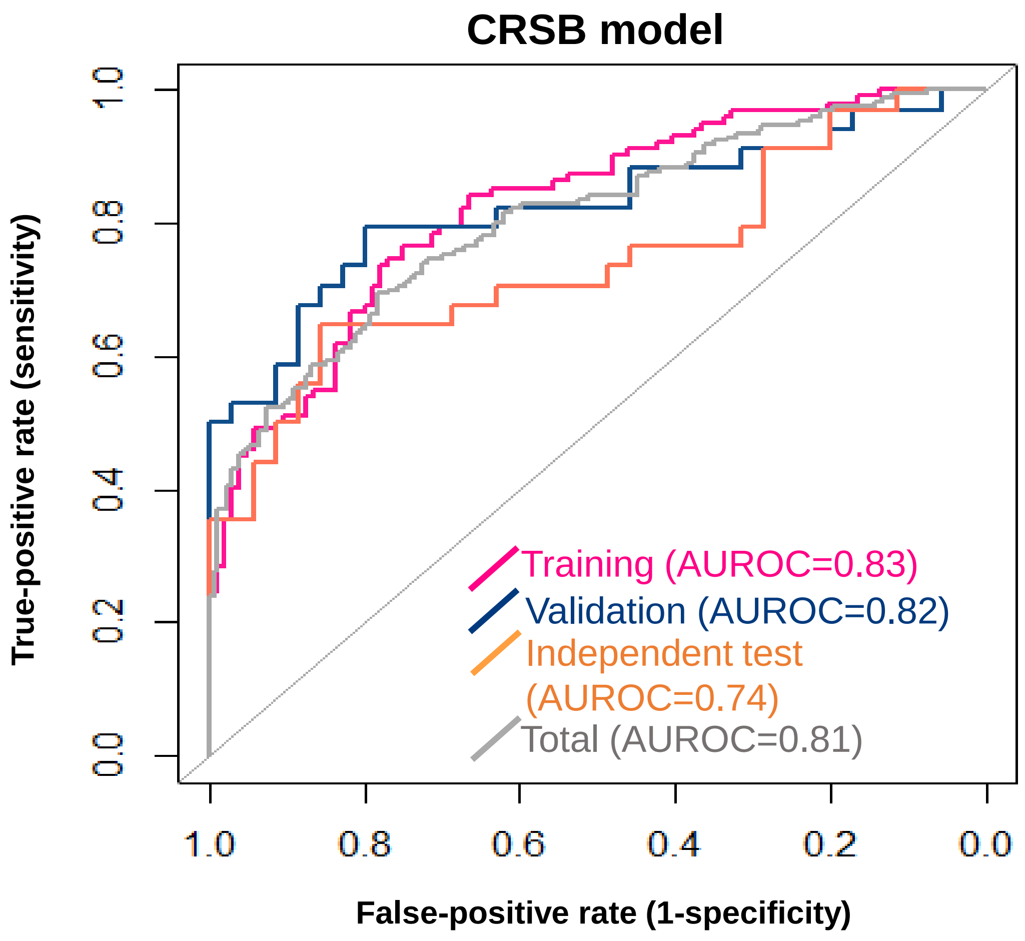

Prediction of diagnostic performances

Performance results (Independent test set)

	Value
AUROC	*0.74*
Optimal cutoff	*0.64*
Accuracy	*0.75*
Sensitivity	*0.65*
Specificity	*0.86*
PPV	*0.82*
NPV	*0.71*

b

MDD vs SCZ

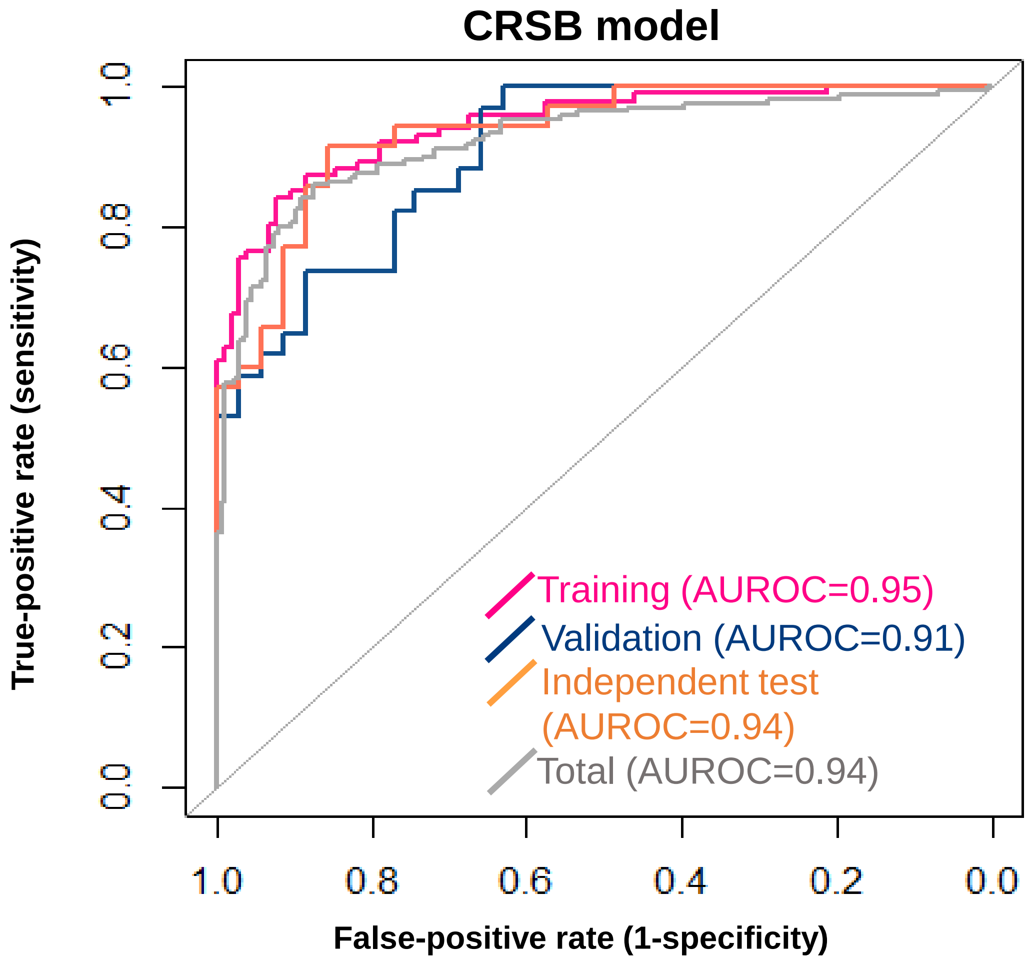

Prediction of diagnostic performances

Performance results (Independent test set)

	Value
AUROC	*0.94*
Optimal cutoff	*0.42*
Accuracy	*0.89*
Sensitivity	*0.91*
Specificity	*0.86*
PPV	*0.87*
NPV	*0.91*

c

BD vs SCZ

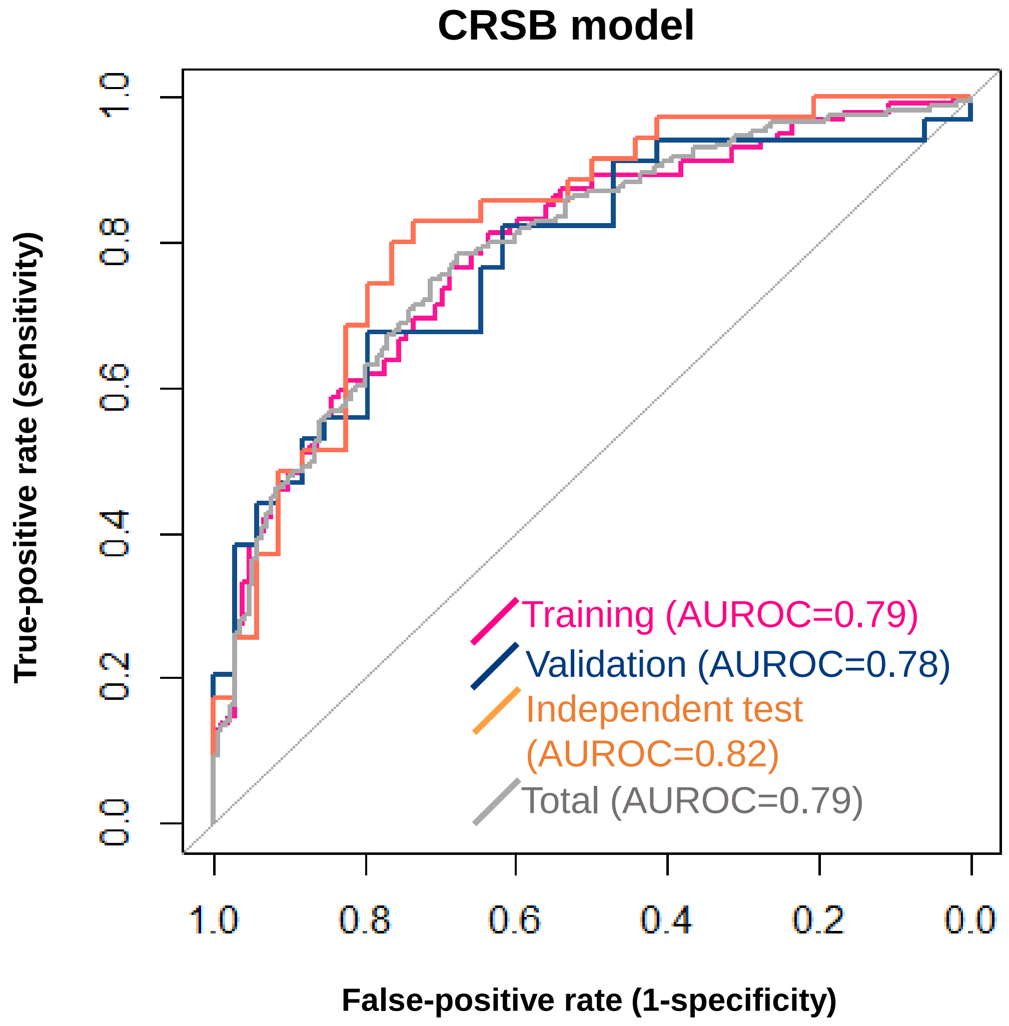

Prediction of diagnostic performances

Performance results (Independent test set)

	Value
AUROC	*0.82*
Optimal cutoff	*0.47*
Accuracy	*0.78*
Sensitivity	*0.80*
Specificity	*0.76*
PPV	*0.78*
NPV	*0.79*

Figure S13. Diagnostic performance of clinician rater score-based models for each comparison of groups. Discriminatory performance presented as AUROC values in the training, validation, independent test, and total sets. Diagnostic performance with the independent test sets presented as accuracy, sensitivity, specificity, PPV, and NPV. Results for (a) MDD vs BD, (b) MDD vs SCZ, and (c) BD vs SCZ. MDD, major depressive disorder; BD, bipolar disorder; SCZ, schizophrenia; AUROC, area under the receiver operating characteristics; PPV, positive predictive value; NPV, negative predictive value; CRSB, clinician rater score-based.

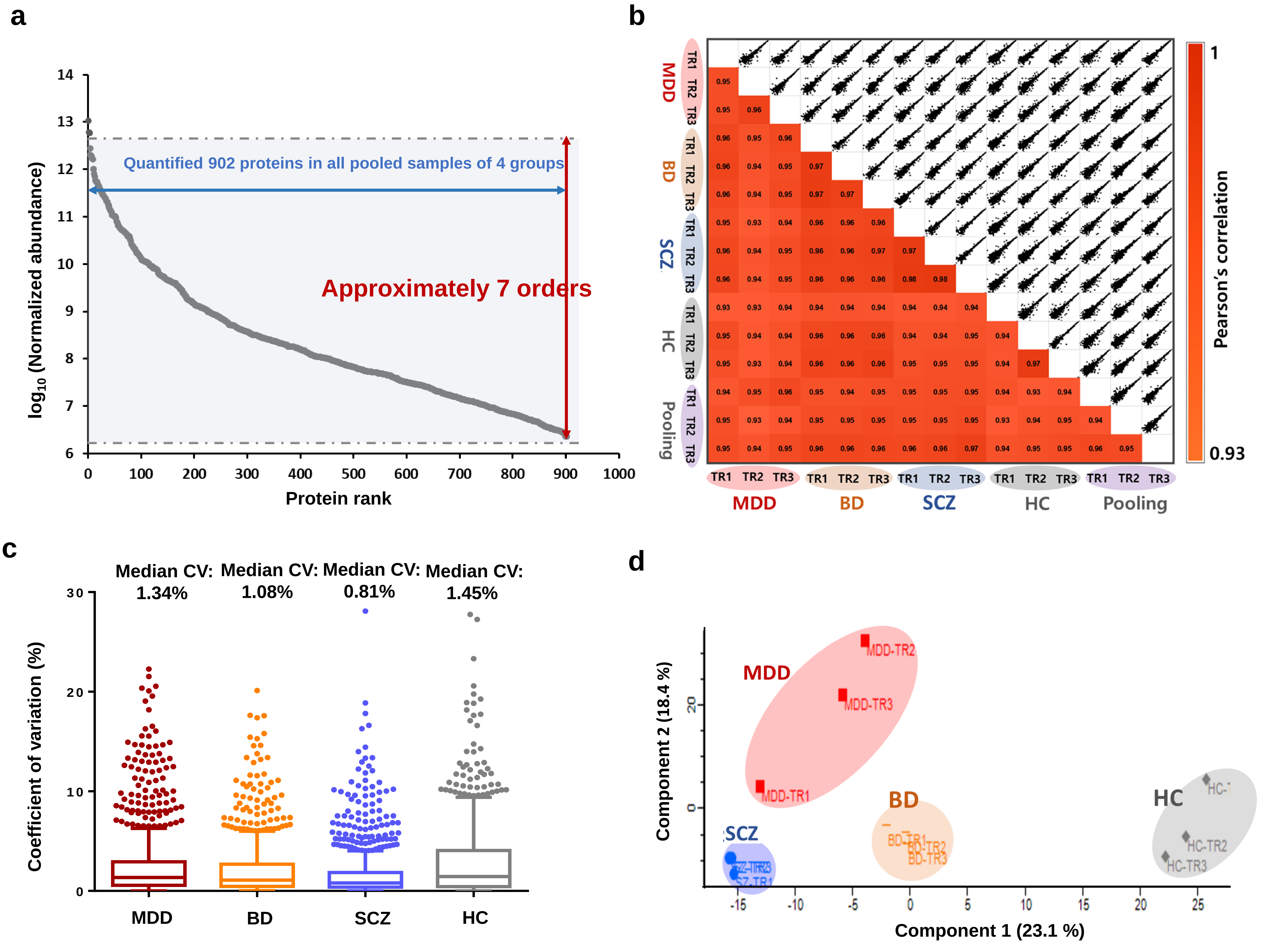

Figure S14. Proteome coverage and assessment of proteomic profiling data of four pooled plasma samples for each group (SCZ, BD, MDD, and HC). (a) Dynamic range of 902 quantified proteins across pooled plasma samples of 4 groups. (b) Technical and biological variances in MS analysis are shown as a multiscatter plot. (c) Coefficient of variation (CV) values between technical replicates of MS analysis. (d) Principal component analysis (PCA) of distinct clusters of pooled plasma proteome for each group. MS, mass spectrometry; MDD, major depressive disorder; BD, bipolar disorder; SCZ, schizophrenia; HC, healthy control; TR, technical replicate.

Table S1. Demographics and clinical characteristics of the subjects†

Characteristics	SCZ n=171	BD n=170	MDD n=174	HC n=160	SCZ vs BD vs MDD vs HC		Post hoc analysis‡	
Sex: Male	74 (43.3%)	58 (34.1%)	61 (35.1%)	48 (30.0%)	χ2 = 6.755	p = 0.08		
Age	39.21 ± 11.80	34.31 ± 12.27	36.31 ± 13.23	35.94 ± 11.20	**F = 4.784**	**p = 0.003**	SCZ > BD	
BMI	25.84 ± 4.84	24.57 ± 4.41	23.46 ± 4.04	22.08 ± 2.72	**F = 25.412**	**p < 0.001**	SCZ > BD = MDD > HC	
Blood collection time: AM	81 (47.4%)	50 (29.4%)	55 (31.6%)	69 (43.1%)	**χ2 = 16.516**	**p = 0.001**		
Fasting time: at least 8 hours	39 (22.8%)	33 (19.4%)	42 (24.1%)	87 (54.4%)	**χ2 = 61.650**	**p < 0.001**		
Exercise: moderate	60 (35.1%)	68 (40.0%)	52 (29.9%)	116 (72.5%)	**χ2 = 73.476**	**p < 0.001**		
Alcohol drinking: at least once a week	32 (18.7%)	60 (35.3%)	63 (36.2%)	75 (46.9%)	**χ2 = 30.098**	**p < 0.001**		
Smoking: current smoker	41 (24.0%)	53 (31.2%)	59 (33.9%)	8 (5.0%)	**χ2 = 46.018**	**p < 0.001**		
Duration from first onset	12.48 ± 10.02	9.39 ± 8.77	6.73 ± 7.75*		**F = 17.985**	**p < 0.001**	SCZ > BD > MDD	
Duration from first medication	11.72 ± 9.89	6.91 ± 8.34	3.68 ± 6.03		**F = 41.613**	**p < 0.001**	SCZ > BD > MDD	
Medication								
Antipsychotics	166 (97.1%)	130 (76.5%)	72 (41.4%)		**χ2 = 134.298**	**p < 0.001**		
Lithium/Anticonvulsants	28 (16.4%)	125 (73.5%)	24 (13.8%)		**χ2 = 172.782**	**p < 0.001**		
Antidepressant	37 (21.6%)	44 (26.0%)	145 (83.3%)*		**χ2 = 166.118**	**p < 0.001**		
Benzodiazepines/hypnotics	104 (60.8%)	106 (62.4%)	117 (67.2%)		χ2 = 1.678	p = 0.43		
Clinician rater score								
BPRS	43.64 ± 11.98	39.47 ± 8.15	40.61 ± 6.98	27.06 ± 3.75	**F = 126.282**	**p < 0.001**	SCZ > BD = MDD > HC	
YMRS	4.49 ± 5.81	5.68 ± 6.95	1.87 ± 2.55	1.19 ± 2.11	**F = 32.242**	**p < 0.001**	SCZ = BD > MDD = HC	
MADRS	13.80 ± 9.54	17.48 ± 10.49	26.05 ± 9.89	4.14 ± 4.24	**F = 171.012**	**p < 0.001**	MDD > BD > SCZ > HC	
HAM-A	8.75 ± 6.67	9.88 ± 5.98	14.94 ± 7.21	2.27 ± 2.01	**F = 130.165**	**p < 0.001**	MDD > SCZ = BD > HC	
Self-reported scale								
Symptom Checklist-90-Revised								
Somatization dimension	0.77 ± 0.75*	0.86 ± 0.74***	1.39 ± 0.94**	0.17 ± 0.21	**F = 81.329**	**p < 0.001**	MDD > SCZ = BD > HC	
Obsessive-compulsive dimension	1.26 ± 0.90*	1.48 ± 0.87***	1.90 ± 0.84**	0.38 ± 0.36	**F = 111.170**	**p < 0.001**	MDD > BD > SCZ > HC	
Interpersonal sensitivity dimension	1.22 ± 0.90*	1.29 ± 0.85***	1.63 ± 0.91**	0.30 ± 0.32	**F = 85.622**	**p < 0.001**	MDD > SCZ = BD > HC	
Depression dimension	1.22 ± 0.96*	1.60 ± 0.94***	2.22 ± 0.92**	0.29 ± 0.35	**F = 154.116**	**p < 0.001**	MDD > BD > SCZ > HC	
Anxiety dimension	1.00 ± 0.89*	1.15 ± 0.85***	1.62 ± 0.95**	0.13 ± 0.24	**F = 100.632**	**p < 0.001**	MDD > SCZ = BD > HC	
Hostility dimension	0.73 ± 0.88*	0.97 ± 0.94***	1.18 ± 0.94**	0.11 ± 0.21	**F = 53.672**	**p < 0.001**	BD = MDD > SCZ > HC	
Phobic anxiety dimension	0.74 ± 0.79*	0.73 ± 0.75***	1.14 ± 0.99**	0.05 ± 0.14	**F = 60.264**	**p < 0.001**	MDD > SCZ = BD > HC	
Paranoid ideation dimension	1.05 ± 0.97*	0.92 ± 0.83***	1.18 ± 0.91**	0.15 ± 0.29	**F = 54.784**	**p < 0.001**	SCZ, BD, MDD > HC	MDD > BD, MDD = SCZ, BD = SCZ
Psychoticism dimension	1.03 ± 0.88*	0.94 ± 0.75***	1.22 ± 0.79**	0.09 ± 0.20	**F = 81.867**	**p < 0.001**	SCZ, BD, MDD > HC	MDD > BD, MDD = SCZ, BD = SCZ
Overeating item	1.13 ± 1.18	1.17 ± 1.34***	1.10 ± 1.28**	0.34 ± 0.54	**F = 19.519**	**p < 0.001**	SCZ = BD = MDD > HC	

* n=1 missing
** n=2 missing
*** n=3 missing

† *P-value* (p) < 0.05 is considered statistically significant and is denoted in bold.

‡ Levels of statistical significance of post hoc analysis are represented as equality and inequality signs (=, >). = signifies no significance between disease types. > denotes relative significance between disease types.

Abbreviations

BPRS: Brief Psychiatric Rating Scale
YMRS: Young Mania Rating Scale
MADRS: Montgomery-Asberg Depression Rating Scale
HAM-A: Hamilton Anxiety Scale
MDD: major depressive disorder
BD: bipolar disorder
SCZ: schizophrenia
HC: healthy control
BMI: body mass index

Table S2. Demographics and clinical characteristics of the subjects of the training set†

Characteristics	SCZ n=102	BD n=102	MDD n=104	SCZ vs BD vs MDD		Post-hoc analysis‡
Sex: Male	41 (40.2%)	33 (32.4%)	38 (36.5%)	χ2 = 1.358	p = 0.51	
Age	40.37 ± 11.35	34.23 ± 12.58	36.32 ± 13.19	**F = 6.481**	**p = 0.002**	SCZ > BD
BMI	26.58 ± 4.84	24.42 ± 4.35	23.24 ± 3.99	**F = 15.242**	**p < 0.001**	SCZ > BD = MDD
Blood collection time : AM	49 (48.0%)	27 (26.5%)	30 (28.8%)	**χ2 = 12.669**	**p = 0.002**	
Fasting time : at least 8 hours	29 (28.4%)	20 (19.6%)	24 (23.1%)	χ2 = 2.230	p = 0.33	
Exercise : moderate	32 (31.4%)	37 (36.3%)	33 (31.7%)	χ2 = 0.689	p = 0.71	
Alcohol drinking : at least once a week	19 (18.6%)	37 (36.3%)	41 (39.4%)	**χ2 = 11.938**	**p = 0.003**	
Smoking : current smoker	24 (23.5%)	35 (34.3%)	35 (33.7%)	χ2 = 3.525	p = 0.17	
Duration from first onset	13.68 ± 10.38	10.41 ± 9.30	7.01 ± 8.06	**F = 13.275**	**p < 0.001**	SCZ > BD > MDD
Duration from first medication	13.04 ± 10.23	7.83 ± 9.07	3.56 ± 6.14	**F = 31.105**	**p < 0.001**	SCZ > BD > MDD
Medication						
Antipsychotics	99 (97.1%)	79 (77.5%)	43 (41.3%)	**χ2 = 81.304**	**p < 0.001**	
Lithium/Anticonvulsants	19 (18.6%)	69 (67.6%)	10 (9.6%)	**χ2 = 92.169**	**p < 0.001**	
Antidepressant	25 (24.5%)	29 (28.4%)	88 (84.6%)	**χ2 = 94.037**	**p < 0.001**	
Benzodiazepines/hypnotics	58 (56.9%)	63 (61.8%)	71 (68.3%)	χ2 = 2.875	p = 0.24	
Clinician rater score						
BPRS	44.31 ± 11.50	39.83 ± 7.51	40.41 ± 6.89	**F = 7.733**	**p = 0.001**	SCZ > BD = MDD
YMRS	4.64 ± 5.57	5.29 ± 6.11	1.96 ± 2.76	**F = 12.767**	**p < 0.001**	SCZ = BD > MDD
MADRS	14.22 ± 9.62	17.70 ± 10.40	26.06 ± 10.06	**F = 37.995**	**p < 0.001**	MDD > BD > SCZ
HAM-A	9.35 ± 7.33	10.06 ± 5.44	15.57 ± 7.22	**F = 26.455**	**p < 0.001**	MDD > SCZ = BD
Self-reported scale						
Symptom Checklist-90-Revised						
Somatization dimension	0.76 ± 0.68*	0.86 ± 0.70***	1.41 ± 0.95*	**F = 20.424**	**p < 0.001**	MDD > SCZ = BD
Obsessive-compulsive dimension	1.24 ± 0.83*	1.51 ± 0.89***	1.95 ± 0.87*	**F = 17.653**	**p < 0.001**	MDD > SCZ = BD
Interpersonal sensitivity dimension	1.26 ± 0.84*	1.31 ± 0.84***	1.68 ± 0.88*	**F = 7.617**	**p = 0.001**	MDD > SCZ = BD
Depression dimension	1.21 ± 0.90*	1.67 ± 0.96***	2.22 ± 0.93*	**F = 30.291**	**p < 0.001**	MDD > BD > SCZ
Anxiety dimension	1.02 ± 0.88*	1.14 ± 0.82***	1.63 ± 0.96*	**F = 13.719**	**p < 0.001**	MDD > SCZ = BD
Hostility dimension	0.71 ± 0.78*	1.00 ± 0.95***	1.19 ± 0.98*	**F = 7.096**	**p = 0.001**	MDD > SCZ
Phobic anxiety dimension	0.78 ± 0.80*	0.67 ± 0.69***	1.16 ± 1.01*	**F = 9.119**	**p < 0.001**	MDD > SCZ = BD
Paranoid ideation dimension	1.04 ± 0.87*	0.95 ± 0.82***	1.23 ± 0.96*	F = 2.516	p = 0.08	
Psychoticism dimension	1.02 ± 0.82*	0.93 ± 0.76***	1.22 ± 0.82*	**F = 3.434**	**p = 0.033**	MDD > BD
Overeating item	1.19 ± 1.16	0.98 ± 1.28***	1.06 ± 1.17*	F = 0.757	p = 0.47	

* n=1 missing
*** n=3 missing

† The *P-value* (p) < 0.05 is considered statistically significant, which is denoted by bold font.

‡ Levels of statistical significance of post-hoc analysis are represented as equality and inequality sign (=, >). The equality sign (=) signifies no statistical significance between disease types. The inequality sign (>) denotes relative statistical significance between disease types.

Abbreviations
BPRS: Brief Psychiatric Rating Scale
YMRS: Young Mania Rating Scale
MADRS: Montgomery-Asberg Depression Rating Scale
HAM-A: Hamilton Anxiety Scale
MDD: major depressive disorder
BD: bipolar disorder
SCZ: schizophrenia
BMI: body mass index

Table S3. Demographics and clinical characteristics of the subjects of the validation set†

Characteristics	SCZ n=34	BD n=34	MDD n=35	SCZ vs MDD vs BD		Post-hoc analysis‡
Sex: Male	15 (44.1%)	10 (29.4%)	8 (22.9%)	$\chi2 = 3.741$	$p = 0.15$	
Age	36.88 ± 11.91	37.21 ± 12.73	36.63 ± 12.94	F = 0.018	p = 0.98	
BMI	25.18 ± 5.38	23.87 ± 3.90	23.61 ± 4.08	F = 1.203	p = 0.31	
Blood collection time : AM	18 (52.9%)	10 (29.4%)	15 (42.9%)	$\chi2 = 3.897$	p = 0.14	
Fasting time : at least 8 hours	7 (20.6%)	3 (8.8%)	11 (31.4%)	$\chi2 = 5.431$	p = 0.07	
Exercise : moderate	13 (38.2%)	17 (50.0%)	9 (25.7%)	$\chi2 = 4.326$	p = 0.12	
Alcohol drinking : at least once a week	10 (29.4%)	14 (41.2%)	10 (28.6%)	$\chi2 = 1.536$	p = 0.46	
Smoking : current smoker	12 (35.3%)	6 (17.6%)	13 (37.1%)	$\chi2 = 3.767$	p = 0.15	
Duration from first onset	11.15 ± 9.07	9.12 ± 9.12	7.00 ± 7.67	F = 1.989	p = 0.14	
Duration from first medication	10.38 ± 9.54	7.56 ± 8.85	4.83 ± 6.06	**F = 3.889**	**p = 0.024**	SCZ > MDD
Medication						
Antipsychotics	34 (100.0%)	23 (67.6%)	17 (48.6%)	**$\chi2 = 22.992$**	**p < 0.001**	
Lithium/Anticonvulsants	4 (11.8%)	26 (76.5%)	6 (17.1%)	**$\chi2 = 38.702$**	**p < 0.001**	
Antidepressant	6 (17.6%)	10 (30.3%)	30 (85.7%)*	**$\chi2 = 36.585$**	**p < 0.001**	
Benzodiazepines/hypnotics	24 (70.6%)	19 (55.9%)	23 (65.7%)	$\chi2 = 1.659$	p = 0.44	
Clinician rater score						
BPRS	40.15 ± 11.82	36.09 ± 6.94	41.60 ± 6.93	**F = 3.574**	**p = 0.032**	MDD > BD
YMRS	3.47 ± 5.09	5.21 ± 6.07	1.51 ± 1.96	**F = 5.345**	**p = 0.006**	BD > MDD
MADRS	13.47 ± 11.16	15.21 ± 10.19	27.63 ± 8.96	**F = 20.112**	**p < 0.001**	MDD > SCZ = BD
HAMA	7.53 ± 5.82	8.50 ± 5.84	14.03 ± 5.71	**F = 12.712**	**p < 0.001**	MDD > SCZ = BD
Self-reported scale						
Symptom Checklist-90-Revised						
Somatization dimension	0.83 ± 0.80	0.87 ± 0.84	1.58 ± 0.89	**F = 8.642**	**p < 0.001**	MDD > SCZ = BD
Obsessive-compulsive dimension	1.42 ± 1.04	1.30 ± 0.85	2.06 ± 0.77	**F = 7.180**	**p = 0.001**	MDD > SCZ = BD
Interpersonal sensitivity dimension	1.25 ± 0.92	1.15 ± 0.82	1.84 ± 0.84	**F = 6.418**	**p = 0.002**	MDD > SCZ = BD
Depression dimension	1.45 ± 1.08	1.40 ± 0.88	2.46 ± 0.82	**F = 14.246**	**p < 0.001**	MDD > SCZ = BD
Anxiety dimension	1.09 ± 1.02	1.09 ± 0.95	1.89 ± 0.95	**F = 7.722**	**p = 0.001**	MDD > SCZ = BD
Hostility dimension	0.90 ± 1.09	0.84 ± 0.91	1.38 ± 0.90	**F = 3.175**	**p = 0.046**	MDD=BD=SCZ
Phobic anxiety dimension	0.70 ± 0.77	0.72 ± 0.93	1.29 ± 0.98	**F = 4.851**	**p = 0.010**	MDD > SCZ = BD
Paranoid ideation dimension	1.08 ± 1.12	0.87 ± 0.82	1.29 ± 0.79	F = 1.777	p = 0.17	
Psychoticism dimension	1.10 ± 1.02	0.91 ± 0.79	1.43 ± 0.65	**F = 3.432**	**p = 0.036**	MDD > BD
Overeating item	1.03 ± 1.24	1.62 ± 1.37	1.14 ± 1.40	F = 1.851	p = 0.16	

* n=1 missing

† The *P-value* (p) < 0.05 is considered statistically significant, which is denoted by bold font.

‡ Levels of statistical significance of post-hoc analysis are represented as equality and inequality sign (=, >). The equality sign (=) signifies no statistical significance between disease types. The inequality sign (>) denotes relative statistical significance between disease types.

Abbreviations
BPRS: Brief Psychiatric Rating Scale
YMRS: Young Mania Rating Scale
MADRS: Montgomery-Asberg Depression Rating Scale
HAM-A: Hamilton Anxiety Scale
MDD: major depressive disorder
BD: bipolar disorder
SCZ: schizophrenia
BMI: body mass index

Table S4. Demographics and clinical characteristics of the subjects of the independent test set†

Characteristics	SCZ n=35	BD n=34	MDD n=35	SCZ vs BD vs MDD		Post-hoc analysis‡
Sex: Male	18 (51.4%)	15 (44.1%)	15 (42.9%)	χ2 = 0.602	p = 0.74	
Age	38.06 ± 12.86	31.65 ± 10.39	35.94 ± 13.98	F = 2.342	p = 0.10	
BMI	24.33 ± 3.89	25.71 ± 4.92	23.96 ± 4.20	F = 1.547	p = 0.22	
Blood collection time : AM	14 (40.0%)	13 (38.2%)	10 (28.6%)	χ2 = 1.153	p = 0.56	
Fasting time : at least 8 hours	3 (8.6%)	10 (29.4%)	7 (20.0%)	χ2 = 4.843	p = 0.09	
Exercise : moderate	15 (42.9%)	14 (41.2%)	10 (28.6%)	χ2 = 1.815	p = 0.40	
Alcohol drinking : at least once a week	3 (8.6%)	9 (26.5%)	12 (34.3%)	**χ2 = 6.846**	**p = 0.033**	
Smoking : current smoker	5 (14.3%)	12 (35.3%)	11 (31.4%)	χ2 = 4.413	p = 0.11	
Duration from first onset	10.29 ± 9.55	6.59 ± 5.88	5.62 ± 6.93*	**F = 3.605**	**p = 0.031**	SCZ > MDD
Duration from first medication	9.17 ± 8.72	3.47 ± 3.34	2.91 ± 5.63	**F = 10.483**	**p < 0.001**	SCZ > BD = MDD
Medication						
Antipsychotics	33 (94.3%)	28 (82.4%)	12 (34.3%)	**χ2 = 33.681**	**p < 0.001**	
Lithium/Anticonvulsants	5 (14.3%)	30 (88.2%)	8 (22.9%)	**χ2 = 46.326**	**p < 0.001**	
Antidepressant	6 (17.1%)	5 (14.7%)	27 (77.1%)	**χ2 = 37.553**	**p < 0.001**	
Benzodiazepines/hypnotics	22 (62.9%)	24 (70.6%)	23 (65.7%)	χ2 = 0.471	p = 0.79	
Clinician rater score						
BPRS	45.09 ± 13.16	41.76 ± 10.10	40.23 ± 7.41	F = 1.958	p = 0.15	
YMRS	5.06 ± 7.07	7.29 ± 9.64	1.94 ± 2.46	**F = 5.069**	**p = 0.008**	BD > MDD
MADRS	12.89 ± 7.56	19.09 ± 10.96	24.43 ± 10.29	**F = 12.406**	**p < 0.001**	BD = MDD > SCZ
HAMA	8.17 ± 5.22	10.71 ± 7.44	13.97 ± 8.41	**F = 5.797**	**p = 0.004**	MDD > SCZ
Self-reported scale						
Symptom Checklist-90-Revised						
Somatization dimension	0.75 ± 0.88	0.85 ± 0.75	1.16 ± 0.94*	F = 2.147	p = 0.12	
Obsessive-compulsive dimension	1.14 ± 0.94	1.60 ± 0.82	1.56 ± 0.75*	**F = 3.129**	**p = 0.048**	MDD=BD=SCZ
Interpersonal sensitivity dimension	1.11 ± 1.03	1.39 ± 0.90	1.25 ± 0.97*	F = 0.727	p = 0.49	
Depression dimension	1.02 ± 0.97	1.62 ± 0.96	2.00 ± 0.93*	**F = 9.262**	**p < 0.001**	BD = MDD > SCZ
Anxiety dimension	0.85 ± 0.81	1.23 ± 0.83	1.31 ± 0.86*	F = 2.976	p = 0.06	
Hostility dimension	0.62 ± 0.90	0.99 ± 0.97	0.96 ± 0.84*	F = 1.763	p = 0.18	
Phobic anxiety dimension	0.63 ± 0.78	0.91 ± 0.68	0.91 ± 0.92*	F = 1.368	p = 0.26	
Paranoid ideation dimension	1.05 ± 1.12	0.91 ± 0.89	0.91 ± 0.83*	F = 0.242	p = 0.79	
Psychoticism dimension	1.02 ± 0.92	0.97 ± 0.71	1.02 ± 0.78*	F = 0.040	p = 0.96	
Overeating item	1.06 ± 1.21	1.26 ± 1.42	1.18 ± 1.51*	F = 0.196	p = 0.82	

* n=1 missing

† The *P-value* (p) < 0.05 is considered statistically significant, which is denoted by bold font.

‡ Levels of statistical significance of post-hoc analysis are represented as equality and inequality sign (=, >). The equality sign (=) signifies no statistical significance between disease types. The inequality sign (>) denotes relative statistical significance between disease types.

Abbreviations
BPRS: Brief Psychiatric Rating Scale
YMRS: Young Mania Rating Scale
MADRS: Montgomery-Asberg Depression Rating Scale
HAM-A: Hamilton Anxiety Scale
MDD: major depressive disorder
BD: bipolar disorder
SCZ: schizophrenia
BMI: body mass index

Table S5. Proteomic candidate features for each pairwise comparison of groups originating from training sets[†]

MDD vs BD (23)		
Uniprot accession number	Protein	peptide
P09972	ALDOC	ALQASALNAWR
P15144	AMPN	AQIINDAFNLASAHK
Q86TY3	ARMD4	TVVPSITR
Q9NZP8	C1RL	GSEAINAPGDNPAK
P55290	CAD13	INNTHALVSLLQNLNK
P08571	CD14	VLDLSCNR
P11597	CETP	ASYPDITGEK
Q96KN2	CNDP1	AIHLDLEEYR
Q03692	COAA1	GTHVWVGLYK
O60716	CTND1	GYELLFQPEVVR
Q08345	DDR1	LHLVALVGTQGR
P28845	DHI1	VIVTGASK
P09172	DOPO	TPEGLTLLFK
P05155	IC1	TTFDPK
Q9NPH3	IL1AP	NEVWWTIDGK
P19823	ITIH2	IQPSGGTNINEALLR
P13473	LAMP2	IPLNDLFR
P61916	NPC2	LVVEWQLQDDK
O60486	PLXC1	LNTIGHYEISNGSTIK
P62826	RAN	FNVWDTAGQEK
P0DJI8	SAA1	FFGHGAEDSLADQAANEWGR
Q01082	SPTB2	LTVQTK
P02787	TRFE	ASYLDCIR

MDD vs SCZ (29)		
Uniprot accession number	Protein	peptide
P04217	A1BG	LLELTGPK
P19823	ITIH2	IQPSGGTNINEALLR
P52758	RIDA	AAYQVAALPK
P35542	SAA4	GPGGVWAAK
P09972	ALDOC	ALQASALNAWR
P24593	IBP5	AVYLPNCDR
P07225	PROS	NNLELSTPLK
P35858	ALS	DFALQNPSAVPR
P17936	IBP3	YGQPLPGYTTK
P04070	PROC	TFVLNFIK
P08185	CBG	HLVALSPK
P22352	GPX3	FYTFLK
Q15262	PTPRK	QNVVDVFHAVK
P15144	AMPN	AQIINDAFNLASAHK
Q96IY4	CBPB2	DTGTYGFLLPER
Q9Y210	TRPC6	LGILGSHEDLSK
P10646	TFPI1	IAYEEIFVK
P04278	SHBG	TSSSFEVR
P54802	ANAG	DFCGCHVAWSGSQLR
Q03692	COAA1	GTHVWVGLYK
P0DJI8	SAA1	FFGHGAEDSLADQAANEWGR
Q8IYB8	SUV3	LLNLEGFPSGSQSR
Q86TY3	ARMD4	TVVPSITR
P25774	CATS	YTELPYGR
P05023	AT1A1	IVEIPFNSTNK
P20142	PEPC	AECGLGVPTTR
Q14894	CRYM	TVVPVTK
Q13976	KGP1	EEEIQELK
P14618	KPYM	IYVDDGLISLQVK

BD vs SCZ (30)		
Uniprot accession number	Protein	peptide
P04217	A1BG	LLELTGPK
P02787	TRFE	ASYLDCIR
Q99460	PSMD1	VSTAVLSITAK
P05546	HEP2	TLEAQLTPR
P05155	IC1	TTFDPK
P01011	AACT	DEELSCTVVELK
Q8TDL5	BPIB1	ALGFEAAESSLTK
P08185	CBG	HLVALSPK
P00751	CFAB	DISEVVTPR
P24593	IBP5	AVYLPNCDR
P13473	LAMP2	IPLNDLFR
Q8IYB8	SUV3	LLNLEGFPSGSQSR
Q01082	SPTB2	LTVQTK
P15144	AMPN	AQIINDAFNLASAHK
P55290	CAD13	INNTHALVSLLQNLNK
P09172	DOPO	VISTLEEPTPQCPTSQGR
O15551	CLD3	DFYNPVVPEAQK
Q08345	DDR1	LHLVALVGTQGR
P07911	UROM	VLNLGPITR
O15354	GPR37	ISPDLPDTIYVLALTYDSAR
Q13283	G3BP1	AVYLPNCDR
O60486	PLXC1	LNTIGHYEISNGSTIK
Q9NZP8	C1RL	GSEAINAPGDNPAK
Q96IY4	CBPB2	DTGTYGFLLPER
Q96KN2	CNDP1	AIHLDLEEYR
P10646	TFPI1	IAYEEIFVK
Q9NY15	STAB1	SLEAQGNSSHLDADTVR
P62888	RL30	SLESINSR
P61916	NPC2	LVVEWQLQDDK
P11226	MBL2	FQASVATPR

[†] Uniprot accession number, protein entry, and peptide sequence for proteomic candidate features in each pairwise comparison of groups.

Abbreviations
MDD: major depressive disorder, BD: bipolar disorder, SCZ: schizophrenia

Table S6. Summary of unique models originating from combinations of selected features for each pairwise comparison of groups†

MDD vs BD	Model #	Unique model (combination of selected features)	Number of features combined	Model Frequency	Model probability
	1	ALDOC_ALQASALNAWR+ AMPN_AQIINDAFNLASAHK+ ARMD4_TVVPSITR+ C1RL_GSEAINAPGDNPAK+ CAD13_INNTHALVSLLQNLNK+ CD14_VLDLSCNR+ CETP_ASYPDITGEK+ CNDP1_AIHLDLEEYR+ COAA1_GTHVWVGLYK+ CTND1_GYELLFQPEVVR+ DDR1_LHLVALVGTQGR+ DHI1_VIVTGASK+ DOPO_TPEGLTLLFK+ IC1_TTFDPK+ IL1AP_NEVWWTIDGK+ ITIH2_IQPSGGTNINEALLR+ LAMP2_IPLNDLFR+ NPC2_LVVEWQLQDDK+ PLXC1_LNTIGHYEISNGSTIK+ RAN_FNVWDTAGQEK+ SAA1_FFGHGAEDSLADQAANEWGR+ SPTB2_LTVQTK+ TRFE_ASYLDCIR+(intercept)	23	97	9.800E-01
	2	ALDOC_ALQASALNAWR+ AMPN_AQIINDAFNLASAHK+ ARMD4_TVVPSITR+ C1RL_GSEAINAPGDNPAK+ CAD13_INNTHALVSLLQNLNK+ CETP_ASYPDITGEK+ CNDP1_AIHLDLEEYR+ COAA1_GTHVWVGLYK+ CTND1_GYELLFQPEVVR+ DDR1_LHLVALVGTQGR+ DHI1_VIVTGASK+ DOPO_TPEGLTLLFK+ IC1_TTFDPK+ IL1AP_NEVWWTIDGK+ ITIH2_IQPSGGTNINEALLR+ LAMP2_IPLNDLFR+ NPC2_LVVEWQLQDDK+ PLXC1_LNTIGHYEISNGSTIK+ RAN_FNVWDTAGQEK+ SAA1_FFGHGAEDSLADQAANEWGR+ SPTB2_LTVQTK+ TRFE_ASYLDCIR+(intercept)	22	1	3.19E-05
	3	ALDOC_ALQASALNAWR+ AMPN_AQIINDAFNLASAHK+ ARMD4_TVVPSITR+ C1RL_GSEAINAPGDNPAK+ CAD13_INNTHALVSLLQNLNK+ CETP_ASYPDITGEK+ COAA1_GTHVWVGLYK+ CTND1_GYELLFQPEVVR+ DDR1_LHLVALVGTQGR+ DOPO_TPEGLTLLFK+ IC1_TTFDPK+ IL1AP_NEVWWTIDGK+ ITIH2_IQPSGGTNINEALLR+ NPC2_LVVEWQLQDDK+ RAN_FNVWDTAGQEK+ SAA1_FFGHGAEDSLADQAANEWGR+ TRFE_ASYLDCIR+(intercept)	17	1	4.69E-09
	4	ALDOC_ALQASALNAWR+ AMPN_AQIINDAFNLASAHK+ ARMD4_TVVPSITR+ C1RL_GSEAINAPGDNPAK+ CAD13_INNTHALVSLLQNLNK+ CETP_ASYPDITGEK+ COAA1_GTHVWVGLYK+ CTND1_GYELLFQPEVVR+ DDR1_LHLVALVGTQGR+ DOPO_TPEGLTLLFK+ IC1_TTFDPK+ IL1AP_NEVWWTIDGK+ ITIH2_IQPSGGTNINEALLR+ NPC2_LVVEWQLQDDK+ RAN_FNVWDTAGQEK+ SAA1_FFGHGAEDSLADQAANEWGR+ TRFE_ASYLDCIR+ LAMP2_IPLNDLFR+(intercept)	18	1	5.65E-06

MDD vs SCZ	Model #	Unique model (combination of selected features)	Number of features combined	Model Frequency	Model probability

1	ALDOC_ALQASALNAWR+ ALS_DFALQNPSAVPR+ ANAG_DFCGCHVAWSGSQLR+ AT1A1_IVEIPFNSTNK+ CATS_YTELPYGR+ CBG_HLVALSPK+ CBPB2_DTGTYGFLLPER+ COAA1_GTHVWVGLYK+ CRYM_TVVPVTK+ GPX3_FYTFLK+ IBP3_YGQPLPGYTTK+ IBP5_AVYLPNCDR+ ITIH2_IQPSGGTNINEALLR+ PEPC_AECGLGVPTTR+ PROC_TFVLNFIK+ PROS_NNLELSTPLK+ PTPRK_QNVVDVFHAVK+ RIDA_AAYQVAALPK+ SAA1_FFGHGAEDSLADQAANEWGR+ SAA4_GPGGVWAAK+ TFPI1_IAYEEIFVK+ (intercept)	22	10	6.86E-03
2	A1BG_LLELTGPK+ ALDOC_ALQASALNAWR+ ALS_DFALQNPSAVPR+ AMPN_AQIINDAFNLASAHK+ ANAG_DFCGCHVAWSGSQLR+ AT1A1_IVEIPFNSTNK+ CATS_YTELPYGR+ CBG_HLVALSPK+ CBPB2_DTGTYGFLLPER+ COAA1_GTHVWVGLYK+ CRYM_TVVPVTK+ GPX3_FYTFLK+ IBP3_YGQPLPGYTTK+ IBP5_AVYLPNCDR+ ITIH2_IQPSGGTNINEALLR+ KGP1_EEEIQELK+ KPYM_IYVDDGLISLQVK+ PEPC_AECGLGVPTTR+ PROC_TFVLNFIK+ PROS_NNLELSTPLK+ PTPRK_QNVVDVFHAVK+ RIDA_AAYQVAALPK+ SAA1_FFGHGAEDSLADQAANEWGR+ SAA4_GPGGVWAAK+ SHBG_TSSSFEVR+ SUV3_LLNLEGFPSGSQSR+ TFPI1_IAYEEIFVK+ (Intercept)	28	13	1.56E-01
3	A1BG_LLELTGPK+ ALDOC_ALQASALNAWR+ ALS_DFALQNPSAVPR+ AMPN_AQIINDAFNLASAHK+ ANAG_DFCGCHVAWSGSQLR+ ARMD4_TVVPSITR+ AT1A1_IVEIPFNSTNK+ CATS_YTELPYGR+ CBG_HLVALSPK+ CBPB2_DTGTYGFLLPER+ COAA1_GTHVWVGLYK+ CRYM_TVVPVTK+ GPX3_FYTFLK+ IBP3_YGQPLPGYTTK+ IBP5_AVYLPNCDR+ ITIH2_IQPSGGTNINEALLR+ KGP1_EEEIQELK+ KPYM_IYVDDGLISLQVK+ PEPC_AECGLGVPTTR+ PROC_TFVLNFIK+ PROS_NNLELSTPLK+ PTPRK_QNVVDVFHAVK+ RIDA_AAYQVAALPK+ SAA1_FFGHGAEDSLADQAANEWGR+ SAA4_GPGGVWAAK+ SHBG_TSSSFEVR+ SUV3_LLNLEGFPSGSQSR+ TFPI1_IAYEEIFVK+ TRPC6_LGILGSHEDLSK+ (intercept)	30	21	3.23E-01
4	A1BG_LLELTGPK+ ALDOC_ALQASALNAWR+ ALS_DFALQNPSAVPR+ AMPN_AQIINDAFNLASAHK+ ANAG_DFCGCHVAWSGSQLR+ AT1A1_IVEIPFNSTNK+ CATS_YTELPYGR+ CBG_HLVALSPK+ CBPB2_DTGTYGFLLPER+ COAA1_GTHVWVGLYK+ CRYM_TVVPVTK+ GPX3_FYTFLK+ IBP3_YGQPLPGYTTK+ IBP5_AVYLPNCDR+ ITIH2_IQPSGGTNINEALLR+ PEPC_AECGLGVPTTR+ PROC_TFVLNFIK+ PROS_NNLELSTPLK+ PTPRK_QNVVDVFHAVK+ RIDA_AAYQVAALPK+ SAA1_FFGHGAEDSLADQAANEWGR+ SAA4_GPGGVWAAK+ SHBG_TSSSFEVR+ SUV3_LLNLEGFPSGSQSR+ TFPI1_IAYEEIFVK+ (intercept)	26	7	6.00E-02
5	ALDOC_ALQASALNAWR+ ALS_DFALQNPSAVPR+ AMPN_AQIINDAFNLASAHK+ ANAG_DFCGCHVAWSGSQLR+ AT1A1_IVEIPFNSTNK+ CATS_YTELPYGR+ CBG_HLVALSPK+ CBPB2_DTGTYGFLLPER+ COAA1_GTHVWVGLYK+ CRYM_TVVPVTK+ GPX3_FYTFLK+ IBP3_YGQPLPGYTTK+ IBP5_AVYLPNCDR+ ITIH2_IQPSGGTNINEALLR+ PEPC_AECGLGVPTTR+ PROC_TFVLNFIK+ PROS_NNLELSTPLK+ PTPRK_QNVVDVFHAVK+ RIDA_AAYQVAALPK+ SAA1_FFGHGAEDSLADQAANEWGR+ SAA4_GPGGVWAAK+ SHBG_TSSSFEVR+ SUV3_LLNLEGFPSGSQSR+ TFPI1_IAYEEIFVK+ (intercept)	25	16	7.24E-02

	6	A1BG_LLELTGPK+ ALDOC_ALQASALNAWR+ ALS_DFALQNPSAVPR+ AMPN_AQIINDAFNLASAHK+ ANAG_DFCGCHVAWSGSQLR+ ARMD4_TVVPSITR+ AT1A1_IVEIPFNSTNK+ CATS_YTELPYGR+ CBG_HLVALSPK+ CBPB2_DTGTYGFLLPER+ COAA1_GTHVWVGLYK+ CRYM_TVVPVTK+ GPX3_FYTFLK+ IBP3_YGQPLPGYTTK+ IBP5_AVYLPNCDR+ ITIH2_IQPSGGTNINEALLR+ KGP1_EEEIQELK+ KPYM_IYVDDGLISLQVK+ PEPC_AECGLGVPTTR+ PROC_TFVLNFIK+ PROS_NNLELSTPLK+ PTPRK_QNVVDVFHAVK+ RIDA_AAYQVAALPK+ SAA1_FFGHGAEDSLADQAANEWGR+ SAA4_GPGGVWAAK+ SHBG_TSSSFEVR+ SUV3_LLNLEGFPSGSQSR+ TFPI1_IAYEEIFVK+ (intercept)	29	17	2.91E-01
	7	A1BG_LLELTGPK+ ALDOC_ALQASALNAWR+ ALS_DFALQNPSAVPR+ AMPN_AQIINDAFNLASAHK+ ANAG_DFCGCHVAWSGSQLR+ AT1A1_IVEIPFNSTNK+ CATS_YTELPYGR+ CBG_HLVALSPK+ CBPB2_DTGTYGFLLPER+ COAA1_GTHVWVGLYK+ CRYM_TVVPVTK+ GPX3_FYTFLK+ IBP3_YGQPLPGYTTK+ IBP5_AVYLPNCDR+ ITIH2_IQPSGGTNINEALLR+ KGP1_EEEIQELK+ PEPC_AECGLGVPTTR+ PROC_TFVLNFIK+ PROS_NNLELSTPLK+ PTPRK_QNVVDVFHAVK+ RIDA_AAYQVAALPK+ SAA1_FFGHGAEDSLADQAANEWGR+ SAA4_GPGGVWAAK+ SHBG_TSSSFEVR+ SUV3_LLNLEGFPSGSQSR+ TFPI1_IAYEEIFVK+ (intercept)	27	10	8.74E-02
	8	ALDOC_ALQASALNAWR+ ALS_DFALQNPSAVPR+ ANAG_DFCGCHVAWSGSQLR+ AT1A1_IVEIPFNSTNK+ CATS_YTELPYGR+ CBG_HLVALSPK+ CBPB2_DTGTYGFLLPER+ COAA1_GTHVWVGLYK+ CRYM_TVVPVTK+ GPX3_FYTFLK+ IBP3_YGQPLPGYTTK+ IBP5_AVYLPNCDR+ ITIH2_IQPSGGTNINEALLR+ PEPC_AECGLGVPTTR+ PROC_TFVLNFIK+ PROS_NNLELSTPLK+ PTPRK_QNVVDVFHAVK+ RIDA_AAYQVAALPK+ SAA1_FFGHGAEDSLADQAANEWGR+ SAA4_GPGGVWAAK+ SHBG_TSSSFEVR+ SUV3_LLNLEGFPSGSQSR+ TFPI1_IAYEEIFVK+ (intercept)	24	4	2.71E-03
	9	ALDOC_ALQASALNAWR+ ALS_DFALQNPSAVPR+ ANAG_DFCGCHVAWSGSQLR+ AT1A1_IVEIPFNSTNK+ CATS_YTELPYGR+ CBG_HLVALSPK+ CBPB2_DTGTYGFLLPER+ COAA1_GTHVWVGLYK+ CRYM_TVVPVTK+ GPX3_FYTFLK+ IBP3_YGQPLPGYTTK+ IBP5_AVYLPNCDR+ ITIH2_IQPSGGTNINEALLR+ PEPC_AECGLGVPTTR+ PROC_TFVLNFIK+ PROS_NNLELSTPLK+ RIDA_AAYQVAALPK+ SAA1_FFGHGAEDSLADQAANEWGR+ SAA4_GPGGVWAAK+ TFPI1_IAYEEIFVK+ (intercept)	21	2	2.06E-06

**BD vs SCZ**	**Model #**	**Unique model (combination of selected features)**	**Number of features combined**	**Model Frequency**	**Model probability**
	1	AACT_DEELSCTVVELK+AMPN_AQIINDAFNLASAHK+BPIB1_ALGFEAAESSLTK+C1RL_GSEAINAPGDNPAK+CBG_HLVALSPK+CBPB2_DTGTYGFLLPER+CFAB_DISEVVTPR+CLD3_DFYNPVVPEAQK+CNDP1_AIHLDLEEYR+DDR1_LHLVALVGTQGR+DOPO_VISTLEEPTPQCPTSQGR+G3BP1_INIPPQR+GPR37_ISPDLPDTIYVLALTYDSAR+HEP2_TLEAQLTPR+IBP5_AVYLPNCDR+IC1_TTFDPK+LAMP2_IPLNDLFR+MBL2_FQASVATPR+NPC2_LVVEWQLQDDK+PLXC1_LNTIGHYEISNGSTIK+PSMD1_VSTAVLSITAK+RL30_SLESINSR+SPTB2_LTVQTK+SUV3_LLNLEGFPSGSQSR+TFPI1_IAYEEIFVK+TRFE_ASYLDCIR+UROM_VLNLGPITR+(intercept)	28	9	9.87E-02

2	AACT_DEELSCTVVELK+AMPN_AQIINDAFNLASAHK+BPIB1_ALGFEAAESSLTK+C1RL_GSEAINAPGDNPAK+CBG_HLVALSPK+CBPB2_DTGTYGFLLPER+CFAB_DISEVVTPR+CLD3_DFYNPVVPEAQK+CNDP1_AIHLDLEEYR+DDR1_LHLVALVGTQGR+DOPO_VISTLEEPTPQCPTSQGR+G3BP1_INIPPQR+GPR37_ISPDLPDTIYVLALTYDSAR+HEP2_TLEAQLTPR+IBP5_AVYLPNCDR+IC1_TTFDPK+LAMP2_IPLNDLFR+MBL2_FQASVATPR+NPC2_LVVEWQLQDDK+PLXC1_LNTIGHYEISNGSTIK+PSMD1_VSTAVLSITAK+RL30_SLESINSR+SPTB2_LTVQTK+TFPI1_IAYEEIFVK+TRFE_ASYLDCIR+UROM_VLNLGPITR+(intercept)	27	17	3.17E-01
3	AACT_DEELSCTVVELK+AMPN_AQIINDAFNLASAHK+BPIB1_ALGFEAAESSLTK+C1RL_GSEAINAPGDNPAK+CAD13_INNTHALVSLLQNLNK+CBG_HLVALSPK+CBPB2_DTGTYGFLLPER+CFAB_DISEVVTPR+CLD3_DFYNPVVPEAQK+CNDP1_AIHLDLEEYR+DDR1_LHLVALVGTQGR+DOPO_VISTLEEPTPQCPTSQGR+G3BP1_INIPPQR+GPR37_ISPDLPDTIYVLALTYDSAR+HEP2_TLEAQLTPR+IBP5_AVYLPNCDR+IC1_TTFDPK+LAMP2_IPLNDLFR+MBL2_FQASVATPR+NPC2_LVVEWQLQDDK+PLXC1_LNTIGHYEISNGSTIK+PSMD1_VSTAVLSITAK+RL30_SLESINSR+SPTB2_LTVQTK+SUV3_LLNLEGFPSGSQSR+TFPI1_IAYEEIFVK+TRFE_ASYLDCIR+UROM_VLNLGPITR+(intercept)	29	39	2.65E-01
4	AACT_DEELSCTVVELK+AMPN_AQIINDAFNLASAHK+BPIB1_ALGFEAAESSLTK+C1RL_GSEAINAPGDNPAK+CBG_HLVALSPK+CBPB2_DTGTYGFLLPER+CFAB_DISEVVTPR+CLD3_DFYNPVVPEAQK+CNDP1_AIHLDLEEYR+DDR1_LHLVALVGTQGR+DOPO_VISTLEEPTPQCPTSQGR+G3BP1_INIPPQR+GPR37_ISPDLPDTIYVLALTYDSAR+HEP2_TLEAQLTPR+IBP5_AVYLPNCDR+IC1_TTFDPK+LAMP2_IPLNDLFR+MBL2_FQASVATPR+NPC2_LVVEWQLQDDK+PLXC1_LNTIGHYEISNGSTIK+PSMD1_VSTAVLSITAK+SPTB2_LTVQTK+TFPI1_IAYEEIFVK+TRFE_ASYLDCIR+UROM_VLNLGPITR+(intercept)	26	27	3.07E-01
5	AACT_DEELSCTVVELK+AMPN_AQIINDAFNLASAHK+BPIB1_ALGFEAAESSLTK+C1RL_GSEAINAPGDNPAK+CAD13_INNTHALVSLLQNLNK+CBG_HLVALSPK+CBPB2_DTGTYGFLLPER+CFAB_DISEVVTPR+CLD3_DFYNPVVPEAQK+CNDP1_AIHLDLEEYR+DDR1_LHLVALVGTQGR+DOPO_VISTLEEPTPQCPTSQGR+G3BP1_INIPPQR+GPR37_ISPDLPDTIYVLALTYDSAR+HEP2_TLEAQLTPR+IBP5_AVYLPNCDR+IC1_TTFDPK+LAMP2_IPLNDLFR+MBL2_FQASVATPR+NPC2_LVVEWQLQDDK+PLXC1_LNTIGHYEISNGSTIK+PSMD1_VSTAVLSITAK+RL30_SLESINSR+SPTB2_LTVQTK+STAB1_SLEAQGNSSHLDADTVR+SUV3_LLNLEGFPSGSQSR+TFPI1_IAYEEIFVK+TRFE_ASYLDCIR+UROM_VLNLGPITR+(intercept)	30	5	1.30E-02
6	AACT_DEELSCTVVELK+AMPN_AQIINDAFNLASAHK+BPIB1_ALGFEAAESSLTK+C1RL_GSEAINAPGDNPAK+CBPB2_DTGTYGFLLPER+CFAB_DISEVVTPR+CLD3_DFYNPVVPEAQK+DOPO_VISTLEEPTPQCPTSQGR+G3BP1_INIPPQR+GPR37_ISPDLPDTIYVLALTYDSAR+HEP2_TLEAQLTPR+IBP5_AVYLPNCDR+IC1_TTFDPK+LAMP2_IPLNDLFR+MBL2_FQASVATPR+NPC2_LVVEWQLQDDK+PLXC1_LNTIGHYEISNGSTIK+PSMD1_VSTAVLSITAK+TFPI1_IAYEEIFVK+TRFE_ASYLDCIR+UROM_VLNLGPITR+(intercept)	22	2	2.88E-09

7	AACT_DEELSCTVVELK+AMPN_AQIINDAFNLASAHK+BPIB1_ALGFEAAESSLTK+C1RL_GSEAINAPGDNPAK+CFAB_DISEVVTPR+CLD3_DFYNPVVPEAQK+DOPO_VISTLEEPTPQCPTSQGR+GPR37_ISPDLPDTIYVLALTYDSAR+HEP2_TLEAQLTPR+IBP5_AVYLPNCDR+IC1_TTFDPK+MBL2_FQASVATPR+NPC2_LVVEWQLQDDK+PLXC1_LNTIGHYEISNGSTIK+PSMD1_VSTAVLSITAK+TFPI1_IAYEEIFVK+UROM_VLNLGPITR+(intercept)	18	1	2.81E-11

† Unique models were generated for each comparison of groups. Number of features, frequency, and model probability for each unique model are listed. Protein_peptide sequence of the components is listed for each combination.

Abbreviations

MDD: major depressive disorder, BD: bipolar disorder, SCZ: schizophrenia

Table S7. Covariate analysis of proteomic features (proteins) of MPM models that showed statistically significant correlation with clinical variables in the training set†

MPM model	Proteomic features (proteins)	Covariates	P-value (ANCOVA)
MDD vs BD	ALDOC_ALQASALNAWR	Group (MDD vs BD)	0.121
		SCL_PAR	**0.011**
	ALDOC_ALQASALNAWR	Group (MDD vs BD)	0.080
		SCL_ANG	**0.049**
	ARMD4_TVVPSITR	Group (MDD vs BD)	0.073
		SCL_OCD	**0.022**
	CTND1_GYELLFQPEVVR	Group (MDD vs BD)	0.053
		BPRS	**0.036**
MDD vs SCZ	ALDOC_ALQASALNAWR	Group (MDD vs SCZ)	0.124
		SCL_PSY	**0.015**
	ALDOC_ALQASALNAWR	Group (MDD vs SCZ)	0.112
		SCL_PAR	**0.025**
	ALDOC_ALQASALNAWR	Group (MDD vs SCZ)	0.267
		SCL_ANG	**0.011**
	IBP3_YGQPLPGYTTK	Group (MDD vs SCZ)	0.173
		SCL_OCD	**0.008**
BD vs SCZ	GPR37_ ISPDLPDTIYVLALTYDSAR	Group (BD vs SCZ)	0.113
		BPRS	**0.024**
	UROM_ VLNLGPITR	Group (BD vs SCZ)	0.141
		BPRS	**0.039**

† Analysis of covariance (ANCOVA) was performed to assess the potential influence of covariates that correlated significantly with the proteomic features (proteins) in each pairwise MPM model. Bold font denotes statistical significance at *P-value* < 0.05. Protein_peptide sequence is listed for each feature.

Abbreviations

MDD: major depressive disorder, BD: bipolar disorder, SCZ: schizophrenia, SCL: Symptom Checklist-90-Revised, BPRS: Brief Psychiatric Rating Scale, PAR: paranoid ideation dimension, ANG: hostility dimension, OCD: obsessive-compulsive dimension, PSY: psychoticism dimension, MPM: multiprotein marker

Table S8. Information on mass spectra of the proteins of the MPM models for each pairwise comparison of groups†

MDD vs BD				Mass Information								
**Uniprot accession number**	**Protein**	**Peptide**	**Gene name**	**Precursor ion. Light (m/z)**	**Precursor ion. Heavy (m/z)**	**Precursor ion charge**	**Product ion. Light (m/z)**	**Product ion. Heavy (m/z)**	**Product ion charge**	**Product ion type**	**Collision energy (volt)**	**Retention time (RT)**
P09972	ALDOC	ALQASALNAWR	ALDOC	400.89	404.22	3	508.77	513.77	2.00	y9	9.60	37.47+/-0.49
P15144	AMPN	AQIINDAFNLASAHK	ANPEP	734.40	739.40	2	277.15	277.15	1.00	b2	23.80	42.82+/-0.3
Q86TY3	ARMD4	TVVPSITR	ARMH4	436.76	441.77	2	201.12	201.12	1.00	b2	14.50	28.73+/-0.19
Q9NZP8	C1RL	GSEAINAPGDNPAK	C1RL	670.83	674.83	2	698.35	706.36	1.00	y7	21.80	24.58+/-0.14
P55290	CAD13	INNTHALVSLLQNLNK	CDH13	598.01	600.68	3	729.43	737.44	1.00	y6	16.70	44.85+/-0.36
P11597	CETP	ASYPDITGEK	CETP	540.76	544.77	2	759.39	767.40	1.00	y7	17.80	27.87+/-0.17
Q03692	COAA1	GTHVWVGLYK	COL10A1	387.21	389.88	3	480.28	488.30	1.00	y4	9.10	33.4+/-0.52
O60716	CTND1	GYELLFQPEVVR	CTNND1	725.39	730.39	2	599.35	609.36	1.00	y5	23.50	44.94+/-0.5
Q08345	DDR1	LHLVALVGTQGR	DDR1	632.38	637.38	2	1013.61	1023.62	1.00	y10	20.60	34.94+/-0.25
P09172	DOPO	TPEGLTLLFK	DBH	559.83	563.83	2	509.30	513.31	2.00	y9	18.40	44.83+/-0.31
P05155	IC1	TTFDPK	SERPING1	354.68	358.69	2	244.17	252.18	1.00	y2	12.00	22.55+/-0.76
Q9NPH3	IL1AP	NEVWWTIDGK	IL1RAP	624.31	628.31	2	905.45	913.47	1.00	y7	20.40	40.99+/-0.33
P19823	ITIH2	IQPSGGTNINEALLR	ITIH2	791.93	796.94	2	671.36	676.36	2.00	y13	25.50	35.16+/-0.23
P61916	NPC2	LVVEWQLQDDK	NPC2	458.24	460.91	3	505.23	513.24	1.00	y4	11.70	38.92+/-0.52
P62826	RAN	FNVWDTAGQEK	RAN	647.81	651.81	2	633.32	641.33	1.00	y6	21.10	35.37+/-0.41
P0DJI8	SAA1	FFGHGAEDSLADQAANEWGR	SAA1	726.66	730.00	3	732.34	742.35	1.00	y6	21.40	39.78+/-0.29
P02787	TRFE	ASYLDCIR	TF	499.24	504.25	2	563.26	573.27	1.00	y4	16.50	32.04+/-0.2

MDD vs SCZ				Mass Information								
**Uniprot accession number**	**Protein**	**Peptide**	**Gene name**	**Precursor ion. Light (m/z)**	**Precursor ion. Heavy (m/z)**	**Precursor ion charge**	**Product ion. Light (m/z)**	**Product ion. Heavy (m/z)**	**Product ion charge**	**Product ion type**	**Collision energy (volt)**	**Retention time (RT)**
P09972	ALDOC	ALQASALNAWR	ALDOC	400.89	404.22	3	508.77	513.77	2.00	y9	9.60	37.47+/-0.49
P35858	ALS	DFALQNPSAVPR	IGFALS	657.84	662.85	2	626.36	636.37	1.00	y6	21.40	34.77+/-0.45
P54802	ANAG	DFCGCHVAWSGSQLR	NAGLU	593.93	597.26	3	759.34	764.34	2.00	y13	16.60	34.99+/-0.28
P05023	AT1A1	IVEIPFNSTNK	ATP1A1	631.34	635.35	2	342.20	342.20	1.00	b3	20.60	35.32+/-0.27
P25774	CATS	YTELPYGR	CTSS	499.75	504.75	2	734.38	744.39	1.00	y6	16.50	28.78+/-0.18
P08185	CBG	HLVALSPK	SERPINA6	432.77	436.78	2	251.15	251.15	1.00	b2	14.40	25.22+/-0.23
Q96IY4	CBPB2	DTGTYGFLLPER	CPB2	456.90	460.23	3	401.21	411.22	1.00	y3	11.60	41.48+/-0.35
Q03692	COAA1	GTHVWVGLYK	COL10A1	387.21	389.88	3	480.28	488.30	1.00	y4	9.10	33.4+/-0.52
Q14894	CRYM	TVVPVTK	CRYM	372.24	376.24	2	201.12	201.12	1.00	b2	12.50	24.77+/-0.28
P22352	GPX3	FYTFLK	GPX3	409.73	413.73	2	508.31	516.33	1.00	y4	13.70	37.78+/-0.24
P17936	IBP3	YGQPLPGYTTK	IGFBP3	612.82	616.82	2	876.48	884.50	1.00	y8	20.00	29.26+/-0.19
P24593	IBP5	AVYLPNCDR	IGFBP5	554.27	559.27	2	661.27	671.28	1.00	y5	18.20	28.3+/-0.47
P19823	ITIH2	IQPSGGTNINEALLR	ITIH2	791.93	796.94	2	671.36	676.36	2.00	y13	25.50	35.16+/-0.23
P20142	PEPC	AECGLGVPTTR	PGC	580.79	585.79	2	630.36	640.37	1.00	y6	19.00	27.96+/-0.18
P04070	PROC	TFVLNFIK	PROC	491.29	495.30	2	733.46	741.47	1.00	y6	16.20	45.02+/-0.3
P07225	PROS	NNLELSTPLK	PROS1	564.82	568.82	2	787.46	795.47	1.00	y7	18.50	34.02+/-0.59

P52758	RIDA	AAYQVAALPK	RIDA	516.30	520.30	2	428.29	436.30	1.00	y4	17.00	31.05+/-0.43
P0DJI8	SAA1	FFGHGAEDSLADQAANEWGR	SAA1	726.66	730.00	3	732.34	742.35	1.00	y6	21.40	39.78+/-0.29
P35542	SAA4	GPGGVWAAK	SAA4	421.73	425.74	2	688.38	696.39	1.00	y7	14.10	26.25+/-0.19
P10646	TFPI1	IAYEEIFVK	TFPI	556.31	560.31	2	506.33	514.35	1.00	y4	18.20	39.75+/-0.55

BD vs SCZ				Mass Information								
Uniprot accession number	Protein	Peptide	Gene name	Precursor ion. Light (m/z)	Precursor ion. Heavy (m/z)	Precursor ion charge	Product ion. Light (m/z)	Product ion. Heavy (m/z)	Product ion charge	Product ion type	Collision energy (volt)	Retention time (RT)
P01011	AACT	DEELSCTVVELK	SERPINA3	474.57	477.24	3	488.31	496.32	1.00	y4	12.30	36.4+/-0.28
P15144	AMPN	AQIINDAFNLASAHK	ANPEP	734.40	739.40	2	277.15	277.15	1.00	b2	23.80	42.82+/-0.3
Q8TDL5	BPIB1	ALGFEAAESSLTK	BPIFB1	662.34	666.35	2	735.39	743.40	1.00	y7	21.50	37.2+/-0.24
Q9NZP8	C1RL	GSEAINAPGDNPAK	C1RL	670.83	674.83	2	698.35	706.36	1.00	y7	21.80	24.58+/-0.14
P00751	CFAB	DISEVVTPR	CFB	508.27	513.28	2	787.43	797.44	1.00	y7	16.80	30.12+/-0.2
O15551	CLD3	DFYNPVVPEAQK	CLDN3	703.85	707.86	2	572.30	580.32	1.00	y5	22.80	35.45+/-0.24
P09172	DOPO	VISTLEEPTPQCPTSQGR	DBH	559.83	563.83	2	509.30	513.31	2.00	y9	18.40	44.83+/-0.31
O15354	GPR37	ISPDLPDTIYVLALTYDSAR	GPR37	741.72	745.06	3	825.41	835.42	1.00	y7	21.90	52.74+/-0.15
P05546	HEP2	TLEAQLTPR	SERPIND1	514.79	519.79	2	814.44	824.45	1.00	y7	17.00	29.55+/-0.2
P24593	IBP5	AVYLPNCDR	IGFBP5	554.27	559.27	2	661.27	671.28	1.00	y5	18.20	28.3+/-0.47
P05155	IC1	TTFDPK	SERPING1	354.68	358.69	2	244.17	252.18	1.00	y2	12.00	22.55+/-0.76
P11226	MBL2	FQASVATPR	MBL2	488.76	493.77	2	701.39	711.40	1.00	y7	16.20	26.64+/-0.17
P61916	NPC2	LVVEWQLQDDK	NPC2	458.24	460.91	3	505.23	513.24	1.00	y4	11.70	38.92+/-0.52
O60486	PLXC1	LNTIGHYEISNGSTIK	PLXNC1	582.97	585.64	3	653.33	657.33	2.00	y12	16.20	33.21+/-0.51
Q99460	PSMD1	VSTAVLSITAK	PSMD1	545.33	549.34	2	731.47	739.48	1.00	y7	17.90	33.62+/-0.51
P10646	TFPI1	IAYEEIFVK	TFPI	556.31	560.31	2	506.33	514.35	1.00	y4	18.20	39.75+/-0.55
P07911	UROM	VLNLGPITR	UMOD	491.81	496.81	2	770.45	780.46	1.00	y7	16.20	35.59+/-0.25

† Uniprot accession number, protein entry, gene name, and information pertaining to mass spectra are listed for each proteomic feature (protein) in each pairwise comparison of groups.

Abbreviations

MDD: major depressive disorder, BD: bipolar disorder, SCZ: schizophrenia, MPM: multiprotein marker

Table S9. Alteration in expression level of proteins of each MPM model in the study population (171 SCZ, 170 BD, 174 MDD, and 160 HC)[†]

MDD vs BD	Uniprot accession number	Protein[‡]	Peptide	Gene name	MDD vs BD vs HC		MDD vs BD			MDD vs HC			BD vs HC		
					F-statistics	P-value[§] (ANOVA)	Log$_2$(Fold -change)	Expression pattern	P-value[§] (Tukey's HSD)	Log$_2$(Fold-change)	Expression pattern	P-value[§] (Tukey's HSD)	Log$_2$(Fold-change)	Expression pattern	P-value[§] (Tukey's HSD)
	P09972	ALDOC	ALQASALNAWR	ALDOC	4.286	**0.014**	-0.527	MDD upregulated	**0.043**	0.582	MDD upregulated	**0.024**	0.054	BD upregulated	0.968
	P15144	AMPN	AQIINDAFNLASAHK	ANPEP	3.597	**0.028**	-0.097	MDD upregulated	0.079	0.111	MDD upregulated	**0.040**	0.014	BD upregulated	0.949
	Q86TY3	ARMD4	TVVPSITR	ARMH4	4.882	**0.008**	0.225	BD upregulated	**0.011**	-0.192	MDD downregulated	**0.041**	0.033	BD upregulated	0.907
	Q9NZP8	C1RL	GSEAINAPGDNPAK	C1RL	8.097	**< 0.001**	0.107	BD upregulated	**0.015**	0.045	MDD upregulated	0.474	0.153	BD upregulated	**< 0.001**
	P55290	CAD13	INNTHALVSLLQNLNK	CDH13	1.992	0.137	0.166	BD upregulated	0.115	-0.092	MDD downregulated	0.525	0.075	BD upregulated	0.657
	P11597	CETP	ASYPDITGEK	CETP	6.723	**0.001**	-0.185	MDD upregulated	**0.002**	0.028	MDD upregulated	0.871	-0.158	BD downregulated	**0.013**
	Q03692	COAA1	GTHVWVGLYK	COL10A1	1.769	0.172	-0.347	MDD upregulated	0.146	0.156	MDD upregulated	0.682	-0.190	BD downregulated	0.571
	O60716	CTND1	GYELLFQPEVVR	CTNND1	5.810	**0.003**	0.285	BD upregulated	**0.006**	-0.019	MDD downregulated	0.979	0.266	BD upregulated	**0.014**
	Q08345	DDR1	LHLVALVGTQGR	DDR1	3.520	**0.030**	-0.316	MDD upregulated	**0.025**	0.107	MDD upregulated	0.658	-0.209	BD downregulated	0.210
	P09172	DOPO	TPEGLTLLFK	DBH	3.385	**0.035**	0.148	BD upregulated	0.246	-0.241	MDD downregulated	**0.028**	-0.093	BD downregulated	0.584
	P05155	IC1	TTFDPK	SERPING1	3.679	**0.026**	0.189	BD upregulated	**0.020**	-0.112	MDD downregulated	0.255	0.076	BD upregulated	0.535
	Q9NPH3	IL1AP	NEVWWTIDGK	IL1RAP	2.521	0.081	0.119	BD upregulated	0.090	-0.020	MDD downregulated	0.938	0.099	BD upregulated	0.198
	P19823	ITIH2	IQPSGGTNINEALLR	ITIH2	6.930	**0.001**	-0.133	MDD upregulated	**0.001**	0.038	MDD upregulated	0.559	-0.094	BD downregulated	**0.032**
	P61916	NPC2	LVVEWQLQDDK	NPC2	1.970	0.141	0.257	BD upregulated	0.117	-0.121	MDD downregulated	0.627	0.136	BD upregulated	0.560
	P62826	RAN	FNVWDTAGQEK	RAN	1.519	0.220	-0.185	MDD upregulated	0.197	0.067	MDD upregulated	0.811	-0.118	BD downregulated	0.530
	P0DJI8	SAA1	FFGHGAEDSLADQAANEWGR	SAA1	1.719	0.180	0.249	BD upregulated	0.159	-0.091	MDD downregulated	0.786	0.158	BD upregulated	0.490
	P02787	TRFE	ASYLDCIR	TF	2.993	0.051	0.302	BD upregulated	0.052	-0.093	MDD downregulated	0.749	0.209	BD upregulated	0.237

MDD vs SCZ	Uniprot accession number	Protein[‡]	Peptide	Gene name	MDD vs SCZ vs HC		MDD vs SCZ			MDD vs HC			SCZ vs HC		
					F-statistics	P-value[§] (ANOVA)	Log$_2$(Fold -change)	Expression pattern	P-value[§] (Tukey's HSD)	Log$_2$(Fold-change)	Expression pattern	P-value[§] (Tukey's HSD)	Log$_2$(Fold-change)	Expression pattern	P-value[§] (Tukey's HSD)
	P09972	ALDOC	ALQASALNAWR	ALDOC	4.981	**0.007**	-0.530	MDD upregulated	**0.001**	0.582	MDD upregulated	0.983	0.052	SCZ upregulated	**0.003**
	P35858	ALS	DFALQNPSAVPR	IGFALS	5.596	**0.004**	-0.133	MDD upregulated	0.272	-0.011	MDD downregulated	0.533	-0.144	SCZ downregulated	**0.028**
	P54802	ANAG	DFCGCHVAWSGSQLR	NAGLU	2.317	0.100	0.237	SCZ upregulated	0.119	-0.027	MDD downregulated	0.940	0.210	SCZ upregulated	0.065
	P05023	AT1A1	IVEIPFNSTNK	ATP1A1	7.890	**< 0.001**	-0.234	MDD upregulated	**< 0.001**	0.012	MDD upregulated	0.792	-0.222	SCZ downregulated	**0.004**
	P25774	CATS	YTELPYGR	CTSS	14.931	**< 0.001**	0.222	SCZ upregulated	0.004	0.161	MDD upregulated	0.058	0.383	SCZ upregulated	**< 0.001**
	P08185	CBG	HLVALSPK	SERPINA6	7.439	**0.001**	-0.155	MDD upregulated	**< 0.001**	-0.010	MDD downregulated	0.105	-0.165	SCZ downregulated	**< 0.001**
	Q96IY4	CBPB2	DTGTYGFLLPER	CPB2	3.367	**0.035**	0.070	SCZ upregulated	**0.002**	0.049	MDD upregulated	0.747	0.119	SCZ upregulated	**0.024**
	Q03692	COAA1	GTHVWVGLYK	COL10A1	1.941	0.145	-0.332	MDD upregulated	0.121	0.156	MDD upregulated	0.870	-0.176	SCZ downregulated	0.564
	Q14894	CRYM	TVVPVTK	CRYM	11.382	**< 0.001**	-0.233	MDD upregulated	**0.009**	0.008	MDD upregulated	0.958	-0.226	SCZ downregulated	**0.005**
	P22352	GPX3	FYTFLK	GPX3	18.516	**< 0.001**	-0.145	MDD upregulated	**0.006**	-0.075	MDD downregulated	0.260	-0.220	SCZ downregulated	0.314
	P17936	IBP3	YGQPLPGYTTK	IGFBP3	4.749	**0.009**	-0.149	MDD upregulated	**0.006**	0.077	MDD upregulated	0.260	-0.072	SCZ downregulated	0.314
	P24593	IBP5	AVYLPNCDR	IGFBP5	6.441	**0.002**	-0.292	MDD upregulated	**0.004**	0.063	MDD upregulated	0.058	-0.229	SCZ downregulated	**< 0.001**
	P19823	ITIH2	IQPSGGTNINEALLR	ITIH2	5.056	**0.007**	-0.115	MDD upregulated	**0.025**	0.038	MDD upregulated	**0.014**	-0.076	SCZ downregulated	0.966
	P20142	PEPC	AECGLGVPTTR	PGC	4.047	**0.018**	-0.141	MDD upregulated	0.024	0.018	MDD upregulated	0.940	-0.123	SCZ downregulated	0.065
	P04070	PROC	TFVLNFIK	PROC	9.017	**< 0.001**	-0.206	MDD upregulated	**< 0.001**	0.027	MDD upregulated	0.989	-0.179	SCZ downregulated	**< 0.001**
	P07225	PROS	NNLELSTPLK	PROS1	4.977	**0.007**	0.291	SCZ upregulated	**0.002**	0.075	MDD upregulated	0.977	0.366	SCZ upregulated	**0.002**

P52758	RIDA	AAYQVAALPK	RIDA	2.976	0.052	0.078	SCZ upregulated	0.063	-0.067	MDD downregulated	0.970	0.011	SCZ upregulated	0.948
P0DJI8	SAA1	FFGHGAEDSLADQAANEWGR	SAA1	8.692	**< 0.001**	0.539	SCZ upregulated	**0.044**	-0.091	MDD downregulated	0.813	0.447	SCZ upregulated	**0.009**
P35542	SAA4	GPGGVWAAK	SAA4	2.511	0.082	0.098	SCZ upregulated	0.119	0.011	MDD upregulated	0.973	0.110	SCZ upregulated	0.200
P10646	TFPI1	IAYEEIFVK	TFPI	6.287	**0.002**	-0.260	MDD upregulated	**0.005**	-0.025	MDD downregulated	0.562	-0.285	SCZ downregulated	0.103

BD vs SCZ	Uniprot accession number	Protein‡	Peptide	Gene name	BD vs SCZ vs HC		BD vs SCZ			BD vs HC			SCZ vs HC		
					F-statistics	P-value§ (ANOVA)	Log2(Fold -change)	Expression pattern	P-value§ (Tukey's HSD)	Log2(Fold-change)	Expression pattern	P-value§ (Tukey's HSD)	Log2(Fold-change)	Expression pattern	P-value§ (Tukey's HSD)
	P01011	AACT	DEELSCTVVELK	SERPINA3	3.695	**0.026**	0.274	SCZ upregulated	**0.021**	-0.091	BD downregulated	0.658	0.183	SCZ upregulated	0.186
	P15144	AMPN	AQIINDAFNLASAHK	ANPEP	4.238	**0.015**	0.105	SCZ upregulated	**0.048**	0.014	BD upregulated	0.948	0.119	SCZ upregulated	**0.023**
	Q8TDL5	BPIB1	ALGFEAAESSLTK	BPIFB1	3.677	**0.026**	0.246	SCZ upregulated	0.061	0.019	BD upregulated	0.984	0.265	SCZ upregulated	**0.043**
	Q9NZP8	C1RL	GSEAINAPGDNPAK	C1RL	9.289	**< 0.001**	-0.133	BD upregulated	**0.002**	0.153	BD upregulated	**< 0.001**	0.020	SCZ upregulated	0.868
	P00751	CFAB	DISEVVTPR	CFB	6.793	**0.001**	0.164	SCZ upregulated	**0.004**	-0.005	BD downregulated	0.995	0.159	SCZ upregulated	**0.006**
	O15551	CLD3	DFYNPVVPEAQK	CLDN3	8.529	**< 0.001**	-0.217	BD upregulated	**0.008**	-0.075	BD downregulated	0.571	-0.292	SCZ downregulated	**< 0.001**
	P09172	DOPO	VISTLEEPTPQCPTSQGR	DBH	8.002	**< 0.001**	-0.260	BD upregulated	**0.012**	-0.093	BD downregulated	0.570	-0.353	SCZ downregulated	**< 0.001**
	O15354	GPR37	ISPDLPDTIYVLALTYDSAR	GPR37	5.911	**0.003**	-0.557	BD upregulated	**0.002**	0.284	BD upregulated	0.196	-0.272	SCZ downregulated	0.223
	P05546	HEP2	TLEAQLTPR	SERPIND1	4.275	**0.014**	0.144	SCZ upregulated	**0.016**	-0.030	BD downregulated	0.834	0.114	SCZ upregulated	0.079
	P24593	IBP5	AVYLPNCDR	IGFBP5	11.290	**< 0.001**	-0.395	BD upregulated	**< 0.001**	0.166	BD upregulated	0.125	-0.229	SCZ downregulated	**0.020**
	P05155	IC1	TTFDPK	SERPING1	6.684	**0.001**	-0.241	BD upregulated	**0.001**	0.076	BD upregulated	0.505	-0.165	SCZ downregulated	**0.043**
	P11226	MBL2	FQASVATPR	MBL2	4.366	**0.013**	-0.326	BD upregulated	**0.015**	0.262	BD upregulated	0.070	-0.064	SCZ downregulated	0.853
	P61916	NPC2	LVVEWQLQDDK	NPC2	2.351	0.096	-0.287	BD upregulated	0.078	0.136	BD upregulated	0.571	-0.151	SCZ downregulated	0.500
	O60486	PLXC1	LNTIGHYEISNGSTIK	PLXNC1	1.580	0.207	0.275	SCZ upregulated	0.178	-0.132	BD downregulated	0.679	0.143	SCZ upregulated	0.634
	Q99460	PSMD1	VSTAVLSITAK	PSMD1	5.620	**0.004**	0.105	SCZ upregulated	**0.014**	-0.113	BD downregulated	**0.009**	-0.008	SCZ downregulated	0.975
	P10646	TFPI1	IAYEEIFVK	TFPI	6.455	**0.002**	-0.289	BD upregulated	**0.005**	0.004	BD upregulated	0.999	-0.285	SCZ downregulated	**0.007**
	P07911	UROM	VLNLGPITR	UMOD	5.754	**0.003**	-0.258	BD upregulated	**0.017**	-0.035	BD downregulated	0.929	-0.292	SCZ downregulated	**0.006**

† UniProt accession number, protein entry, gene name are listed for each proteomic feature (protein).

‡ Overlapping proteins between MPM models and their expression patterns are represented as different colors. Common proteins between MPM models for MDD vs BD and MDD vs SCZ (blue), between MPM models for MDD vs BD and BD vs SCZ (green), and between MPM models for MDD vs SCZ and BD vs SCZ (orange) are listed.

§ Statistically significant differences across the selected proteomic features (proteins) of each MPM model were analyzed by ANOVA. Post hoc analysis was performed by Tukey's HSD. Bold font denotes statistical significance at *P*-value < 0.05.

Abbreviations

MDD: major depressive disorder, BD: bipolar disorder, SCZ: schizophrenia, HC: healthy control, ANOVA: analysis of variation, HSD: honestly significant difference, MPM: multiprotein marker

Table S10. Clinical features used to develop symptom checklist-based (SCLB) models and clinician rater score-based (CRSB) models for each pairwise comparison of groups†

SCLB models

MDD vs BD
**Clinical variable**
SCL_SOM
SCL_PSY
SCL_DEP
SCL_60
SCL_IPS

MDD vs SCZ
**Clinical variable**
SCL_SOM
SCL_IPS
SCL_PSY
SCL_PAR
SCL_DEP

BD vs SCZ
**Clinical variable**
SCL_OCD
SCL_IPS
SCL_PSY
SCL_PHO
SCL_DEP
SCL_ANG

CRSB models

MDD vs BD
**Clinical variable**
BPRS
HAM-A
MADRS
YMRS

MDD vs SCZ
**Clinical variable**
BPRS
HAM-A
MADRS
YMRS

BD vs SCZ
**Clinical variable**
BPRS
HAM-A
MADRS
YMRS

† Clinical features used to develop clinical variable-based models in the training sets for each pairwise comparison of groups. For SCLB models, the dimensions of SCL in each pairwise comparison of groups were manually selected by investigating combinations that showed the highest discriminatory performance. In addition, the total scores of all clinician rater scales were used for CRSB models.

Abbreviations

SCL: Symptom Checklist-90-Revised
SCLB: symptom checklist-based
SOM: Somatization dimension
OCD: Obsessive-compulsive dimension
IPS: Interpersonal sensitivity dimension
DEP: Depression dimension
ANG: Hostility dimension
PHO: Phobic anxiety dimension
PAR: Paranoid ideation dimension
PSY: Psychoticism dimension
SCL_60: Overeating item

BPRS: Brief Psychiatric Rating Scale
HAM-A: Hamilton Anxiety Scale
MADRS: Montgomery-Asberg Depression Rating Scale
YMRS: Young Mania Rating Scale
CRSB: clinician rater score-based
MDD: major depressive disorder
BD: bipolar disorder
SCZ: schizophrenia

Table S11. List of predicted networks generated by IPA analysis†

ID	Molecules in Network	Total molecules	Network score	Focus molecules
**1**	**Akt,ALPL,ATP1A1,BPIFB1,C1RL,CDH13,CFB,CG,CTNND1,DBH,DDR1,ERK1/2,FSH,Growth hormone,Histone h3,IGFBP3,IGFBP5,IL1RAP,Lh,MAP4K4,MBL2,NFkB (complex),NPC2,P38 MAPK,PLIN3,PROC,PROS1,PSMD1,PTGS1,SAA1,SERPINA3,TCF,TCF21,TF,TYRO3**	**35**	**41**	**19**
**2**	**ANPEP,APLP2,APOB,CETP,CLDN3,COL10A1,Collagen type II,CPB2,CTSS,EHF,ETV5,F11,GLI1,GLI2,GPR37,HNF1A,HSPA1A/HSPA1B,IL1RN,ITIH2,KLK2,KLK6,LDL,LRP1,MMP2,MSI2,NOS3,NR5A2,PCSK9,PLIN2,SAA4,SERPIND1,SERPING1,STUB1,TFPI,VTN**	**35**	**23**	**12**
3	ALDOC,APC,BMP2,CCL3,CDK5,CTNNB1,CXCL1,EPAS1,ESR2,GPX3,GSK3B,HNRNPA1,IL13,IL22,IPO5,IRS1,JINK1/2,miR-483-3p (miRNAs w/seed CACUCCU),MTOR,MYB,MYOC,OGA,PARP,PARP1,PDIA3,PIK3R1,PKN1,PLXNC1,PPARG,RAN,RASSF1,RPTOR,STK11,SUZ12,TERT	35	6	4
4	PADI2,SERPINA6	2	2	1
5	CRYM,KDM1A,SBDS	3	2	1
6	AR,Hedgehog,PGC,SFTPB	4	2	1
7	GABARAP,GABARAPL1,GABARAPL2,NAGLU,TFEB	5	2	1

† Total 7 networks were generated. For each network, network score, the number of total molecules included in each corresponding network and focus molecules representing proteins of MPM models are presented. The networks satisfying network score≥20 are denoted by bold font.

Abbreviation

IPA: ingenuity pathway analysis

Table S12. List of all quantified proteins in the proteomic profiling of four pooled samples for each group (SCZ, BD, MDD, and HC)†

Identifiers				Information on identification												
Master	Accession	Description	Gene ID	Protein FDR Confidence: Combined	Contaminant	Exp. q-value: Combined	Sum PEP Score	Coverage [%]	# Peptides	# PSMs	# Unique Peptides	# Protein Groups	# AAs	MW [kDa]	calc. pI	Score Sequest HT
Master Protein	P31946	14-3-3 protein beta/alpha OS=Homo sapiens GN=YWHAB PE=1 SV=3	YWHAB	High	FALSE	0	28.879	28	6	114	3	1	246	28.1	4.83	232.25
Master Protein	P62258	14-3-3 protein epsilon OS=Homo sapiens GN=YWHAE PE=1 SV=1	YWHAE	High	FALSE	0	54.763	51	11	179	9	1	255	29.2	4.74	347.46
Master Protein	Q04917	14-3-3 protein eta OS=Homo sapiens GN=YWHAH PE=1 SV=4	YWHAH	High	FALSE	0	25.908	26	6	106	3	1	246	28.2	4.84	229.98
Master Protein	P61981	14-3-3 protein gamma OS=Homo sapiens GN=YWHAG PE=1 SV=2	YWHAG	High	FALSE	0	24.421	24	6	127	3	1	247	28.3	4.89	236.64
Master Protein	P63104	14-3-3 protein zeta/delta OS=Homo sapiens GN=YWHAZ PE=1 SV=1	YWHAZ	High	FALSE	0	84.17	69	14	590	12	1	245	27.7	4.79	1341.38
Master Protein	Q13200	26S proteasome non-ATPase regulatory subunit 2 OS=Homo sapiens GN=PSMD2 PE=1 SV=3	PSMD2	High	FALSE	0.001	7.259	2	2	2	2	1	908	100.1	5.2	3.8
Master Protein	P32754	4-hydroxyphenylpyruvate dioxygenase OS=Homo sapiens GN=HPD PE=1 SV=2	HPD	High	FALSE	0	16.058	13	5	17	5	1	393	44.9	7.01	39.82
Master Protein	P52209	6-phosphogluconate dehydrogenase, decarboxylating OS=Homo sapiens GN=PGD PE=1 SV=3	PGD	High	FALSE	0	18.569	12	6	41	6	1	483	53.1	7.23	74.34
Master Protein	O95336	6-phosphogluconolactonase OS=Homo sapiens GN=PGLS PE=1 SV=2	PGLS	High	FALSE	0	11.563	16	3	6	3	1	258	27.5	6.05	8.85
Master Protein	P08253	72 kDa type IV collagenase OS=Homo sapiens GN=MMP2 PE=1 SV=2	MMP2	High	FALSE	0	47.174	21	11	98	11	1	660	73.8	5.47	192.22
Master Protein	P11021	78 kDa glucose-regulated protein OS=Homo sapiens GN=HSPA5 PE=1 SV=2	HSPA5	High	FALSE	0	120.154	43	23	349	21	1	654	72.3	5.16	791.89
Master Protein	Q76LX8	A disintegrin and metalloproteinase with thrombospondin motifs 13 OS=Homo sapiens GN=ADAMTS13 PE=1 SV=1	ADAMTS13	High	FALSE	0	65.644	13	16	119	16	1	1427	153.5	7.17	226.95
Master Protein	P68032	Actin, alpha cardiac muscle 1 OS=Homo sapiens GN=ACTC1 PE=1 SV=1	ACTC1	High	FALSE	0	119.578	42	14	1613	4	1	377	42	5.39	3265.23
Master Protein	P60709	Actin, cytoplasmic 1 OS=Homo sapiens GN=ACTB PE=1 SV=1	ACTB	High	FALSE	0	227.008	65	20	2836	8	1	375	41.7	5.48	7393.08
Master Protein	O15143	Actin-related protein 2/3 complex subunit 1B OS=Homo sapiens GN=ARPC1B PE=1 SV=3	ARPC1B	High	FALSE	0	18.552	14	4	12	4	1	372	40.9	8.35	27.36
Master Protein	O15144	Actin-related protein 2/3 complex subunit 2 OS=Homo sapiens GN=ARPC2 PE=1 SV=1	ARPC2	High	FALSE	0	13.525	14	4	17	4	1	300	34.3	7.36	22.88
Master Protein	O15145	Actin-related protein 2/3 complex subunit 3 OS=Homo sapiens GN=ARPC3 PE=1 SV=3	ARPC3	High	FALSE	0	10.461	20	3	9	3	1	178	20.5	8.59	22.03
Master Protein	P61158	Actin-related protein 3 OS=Homo sapiens GN=ACTR3 PE=1 SV=3	ACTR3	High	FALSE	0	19.262	16	5	27	5	1	418	47.3	5.88	65.99
Master Protein	C9JIF9	Acylamino-acid-releasing enzyme OS=Homo sapiens GN=APEH PE=1 SV=1	APEH	High	FALSE	0.008	3.404	1	1	3	1	1	737	81.6	5.54	3.64
Master Protein	P07741	Adenine phosphoribosyltransferase OS=Homo sapiens GN=APRT PE=1 SV=2	APRT	High	FALSE	0.004	6.268	13	2	8	2	1	180	19.6	6.02	15.51
Master Protein	Q9NZK5	Adenosine deaminase CECR1 OS=Homo sapiens GN=CECR1 PE=1 SV=2	CECR1	High	FALSE	0	12.736	8	4	21	4	1	511	58.9	7.91	36.32
Master Protein	Q5T9B7	Adenylate kinase isoenzyme 1 OS=Homo sapiens GN=AK1 PE=1 SV=1	AK1	High	FALSE	0.004	6.259	12	2	4	2	1	210	23.4	8.6	3.32
Master Protein	Q01518	Adenylyl cyclase-associated protein 1 OS=Homo sapiens GN=CAP1 PE=1 SV=5	CAP1	High	FALSE	0	60.725	29	10	95	10	1	475	51.9	8.06	233.28
Master Protein	Q9HDC9	Adipocyte plasma membrane-associated protein OS=Homo sapiens GN=APMAP PE=1 SV=2	APMAP	High	FALSE	0	42.049	20	9	127	9	1	416	46.5	6.16	239.63
Master Protein	Q15848	Adiponectin OS=Homo sapiens GN=ADIPOQ PE=1 SV=1	ADIPOQ	High	FALSE	0	31.222	28	5	128	5	1	244	26.4	5.74	250.74
Master Protein	A6NC48	ADP-ribosyl cyclase/cyclic ADP-ribose hydrolase 2 OS=Homo sapiens GN=BST1 PE=4 SV=1	BST1	High	FALSE	0	13.593	8	3	25	3	1	333	37.5	7.97	51.87
Master Protein	P61204	ADP-ribosylation factor 3 OS=Homo sapiens GN=ARF3 PE=1 SV=2	ARF3	High	FALSE	0	26.253	30	4	45	2	1	181	20.6	7.43	88.98
Master Protein	P18085	ADP-ribosylation factor 4 OS=Homo sapiens GN=ARF4 PE=1 SV=3	ARF4	High	FALSE	0	19.678	23	3	24	1	1	180	20.5	7.14	42.91

Master Protein	P43652	Afamin OS=Homo sapiens GN=AFM PE=1 SV=1	AFM	High	FALSE	0	294.513	59	41	5291	41	1	599	69	5.9	13237.52
Master Protein	P24298	Alanine aminotransferase 1 OS=Homo sapiens GN=GPT PE=1 SV=3	GPT	High	FALSE	0	10.15	7	3	12	3	1	496	54.6	7.18	23.93
Master Protein	P00325	Alcohol dehydrogenase 1B OS=Homo sapiens GN=ADH1B PE=1 SV=2	ADH1B	High	FALSE	0	8.654	6	2	13	2	1	375	39.8	8.29	34.21
Master Protein	P11766	Alcohol dehydrogenase class-3 OS=Homo sapiens GN=ADH5 PE=1 SV=4	ADH5	High	FALSE	0.001	7.32	7	2	9	2	1	374	39.7	7.49	15.84
Master Protein	P02763	Alpha-1-acid glycoprotein 1 OS=Homo sapiens GN=ORM1 PE=1 SV=1	ORM1	High	FALSE	0	145.101	60	16	4816	11	1	201	23.5	5.02	13684.89
Master Protein	P19652	Alpha-1-acid glycoprotein 2 OS=Homo sapiens GN=ORM2 PE=1 SV=2	ORM2	High	FALSE	0	117.506	50	14	3816	9	1	201	23.6	5.11	9437.91
Master Protein	P01011	Alpha-1-antichymotrypsin OS=Homo sapiens GN=SERPINA3 PE=1 SV=2	SERPINA3	High	FALSE	0	300.693	62	28	20413	28	1	423	47.6	5.52	61617.56
Master Protein	P01009	Alpha-1-antitrypsin OS=Homo sapiens GN=SERPINA1 PE=1 SV=3	SERPINA1	High	FALSE	0	107.349	51	19	285	19	1	418	46.7	5.59	584.54
Master Protein	P04217	Alpha-1B-glycoprotein OS=Homo sapiens GN=A1BG PE=1 SV=4	A1BG	High	FALSE	0	175.609	51	20	6333	5	1	495	54.2	5.86	17145.07
Master Protein	C9JPV4	Alpha-2-antiplasmin (Fragment) OS=Homo sapiens GN=SERPINF2 PE=3 SV=1	SERPINF2	High	FALSE	0	106.54	51	13	1421	1	1	264	29.5	6.93	3439.93
Master Protein	P08697	Alpha-2-antiplasmin OS=Homo sapiens GN=SERPINF2 PE=1 SV=3	SERPINF2	High	FALSE	0	263.769	58	27	4493	15	1	491	54.5	6.29	10960.9
Master Protein	P02765	Alpha-2-HS-glycoprotein OS=Homo sapiens GN=AHSG PE=1 SV=1	AHSG	High	FALSE	0	184.27	62	18	8103	18	1	367	39.3	5.72	24543.75
Master Protein	P01023	Alpha-2-macroglobulin OS=Homo sapiens GN=A2M PE=1 SV=3	A2M	High	FALSE	0	1235.801	75	116	99449	105	1	1474	163.2	6.46	328592.31
Master Protein	O43707	Alpha-actinin-4 OS=Homo sapiens GN=ACTN4 PE=1 SV=2	ACTN4	High	FALSE	0	83.188	21	17	236	4	1	911	104.8	5.44	632.13
Master Protein	P04745	Alpha-amylase 1 OS=Homo sapiens GN=AMY1A PE=1 SV=2	AMY1A	High	FALSE	0	10.079	5	2	12	1	1	511	57.7	6.93	30.11
Master Protein	P06733	Alpha-enolase OS=Homo sapiens GN=ENO1 PE=1 SV=2	ENO1	High	FALSE	0	54.759	31	9	135	6	1	434	47.1	7.39	335.59
Master Protein	Q9NZD4	Alpha-hemoglobin-stabilizing protein OS=Homo sapiens GN=AHSP PE=1 SV=1	AHSP	High	FALSE	0.001	6.629	25	2	5	2	1	102	11.8	5	8.12
Master Protein	Q16706	Alpha-mannosidase 2 OS=Homo sapiens GN=MAN2A1 PE=1 SV=2	MAN2A1	High	FALSE	0	28.324	9	9	51	9	1	1144	131.1	7.58	83.38
Master Protein	P49641	Alpha-mannosidase 2x OS=Homo sapiens GN=MAN2A2 PE=2 SV=3	MAN2A2	High	FALSE	0	11.637	4	4	5	4	1	1150	130.5	6.84	11.59
Master Protein	P54802	Alpha-N-acetylglucosaminidase OS=Homo sapiens GN=NAGLU PE=1 SV=2	NAGLU	High	FALSE	0	41.274	17	9	37	9	1	743	82.2	6.65	88.37
Master Protein	P54920	Alpha-soluble NSF attachment protein OS=Homo sapiens GN=NAPA PE=1 SV=3	NAPA	High	FALSE	0.007	4.186	3	1	5	1	1	295	33.2	5.36	10.74
Master Protein	P15144	Aminopeptidase N OS=Homo sapiens GN=ANPEP PE=1 SV=4	ANPEP	High	FALSE	0	91.147	20	18	226	18	1	967	109.5	5.48	499.18
Master Protein	P05067	Amyloid beta A4 protein OS=Homo sapiens GN=APP PE=1 SV=3	APP	High	FALSE	0	15.377	7	4	5	4	1	770	86.9	4.82	10.21
Master Protein	P03950	Angiogenin OS=Homo sapiens GN=ANG PE=1 SV=1	ANG	High	FALSE	0	17.32	31	4	54	4	1	147	16.5	9.64	71.04
Master Protein	Q9Y5C1	Angiopoietin-related protein 3 OS=Homo sapiens GN=ANGPTL3 PE=1 SV=1	ANGPTL3	High	FALSE	0	20.333	13	6	47	6	1	460	53.6	6.7	91.55
Master Protein	Q8NI99	Angiopoietin-related protein 6 OS=Homo sapiens GN=ANGPTL6 PE=1 SV=1	ANGPTL6	High	FALSE	0	24.981	21	5	18	5	1	470	51.7	8.53	56.18
Master Protein	P12821	Angiotensin-converting enzyme OS=Homo sapiens GN=ACE PE=1 SV=1	ACE	High	FALSE	0	39.405	9	9	49	9	1	1306	149.6	6.39	81.39
Master Protein	P01019	Angiotensinogen OS=Homo sapiens GN=AGT PE=1 SV=1	AGT	High	FALSE	0	113.324	42	17	3866	17	1	485	53.1	6.32	10230.61
Master Protein	P04083	Annexin A1 OS=Homo sapiens GN=ANXA1 PE=1 SV=2	ANXA1	High	FALSE	0.007	4.173	3	1	2	1	1	346	38.7	7.02	6.31
Master Protein	P12429	Annexin A3 OS=Homo sapiens GN=ANXA3 PE=1 SV=3	ANXA3	High	FALSE	0.007	4.181	4	1	11	1	1	323	36.4	5.92	17.79
Master Protein	P08758	Annexin A5 OS=Homo sapiens GN=ANXA5 PE=1 SV=2	ANXA5	High	FALSE	0	12.235	14	3	7	3	1	320	35.9	5.05	14.93
Master Protein	Q9H6X2	Anthrax toxin receptor 1 OS=Homo sapiens GN=ANTXR1 PE=1 SV=2	ANTXR1	High	FALSE	0	10.794	5	3	21	3	1	564	62.7	7.61	24.75
Master Protein	P01008	Antithrombin-III OS=Homo sapiens GN=SERPINC1 PE=1 SV=1	SERPINC1	High	FALSE	0	400.923	65	40	19278	40	1	464	52.6	6.71	51345.6
Master Protein	P02647	Apolipoprotein A-I OS=Homo sapiens GN=APOA1 PE=1 SV=1	APOA1	High	FALSE	0	521.693	84	53	50521	53	1	267	30.8	5.76	137248.66
Master Protein	V9GYM3	Apolipoprotein A-II OS=Homo sapiens GN=APOA2 PE=1 SV=1	APOA2	High	FALSE	0	118.084	59	15	7520	15	1	133	14.9	8.27	22464.46
Master Protein	P06727	Apolipoprotein A-IV OS=Homo sapiens GN=APOA4 PE=1 SV=3	APOA4	High	FALSE	0	354.224	82	48	9759	47	1	396	45.4	5.38	26393.47
Master Protein	Q6Q788	Apolipoprotein A-V OS=Homo sapiens GN=APOA5 PE=1 SV=1	APOA5	High	FALSE	0	10.027	10	2	9	2	1	366	41.2	6.43	14.44
Master Protein	A0A087WTM7	Apolipoprotein B-100 OS=Homo sapiens GN=APOB PE=1 SV=1	APOB	High	FALSE	0	2841.424	69	335	86588	1	1	4344	489.5	7.15	235469.63

Master Protein	P04114	Apolipoprotein B-100 OS=Homo sapiens GN=APOB PE=1 SV=2	APOB	High	FALSE	0	2960.872	68	350	89663	16	1	4563	515.3	7.05	244871.51
Master Protein	P02654	Apolipoprotein C-I OS=Homo sapiens GN=APOC1 PE=1 SV=1	APOC1	High	FALSE	0	43.375	37	7	1048	7	1	83	9.3	8.47	2177.73
Master Protein	B0YIW2	Apolipoprotein C-III OS=Homo sapiens GN=APOC3 PE=1 SV=1	APOC3	High	FALSE	0	50.012	50	7	3495	7	1	117	12.8	8.18	9910.95
Master Protein	P55056	Apolipoprotein C-IV OS=Homo sapiens GN=APOC4 PE=1 SV=1	APOC4	High	FALSE	0	34.516	28	5	236	3	1	127	14.5	8.92	455.66
Master Protein	C9JF17	Apolipoprotein D (Fragment) OS=Homo sapiens GN=APOD PE=4 SV=1	APOD	High	FALSE	0	66.743	38	9	1736	9	1	215	24.1	5.6	4866.78
Master Protein	P02649	Apolipoprotein E OS=Homo sapiens GN=APOE PE=1 SV=1	APOE	High	FALSE	0	251.514	84	33	8169	33	1	317	36.1	5.73	22010.83
Master Protein	Q13790	Apolipoprotein F OS=Homo sapiens GN=APOF PE=1 SV=2	APOF	High	FALSE	0	19.948	15	4	541	4	1	326	35.4	5.64	1710.22
Master Protein	Q5SRP5	Apolipoprotein M OS=Homo sapiens GN=APOM PE=1 SV=1	APOM	High	FALSE	0	96.186	74	12	948	1	1	129	14.2	9.38	2044.04
Master Protein	O95445	Apolipoprotein M OS=Homo sapiens GN=APOM PE=1 SV=2	APOM	High	FALSE	0	97.086	70	13	981	2	1	188	21.2	6.01	2056.2
Master Protein	P08519	Apolipoprotein(a) OS=Homo sapiens GN=LPA PE=1 SV=1	LPA	High	FALSE	0	109.299	41	22	325	22	1	4548	501	5.88	883.1
Master Protein	P17174	Aspartate aminotransferase, cytoplasmic OS=Homo sapiens GN=GOT1 PE=1 SV=3	GOT1	High	FALSE	0	23.609	16	5	19	5	1	413	46.2	7.01	36.62
Master Protein	P14868	Aspartate--tRNA ligase, cytoplasmic OS=Homo sapiens GN=DARS PE=1 SV=2	DARS	High	FALSE	0.004	6.07	4	2	4	2	1	501	57.1	6.55	4.54
Master Protein	P53396	ATP-citrate synthase OS=Homo sapiens GN=ACLY PE=1 SV=3	ACLY	High	FALSE	0	8.021	3	3	5	3	1	1101	120.8	7.33	2.01
Master Protein	O75882	Attractin OS=Homo sapiens GN=ATRN PE=1 SV=2	ATRN	High	FALSE	0	124.392	19	22	1216	22	1	1429	158.4	7.31	2866.97
Master Protein	P98160	Basement membrane-specific heparan sulfate proteoglycan core protein OS=Homo sapiens GN=HSPG2 PE=1 SV=4	HSPG2	High	FALSE	0	93.106	6	22	187	22	1	4391	468.5	6.51	430.29
Master Protein	P15291	Beta-1,4-galactosyltransferase 1 OS=Homo sapiens GN=B4GALT1 PE=1 SV=5	B4GALT1	High	FALSE	0.001	6.923	5	2	5	2	1	398	43.9	8.65	8.7
Master Protein	P02749	Beta-2-glycoprotein 1 OS=Homo sapiens GN=APOH PE=1 SV=3	APOH	High	FALSE	0	164.234	61	19	4646	19	1	345	38.3	7.97	13343.19
Master Protein	H0YL18	Beta-2-microglobulin form pI 5.3 OS=Homo sapiens GN=B2M PE=1 SV=1	B2M	High	FALSE	0	14.162	16	2	71	2	1	122	14	7.44	194.5
Master Protein	Q562R1	Beta-actin-like protein 2 OS=Homo sapiens GN=ACTBL2 PE=1 SV=2	ACTBL2	High	FALSE	0	56.32	22	7	951	3	1	376	42	5.59	1677.74
Master Protein	Q96KN2	Beta-Ala-His dipeptidase OS=Homo sapiens GN=CNDP1 PE=1 SV=4	CNDP1	High	FALSE	0	117.637	36	16	916	1	1	507	56.7	5.3	2355.04
Master Protein	J3KRP0	Beta-Ala-His dipeptidase OS=Homo sapiens GN=CNDP1 PE=4 SV=2	CNDP1	High	FALSE	0	126.724	43	16	1027	1	1	464	51.9	5.4	2559.05
Master Protein	P13929	Beta-enolase OS=Homo sapiens GN=ENO3 PE=1 SV=5	ENO3	High	FALSE	0	25.1	15	4	48	1	1	434	47	7.71	148.69
Master Protein	O00462	Beta-mannosidase OS=Homo sapiens GN=MANBA PE=1 SV=3	MANBA	High	FALSE	0	12.835	5	3	5	3	1	879	100.8	5.52	13.61
Master Protein	A0A087WUL0	Bifunctional ATP-dependent dihydroxyacetone kinase/FAD-AMP lyase (cyclizing) OS=Homo sapiens GN=DAK PE=1 SV=1	DAK	High	FALSE	0	8.657	6	2	8	2	1	576	59	7.49	10.63
Master Protein	P31939	Bifunctional purine biosynthesis protein PURH OS=Homo sapiens GN=ATIC PE=1 SV=3	ATIC	High	FALSE	0	12.436	7	3	6	3	1	592	64.6	6.71	15.72
Master Protein	P07738	Bisphosphoglycerate mutase OS=Homo sapiens GN=BPGM PE=1 SV=2	BPGM	High	FALSE	0	11.311	15	3	22	3	1	259	30	6.54	43.06
Master Protein	Q13867	Bleomycin hydrolase OS=Homo sapiens GN=BLMH PE=1 SV=1	BLMH	High	FALSE	0.005	4.53	2	1	14	1	1	455	52.5	6.27	28.62
Master Protein	P13727	Bone marrow proteoglycan OS=Homo sapiens GN=PRG2 PE=1 SV=2	PRG2	High	FALSE	0	21.971	23	4	37	4	1	222	25.2	6.76	85.64
Master Protein	P13497	Bone morphogenetic protein 1 OS=Homo sapiens GN=BMP1 PE=1 SV=2	BMP1	High	FALSE	0	7.68	3	3	4	3	1	986	111.2	6.9	4.11
Master Protein	Q9NP55	BPI fold-containing family A member 1 OS=Homo sapiens GN=BPIFA1 PE=1 SV=1	BPIFA1	High	FALSE	0.001	7.479	9	2	3	2	1	256	26.7	5.76	4.17
Master Protein	Q8TDL5	BPI fold-containing family B member 1 OS=Homo sapiens GN=BPIFB1 PE=1 SV=1	BPIFB1	High	FALSE	0	24.035	15	6	19	6	1	484	52.4	7.23	33.98
Master Protein	P80723	Brain acid soluble protein 1 OS=Homo sapiens GN=BASP1 PE=1 SV=2	BASP1	High	FALSE	0	33.019	51	6	54	6	1	227	22.7	4.63	130.31
Master Protein	A0A087X188	Bridging integrator 2 OS=Homo sapiens GN=BIN2 PE=1 SV=1	BIN2	High	FALSE	0	11.433	7	3	8	3	1	597	65	5.38	18.13
Master Protein	F5H2F4	C-1-tetrahydrofolate synthase, cytoplasmic OS=Homo sapiens GN=MTHFD1 PE=1 SV=1	MTHFD1	High	FALSE	0.001	6.88	2	2	5	2	1	1020	110.5	8.43	4.28
Master Protein	P04003	C4b-binding protein alpha chain OS=Homo sapiens GN=C4BPA PE=1 SV=2	C4BPA	High	FALSE	0	209.222	51	25	5059	25	1	597	67	7.3	13377.84

Master Protein	P20851	C4b-binding protein beta chain OS=Homo sapiens GN=C4BPB PE=1 SV=1	C4BPB	High	FALSE	0	51.938	40	7	387	7	1	252	28.3	5.14	745.88
Master Protein	A0A087WXI5	Cadherin-1 OS=Homo sapiens GN=CDH1 PE=1 SV=1	CDH1	High	FALSE	0	15.777	6	4	18	4	1	903	100	4.79	23.49
Master Protein	P33151	Cadherin-5 OS=Homo sapiens GN=CDH5 PE=1 SV=5	CDH5	High	FALSE	0	47.989	14	9	172	9	1	784	87.5	5.43	457.5
Master Protein	P55285	Cadherin-6 OS=Homo sapiens GN=CDH6 PE=1 SV=1	CDH6	High	FALSE	0	11.753	5	3	12	3	1	790	88.3	4.93	26.3
Master Protein	Q9BYE9	Cadherin-related family member 2 OS=Homo sapiens GN=CDHR2 PE=1 SV=2	CDHR2	High	FALSE	0.001	6.741	2	2	5	2	1	1310	141.5	4.5	10.37
Master Protein	Q9HBB8	Cadherin-related family member 5 OS=Homo sapiens GN=CDHR5 PE=1 SV=3	CDHR5	High	FALSE	0.001	6.392	3	2	3	2	1	845	88.2	4.93	2.66
Master Protein	Q05682	Caldesmon OS=Homo sapiens GN=CALD1 PE=1 SV=3	CALD1	High	FALSE	0	15.663	6	4	19	4	1	793	93.2	5.66	45.18
Master Protein	E7EMB3	Calmodulin OS=Homo sapiens GN=CALM2 PE=1 SV=1	CALM2	High	FALSE	0	8.271	13	2	7	2	1	196	21.7	4.56	20.2
Master Protein	K7ELJ7	Calpain small subunit 1 OS=Homo sapiens GN=CAPNS1 PE=1 SV=1	CAPNS1	High	FALSE	0	10.196	14	4	19	4	1	278	29.4	5.31	14.59
Master Protein	P07384	Calpain-1 catalytic subunit OS=Homo sapiens GN=CAPN1 PE=1 SV=1	CAPN1	High	FALSE	0	19.912	9	6	18	6	1	714	81.8	5.67	36.96
Master Protein	B4DUT8	Calponin OS=Homo sapiens GN=CNN2 PE=1 SV=1	CNN2	High	FALSE	0	11.895	12	3	13	3	1	330	35.9	7.3	26.99
Master Protein	P27797	Calreticulin OS=Homo sapiens GN=CALR PE=1 SV=1	CALR	High	FALSE	0	35.73	21	5	34	5	1	417	48.1	4.44	67.28
Master Protein	O94985	Calsyntenin-1 OS=Homo sapiens GN=CLSTN1 PE=1 SV=1	CLSTN1	High	FALSE	0	8.979	3	3	13	3	1	981	109.7	4.91	16.44
Master Protein	P10644	cAMP-dependent protein kinase type I-alpha regulatory subunit OS=Homo sapiens GN=PRKAR1A PE=1 SV=1	PRKAR1A	High	FALSE	0.001	7.296	7	2	8	2	1	381	43	5.35	7.78
Master Protein	B1AK88	Capping protein (Actin filament) muscle Z-line, beta, isoform CRA_d OS=Homo sapiens GN=CAPZB PE=1 SV=1	CAPZB	High	FALSE	0.004	5.31	5	2	10	2	1	301	33.8	6.43	22.79
Master Protein	P00915	Carbonic anhydrase 1 OS=Homo sapiens GN=CA1 PE=1 SV=2	CA1	High	FALSE	0	73.955	40	9	662	9	1	261	28.9	7.12	1814.07
Master Protein	P00918	Carbonic anhydrase 2 OS=Homo sapiens GN=CA2 PE=1 SV=2	CA2	High	FALSE	0	41.212	40	8	203	8	1	260	29.2	7.4	469.19
Master Protein	P16152	Carbonyl reductase [NADPH] 1 OS=Homo sapiens GN=CBR1 PE=1 SV=3	CBR1	High	FALSE	0.001	6.996	11	2	4	2	1	277	30.4	8.32	6.52
Master Protein	Q96IY4	Carboxypeptidase B2 OS=Homo sapiens GN=CPB2 PE=1 SV=2	CPB2	High	FALSE	0	66.282	29	11	477	11	1	423	48.4	7.71	1248.92
Master Protein	P15169	Carboxypeptidase N catalytic chain OS=Homo sapiens GN=CPN1 PE=1 SV=1	CPN1	High	FALSE	0	83.941	30	11	810	11	1	458	52.3	7.34	2072.71
Master Protein	P22792	Carboxypeptidase N subunit 2 OS=Homo sapiens GN=CPN2 PE=1 SV=3	CPN2	High	FALSE	0	78.884	26	12	1301	12	1	545	60.5	5.99	3256.14
Master Protein	A0A087WYY1	Carboxypeptidase Q OS=Homo sapiens GN=CPQ PE=4 SV=1	CPQ	High	FALSE	0	18.086	11	5	27	5	1	540	59.7	7.93	68.53
Master Protein	Q9NQ79	Cartilage acidic protein 1 OS=Homo sapiens GN=CRTAC1 PE=1 SV=2	CRTAC1	High	FALSE	0	68.154	25	12	217	12	1	661	71.4	5.12	576.75
Master Protein	P49747	Cartilage oligomeric matrix protein OS=Homo sapiens GN=COMP PE=1 SV=2	COMP	High	FALSE	0	59.955	20	10	156	8	1	757	82.8	4.6	404.82
Master Protein	P42574	Caspase-3 OS=Homo sapiens GN=CASP3 PE=1 SV=2	CASP3	High	FALSE	0.007	3.85	4	1	3	1	1	277	31.6	6.54	7.75
Master Protein	P04040	Catalase OS=Homo sapiens GN=CAT PE=1 SV=3	CAT	High	FALSE	0	74.816	35	14	190	14	1	527	59.7	7.39	510.6
Master Protein	J3KNB4	Cathelicidin antimicrobial peptide OS=Homo sapiens GN=CAMP PE=4 SV=1	CAMP	High	FALSE	0	37.861	36	8	78	8	1	173	19.6	9.41	156.91
Master Protein	P07858	Cathepsin B OS=Homo sapiens GN=CTSB PE=1 SV=3	CTSB	High	FALSE	0	8.358	9	2	2	2	1	339	37.8	6.3	5.1
Master Protein	P07339	Cathepsin D OS=Homo sapiens GN=CTSD PE=1 SV=1	CTSD	High	FALSE	0	50.565	30	9	64	9	1	412	44.5	6.54	138.22
Master Protein	Q9UBX1	Cathepsin F OS=Homo sapiens GN=CTSF PE=1 SV=1	CTSF	High	FALSE	0.001	7.143	6	2	7	2	1	484	53.3	8.22	17.78
Master Protein	P25774	Cathepsin S OS=Homo sapiens GN=CTSS PE=1 SV=3	CTSS	High	FALSE	0.004	5.694	6	2	9	2	1	331	37.5	8.34	9.13
Master Protein	Q9UBR2	Cathepsin Z OS=Homo sapiens GN=CTSZ PE=1 SV=1	CTSZ	High	FALSE	0.004	5.571	6	2	6	2	1	303	33.8	7.11	10.63
Master Protein	P11717	Cation-independent mannose-6-phosphate receptor OS=Homo sapiens GN=IGF2R PE=1 SV=3	IGF2R	High	FALSE	0	41.782	6	11	40	11	1	2491	274.2	5.94	75.22
Master Protein	Q6YHK3	CD109 antigen OS=Homo sapiens GN=CD109 PE=1 SV=2	CD109	High	FALSE	0	45.937	10	11	100	11	1	1445	161.6	5.85	242.48
Master Protein	Q13740	CD166 antigen OS=Homo sapiens GN=ALCAM PE=1 SV=2	ALCAM	High	FALSE	0	21.721	11	5	29	5	1	583	65.1	6.25	58.97
Master Protein	P16070	CD44 antigen OS=Homo sapiens GN=CD44 PE=1 SV=3	CD44	High	FALSE	0	8.168	3	2	16	2	1	742	81.5	5.33	35.6
Master Protein	O43866	CD5 antigen-like OS=Homo sapiens GN=CD5L PE=1 SV=1	CD5L	High	FALSE	0	59.938	40	10	251	10	1	347	38.1	5.47	571.59

Master Protein	P60953	Cell division control protein 42 homolog OS=Homo sapiens GN=CDC42 PE=1 SV=2	CDC42	High	FALSE	0	8.396	9	1	4	1	1	191	21.2	6.55	9.05
Master Protein	P43121	Cell surface glycoprotein MUC18 OS=Homo sapiens GN=MCAM PE=1 SV=2	MCAM	High	FALSE	0	35.04	14	8	97	8	1	646	71.6	5.76	250.86
Master Protein	H7C5N5	Ceruloplasmin (Fragment) OS=Homo sapiens GN=CP PE=4 SV=1	CP	High	FALSE	0	65.046	36	7	1371	1	1	225	26.1	5.94	3575.73
Master Protein	D6RE86	Ceruloplasmin (Fragment) OS=Homo sapiens GN=CP PE=4 SV=3	CP	High	FALSE	0	37.154	41	6	207	1	1	179	20.9	7.24	517.57
Master Protein	P00450	Ceruloplasmin OS=Homo sapiens GN=CP PE=1 SV=1	CP	High	FALSE	0	615.078	62	66	15425	55	1	1065	122.1	5.72	47213.14
Master Protein	P36222	Chitinase-3-like protein 1 OS=Homo sapiens GN=CHI3L1 PE=1 SV=2	CHI3L1	High	FALSE	0	15.222	14	4	13	4	1	383	42.6	8.46	22.73
Master Protein	O00299	Chloride intracellular channel protein 1 OS=Homo sapiens GN=CLIC1 PE=1 SV=4	CLIC1	High	FALSE	0	48.412	60	11	115	11	1	241	26.9	5.17	228.32
Master Protein	P11597	Cholesteryl ester transfer protein OS=Homo sapiens GN=CETP PE=1 SV=2	CETP	High	FALSE	0	69.017	30	12	189	12	1	493	54.7	6.09	426.47
Master Protein	P06276	Cholinesterase OS=Homo sapiens GN=BCHE PE=1 SV=1	BCHE	High	FALSE	0	88.959	25	15	658	15	1	602	68.4	7.42	1584.53
Master Protein	Q6UVK1	Chondroitin sulfate proteoglycan 4 OS=Homo sapiens GN=CSPG4 PE=1 SV=2	CSPG4	High	FALSE	0.006	3.482	1	1	1	1	1	2322	250.4	5.47	2.06
Master Protein	P26992	Ciliary neurotrophic factor receptor subunit alpha OS=Homo sapiens GN=CNTFR PE=1 SV=2	CNTFR	High	FALSE	0	8.881	3	1	2	1	1	372	40.6	6.76	7.07
Master Protein	Q14019	Coactosin-like protein OS=Homo sapiens GN=COTL1 PE=1 SV=3	COTL1	High	FALSE	0	12.583	35	4	49	4	1	142	15.9	5.67	84.51
Master Protein	P00740	Coagulation factor IX OS=Homo sapiens GN=F9 PE=1 SV=2	F9	High	FALSE	0	87.446	26	11	379	11	1	461	51.7	5.47	1038.74
Master Protein	P12259	Coagulation factor V OS=Homo sapiens GN=F5 PE=1 SV=4	F5	High	FALSE	0	299.788	26	51	1465	51	1	2224	251.5	6.05	3587.34
Master Protein	P08709	Coagulation factor VII OS=Homo sapiens GN=F7 PE=1 SV=1	F7	High	FALSE	0	34.64	17	5	75	5	1	466	51.6	7.23	227.87
Master Protein	P00451	Coagulation factor VIII OS=Homo sapiens GN=F8 PE=1 SV=1	F8	High	FALSE	0	12.656	2	3	17	3	1	2351	266.8	7.36	21.06
Master Protein	P00742	Coagulation factor X OS=Homo sapiens GN=F10 PE=1 SV=2	F10	High	FALSE	0	89.389	28	13	635	13	1	488	54.7	5.94	1777.61
Master Protein	P03951	Coagulation factor XI OS=Homo sapiens GN=F11 PE=1 SV=1	F11	High	FALSE	0	116.414	41	22	745	22	1	625	70.1	8.1	1676.08
Master Protein	P00748	Coagulation factor XII OS=Homo sapiens GN=F12 PE=1 SV=3	F12	High	FALSE	0	97.555	33	16	922	16	1	615	67.7	7.74	2473.49
Master Protein	P00488	Coagulation factor XIII A chain OS=Homo sapiens GN=F13A1 PE=1 SV=4	F13A1	High	FALSE	0	152.667	26	19	1122	19	1	732	83.2	6.09	2737.12
Master Protein	P05160	Coagulation factor XIII B chain OS=Homo sapiens GN=F13B PE=1 SV=3	F13B	High	FALSE	0	122.709	40	22	663	22	1	661	75.5	6.39	1584.72
Master Protein	E9PK25	Cofilin-1 OS=Homo sapiens GN=CFL1 PE=1 SV=1	CFL1	High	FALSE	0	23.219	26	4	153	4	1	204	22.7	8.34	306.27
Master Protein	P12109	Collagen alpha-1(VI) chain OS=Homo sapiens GN=COL6A1 PE=1 SV=3	COL6A1	High	FALSE	0	20.486	6	4	30	4	1	1028	108.5	5.43	59.13
Master Protein	P39059	Collagen alpha-1(XV) chain OS=Homo sapiens GN=COL15A1 PE=1 SV=2	COL15A1	High	FALSE	0	8.473	2	2	10	2	1	1388	141.6	5	21.01
Master Protein	P39060	Collagen alpha-1(XVIII) chain OS=Homo sapiens GN=COL18A1 PE=1 SV=5	COL18A1	High	FALSE	0	22.505	5	7	26	7	1	1754	178.1	6.01	64.04
Master Protein	P12111	Collagen alpha-3(VI) chain OS=Homo sapiens GN=COL6A3 PE=1 SV=5	COL6A3	High	FALSE	0	151.654	14	37	256	37	1	3177	343.5	6.68	635.67
Master Protein	Q9Y6Z7	Collectin-10 OS=Homo sapiens GN=COLEC10 PE=2 SV=2	COLEC10	High	FALSE	0	13.218	9	2	37	2	1	277	30.7	7.33	103.16
Master Protein	P02745	Complement C1q subcomponent subunit A OS=Homo sapiens GN=C1QA PE=1 SV=2	C1QA	High	FALSE	0	27.542	29	6	237	6	1	245	26	9.11	565.2
Master Protein	P02746	Complement C1q subcomponent subunit B OS=Homo sapiens GN=C1QB PE=1 SV=3	C1QB	High	FALSE	0	74.992	46	10	1966	10	1	253	26.7	8.63	4553.31
Master Protein	P02747	Complement C1q subcomponent subunit C OS=Homo sapiens GN=C1QC PE=1 SV=3	C1QC	High	FALSE	0	44.925	27	6	2287	6	1	245	25.8	8.41	6494.06
Master Protein	B4DPQ0	Complement C1r subcomponent OS=Homo sapiens GN=C1R PE=2 SV=1	C1R	High	FALSE	0	190.539	48	28	2110	26	1	719	81.8	6.37	5710.7
Master Protein	F5H6S5	Complement C1r subcomponent-like protein (Fragment) OS=Homo sapiens GN=C1RL PE=4 SV=3	C1RL	High	FALSE	0	22.854	13	3	80	2	1	192	21	7.65	213.42
Master Protein	Q9NZP8	Complement C1r subcomponent-like protein OS=Homo sapiens GN=C1RL PE=1 SV=2	C1RL	High	FALSE	0	41.09	24	9	459	6	1	487	53.5	7.2	1159.09
Master Protein	C9IZP8	Complement C1s subcomponent (Fragment) OS=Homo sapiens GN=C1S PE=4 SV=1	C1S	High	FALSE	0.005	4.76	17	1	40	1	1	103	11.8	4.2	129.4

Master Protein	P09871	Complement C1s subcomponent OS=Homo sapiens GN=C1S PE=1 SV=1	C1S	High	FALSE	0	212.807	44	24	3966	23	1	688	76.6	4.96	9042.12
Master Protein	P06681	Complement C2 OS=Homo sapiens GN=C2 PE=1 SV=2	C2	High	FALSE	0	223.581	42	28	2149	6	1	752	83.2	7.42	5502.29
Master Protein	P01024	Complement C3 OS=Homo sapiens GN=C3 PE=1 SV=2	C3	High	FALSE	0	1728.917	89	170	102493	170	1	1663	187	6.4	310815.88
Master Protein	P0C0L4	Complement C4-A OS=Homo sapiens GN=C4A PE=1 SV=2	C4A	High	FALSE	0	979.886	68	119	32243	5	1	1744	192.7	7.08	89968.47
Master Protein	P0C0L5	Complement C4-B OS=Homo sapiens GN=C4B PE=1 SV=2	C4B	High	FALSE	0	993.593	68	121	32939	7	1	1744	192.6	7.27	93796.38
Master Protein	P01031	Complement C5 OS=Homo sapiens GN=C5 PE=1 SV=4	C5	High	FALSE	0	583.391	50	73	12339	73	1	1676	188.2	6.52	29892.62
Master Protein	Q9NPY3	Complement component C1q receptor OS=Homo sapiens GN=CD93 PE=1 SV=3	CD93	High	FALSE	0.001	6.934	6	3	17	3	1	652	68.5	5.44	31.09
Master Protein	P13671	Complement component C6 OS=Homo sapiens GN=C6 PE=1 SV=3	C6	High	FALSE	0	214.667	42	35	2232	35	1	934	104.7	6.76	6513.04
Master Protein	P10643	Complement component C7 OS=Homo sapiens GN=C7 PE=1 SV=2	C7	High	FALSE	0	289.23	53	34	3511	33	1	843	93.5	6.48	9647.41
Master Protein	P07357	Complement component C8 alpha chain OS=Homo sapiens GN=C8A PE=1 SV=2	C8A	High	FALSE	0	157.959	43	21	2212	21	1	584	65.1	6.47	5828.9
Master Protein	P07358	Complement component C8 beta chain OS=Homo sapiens GN=C8B PE=1 SV=3	C8B	High	FALSE	0	218.481	47	23	3594	23	1	591	67	8.13	8376.54
Master Protein	P07360	Complement component C8 gamma chain OS=Homo sapiens GN=C8G PE=1 SV=3	C8G	High	FALSE	0	97.506	60	11	1542	11	1	202	22.3	8.31	4929.47
Master Protein	P02748	Complement component C9 OS=Homo sapiens GN=C9 PE=1 SV=2	C9	High	FALSE	0	134.31	42	21	3157	21	1	559	63.1	5.59	8246.12
Master Protein	K7ERG9	Complement factor D OS=Homo sapiens GN=CFD PE=3 SV=1	CFD	High	FALSE	0	81.75	50	10	138	10	1	260	27.8	7.25	451.51
Master Protein	P08603	Complement factor H OS=Homo sapiens GN=CFH PE=1 SV=4	CFH	High	FALSE	0	581.524	53	70	14622	62	1	1231	139	6.61	38144.01
Master Protein	Q03591	Complement factor H-related protein 1 OS=Homo sapiens GN=CFHR1 PE=1 SV=2	CFHR1	High	FALSE	0	99.846	45	16	1965	3	1	330	37.6	7.39	4891
Master Protein	P36980	Complement factor H-related protein 2 OS=Homo sapiens GN=CFHR2 PE=1 SV=1	CFHR2	High	FALSE	0	53.175	39	8	414	4	1	270	30.6	6.38	1036.87
Master Protein	Q02985	Complement factor H-related protein 3 OS=Homo sapiens GN=CFHR3 PE=1 SV=2	CFHR3	High	FALSE	0	19.206	8	3	589	2	1	330	37.3	7.55	1694.23
Master Protein	Q9BXR6	Complement factor H-related protein 5 OS=Homo sapiens GN=CFHR5 PE=1 SV=1	CFHR5	High	FALSE	0	61.564	25	13	197	11	1	569	64.4	7.06	481
Master Protein	E7ETH0	Complement factor I light chain OS=Homo sapiens GN=CFI PE=3 SV=1	CFI	High	FALSE	0	132.336	31	18	2300	18	1	591	66.6	7.59	5877.78
Master Protein	P17927	Complement receptor type 1 OS=Homo sapiens GN=CR1 PE=1 SV=3	CR1	High	FALSE	0.01	3.219	1	1	1	1	1	2039	223.5	6.95	1.67
Master Protein	Q12860	Contactin-1 OS=Homo sapiens GN=CNTN1 PE=1 SV=1	CNTN1	High	FALSE	0	49.46	15	12	96	12	1	1018	113.2	5.9	219.57
Master Protein	Q9P232	Contactin-3 OS=Homo sapiens GN=CNTN3 PE=1 SV=3	CNTN3	High	FALSE	0	10.806	3	3	20	3	1	1028	112.8	6.3	46.73
Master Protein	Q8IWV2	Contactin-4 OS=Homo sapiens GN=CNTN4 PE=1 SV=1	CNTN4	High	FALSE	0	19.782	6	6	19	6	1	1026	113.4	7.47	41.13
Master Protein	P31146	Coronin-1A OS=Homo sapiens GN=CORO1A PE=1 SV=4	CORO1A	High	FALSE	0	31.075	17	7	97	7	1	461	51	6.68	193.94
Master Protein	Q9BR76	Coronin-1B OS=Homo sapiens GN=CORO1B PE=1 SV=1	CORO1B	High	FALSE	0.006	3.995	2	1	9	1	1	489	54.2	5.88	22.89
Master Protein	P08185	Corticosteroid-binding globulin OS=Homo sapiens GN=SERPINA6 PE=1 SV=1	SERPINA6	High	FALSE	0	79.495	39	11	2312	11	1	405	45.1	6.04	6205.07
Master Protein	P24387	Corticotropin-releasing factor-binding protein OS=Homo sapiens GN=CRHBP PE=1 SV=2	CRHBP	High	FALSE	0.009	3.256	3	1	12	1	1	322	36.1	6.52	22.19
Master Protein	Q9P1F3	Costars family protein ABRACL OS=Homo sapiens GN=ABRACL PE=1 SV=1	ABRACL	High	FALSE	0.007	3.776	16	1	2	1	1	81	9.1	6.29	4.81
Master Protein	P02741	C-reactive protein OS=Homo sapiens GN=CRP PE=1 SV=1	CRP	High	FALSE	0	17.84	22	5	334	5	1	224	25	5.63	816.24
Master Protein	P06732	Creatine kinase M-type OS=Homo sapiens GN=CKM PE=1 SV=2	CKM	High	FALSE	0	65.645	31	10	139	10	1	381	43.1	7.25	359.43
Master Protein	Q9UBG0	C-type mannose receptor 2 OS=Homo sapiens GN=MRC2 PE=1 SV=2	MRC2	High	FALSE	0	8.437	2	2	12	2	1	1479	166.6	5.83	25.01
Master Protein	Q86VP6	Cullin-associated NEDD8-dissociated protein 1 OS=Homo sapiens GN=CAND1 PE=1 SV=2	CAND1	High	FALSE	0	21.252	5	5	11	5	1	1230	136.3	5.78	21.21
Master Protein	P01034	Cystatin-C OS=Homo sapiens GN=CST3 PE=1 SV=1	CST3	High	FALSE	0	37.254	31	4	102	4	1	146	15.8	8.75	306.96
Master Protein	Q15828	Cystatin-M OS=Homo sapiens GN=CST6 PE=1 SV=1	CST6	High	FALSE	0	7.968	21	2	7	2	1	149	16.5	8.09	19.74

Master Protein	P21291	Cysteine and glycine-rich protein 1 OS=Homo sapiens GN=CSRP1 PE=1 SV=3	CSRP1	High	FALSE	0	8.658	13	2	6	2	1	193	20.6	8.57	13.99
Master Protein	J3KPA1	Cysteine-rich secretory protein 3 OS=Homo sapiens GN=CRISP3 PE=4 SV=1	CRISP3	High	FALSE	0	11.599	13	3	16	3	1	276	31	7.61	38.1
Master Protein	P21399	Cytoplasmic aconitate hydratase OS=Homo sapiens GN=ACO1 PE=1 SV=3	ACO1	High	FALSE	0.01	3.227	2	1	2	1	1	889	98.3	6.68	3.51
Master Protein	P28838	Cytosol aminopeptidase OS=Homo sapiens GN=LAP3 PE=1 SV=3	LAP3	High	FALSE	0.006	4.071	2	1	7	1	1	519	56.1	7.93	9.21
Master Protein	Q96KP4	Cytosolic non-specific dipeptidase OS=Homo sapiens GN=CNDP2 PE=1 SV=2	CNDP2	High	FALSE	0.004	6.331	7	2	5	2	1	475	52.8	5.97	9.89
Master Protein	J3KQ18	D-dopachrome decarboxylase OS=Homo sapiens GN=DDT PE=1 SV=1	DDT	High	FALSE	0.001	6.729	14	2	13	2	1	132	14.2	7.3	14.44
Master Protein	Q14126	Desmoglein-2 OS=Homo sapiens GN=DSG2 PE=1 SV=2	DSG2	High	FALSE	0	23.257	6	6	66	6	1	1118	122.2	5.24	145.08
Master Protein	P60981	Destrin OS=Homo sapiens GN=DSTN PE=1 SV=3	DSTN	High	FALSE	0.007	4.289	7	1	1	1	1	165	18.5	7.85	2.23
Master Protein	Q01459	Di-N-acetylchitobiase OS=Homo sapiens GN=CTBS PE=1 SV=1	CTBS	High	FALSE	0	8.105	6	2	34	2	1	385	43.7	6.64	75.49
Master Protein	Q9H4A9	Dipeptidase 2 OS=Homo sapiens GN=DPEP2 PE=1 SV=2	DPEP2	High	FALSE	0	10.368	6	2	10	2	1	486	53.3	6.4	26.73
Master Protein	G3V180	Dipeptidyl peptidase 3 OS=Homo sapiens GN=DPP3 PE=1 SV=1	DPP3	High	FALSE	0.004	5.534	3	2	2	2	1	757	84.3	5.03	4.36
Master Protein	P27487	Dipeptidyl peptidase 4 OS=Homo sapiens GN=DPP4 PE=1 SV=2	DPP4	High	FALSE	0	38.396	14	11	137	11	1	766	88.2	6.04	345.61
Master Protein	P09172	Dopamine beta-hydroxylase OS=Homo sapiens GN=DBH PE=1 SV=3	DBH	High	FALSE	0	66.193	25	11	212	11	1	617	69	6.42	574.68
Master Protein	Q9UJU6	Drebrin-like protein OS=Homo sapiens GN=DBNL PE=1 SV=1	DBNL	High	FALSE	0	12.693	10	3	8	3	1	430	48.2	5.05	18.78
Master Protein	P51452	Dual specificity protein phosphatase 3 OS=Homo sapiens GN=DUSP3 PE=1 SV=1	DUSP3	High	FALSE	0.006	4.111	6	1	2	1	1	185	20.5	7.8	4.64
Master Protein	Q14118	Dystroglycan OS=Homo sapiens GN=DAG1 PE=1 SV=2	DAG1	High	FALSE	0.001	7.544	4	2	10	2	1	895	97.4	8.56	21.08
Master Protein	Q13508	Ecto-ADP-ribosyltransferase 3 OS=Homo sapiens GN=ART3 PE=1 SV=2	ART3	High	FALSE	0	10.059	10	3	8	3	1	389	43.9	6.06	9.18
Master Protein	O75356	Ectonucleoside triphosphate diphosphohydrolase 5 OS=Homo sapiens GN=ENTPD5 PE=1 SV=1	ENTPD5	High	FALSE	0.007	3.898	3	1	1	1	1	428	47.5	6.33	3.34
Master Protein	Q9HBW9	EGF, latrophilin and seven transmembrane domain-containing protein 1 OS=Homo sapiens GN=ELTD1 PE=1 SV=3	ELTD1	High	FALSE	0.006	4.041	2	1	4	1	1	690	77.8	7.61	10.14
Master Protein	Q12805	EGF-containing fibulin-like extracellular matrix protein 1 OS=Homo sapiens GN=EFEMP1 PE=1 SV=2	EFEMP1	High	FALSE	0	47.393	24	9	160	9	1	493	54.6	5.07	362.93
Master Protein	A0A024R571	EH domain-containing protein 1 OS=Homo sapiens GN=EHD1 PE=1 SV=1	EHD1	High	FALSE	0	10.495	6	3	7	1	1	548	61.9	6.71	8.05
Master Protein	Q9NZN3	EH domain-containing protein 3 OS=Homo sapiens GN=EHD3 PE=1 SV=2	EHD3	High	FALSE	0	13.22	8	4	8	2	1	535	60.8	6.57	10.01
Master Protein	P68104	Elongation factor 1-alpha 1 OS=Homo sapiens GN=EEF1A1 PE=1 SV=1	EEF1A1	High	FALSE	0	8.505	9	3	6	3	1	462	50.1	9.01	7.42
Master Protein	P17813	Endoglin OS=Homo sapiens GN=ENG PE=1 SV=2	ENG	High	FALSE	0	14.077	7	3	18	3	1	658	70.5	6.61	51.86
Master Protein	P30040	Endoplasmic reticulum resident protein 29 OS=Homo sapiens GN=ERP29 PE=1 SV=4	ERP29	High	FALSE	0.004	6.023	10	2	6	2	1	261	29	7.31	14.22
Master Protein	Q9BS26	Endoplasmic reticulum resident protein 44 OS=Homo sapiens GN=ERP44 PE=1 SV=1	ERP44	High	FALSE	0	13.808	10	3	16	3	1	406	46.9	5.26	43.82
Master Protein	P14625	Endoplasmin OS=Homo sapiens GN=HSP90B1 PE=1 SV=1	HSP90B1	High	FALSE	0	62.954	21	14	169	13	1	803	92.4	4.84	409.56
Master Protein	Q9HCU0	Endosialin OS=Homo sapiens GN=CD248 PE=1 SV=1	CD248	High	FALSE	0	10.657	4	3	13	3	1	757	80.8	5.35	29.15
Master Protein	Q9UNN8	Endothelial protein C receptor OS=Homo sapiens GN=PROCR PE=1 SV=1	PROCR	High	FALSE	0	11.219	15	3	59	3	1	238	26.7	7.18	137.5
Master Protein	P54764	Ephrin type-A receptor 4 OS=Homo sapiens GN=EPHA4 PE=1 SV=1	EPHA4	High	FALSE	0.007	3.788	2	1	3	1	1	986	109.8	6.61	5.74
Master Protein	P00533	Epidermal growth factor receptor OS=Homo sapiens GN=EGFR PE=1 SV=2	EGFR	High	FALSE	0	8.395	2	2	6	2	1	1210	134.2	6.68	16.25
Master Protein	P60842	Eukaryotic initiation factor 4A-I OS=Homo sapiens GN=EIF4A1 PE=1 SV=1	EIF4A1	High	FALSE	0	11.004	11	4	21	4	1	406	46.1	5.48	36.45
Master Protein	Q16394	Exostosin-1 OS=Homo sapiens GN=EXT1 PE=1 SV=2	EXT1	High	FALSE	0	13.384	6	4	18	4	1	746	86.2	9.04	40.12

Master Protein	Q9UBQ6	Exostosin-like 2 OS=Homo sapiens GN=EXTL2 PE=1 SV=1	EXTL2	High	FALSE	0	9.078	9	2	13	2	1	330	37.4	8.95	28.03
Master Protein	Q16610	Extracellular matrix protein 1 OS=Homo sapiens GN=ECM1 PE=1 SV=2	ECM1	High	FALSE	0	159.826	60	25	1171	25	1	540	60.6	6.71	2867.31
Master Protein	Q8IXL6	Extracellular serine/threonine protein kinase FAM20C OS=Homo sapiens GN=FAM20C PE=1 SV=2	FAM20C	High	FALSE	0	8.539	5	2	27	2	1	584	66.2	7.74	45.58
Master Protein	P08294	Extracellular superoxide dismutase [Cu-Zn] OS=Homo sapiens GN=SOD3 PE=1 SV=2	SOD3	High	FALSE	0	36.286	34	5	73	5	1	240	25.8	6.61	266.63
Master Protein	P52907	F-actin-capping protein subunit alpha-1 OS=Homo sapiens GN=CAPZA1 PE=1 SV=3	CAPZA1	High	FALSE	0	12.781	14	3	9	3	1	286	32.9	5.69	20.21
Master Protein	P07148	Fatty acid-binding protein, liver OS=Homo sapiens GN=FABP1 PE=1 SV=1	FABP1	High	FALSE	0.001	6.763	17	2	9	2	1	127	14.2	7.18	9.36
Master Protein	Q96RD9	Fc receptor-like protein 5 OS=Homo sapiens GN=FCRL5 PE=1 SV=3	FCRL5	High	FALSE	0	9.769	5	3	4	3	1	977	106.4	7.14	10.31
Master Protein	Q86UX7	Fermitin family homolog 3 OS=Homo sapiens GN=FERMT3 PE=1 SV=1	FERMT3	High	FALSE	0	151.278	52	26	600	26	1	667	75.9	6.98	1676.99
Master Protein	P02792	Ferritin light chain OS=Homo sapiens GN=FTL PE=1 SV=2	FTL	High	FALSE	0	10.282	17	2	12	2	1	175	20	5.78	30.63
Master Protein	Q9UGM5	Fetuin-B OS=Homo sapiens GN=FETUB PE=1 SV=2	FETUB	High	FALSE	0	71.796	40	13	399	13	1	382	42	6.83	762.66
Master Protein	P35555	Fibrillin-1 OS=Homo sapiens GN=FBN1 PE=1 SV=3	FBN1	High	FALSE	0.001	6.689	1	2	3	2	1	2871	312	4.93	5.62
Master Protein	P02671	Fibrinogen alpha chain OS=Homo sapiens GN=FGA PE=1 SV=2	FGA	High	FALSE	0	758.591	58	72	27210	72	1	866	94.9	6.01	75577.02
Master Protein	P02675	Fibrinogen beta chain OS=Homo sapiens GN=FGB PE=1 SV=2	FGB	High	FALSE	0	543.421	77	48	27677	48	1	491	55.9	8.27	84896.41
Master Protein	C9JU00	Fibrinogen gamma chain (Fragment) OS=Homo sapiens GN=FGG PE=1 SV=1	FGG	High	FALSE	0	163.099	97	19	9211	2	1	123	14	7.2	24749.31
Master Protein	Q08830	Fibrinogen-like protein 1 OS=Homo sapiens GN=FGL1 PE=1 SV=3	FGL1	High	FALSE	0	11.291	10	3	11	3	1	312	36.4	5.87	21.41
Master Protein	P11362	Fibroblast growth factor receptor 1 OS=Homo sapiens GN=FGFR1 PE=1 SV=3	FGFR1	High	FALSE	0	13.007	5	3	14	3	1	822	91.8	6.21	30.75
Master Protein	P02751	Fibronectin OS=Homo sapiens GN=FN1 PE=1 SV=4	FN1	High	FALSE	0	840.535	53	93	18163	6	1	2386	262.5	5.71	56253.25
Master Protein	P23142	Fibulin-1 OS=Homo sapiens GN=FBLN1 PE=1 SV=4	FBLN1	High	FALSE	0	106.272	30	15	741	5	1	703	77.2	5.22	1652.84
Master Protein	B1AHL2	Fibulin-1 OS=Homo sapiens GN=FBLN1 PE=3 SV=1	FBLN1	High	FALSE	0	104.072	30	15	691	5	1	721	78.3	5.39	1572.26
Master Protein	Q53RD9	Fibulin-7 OS=Homo sapiens GN=FBLN7 PE=2 SV=1	FBLN7	High	FALSE	0.004	5.22	4	2	9	2	1	439	47.3	7.62	9.24
Master Protein	A0A087WVE2	Ficolin-1 OS=Homo sapiens GN=FCN1 PE=4 SV=1	FCN1	High	FALSE	0.006	3.523	3	1	3	1	1	360	39.2	8.46	7.29
Master Protein	O75636	Ficolin-3 OS=Homo sapiens GN=FCN3 PE=1 SV=2	FCN3	High	FALSE	0	87.928	52	12	869	12	1	299	32.9	6.67	2102.29
Master Protein	P21333	Filamin-A OS=Homo sapiens GN=FLNA PE=1 SV=4	FLNA	High	FALSE	0	304.019	33	62	530	58	1	2647	280.6	6.06	1301.02
Master Protein	P30043	Flavin reductase (NADPH) OS=Homo sapiens GN=BLVRB PE=1 SV=3	BLVRB	High	FALSE	0	43.206	38	5	85	5	1	206	22.1	7.65	218.68
Master Protein	P09467	Fructose-1,6-bisphosphatase 1 OS=Homo sapiens GN=FBP1 PE=1 SV=5	FBP1	High	FALSE	0.001	7.176	10	2	9	2	1	338	36.8	6.99	15.49
Master Protein	P05062	Fructose-bisphosphate aldolase B OS=Homo sapiens GN=ALDOB PE=1 SV=2	ALDOB	High	FALSE	0	38.805	32	9	113	8	1	364	39.4	7.87	291.7
Master Protein	P09972	Fructose-bisphosphate aldolase C OS=Homo sapiens GN=ALDOC PE=1 SV=2	ALDOC	High	FALSE	0	21.893	15	4	129	3	1	364	39.4	6.87	239.15
Master Protein	P16930	Fumarylacetoacetase OS=Homo sapiens GN=FAH PE=1 SV=2	FAH	High	FALSE	0	29.818	20	7	61	7	1	419	46.3	6.95	151.05
Master Protein	Q08380	Galectin-3-binding protein OS=Homo sapiens GN=LGALS3BP PE=1 SV=1	LGALS3BP	High	FALSE	0	90.505	35	14	540	14	1	585	65.3	5.27	1339.36
Master Protein	Q3ZCW2	Galectin-related protein OS=Homo sapiens GN=LGALSL PE=1 SV=2	LGALSL	High	FALSE	0	19.787	23	3	66	3	1	172	19	5.35	128.36
Master Protein	P09104	Gamma-enolase OS=Homo sapiens GN=ENO2 PE=1 SV=3	ENO2	High	FALSE	0	19.446	12	3	35	1	1	434	47.2	5.03	107.22
Master Protein	Q92820	Gamma-glutamyl hydrolase OS=Homo sapiens GN=GGH PE=1 SV=2	GGH	High	FALSE	0	29.872	21	6	83	6	1	318	35.9	7.11	186.23
Master Protein	P17900	Ganglioside GM2 activator OS=Homo sapiens GN=GM2A PE=1 SV=4	GM2A	High	FALSE	0.007	3.856	5	1	1	1	1	193	20.8	5.31	2.36
Master Protein	Q9NS71	Gastrokine-1 OS=Homo sapiens GN=GKN1 PE=2 SV=3	GKN1	High	FALSE	0.006	4.436	7	1	1	1	1	199	22	6.32	2.79
Master Protein	R4GMU1	GDH/6PGL endoplasmic bifunctional protein OS=Homo sapiens GN=H6PD PE=4 SV=1	H6PD	High	FALSE	0	45.054	15	11	55	11	1	802	90.1	7.23	136.3
Master Protein	P06396	Gelsolin OS=Homo sapiens GN=GSN PE=1 SV=1	GSN	High	FALSE	0	377.769	58	40	6156	40	1	782	85.6	6.28	20189.69
Master Protein	M0QXZ6	Glia maturation factor gamma (Fragment) OS=Homo sapiens GN=GMFG PE=4 SV=1	GMFG	High	FALSE	0.004	5.492	27	1	4	1	1	59	6.6	8.97	10.82

Master Protein	O60234	Glia maturation factor gamma OS=Homo sapiens GN=GMFG PE=1 SV=1	GMFG	High	FALSE	0	12.201	27	4	21	4	1	142	16.8	5.26	42.54
Master Protein	K7ELL7	Glucosidase 2 subunit beta OS=Homo sapiens GN=PRKCSH PE=1 SV=1	PRKCSH	High	FALSE	0	18.473	12	5	24	5	1	535	60.2	4.41	59.92
Master Protein	P48506	Glutamate--cysteine ligase catalytic subunit OS=Homo sapiens GN=GCLC PE=1 SV=2	GCLC	High	FALSE	0.001	7.165	3	2	5	2	1	637	72.7	6.09	9.39
Master Protein	Q07075	Glutamyl aminopeptidase OS=Homo sapiens GN=ENPEP PE=1 SV=3	ENPEP	High	FALSE	0	13.805	5	4	11	4	1	957	109.2	5.47	12.9
Master Protein	P35754	Glutaredoxin-1 OS=Homo sapiens GN=GLRX PE=1 SV=2	GLRX	High	FALSE	0.006	3.966	10	1	1	1	1	106	11.8	8.09	2.38
Master Protein	P22352	Glutathione peroxidase 3 OS=Homo sapiens GN=GPX3 PE=1 SV=2	GPX3	High	FALSE	0	37.669	32	6	1012	6	1	226	25.5	8.13	1823.17
Master Protein	P00390	Glutathione reductase, mitochondrial OS=Homo sapiens GN=GSR PE=1 SV=2	GSR	High	FALSE	0	18.945	13	5	27	5	1	522	56.2	8.5	56.9
Master Protein	P78417	Glutathione S-transferase omega-1 OS=Homo sapiens GN=GSTO1 PE=1 SV=2	GSTO1	High	FALSE	0	32.64	34	8	117	8	1	241	27.5	6.6	268.25
Master Protein	P09211	Glutathione S-transferase P OS=Homo sapiens GN=GSTP1 PE=1 SV=2	GSTP1	High	FALSE	0	13.63	29	4	25	4	1	210	23.3	5.64	33.06
Master Protein	P48637	Glutathione synthetase OS=Homo sapiens GN=GSS PE=1 SV=1	GSS	High	FALSE	0	10.784	5	3	22	3	1	474	52.4	5.92	50.1
Master Protein	P04406	Glyceraldehyde-3-phosphate dehydrogenase OS=Homo sapiens GN=GAPDH PE=1 SV=3	GAPDH	High	FALSE	0	35.544	27	6	149	6	1	335	36	8.46	395.63
Master Protein	P11216	Glycogen phosphorylase, brain form OS=Homo sapiens GN=PYGB PE=1 SV=5	PYGB	High	FALSE	0	16.745	7	5	24	2	1	843	96.6	6.86	58.72
Master Protein	P06737	Glycogen phosphorylase, liver form OS=Homo sapiens GN=PYGL PE=1 SV=4	PYGL	High	FALSE	0	14.012	6	5	15	3	1	847	97.1	7.17	29.42
Master Protein	P11217	Glycogen phosphorylase, muscle form OS=Homo sapiens GN=PYGM PE=1 SV=6	PYGM	High	FALSE	0	34.348	11	9	25	6	1	842	97	7.03	58.33
Master Protein	F6TLX2	Glyoxalase domain-containing protein 4 OS=Homo sapiens GN=GLOD4 PE=1 SV=1	GLOD4	High	FALSE	0	8.528	5	2	8	2	1	502	54.7	8.7	17.14
Master Protein	Q8NBJ4	Golgi membrane protein 1 OS=Homo sapiens GN=GOLM1 PE=1 SV=1	GOLM1	High	FALSE	0.004	5.347	4	2	8	2	1	401	45.3	4.97	14.82
Master Protein	Q9H4G4	Golgi-associated plant pathogenesis-related protein 1 OS=Homo sapiens GN=GLIPR2 PE=1 SV=3	GLIPR2	High	FALSE	0	9.336	17	2	36	2	1	154	17.2	9.41	116.72
Master Protein	P62993	Growth factor receptor-bound protein 2 OS=Homo sapiens GN=GRB2 PE=1 SV=1	GRB2	High	FALSE	0.006	4.09	6	1	6	1	1	217	25.2	6.32	13.35
Master Protein	O14793	Growth/differentiation factor 8 OS=Homo sapiens GN=MSTN PE=1 SV=1	MSTN	High	FALSE	0.008	3.291	3	1	2	1	1	375	42.7	6.76	3.6
Master Protein	J3KQE5	GTP-binding nuclear protein Ran (Fragment) OS=Homo sapiens GN=RAN PE=1 SV=1	RAN	High	FALSE	0	8.754	14	3	6	3	1	234	26.8	9.58	14.54
Master Protein	P16520	Guanine nucleotide-binding protein G(I)/G(S)/G(T) subunit beta-3 OS=Homo sapiens GN=GNB3 PE=1 SV=1	GNB3	High	FALSE	0.006	3.687	3	1	2	1	1	340	37.2	5.67	2.5
Master Protein	P00738	Haptoglobin OS=Homo sapiens GN=HP PE=1 SV=1	HP	High	FALSE	0	160.068	55	23	2992	12	1	406	45.2	6.58	7325.58
Master Protein	I3L0A0	HCG2044781 OS=Homo sapiens GN=TMEM189-UBE2V1 PE=4 SV=1	TMEM189-UBE2V1	High	FALSE	0.004	5.097	5	2	8	2	1	370	42.2	6.71	10.24
Master Protein	P08107	Heat shock 70 kDa protein 1A/1B OS=Homo sapiens GN=HSPA1A PE=1 SV=5	HSPA1A	High	FALSE	0	40.033	26	10	55	8	1	641	70	5.66	141.48
Master Protein	P34932	Heat shock 70 kDa protein 4 OS=Homo sapiens GN=HSPA4 PE=1 SV=4	HSPA4	High	FALSE	0	10.819	5	3	9	3	1	840	94.3	5.19	21.92
Master Protein	P11142	Heat shock cognate 71 kDa protein OS=Homo sapiens GN=HSPA8 PE=1 SV=1	HSPA8	High	FALSE	0	91.481	33	17	156	14	1	646	70.9	5.52	398.82
Master Protein	P04792	Heat shock protein beta-1 OS=Homo sapiens GN=HSPB1 PE=1 SV=2	HSPB1	High	FALSE	0.007	4.224	5	1	5	1	1	205	22.8	6.4	11.55
Master Protein	P08238	Heat shock protein HSP 90-beta OS=Homo sapiens GN=HSP90AB1 PE=1 SV=4	HSP90AB1	High	FALSE	0	22.507	9	6	64	1	1	724	83.2	5.03	153.81
Master Protein	P69905	Hemoglobin subunit alpha OS=Homo sapiens GN=HBA1 PE=1 SV=2	HBA1	High	FALSE	0	112.729	64	9	2769	8	1	142	15.2	8.68	7930.8
Master Protein	P68871	Hemoglobin subunit beta OS=Homo sapiens GN=HBB PE=1 SV=2	HBB	High	FALSE	0	135.718	80	13	4677	7	1	147	16	7.28	13245.42
Master Protein	P02042	Hemoglobin subunit delta OS=Homo sapiens GN=HBD PE=1 SV=2	HBD	High	FALSE	0	115.959	79	11	1934	5	1	147	16	8.05	5763.88
Master Protein	P69891	Hemoglobin subunit gamma-1 OS=Homo sapiens GN=HBG1 PE=1 SV=2	HBG1	High	FALSE	0	34.491	66	8	157	1	1	147	16.1	7.2	383.07
Master Protein	P02008	Hemoglobin subunit zeta OS=Homo sapiens GN=HBZ PE=1 SV=2	HBZ	High	FALSE	0	11.038	23	3	82	2	1	142	15.6	8.21	175.48

Master Protein	P02790	Hemopexin OS=Homo sapiens GN=HPX PE=1 SV=2	HPX	High	FALSE	0	278.202	70	37	20151	37	1	462	51.6	7.02	51036.4
Master Protein	Q9Y251	Heparanase OS=Homo sapiens GN=HPSE PE=1 SV=2	HPSE	High	FALSE	0.004	5.543	3	2	4	2	1	543	61.1	9.28	6.92
Master Protein	P05546	Heparin cofactor 2 OS=Homo sapiens GN=SERPIND1 PE=1 SV=3	SERPIND1	High	FALSE	0	188.387	54	25	6018	25	1	499	57	6.9	14487.42
Master Protein	E7EUJ1	Hepatic triacylglycerol lipase OS=Homo sapiens GN=LIPC PE=3 SV=1	LIPC	High	FALSE	0.008	3.353	4	1	1	1	1	499	55.6	8.65	2.47
Master Protein	Q04756	Hepatocyte growth factor activator OS=Homo sapiens GN=HGFAC PE=1 SV=1	HGFAC	High	FALSE	0	73.122	24	13	706	1	1	655	70.6	7.24	1813.57
Master Protein	D6RAR4	Hepatocyte growth factor activator OS=Homo sapiens GN=HGFAC PE=3 SV=1	HGFAC	High	FALSE	0	73.651	24	13	605	1	1	662	71.4	7.05	1619.12
Master Protein	G3XAK1	Hepatocyte growth factor-like protein alpha chain OS=Homo sapiens GN=MST1 PE=3 SV=1	MST1	High	FALSE	0	146.867	39	23	781	23	1	725	81.9	7.8	1669.38
Master Protein	P37235	Hippocalcin-like protein 1 OS=Homo sapiens GN=HPCAL1 PE=1 SV=3	HPCAL1	High	FALSE	0.004	6.122	13	2	4	2	1	193	22.3	5.35	1.85
Master Protein	P04196	Histidine-rich glycoprotein OS=Homo sapiens GN=HRG PE=1 SV=1	HRG	High	FALSE	0	197.813	43	23	4968	23	1	525	59.5	7.5	13163.4
Master Protein	P05534	HLA class I histocompatibility antigen, A-24 alpha chain OS=Homo sapiens GN=HLA-A PE=1 SV=2	HLA-A	High	FALSE	0	56.906	37	10	104	7	1	365	40.7	6.34	247.39
Master Protein	P01889	HLA class I histocompatibility antigen, B-7 alpha chain OS=Homo sapiens GN=HLA-B PE=1 SV=3	HLA-B	High	FALSE	0	14.485	13	4	26	1	1	362	40.4	5.85	65.01
Master Protein	P50502	Hsc70-interacting protein OS=Homo sapiens GN=ST13 PE=1 SV=2	ST13	High	FALSE	0.001	6.987	7	2	6	2	1	369	41.3	5.27	5.77
Master Protein	Q14520	Hyaluronan-binding protein 2 OS=Homo sapiens GN=HABP2 PE=1 SV=1	HABP2	High	FALSE	0	108.569	31	15	1141	15	1	560	62.6	6.54	2502.67
Master Protein	Q9Y4L1	Hypoxia up-regulated protein 1 OS=Homo sapiens GN=HYOU1 PE=1 SV=1	HYOU1	High	FALSE	0	47.815	9	7	47	7	1	999	111.3	5.22	98.1
Master Protein	K4DIA0	ICOS ligand OS=Homo sapiens GN=ICOSLG PE=4 SV=1	ICOSLG	High	FALSE	0	11.107	8	3	21	3	1	473	51.9	8.05	45.61
Master Protein	P01876	Ig alpha-1 chain C region OS=Homo sapiens GN=IGHA1 PE=1 SV=2	IGHA1	High	FALSE	0	89.794	45	13	816	9	1	353	37.6	6.51	2093.65
Master Protein	A0A075B6N7	Ig alpha-2 chain C region (Fragment) OS=Homo sapiens GN=IGHA2 PE=4 SV=1	IGHA2	High	FALSE	0	36.555	29	8	314	4	1	340	36.6	6.27	655.83
Master Protein	A0A087WYC5	Ig gamma-1 chain C region OS=Homo sapiens GN=IGHG1 PE=1 SV=1	IGHG1	High	FALSE	0	34.875	21	8	202	1	1	475	52.4	8.21	505.16
Master Protein	A0A087WV47	Ig gamma-1 chain C region OS=Homo sapiens GN=IGHG1 PE=1 SV=1	IGHG1	High	FALSE	0	37.713	24	9	217	2	1	466	51.1	7.55	561.45
Master Protein	P01859	Ig gamma-2 chain C region OS=Homo sapiens GN=IGHG2 PE=1 SV=2	IGHG2	High	FALSE	0	23.564	22	6	162	2	1	326	35.9	7.59	359.27
Master Protein	A0A087WXL8	Ig gamma-3 chain C region OS=Homo sapiens GN=IGHG3 PE=4 SV=1	IGHG3	High	FALSE	0	65.598	31	11	540	6	1	517	56.9	8.25	1453.08
Master Protein	P06331	Ig heavy chain V-II region ARH-77 OS=Homo sapiens PE=4 SV=1	IGHV4-34	High	FALSE	0	8.231	17	2	51	2	1	146	16.2	8.28	102.05
Master Protein	P01765	Ig heavy chain V-III region TIL OS=Homo sapiens PE=1 SV=1	IGHV3-23	High	FALSE	0	13.91	26	2	21	2	1	115	12.3	9.13	59.95
Master Protein	A0A087WTX5	Ig kappa chain C region OS=Homo sapiens GN=IGKC PE=4 SV=1	IGKC	High	FALSE	0	58.5	36	6	1518	1	1	236	25.6	7.97	3264.11
Master Protein	A0A087X130	Ig kappa chain C region OS=Homo sapiens GN=IGKC PE=4 SV=1	IGKC	High	FALSE	0	62.236	50	7	1529	2	1	231	25.1	6.32	3284.66
Master Protein	P01598	Ig kappa chain V-I region EU OS=Homo sapiens PE=1 SV=1	IGKV1-5	High	FALSE	0	18.284	27	2	46	2	1	108	11.8	8.44	137.1
Master Protein	P01611	Ig kappa chain V-I region Wes OS=Homo sapiens PE=1 SV=1	IGKV1D-12	High	FALSE	0.006	4.357	17	1	5	1	1	108	11.6	7.28	11.74
Master Protein	P01617	Ig kappa chain V-II region TEW OS=Homo sapiens PE=1 SV=1	IGKV2D-28	High	FALSE	0	9.74	33	2	70	2	1	113	12.3	6	141.55
Master Protein	P01620	Ig kappa chain V-III region SIE OS=Homo sapiens PE=1 SV=1	IGKV3-20	High	FALSE	0	21.591	46	4	92	4	1	109	11.8	8.48	243.84
Master Protein	P06313	Ig kappa chain V-IV region JI OS=Homo sapiens PE=4 SV=1	IGKV4-1	High	FALSE	0	8.093	20	2	5	1	1	133	14.6	6.58	9.14
Master Protein	P01625	Ig kappa chain V-IV region Len OS=Homo sapiens PE=1 SV=2	IGKV4-1	High	FALSE	0	26.197	29	2	48	1	1	114	12.6	7.93	155.47
Master Protein	P01701	Ig lambda chain V-I region NEW OS=Homo sapiens PE=1 SV=1	IGLV1-51	High	FALSE	0.01	3.211	15	1	1	1	1	111	11.4	8	2.57
Master Protein	P01703	Ig lambda chain V-I region NEWM OS=Homo sapiens PE=1 SV=1	IGLV1-40	High	FALSE	0.006	3.634	17	1	4	1	1	103	10.9	9.29	3.96
Master Protein	P80748	Ig lambda chain V-III region LOI OS=Homo sapiens PE=1 SV=1	IGLV3-21	High	FALSE	0.001	6.545	22	2	15	2	1	111	11.9	5.08	37.08
Master Protein	P01717	Ig lambda chain V-IV region Hil OS=Homo sapiens PE=1 SV=1	IGLV3-25	High	FALSE	0	9.446	18	1	12	1	1	107	11.5	6.51	30.27
Master Protein	A0A075B6L0	Ig lambda-3 chain C regions (Fragment) OS=Homo sapiens GN=IGLC3 PE=4 SV=2	IGLC3	High	FALSE	0	48.951	65	6	953	4	1	106	11.3	7.24	2160.66
Master Protein	A0A087WYJ9	Ig mu chain C region OS=Homo sapiens GN=IGHM PE=1 SV=1	IGHM	High	FALSE	0	176.965	39	22	4462	10	1	599	65.7	6.95	11472.94
Master Protein	P04220	Ig mu heavy chain disease protein OS=Homo sapiens PE=1 SV=1	IGHM	High	FALSE	0	101.396	44	14	2408	2	1	391	43	5.24	6297.63

Master Protein	Q9Y6R7	IgGFc-binding protein OS=Homo sapiens GN=FCGBP PE=1 SV=3	FCGBP	High	FALSE	0	227.515	18	45	560	45	1	5405	571.6	5.34	1421.41
Master Protein	P01591	Immunoglobulin J chain OS=Homo sapiens GN=IGJ PE=1 SV=4	IGJ	High	FALSE	0	20.818	28	4	91	4	1	159	18.1	5.24	225.07
Master Protein	A0A087WX49	Immunoglobulin lambda-like polypeptide 5 OS=Homo sapiens GN=IGLL5 PE=4 SV=1	IGLL5	High	FALSE	0	42.19	26	5	470	3	1	235	24.9	6.52	1113.36
Master Protein	O14498	Immunoglobulin superfamily containing leucine-rich repeat protein OS=Homo sapiens GN=ISLR PE=1 SV=1	ISLR	High	FALSE	0.004	5.596	5	2	6	2	1	428	46	5.15	6.04
Master Protein	Q8TDY8	Immunoglobulin superfamily DCC subclass member 4 OS=Homo sapiens GN=IGDCC4 PE=1 SV=1	IGDCC4	High	FALSE	0.001	6.689	2	2	5	2	1	1250	134.1	6.2	9.32
Master Protein	Q14974	Importin subunit beta-1 OS=Homo sapiens GN=KPNB1 PE=1 SV=2	KPNB1	High	FALSE	0	7.706	2	2	3	2	1	876	97.1	4.78	7.17
Master Protein	Q14623	Indian hedgehog protein OS=Homo sapiens GN=IHH PE=1 SV=4	IHH	High	FALSE	0	21.41	16	5	30	5	1	411	45.2	8.76	83.34
Master Protein	P55103	Inhibin beta C chain OS=Homo sapiens GN=INHBC PE=1 SV=1	INHBC	High	FALSE	0	22.549	16	4	30	4	1	352	38.2	7.11	68.77
Master Protein	P58166	Inhibin beta E chain OS=Homo sapiens GN=INHBE PE=1 SV=1	INHBE	High	FALSE	0.005	4.491	3	1	2	1	1	350	38.5	9.25	9.2
Master Protein	P05019	Insulin-like growth factor I OS=Homo sapiens GN=IGF1 PE=1 SV=1	IGF1	High	FALSE	0.004	4.985	7	1	13	1	1	195	21.8	9.72	30.96
Master Protein	P18065	Insulin-like growth factor-binding protein 2 OS=Homo sapiens GN=IGFBP2 PE=1 SV=2	IGFBP2	High	FALSE	0	33.157	21	5	62	5	1	325	34.8	7.5	174.65
Master Protein	P22692	Insulin-like growth factor-binding protein 4 OS=Homo sapiens GN=IGFBP4 PE=1 SV=2	IGFBP4	High	FALSE	0	12.847	15	3	18	3	1	258	27.9	7.15	39.81
Master Protein	P24593	Insulin-like growth factor-binding protein 5 OS=Homo sapiens GN=IGFBP5 PE=1 SV=1	IGFBP5	High	FALSE	0	28.701	17	4	54	4	1	272	30.6	8.21	123.54
Master Protein	P24592	Insulin-like growth factor-binding protein 6 OS=Homo sapiens GN=IGFBP6 PE=1 SV=1	IGFBP6	High	FALSE	0	9.125	10	2	5	2	1	240	25.3	7.81	11.06
Master Protein	Q16270	Insulin-like growth factor-binding protein 7 OS=Homo sapiens GN=IGFBP7 PE=1 SV=1	IGFBP7	High	FALSE	0	15.102	15	4	20	4	1	282	29.1	7.9	50.06
Master Protein	P17301	Integrin alpha-2 OS=Homo sapiens GN=ITGA2 PE=1 SV=1	ITGA2	High	FALSE	0	13.68	4	5	17	5	1	1181	129.2	5.31	27.09
Master Protein	P08514	Integrin alpha-IIb OS=Homo sapiens GN=ITGA2B PE=1 SV=3	ITGA2B	High	FALSE	0	15.671	5	4	9	4	1	1039	113.3	5.38	20.95
Master Protein	P05556	Integrin beta-1 OS=Homo sapiens GN=ITGB1 PE=1 SV=2	ITGB1	High	FALSE	0	27.292	8	6	30	6	1	798	88.4	5.39	83.19
Master Protein	P05107	Integrin beta-2 OS=Homo sapiens GN=ITGB2 PE=1 SV=2	ITGB2	High	FALSE	0.007	3.792	1	1	2	1	1	769	84.7	6.95	4.62
Master Protein	P05106	Integrin beta-3 OS=Homo sapiens GN=ITGB3 PE=1 SV=2	ITGB3	High	FALSE	0.001	6.851	3	2	2	2	1	788	87	5.24	4.53
Master Protein	Q13418	Integrin-linked protein kinase OS=Homo sapiens GN=ILK PE=1 SV=2	ILK	High	FALSE	0	47.012	30	12	81	12	1	452	51.4	8.07	162.28
Master Protein	P19827	Inter-alpha-trypsin inhibitor heavy chain H1 OS=Homo sapiens GN=ITIH1 PE=1 SV=3	ITIH1	High	FALSE	0	363.475	42	34	10963	34	1	911	101.3	6.79	36735.87
Master Protein	P19823	Inter-alpha-trypsin inhibitor heavy chain H2 OS=Homo sapiens GN=ITIH2 PE=1 SV=2	ITIH2	High	FALSE	0	385.971	48	45	12729	45	1	946	106.4	6.86	31705.49
Master Protein	Q06033	Inter-alpha-trypsin inhibitor heavy chain H3 OS=Homo sapiens GN=ITIH3 PE=1 SV=2	ITIH3	High	FALSE	0	184.527	32	23	1661	23	1	890	99.8	5.74	4313.23
Master Protein	Q14624	Inter-alpha-trypsin inhibitor heavy chain H4 OS=Homo sapiens GN=ITIH4 PE=1 SV=4	ITIH4	High	FALSE	0	402.634	50	47	12241	1	1	930	103.3	6.98	33942.25
Master Protein	P05362	Intercellular adhesion molecule 1 OS=Homo sapiens GN=ICAM1 PE=1 SV=2	ICAM1	High	FALSE	0	26.095	12	5	60	5	1	532	57.8	7.99	162.46
Master Protein	P13598	Intercellular adhesion molecule 2 OS=Homo sapiens GN=ICAM2 PE=1 SV=2	ICAM2	High	FALSE	0	14.042	9	2	66	2	1	275	30.6	7.43	185.91
Master Protein	P32942	Intercellular adhesion molecule 3 OS=Homo sapiens GN=ICAM3 PE=1 SV=2	ICAM3	High	FALSE	0.007	3.803	2	1	3	1	1	547	59.5	5.43	8.13
Master Protein	P40189	Interleukin-6 receptor subunit beta OS=Homo sapiens GN=IL6ST PE=1 SV=2	IL6ST	High	FALSE	0	18.421	5	5	31	5	1	918	103.5	5.95	48.72
Master Protein	Q96CN7	Isochorismatase domain-containing protein 1 OS=Homo sapiens GN=ISOC1 PE=1 SV=3	ISOC1	High	FALSE	0.006	3.558	5	1	2	1	1	298	32.2	7.39	2.09
Master Protein	O75874	Isocitrate dehydrogenase [NADP] cytoplasmic OS=Homo sapiens GN=IDH1 PE=1 SV=2	IDH1	High	FALSE	0	20.324	11	4	13	4	1	414	46.6	7.01	30.15
Master Protein	Q9BWP8-10	Isoform 10 of Collectin-11 OS=Homo sapiens GN=COLEC11	COLEC11	High	FALSE	0	35.918	25	6	49	6	1	285	30.2	6.25	133.1
Master Protein	P02751-10	Isoform 10 of Fibronectin OS=Homo sapiens GN=FN1	FN1	High	FALSE	0	788.034	57	88	17910	1	1	2176	239.5	5.88	55390.5

Master Protein	Q8WZ42-12	Isoform 12 of Titin OS=Homo sapiens GN=TTN	TTN	High	FALSE	0.001	7.065	0	2	3	2	1	35991	3992.1	6.39	5.1
Master Protein	P61160-2	Isoform 2 of Actin-related protein 2 OS=Homo sapiens GN=ACTR2	ACTR2	High	FALSE	0	9.663	11	3	9	3	1	399	45.3	7.2	14.46
Master Protein	O15511-2	Isoform 2 of Actin-related protein 2/3 complex subunit 5 OS=Homo sapiens GN=ARPC5	ARPC5	High	FALSE	0.006	4.128	8	1	7	1	1	154	16.6	6.02	19.63
Master Protein	P08319-2	Isoform 2 of Alcohol dehydrogenase 4 OS=Homo sapiens GN=ADH4	ADH4	High	FALSE	0.001	6.352	5	2	2	2	1	399	42.6	7.91	2.46
Master Protein	P04217-2	Isoform 2 of Alpha-1B-glycoprotein OS=Homo sapiens GN=A1BG	A1BG	High	FALSE	0	138.275	56	16	3117	1	1	373	40.7	5.77	8670.5
Master Protein	P07355-2	Isoform 2 of Annexin A2 OS=Homo sapiens GN=ANXA2	ANXA2	High	FALSE	0.006	3.939	3	1	2	1	1	357	40.4	8.37	4.49
Master Protein	O14791-2	Isoform 2 of Apolipoprotein L1 OS=Homo sapiens GN=APOL1	APOL1	High	FALSE	0	86.801	31	11	1277	11	1	414	45.9	6.29	3441.58
Master Protein	P17858-2	Isoform 2 of ATP-dependent 6-phosphofructokinase, liver type OS=Homo sapiens GN=PFKL	PFKL	High	FALSE	0	16.179	6	5	11	5	1	827	90.1	7.83	22.37
Master Protein	Q9HBI1-2	Isoform 2 of Beta-parvin OS=Homo sapiens GN=PARVB	PARVB	High	FALSE	0	37.028	19	6	73	6	1	397	45.2	6.25	187.96
Master Protein	P55957-2	Isoform 2 of BH3-interacting domain death agonist OS=Homo sapiens GN=BID	BID	High	FALSE	0.008	3.451	5	1	1	1	1	241	26.8	7.08	1.93
Master Protein	P43251-2	Isoform 2 of Biotinidase OS=Homo sapiens GN=BTD	BTD	High	FALSE	0	101.408	23	10	810	10	1	545	61.5	6.14	1897.7
Master Protein	P23280-2	Isoform 2 of Carbonic anhydrase 6 OS=Homo sapiens GN=CA6	CA6	High	FALSE	0.004	6.313	7	2	6	2	1	313	35.3	8.4	13.58
Master Protein	P10909-2	Isoform 2 of Clusterin OS=Homo sapiens GN=CLU	CLU	High	FALSE	0	217.903	39	26	7359	26	1	501	57.8	6.68	21422.43
Master Protein	P13716-2	Isoform 2 of Delta-aminolevulinic acid dehydratase OS=Homo sapiens GN=ALAD	ALAD	High	FALSE	0	12.958	11	3	23	3	1	359	39	7.65	64.4
Master Protein	P81605-2	Isoform 2 of Dermcidin OS=Homo sapiens GN=DCD	DCD	High	FALSE	0	7.829	18	2	13	2	1	121	12.4	7.97	36.2
Master Protein	Q9NZ08-2	Isoform 2 of Endoplasmic reticulum aminopeptidase 1 OS=Homo sapiens GN=ERAP1	ERAP1	High	FALSE	0	24.354	8	7	35	7	1	948	107.8	6.28	77.33
Master Protein	P98095-2	Isoform 2 of Fibulin-2 OS=Homo sapiens GN=FBLN2	FBLN2	High	FALSE	0.008	3.423	1	1	3	1	1	1231	131.8	4.86	2.17
Master Protein	P04075-2	Isoform 2 of Fructose-bisphosphate aldolase A OS=Homo sapiens GN=ALDOA	ALDOA	High	FALSE	0	65.287	27	11	224	10	1	418	45.2	8.25	608.61
Master Protein	P06744-2	Isoform 2 of Glucose-6-phosphate isomerase OS=Homo sapiens GN=GPI	GPI	High	FALSE	0	23.956	14	6	25	6	1	569	64.3	8.91	44.55
Master Protein	P00739-2	Isoform 2 of Haptoglobin-related protein OS=Homo sapiens GN=HPR	HPR	High	FALSE	0	122.09	46	18	1374	7	1	385	43	7.08	3090.83
Master Protein	P07900-2	Isoform 2 of Heat shock protein HSP 90-alpha OS=Homo sapiens GN=HSP90AA1	HSP90AA1	High	FALSE	0	33.779	12	9	53	5	1	854	98.1	5.16	110.24
Master Protein	P08581-2	Isoform 2 of Hepatocyte growth factor receptor OS=Homo sapiens GN=MET	MET	High	FALSE	0	14.201	2	3	9	3	1	1408	157.6	7.33	15.25
Master Protein	P01880-2	Isoform 2 of Ig delta chain C region OS=Homo sapiens GN=IGHD	IGHD	High	FALSE	0	29.292	19	5	74	5	1	430	47.4	7.2	221.54
Master Protein	P17936-2	Isoform 2 of Insulin-like growth factor-binding protein 3 OS=Homo sapiens GN=IGFBP3	IGFBP3	High	FALSE	0	55.083	29	7	601	7	1	297	32.2	8.62	1545.81
Master Protein	P35858-2	Isoform 2 of Insulin-like growth factor-binding protein complex acid labile subunit OS=Homo sapiens GN=IGFALS	IGFALS	High	FALSE	0	145.553	40	21	2044	21	1	643	70.2	6.93	5729.69
Master Protein	P11215-2	Isoform 2 of Integrin alpha-M OS=Homo sapiens GN=ITGAM	ITGAM	High	FALSE	0.01	3.223	1	1	3	1	1	1153	127.2	7.23	1.8
Master Protein	Q14624-2	Isoform 2 of Inter-alpha-trypsin inhibitor heavy chain H4 OS=Homo sapiens GN=ITIH4	ITIH4	High	FALSE	0	412.364	52	48	12069	3	1	914	101.2	6.65	33459
Master Protein	Q14847-2	Isoform 2 of LIM and SH3 domain protein 1 OS=Homo sapiens GN=LASP1	LASP1	High	FALSE	0	10.871	10	3	8	3	1	323	36	8.62	20.24
Master Protein	P23141-2	Isoform 2 of Liver carboxylesterase 1 OS=Homo sapiens GN=CES1	CES1	High	FALSE	0	29.39	19	9	34	9	1	568	62.6	6.6	69.37
Master Protein	P14151-2	Isoform 2 of L-selectin OS=Homo sapiens GN=SELL	SELL	High	FALSE	0	17.198	12	4	127	4	1	385	43.6	7.15	148.62
Master Protein	P42785-2	Isoform 2 of Lysosomal Pro-X carboxypeptidase OS=Homo sapiens GN=PRCP	PRCP	High	FALSE	0	8.516	6	2	10	2	1	517	58.1	7.4	28.76
Master Protein	P48740-2	Isoform 2 of Mannan-binding lectin serine protease 1 OS=Homo sapiens GN=MASP1	MASP1	High	FALSE	0	85.378	27	16	527	5	1	728	81.8	5.12	1433.87
Master Protein	Q7L9L4-2	Isoform 2 of MOB kinase activator 1B OS=Homo sapiens GN=MOB1B	MOB1B	High	FALSE	0.001	6.675	10	2	12	2	1	221	25.5	6.15	23.4
Master Protein	Q96PD5-2	Isoform 2 of N-acetylmuramoyl-L-alanine amidase OS=Homo sapiens GN=PGLYRP2	PGLYRP2	High	FALSE	0	174.205	33	16	2319	16	1	634	68	7.68	6120.46

Master Protein	O00533-2	Isoform 2 of Neural cell adhesion molecule L1-like protein OS=Homo sapiens GN=CHL1	CHL1	High	FALSE	0	108.288	24	22	242	22	1	1224	136.6	5.8	559.82
Master Protein	Q14697-2	Isoform 2 of Neutral alpha-glucosidase AB OS=Homo sapiens GN=GANAB	GANAB	High	FALSE	0	15.516	5	5	34	5	1	966	109.4	6.24	59.92
Master Protein	P15531-2	Isoform 2 of Nucleoside diphosphate kinase A OS=Homo sapiens GN=NME1	NME1	High	FALSE	0	14.239	23	3	7	3	1	177	19.6	5.58	13.35
Master Protein	Q99733-2	Isoform 2 of Nucleosome assembly protein 1-like 4 OS=Homo sapiens GN=NAP1L4	NAP1L4	High	FALSE	0.004	5.612	7	2	2	1	1	386	44.1	4.7	5.39
Master Protein	Q15084-2	Isoform 2 of Protein disulfide-isomerase A6 OS=Homo sapiens GN=PDIA6	PDIA6	High	FALSE	0	13.854	9	3	16	3	1	492	53.9	5.33	41.83
Master Protein	P12931-2	Isoform 2 of Proto-oncogene tyrosine-protein kinase Src OS=Homo sapiens GN=SRC	SRC	High	FALSE	0	10.009	8	3	14	3	1	542	60.6	7.84	16.47
Master Protein	P51148-2	Isoform 2 of Ras-related protein Rab-5C OS=Homo sapiens GN=RAB5C	RAB5C	High	FALSE	0.004	6.038	9	2	3	2	1	249	27	8.66	4.36
Master Protein	P11908-2	Isoform 2 of Ribose-phosphate pyrophosphokinase 2 OS=Homo sapiens GN=PRPS2	PRPS2	High	FALSE	0.007	3.87	4	1	6	1	1	321	35	6.46	14.32
Master Protein	Q86VB7-2	Isoform 2 of Scavenger receptor cysteine-rich type 1 protein M130 OS=Homo sapiens GN=CD163	CD163	High	FALSE	0	44.726	12	11	75	11	1	1161	125.9	6.14	149.24
Master Protein	Q9NVA2-2	Isoform 2 of Septin-11 OS=Homo sapiens GN=SEPT11	SEPT11	High	FALSE	0	11.095	7	3	23	2	1	439	50.8	7.01	51.62
Master Protein	Q15019-2	Isoform 2 of Septin-2 OS=Homo sapiens GN=SEPT2	SEPT2	High	FALSE	0.007	3.933	2	1	3	1	1	396	45.4	6.89	6.35
Master Protein	P31948-2	Isoform 2 of Stress-induced-phosphoprotein 1 OS=Homo sapiens GN=STIP1	STIP1	High	FALSE	0.007	4.165	2	1	1	1	1	590	68	7.74	2.84
Master Protein	P37802-2	Isoform 2 of Transgelin-2 OS=Homo sapiens GN=TAGLN2	TAGLN2	High	FALSE	0	98.553	66	13	526	13	1	220	24.4	8.25	1028.08
Master Protein	P29401-2	Isoform 2 of Transketolase OS=Homo sapiens GN=TKT	TKT	High	FALSE	0	32.61	17	7	43	7	1	631	68.8	7.52	99.9
Master Protein	P06753-2	Isoform 2 of Tropomyosin alpha-3 chain OS=Homo sapiens GN=TPM3	TPM3	High	FALSE	0	37.237	23	7	95	4	1	248	29	4.78	238.19
Master Protein	O75347-2	Isoform 2 of Tubulin-specific chaperone A OS=Homo sapiens GN=TBCA	TBCA	High	FALSE	0.008	3.401	7	1	5	1	1	129	15.8	9.35	6.15
Master Protein	P78324-2	Isoform 2 of Tyrosine-protein phosphatase non-receptor type substrate 1 OS=Homo sapiens GN=SIRPA	SIRPA	High	FALSE	0.001	7.031	5	2	6	2	1	508	55.4	6.98	13.86
Master Protein	Q96FW1-2	Isoform 2 of Ubiquitin thioesterase OTUB1 OS=Homo sapiens GN=OTUB1	OTUB1	High	FALSE	0.006	3.494	3	1	4	1	1	315	35.3	8.51	6.08
Master Protein	P04070-2	Isoform 2 of Vitamin K-dependent protein C OS=Homo sapiens GN=PROC	PROC	High	FALSE	0	47.744	21	8	215	8	1	516	57.5	6.68	422.84
Master Protein	P22891-2	Isoform 2 of Vitamin K-dependent protein Z OS=Homo sapiens GN=PROZ	PROZ	High	FALSE	0	42.209	25	8	132	8	1	422	47	6.04	332.33
Master Protein	P12110-2	Isoform 2C2A of Collagen alpha-2(VI) chain OS=Homo sapiens GN=COL6A2	COL6A2	High	FALSE	0.006	4.119	1	1	1	1	1	918	97.4	5.55	2.56
Master Protein	Q6UY14-3	Isoform 3 of ADAMTS-like protein 4 OS=Homo sapiens GN=ADAMTSL4	ADAMTSL4	High	FALSE	0.001	6.669	3	2	4	2	1	1097	118.7	8.34	9.05
Master Protein	P12814-3	Isoform 3 of Alpha-actinin-1 OS=Homo sapiens GN=ACTN1	ACTN1	High	FALSE	0	242.749	48	39	520	26	1	914	105.5	5.41	1348.75
Master Protein	Q9BY67-3	Isoform 3 of Cell adhesion molecule 1 OS=Homo sapiens GN=CADM1	CADM1	High	FALSE	0.006	4.038	3	1	7	1	1	471	51.5	4.87	17.31
Master Protein	Q9ULV4-3	Isoform 3 of Coronin-1C OS=Homo sapiens GN=CORO1C	CORO1C	High	FALSE	0	19.816	10	5	16	5	1	527	58.9	7.75	38.14
Master Protein	Q13822-3	Isoform 3 of Ectonucleotide pyrophosphatase/phosphodiesterase family member 2 OS=Homo sapiens GN=ENPP2	ENPP2	High	FALSE	0	30.559	10	6	49	6	1	888	101.9	7.59	92.34
Master Protein	Q93063-3	Isoform 3 of Exostosin-2 OS=Homo sapiens GN=EXT2	EXT2	High	FALSE	0	12.803	6	4	20	4	1	751	85.8	6.32	37.48
Master Protein	O15117-3	Isoform 3 of FYN-binding protein OS=Homo sapiens GN=FYB	FYB	High	FALSE	0.006	3.503	2	1	2	1	1	839	91.6	6.9	2.08
Master Protein	Q86SQ4-3	Isoform 3 of G-protein coupled receptor 126 OS=Homo sapiens GN=GPR126	GPR126	High	FALSE	0	11.894	3	3	23	3	1	1250	139.8	7.75	42.9
Master Protein	P19367-3	Isoform 3 of Hexokinase-1 OS=Homo sapiens GN=HK1	HK1	High	FALSE	0	8.873	4	3	5	3	1	921	102.7	6.76	8.52
Master Protein	P01344-3	Isoform 3 of Insulin-like growth factor II OS=Homo sapiens GN=IGF2	IGF2	High	FALSE	0	13.893	11	2	88	2	1	236	26.3	9.13	248.26
Master Protein	Q8N6C8-3	Isoform 3 of Leukocyte immunoglobulin-like receptor subfamily A member 3 OS=Homo sapiens GN=LILRA3	LILRA3	High	FALSE	0	10.604	8	3	11	3	1	456	49.2	8.09	26.95
Master Protein	O75023-3	Isoform 3 of Leukocyte immunoglobulin-like receptor subfamily B member 5 OS=Homo sapiens GN=LILRB5	LILRB5	High	FALSE	0.001	7.091	4	2	12	2	1	591	64.2	7.3	29.22
Master Protein	P48059-3	Isoform 3 of LIM and senescent cell antigen-like-containing domain protein 1 OS=Homo sapiens GN=LIMS1	LIMS1	High	FALSE	0	14.024	11	4	20	4	1	387	44.4	8.02	55.11
Master Protein	P00338-3	Isoform 3 of L-lactate dehydrogenase A chain OS=Homo sapiens GN=LDHA	LDHA	High	FALSE	0	41.159	28	9	139	8	1	361	39.8	8.43	303.07

Master Protein	P40925-3	Isoform 3 of Malate dehydrogenase, cytoplasmic OS=Homo sapiens GN=MDH1	MDH1	High	FALSE	0	21.125	13	4	48	4	1	352	38.6	7.71	133.77
Master Protein	P05155-3	Isoform 3 of Plasma protease C1 inhibitor OS=Homo sapiens GN=SERPING1	SERPING1	High	FALSE	0	261.574	40	27	11390	27	1	505	55.7	6.4	30453.02
Master Protein	Q9HCN6-3	Isoform 3 of Platelet glycoprotein VI OS=Homo sapiens GN=GP6	GP6	High	FALSE	0	12.153	5	3	10	3	1	620	67.4	8.79	21.24
Master Protein	Q70J99-3	Isoform 3 of Protein unc-13 homolog D OS=Homo sapiens GN=UNC13D	UNC13D	High	FALSE	0.001	6.386	2	2	2	2	1	1142	128.7	6.62	4.6
Master Protein	O43665-3	Isoform 3 of Regulator of G-protein signaling 10 OS=Homo sapiens GN=RGS10	RGS10	High	FALSE	0	8.328	14	2	10	2	1	181	21.2	7.49	18.58
Master Protein	Q15833-3	Isoform 3 of Syntaxin-binding protein 2 OS=Homo sapiens GN=STXBP2	STXBP2	High	FALSE	0	10.033	6	3	5	3	1	604	67.7	6.68	5.66
Master Protein	P61077-3	Isoform 3 of Ubiquitin-conjugating enzyme E2 D3 OS=Homo sapiens GN=UBE2D3	UBE2D3	High	FALSE	0.008	3.402	7	1	4	1	1	149	16.9	7.74	2.26
Master Protein	P02774-3	Isoform 3 of Vitamin D-binding protein OS=Homo sapiens GN=GC	GC	High	FALSE	0	427	82	52	14920	4	1	493	55.1	5.74	41140
Master Protein	P08195-4	Isoform 4 of 4F2 cell-surface antigen heavy chain OS=Homo sapiens GN=SLC3A2	SLC3A2	High	FALSE	0	40.817	18	10	50	10	1	661	71.1	4.97	112.41
Master Protein	P58335-4	Isoform 4 of Anthrax toxin receptor 2 OS=Homo sapiens GN=ANTXR2	ANTXR2	High	FALSE	0	18.659	11	5	21	5	1	488	53.5	8.27	41.53
Master Protein	P55290-4	Isoform 4 of Cadherin-13 OS=Homo sapiens GN=CDH13	CDH13	High	FALSE	0	16.942	5	3	28	3	1	760	83.3	5.12	67.23
Master Protein	Q13642-4	Isoform 4 of Four and a half LIM domains protein 1 OS=Homo sapiens GN=FHL1	FHL1	High	FALSE	0.005	4.754	6	2	6	2	1	309	35	8.32	9.09
Master Protein	Q9NPH3-5	Isoform 4 of Interleukin-1 receptor accessory protein OS=Homo sapiens GN=IL1RAP	IL1RAP	High	FALSE	0	27.268	10	8	99	8	1	687	78.6	7.12	237.96
Master Protein	Q13228-4	Isoform 4 of Selenium-binding protein 1 OS=Homo sapiens GN=SELENBP1	SELENBP1	High	FALSE	0	53.076	32	13	107	13	1	514	56.8	6.48	219
Master Protein	P29350-4	Isoform 4 of Tyrosine-protein phosphatase non-receptor type 6 OS=Homo sapiens GN=PTPN6	PTPN6	High	FALSE	0	17.588	8	5	18	5	1	624	70.1	7.9	39.34
Master Protein	P19021-5	Isoform 5 of Peptidyl-glycine alpha-amidating monooxygenase OS=Homo sapiens GN=PAM	PAM	High	FALSE	0	14.731	5	4	25	4	1	974	108.3	6.42	46.23
Master Protein	O00429-6	Isoform 6 of Dynamin-1-like protein OS=Homo sapiens GN=DNM1L	DNM1L	High	FALSE	0.001	7.477	3	2	4	2	1	749	83.3	7.08	8.04
Master Protein	Q9H2X3-7	Isoform 7 of C-type lectin domain family 4 member M OS=Homo sapiens GN=CLEC4M	CLEC4M	High	FALSE	0.006	3.478	3	1	3	1	1	263	30.1	5.26	5.96
Master Protein	O43399-7	Isoform 7 of Tumor protein D54 OS=Homo sapiens GN=TPD52L2	TPD52L2	High	FALSE	0.007	3.859	5	1	2	1	1	229	24.8	6.42	2.02
Master Protein	P09493-8	Isoform 8 of Tropomyosin alpha-1 chain OS=Homo sapiens GN=TPM1	TPM1	High	FALSE	0	20.693	17	6	74	1	1	284	32.8	4.75	163.18
Master Protein	O60888-2	Isoform A of Protein CutA OS=Homo sapiens GN=CUTA	CUTA	High	FALSE	0.005	4.608	7	1	4	1	1	198	20.9	5.48	10.48
Master Protein	P20023-3	Isoform C of Complement receptor type 2 OS=Homo sapiens GN=CR2	CR2	High	FALSE	0	18.009	6	6	21	6	1	1092	119.1	7.52	35.36
Master Protein	P02679-2	Isoform Gamma-A of Fibrinogen gamma chain OS=Homo sapiens GN=FGG	FGG	High	FALSE	0	406.394	73	42	20989	1	1	437	49.5	6.09	63074.82
Master Protein	P05164-3	Isoform H7 of Myeloperoxidase OS=Homo sapiens GN=MPO	MPO	High	FALSE	0	18.055	7	5	14	5	1	777	87.2	9.07	26.85
Master Protein	P13473-3	Isoform LAMP-2C of Lysosome-associated membrane glycoprotein 2 OS=Homo sapiens GN=LAMP2	LAMP2	High	FALSE	0	12.091	7	3	56	3	1	411	45.1	5.91	132.76
Master Protein	P01042-2	Isoform LMW of Kininogen-1 OS=Homo sapiens GN=KNG1	KNG1	High	FALSE	0	299.417	75	40	6006	4	1	427	47.9	6.65	15367.8
Master Protein	P11413-2	Isoform Long of Glucose-6-phosphate 1-dehydrogenase OS=Homo sapiens GN=G6PD	G6PD	High	FALSE	0.004	5.759	3	2	6	2	1	561	63.8	6.89	5.31
Master Protein	P25786-2	Isoform Long of Proteasome subunit alpha type-1 OS=Homo sapiens GN=PSMA1	PSMA1	High	FALSE	0	7.789	10	3	7	3	1	269	30.2	6.99	13.24
Master Protein	B7ZKJ8	ITIH4 protein OS=Homo sapiens GN=ITIH4 PE=1 SV=1	ITIH4	High	FALSE	0	409.168	50	47	12107	1	1	935	103.8	6.89	33635.97
Master Protein	P29622	Kallistatin OS=Homo sapiens GN=SERPINA4 PE=1 SV=3	SERPINA4	High	FALSE	0	147.879	48	20	4513	20	1	427	48.5	7.75	9965.58
Master Protein	P13645	Keratin, type I cytoskeletal 10 OS=Homo sapiens GN=KRT10 PE=1 SV=6	KRT10	High	FALSE	0	151.878	52	24	940	22	1	584	58.8	5.21	2313.86
Master Protein	P02533	Keratin, type I cytoskeletal 14 OS=Homo sapiens GN=KRT14 PE=1 SV=4	KRT14	High	FALSE	0	58.025	35	14	208	7	1	472	51.5	5.16	457.54
Master Protein	P35527	Keratin, type I cytoskeletal 9 OS=Homo sapiens GN=KRT9 PE=1 SV=3	KRT9	High	FALSE	0	210.064	65	29	586	29	1	623	62	5.24	1489.34
Master Protein	Q9NSB4	Keratin, type II cuticular Hb2 OS=Homo sapiens GN=KRT82 PE=1 SV=3	KRT82	High	FALSE	0.006	3.674	2	1	1	1	1	513	56.6	6.74	3.38

Master Protein	P04264	Keratin, type II cytoskeletal 1 OS=Homo sapiens GN=KRT1 PE=1 SV=6	KRT1	High	FALSE	0	182.43	59	31	941	27	1	644	66	8.12	2411
Master Protein	P35908	Keratin, type II cytoskeletal 2 epidermal OS=Homo sapiens GN=KRT2 PE=1 SV=2	KRT2	High	FALSE	0	132.793	54	28	532	23	1	639	65.4	8	1174.23
Master Protein	O95678	Keratin, type II cytoskeletal 75 OS=Homo sapiens GN=KRT75 PE=1 SV=2	KRT75	High	FALSE	0	20.609	11	6	99	1	1	551	59.5	7.74	234.57
Master Protein	O75037	Kinesin-like protein KIF21B OS=Homo sapiens GN=KIF21B PE=1 SV=2	KIF21B	High	FALSE	0.007	3.809	1	2	22	1	1	1637	182.5	7.08	23.33
Master Protein	P01042	Kininogen-1 OS=Homo sapiens GN=KNG1 PE=1 SV=2	KNG1	High	FALSE	0	293.068	53	40	5711	4	1	644	71.9	6.81	14298.11
Master Protein	P02788	Lactotransferrin OS=Homo sapiens GN=LTF PE=1 SV=6	LTF	High	FALSE	0	35.795	17	10	50	10	1	710	78.1	8.12	95.48
Master Protein	Q04760	Lactoylglutathione lyase OS=Homo sapiens GN=GLO1 PE=1 SV=4	GLO1	High	FALSE	0.001	6.632	14	2	5	2	1	184	20.8	5.31	11.1
Master Protein	P24043	Laminin subunit alpha-2 OS=Homo sapiens GN=LAMA2 PE=1 SV=4	LAMA2	High	FALSE	0.001	6.859	1	2	11	2	1	3122	343.7	6.4	23.19
Master Protein	G3XAI2	Laminin subunit beta-1 OS=Homo sapiens GN=LAMB1 PE=1 SV=1	LAMB1	High	FALSE	0	30.113	5	9	38	9	1	1810	200.3	4.96	81.39
Master Protein	P55268	Laminin subunit beta-2 OS=Homo sapiens GN=LAMB2 PE=1 SV=2	LAMB2	High	FALSE	0.008	3.416	1	1	3	1	1	1798	195.9	6.52	6.43
Master Protein	P11047	Laminin subunit gamma-1 OS=Homo sapiens GN=LAMC1 PE=1 SV=3	LAMC1	High	FALSE	0.004	5.17	1	1	3	1	1	1609	177.5	5.12	6.91
Master Protein	Q9BS40	Latexin OS=Homo sapiens GN=LXN PE=1 SV=2	LXN	High	FALSE	0.006	3.598	4	1	1	1	1	222	25.7	5.78	1.68
Master Protein	P02750	Leucine-rich alpha-2-glycoprotein OS=Homo sapiens GN=LRG1 PE=1 SV=2	LRG1	High	FALSE	0	67.188	39	11	1448	11	1	347	38.2	6.95	4143.51
Master Protein	O14960	Leukocyte cell-derived chemotaxin-2 OS=Homo sapiens GN=LECT2 PE=1 SV=2	LECT2	High	FALSE	0.007	3.885	6	1	21	1	1	151	16.4	9.39	55.5
Master Protein	P30740	Leukocyte elastase inhibitor OS=Homo sapiens GN=SERPINB1 PE=1 SV=1	SERPINB1	High	FALSE	0	31.819	24	8	38	8	1	379	42.7	6.28	84.71
Master Protein	P09960	Leukotriene A-4 hydrolase OS=Homo sapiens GN=LTA4H PE=1 SV=2	LTA4H	High	FALSE	0	20.545	11	5	9	5	1	611	69.2	6.18	15.66
Master Protein	H3BLU2	Limbic system-associated membrane protein (Fragment) OS=Homo sapiens GN=LSAMP PE=4 SV=1	LSAMP	High	FALSE	0.004	6.115	8	2	7	2	1	345	38	6.86	8.06
Master Protein	P18428	Lipopolysaccharide-binding protein OS=Homo sapiens GN=LBP PE=1 SV=3	LBP	High	FALSE	0	83.771	30	10	530	10	1	481	53.4	6.7	1226.05
Master Protein	P05451	Lithostathine-1-alpha OS=Homo sapiens GN=REG1A PE=1 SV=3	REG1A	High	FALSE	0.006	4.118	7	1	8	1	1	166	18.7	5.94	5.84
Master Protein	P07195	L-lactate dehydrogenase B chain OS=Homo sapiens GN=LDHB PE=1 SV=2	LDHB	High	FALSE	0	67.553	39	11	325	10	1	334	36.6	6.05	745.14
Master Protein	M9MML6	Low affinity immunoglobulin gamma Fc region receptor III-B OS=Homo sapiens GN=FCGR3B PE=4 SV=1	FCGR3B	High	FALSE	0.001	6.51	9	2	11	2	1	269	29.9	6.92	17.47
Master Protein	P24666	Low molecular weight phosphotyrosine protein phosphatase OS=Homo sapiens GN=ACP1 PE=1 SV=3	ACP1	High	FALSE	0	8.194	17	2	8	2	1	158	18	6.74	20.12
Master Protein	H0YMD1	Low-density lipoprotein receptor OS=Homo sapiens GN=LDLR PE=1 SV=1	LDLR	High	FALSE	0.006	4.134	1	1	1	1	1	948	104.7	5.5	3.35
Master Protein	P51884	Lumican OS=Homo sapiens GN=LUM PE=1 SV=2	LUM	High	FALSE	0	116.257	43	15	2225	15	1	338	38.4	6.61	5122.94
Master Protein	Q9Y5Y7	Lymphatic vessel endothelial hyaluronic acid receptor 1 OS=Homo sapiens GN=LYVE1 PE=1 SV=2	LYVE1	High	FALSE	0	28.094	15	7	153	7	1	322	35.2	8.28	314.91
Master Protein	P19256	Lymphocyte function-associated antigen 3 OS=Homo sapiens GN=CD58 PE=1 SV=1	CD58	High	FALSE	0.006	3.969	4	1	9	1	1	250	28.1	6.76	21.59
Master Protein	P11279	Lysosome-associated membrane glycoprotein 1 OS=Homo sapiens GN=LAMP1 PE=1 SV=3	LAMP1	High	FALSE	0.004	5.554	4	2	15	2	1	417	44.9	8.75	30.53
Master Protein	P61626	Lysozyme C OS=Homo sapiens GN=LYZ PE=1 SV=1	LYZ	High	FALSE	0	27.65	45	5	115	5	1	148	16.5	9.16	223.26
Master Protein	P07333	Macrophage colony-stimulating factor 1 receptor OS=Homo sapiens GN=CSF1R PE=1 SV=2	CSF1R	High	FALSE	0	33.257	8	5	111	5	1	972	107.9	6.37	263.35
Master Protein	P22897	Macrophage mannose receptor 1 OS=Homo sapiens GN=MRC1 PE=1 SV=1	MRC1	High	FALSE	0	27.756	6	8	45	8	1	1456	165.9	6.54	80.63
Master Protein	P14174	Macrophage migration inhibitory factor OS=Homo sapiens GN=MIF PE=1 SV=4	MIF	High	FALSE	0.006	3.632	8	1	9	1	1	115	12.5	7.88	22.72
Master Protein	P48740	Mannan-binding lectin serine protease 1 OS=Homo sapiens GN=MASP1 PE=1 SV=3	MASP1	High	FALSE	0	90.132	29	16	501	5	1	699	79.2	5.49	1446.09
Master Protein	O00187	Mannan-binding lectin serine protease 2 OS=Homo sapiens GN=MASP2 PE=1 SV=4	MASP2	High	FALSE	0	53.908	21	11	258	11	1	686	75.7	5.63	705.77

Master Protein	P11226	Mannose-binding protein C OS=Homo sapiens GN=MBL2 PE=1 SV=2	MBL2	High	FALSE	0	34.083	35	8	376	8	1	248	26.1	5.49	859.67
Master Protein	P33908	Mannosyl-oligosaccharide 1,2-alpha-mannosidase IA OS=Homo sapiens GN=MAN1A1 PE=1 SV=3	MAN1A1	High	FALSE	0	34.67	10	6	156	6	1	653	72.9	6.47	375.59
Master Protein	P10721	Mast/stem cell growth factor receptor Kit OS=Homo sapiens GN=KIT PE=1 SV=1	KIT	High	FALSE	0	12.011	3	3	5	3	1	976	109.8	6.98	11.79
Master Protein	P14780	Matrix metalloproteinase-9 OS=Homo sapiens GN=MMP9 PE=1 SV=3	MMP9	High	FALSE	0	15.418	7	4	13	4	1	707	78.4	6.06	32.01
Master Protein	Q16853	Membrane primary amine oxidase OS=Homo sapiens GN=AOC3 PE=1 SV=3	AOC3	High	FALSE	0	25.711	7	5	56	5	1	763	84.6	6.52	132.25
Master Protein	P55145	Mesencephalic astrocyte-derived neurotrophic factor OS=Homo sapiens GN=MANF PE=1 SV=3	MANF	High	FALSE	0	16.017	23	4	11	4	1	182	20.7	8.69	25.68
Master Protein	P01033	Metalloproteinase inhibitor 1 OS=Homo sapiens GN=TIMP1 PE=1 SV=1	TIMP1	High	FALSE	0	16.23	25	4	35	4	1	207	23.2	8.1	84.76
Master Protein	P16035	Metalloproteinase inhibitor 2 OS=Homo sapiens GN=TIMP2 PE=1 SV=2	TIMP2	High	FALSE	0.001	7.217	11	3	13	3	1	220	24.4	7.49	14.36
Master Protein	Q15691	Microtubule-associated protein RP/EB family member 1 OS=Homo sapiens GN=MAPRE1 PE=1 SV=3	MAPRE1	High	FALSE	0	7.825	10	2	6	2	1	268	30	5.14	15.43
Master Protein	P20774	Mimecan OS=Homo sapiens GN=OGN PE=1 SV=1	OGN	High	FALSE	0	11.175	11	4	27	4	1	298	33.9	5.63	52.23
Master Protein	P26038	Moesin OS=Homo sapiens GN=MSN PE=1 SV=3	MSN	High	FALSE	0	80.571	34	20	192	20	1	577	67.8	6.4	397.25
Master Protein	P08571	Monocyte differentiation antigen CD14 OS=Homo sapiens GN=CD14 PE=1 SV=2	CD14	High	FALSE	0	97.267	33	10	912	10	1	375	40.1	6.23	2396.32
Master Protein	Q99685	Monoglyceride lipase OS=Homo sapiens GN=MGLL PE=1 SV=2	MGLL	High	FALSE	0.006	3.542	5	1	1	1	1	303	33.2	6.99	2.2
Master Protein	A0A087WXC9	Mucosal addressin cell adhesion molecule 1 OS=Homo sapiens GN=MADCAM1 PE=4 SV=1	MADCAM1	High	FALSE	0.004	5.41	4	1	6	1	1	406	42.4	5.07	21.02
Master Protein	Q13201	Multimerin-1 OS=Homo sapiens GN=MMRN1 PE=1 SV=3	MMRN1	High	FALSE	0	15.59	4	4	19	4	1	1228	138	7.93	37.08
Master Protein	Q9H8L6	Multimerin-2 OS=Homo sapiens GN=MMRN2 PE=1 SV=2	MMRN2	High	FALSE	0.007	3.742	1	1	19	1	1	949	104.3	5.86	41.9
Master Protein	Q7Z7M0	Multiple epidermal growth factor-like domains protein 8 OS=Homo sapiens GN=MEGF8 PE=1 SV=2	MEGF8	High	FALSE	0	40.593	6	12	78	12	1	2845	302.9	6.87	157.96
Master Protein	Q9UNW1	Multiple inositol polyphosphate phosphatase 1 OS=Homo sapiens GN=MINPP1 PE=1 SV=1	MINPP1	High	FALSE	0	36.62	20	8	109	8	1	487	55	7.81	261.85
Master Protein	Q99972	Myocilin OS=Homo sapiens GN=MYOC PE=1 SV=2	MYOC	High	FALSE	0	9.345	6	3	30	3	1	504	56.9	5.54	49.38
Master Protein	P02144	Myoglobin OS=Homo sapiens GN=MB PE=1 SV=2	MB	High	FALSE	0	8.575	19	2	13	2	1	154	17.2	7.68	45.66
Master Protein	Q15746	Myosin light chain kinase, smooth muscle OS=Homo sapiens GN=MYLK PE=1 SV=4	MYLK	High	FALSE	0	13.221	2	4	10	4	1	1914	210.6	6.15	11.3
Master Protein	Q9UKX2	Myosin-2 OS=Homo sapiens GN=MYH2 PE=1 SV=1	MYH2	High	FALSE	0	12.568	2	3	5	3	1	1941	222.9	5.82	7.16
Master Protein	P58546	Myotrophin OS=Homo sapiens GN=MTPN PE=1 SV=2	MTPN	High	FALSE	0.004	5.774	14	1	15	1	1	118	12.9	5.52	43.33
Master Protein	P29966	Myristoylated alanine-rich C-kinase substrate OS=Homo sapiens GN=MARCKS PE=1 SV=4	MARCKS	High	FALSE	0	9.037	11	2	2	2	1	332	31.5	4.45	6.82
Master Protein	P20933	N(4)-(beta-N-acetylglucosaminyl)-L-asparaginase OS=Homo sapiens GN=AGA PE=1 SV=2	AGA	High	FALSE	0.006	4.41	3	1	14	1	1	346	37.2	6.28	30.21
Master Protein	O14745	Na(+)/H(+) exchange regulatory cofactor NHE-RF1 OS=Homo sapiens GN=SLC9A3R1 PE=1 SV=4	SLC9A3R1	High	FALSE	0	10.154	8	2	15	2	1	358	38.8	5.77	32.31
Master Protein	Q9UJJ9	N-acetylglucosamine-1-phosphotransferase subunit gamma OS=Homo sapiens GN=GNPTG PE=1 SV=1	GNPTG	High	FALSE	0	12.886	10	2	21	2	1	305	34	6.95	44.74
Master Protein	O43505	N-acetyllactosaminide beta-1,3-N-acetylglucosaminyltransferase OS=Homo sapiens GN=B3GNT1 PE=1 SV=1	B3GNT1	High	FALSE	0	12.016	9	4	29	4	1	415	47.1	7.2	57.46
Master Protein	Q8NCW5	NAD(P)H-hydrate epimerase OS=Homo sapiens GN=APOA1BP PE=1 SV=2	APOA1BP	High	FALSE	0.006	4.051	7	1	5	1	1	288	31.7	7.66	5.91
Master Protein	Q92859	Neogenin OS=Homo sapiens GN=NEO1 PE=1 SV=2	NEO1	High	FALSE	0	29.514	7	8	57	8	1	1461	159.9	6.54	116.56
Master Protein	P13591	Neural cell adhesion molecule 1 OS=Homo sapiens GN=NCAM1 PE=1 SV=3	NCAM1	High	FALSE	0	55.588	22	13	114	13	1	858	94.5	4.87	216.46
Master Protein	O15394	Neural cell adhesion molecule 2 OS=Homo sapiens GN=NCAM2 PE=1 SV=2	NCAM2	High	FALSE	0	18.742	8	6	29	6	1	837	93	5.6	60.97

Master Protein	P32004	Neural cell adhesion molecule L1 OS=Homo sapiens GN=L1CAM PE=1 SV=2	L1CAM	High	FALSE	0.007	4.288	1	1	3	1	1	1257	139.9	6.24	8.81
Master Protein	Q9UM47	Neurogenic locus notch homolog protein 3 OS=Homo sapiens GN=NOTCH3 PE=1 SV=2	NOTCH3	High	FALSE	0.004	6.235	1	2	9	2	1	2321	243.5	5.39	20.15
Master Protein	Q7Z3B1	Neuronal growth regulator 1 OS=Homo sapiens GN=NEGR1 PE=1 SV=3	NEGR1	High	FALSE	0.005	4.843	4	1	8	1	1	354	38.7	6.21	19.4
Master Protein	O14786	Neuropilin-1 OS=Homo sapiens GN=NRP1 PE=1 SV=3	NRP1	High	FALSE	0	22.143	6	5	72	5	1	923	103.1	5.88	125.73
Master Protein	O60462	Neuropilin-2 OS=Homo sapiens GN=NRP2 PE=1 SV=2	NRP2	High	FALSE	0.007	4.227	1	1	2	1	1	931	104.8	5.17	5.18
Master Protein	P59665	Neutrophil defensin 1 OS=Homo sapiens GN=DEFA1 PE=1 SV=1	DEFA1	High	FALSE	0.001	7.456	19	2	38	2	1	94	10.2	6.99	102.09
Master Protein	X6R8F3	Neutrophil gelatinase-associated lipocalin OS=Homo sapiens GN=LCN2 PE=1 SV=1	LCN2	High	FALSE	0	17.263	22	3	37	3	1	200	22.8	8.5	85.64
Master Protein	P14543	Nidogen-1 OS=Homo sapiens GN=NID1 PE=1 SV=3	NID1	High	FALSE	0	24.11	6	6	35	6	1	1247	136.3	5.29	78.68
Master Protein	A0A087X117	Nodal modulator 1 OS=Homo sapiens GN=NOMO1 PE=4 SV=1	NOMO1	High	FALSE	0.007	4.19	1	1	1	1	1	1267	139.4	5.85	2.4
Master Protein	Q99784	Noelin OS=Homo sapiens GN=OLFM1 PE=1 SV=4	OLFM1	High	FALSE	0	16.808	9	3	23	3	1	485	55.3	6.95	50.62
Master Protein	O95897	Noelin-2 OS=Homo sapiens GN=OLFM2 PE=2 SV=2	OLFM2	High	FALSE	0.006	3.537	3	1	5	1	1	454	51.4	7.94	4.97
Master Protein	Q13232	Nucleoside diphosphate kinase 3 OS=Homo sapiens GN=NME3 PE=1 SV=2	NME3	High	FALSE	0	8.444	16	2	10	2	1	169	19	7.84	25.89
Master Protein	P55209	Nucleosome assembly protein 1-like 1 OS=Homo sapiens GN=NAP1L1 PE=1 SV=1	NAP1L1	High	FALSE	0	15.584	14	4	11	3	1	391	45.3	4.46	26
Master Protein	J3KQ32	Obg-like ATPase 1 OS=Homo sapiens GN=OLA1 PE=1 SV=1	OLA1	High	FALSE	0.007	4.217	3	1	5	1	1	416	46.9	8.06	11.81
Master Protein	Q8WWZ8	Oncoprotein-induced transcript 3 protein OS=Homo sapiens GN=OIT3 PE=1 SV=2	OIT3	High	FALSE	0	10.519	6	3	16	3	1	545	60	5.58	32.62
Master Protein	Q99650	Oncostatin-M-specific receptor subunit beta OS=Homo sapiens GN=OSMR PE=1 SV=1	OSMR	High	FALSE	0.006	3.626	1	1	19	1	1	979	110.4	5.82	32.46
Master Protein	Q86UD1	Out at first protein homolog OS=Homo sapiens GN=OAF PE=2 SV=1	OAF	High	FALSE	0	13.21	17	4	40	4	1	273	30.7	6.84	79.01
Master Protein	P04746	Pancreatic alpha-amylase OS=Homo sapiens GN=AMY2A PE=1 SV=2	AMY2A	High	FALSE	0	10.054	5	2	12	1	1	511	57.7	7.05	27.06
Master Protein	O95497	Pantetheinase OS=Homo sapiens GN=VNN1 PE=1 SV=2	VNN1	High	FALSE	0	34.553	15	6	78	6	1	513	57	5.55	191.02
Master Protein	O00151	PDZ and LIM domain protein 1 OS=Homo sapiens GN=PDLIM1 PE=1 SV=4	PDLIM1	High	FALSE	0	18.771	19	5	36	5	1	329	36	7.02	67.17
Master Protein	Q6UXB8	Peptidase inhibitor 16 OS=Homo sapiens GN=PI16 PE=1 SV=1	PI16	High	FALSE	0	30.13	9	4	68	4	1	463	49.4	5.39	193.7
Master Protein	P62937	Peptidyl-prolyl cis-trans isomerase A OS=Homo sapiens GN=PPIA PE=1 SV=2	PPIA	High	FALSE	0	27.523	30	5	175	5	1	165	18	7.81	330.56
Master Protein	P23284	Peptidyl-prolyl cis-trans isomerase B OS=Homo sapiens GN=PPIB PE=1 SV=2	PPIB	High	FALSE	0	22.267	25	5	32	5	1	216	23.7	9.41	74.85
Master Protein	Q15063	Periostin OS=Homo sapiens GN=POSTN PE=1 SV=2	POSTN	High	FALSE	0	68.221	22	15	113	1	1	836	93.3	7.53	263.21
Master Protein	B1ALD9	Periostin OS=Homo sapiens GN=POSTN PE=4 SV=1	POSTN	High	FALSE	0	68.191	23	15	117	1	1	808	90.1	7.94	272.85
Master Protein	Q06830	Peroxiredoxin-1 OS=Homo sapiens GN=PRDX1 PE=1 SV=1	PRDX1	High	FALSE	0	26.071	38	7	54	6	1	199	22.1	8.13	123.45
Master Protein	P32119	Peroxiredoxin-2 OS=Homo sapiens GN=PRDX2 PE=1 SV=5	PRDX2	High	FALSE	0	48.47	51	9	325	8	1	198	21.9	5.97	886.25
Master Protein	P30044	Peroxiredoxin-5, mitochondrial OS=Homo sapiens GN=PRDX5 PE=1 SV=4	PRDX5	High	FALSE	0	10.82	17	3	15	3	1	214	22.1	8.7	36.06
Master Protein	P30041	Peroxiredoxin-6 OS=Homo sapiens GN=PRDX6 PE=1 SV=3	PRDX6	High	FALSE	0	30.315	33	8	79	8	1	224	25	6.38	182.93
Master Protein	P04180	Phosphatidylcholine-sterol acyltransferase OS=Homo sapiens GN=LCAT PE=1 SV=1	LCAT	High	FALSE	0	52.21	30	9	709	9	1	440	49.5	6.11	1575.15
Master Protein	P30086	Phosphatidylethanolamine-binding protein 1 OS=Homo sapiens GN=PEBP1 PE=1 SV=3	PEBP1	High	FALSE	0	10.263	23	3	7	3	1	187	21	7.53	11.39
Master Protein	Q96S96	Phosphatidylethanolamine-binding protein 4 OS=Homo sapiens GN=PEBP4 PE=1 SV=3	PEBP4	High	FALSE	0.004	5.198	7	2	22	2	1	227	25.7	6.54	22.84
Master Protein	P80108	Phosphatidylinositol-glycan-specific phospholipase D OS=Homo sapiens GN=GPLD1 PE=1 SV=3	GPLD1	High	FALSE	0	128.707	30	24	1978	24	1	840	92.3	6.37	4997.7
Master Protein	P36871	Phosphoglucomutase-1 OS=Homo sapiens GN=PGM1 PE=1 SV=3	PGM1	High	FALSE	0	29.888	16	7	29	7	1	562	61.4	6.76	80.46
Master Protein	Q96G03	Phosphoglucomutase-2 OS=Homo sapiens GN=PGM2 PE=1 SV=4	PGM2	High	FALSE	0.007	3.763	2	1	2	1	1	612	68.2	6.73	4.01

Master Protein	P00558	Phosphoglycerate kinase 1 OS=Homo sapiens GN=PGK1 PE=1 SV=3	PGK1	High	FALSE	0	75.218	42	14	118	14	1	417	44.6	8.1	275.48
Master Protein	P18669	Phosphoglycerate mutase 1 OS=Homo sapiens GN=PGAM1 PE=1 SV=2	PGAM1	High	FALSE	0	18.641	27	5	26	5	1	254	28.8	7.18	38.88
Master Protein	F5H2B5	Phospholipase D4 OS=Homo sapiens GN=PLD4 PE=4 SV=1	PLD4	High	FALSE	0.006	3.974	2	1	5	1	1	513	56.3	8.28	13.88
Master Protein	P55058	Phospholipid transfer protein OS=Homo sapiens GN=PLTP PE=1 SV=1	PLTP	High	FALSE	0	102.397	33	12	478	12	1	493	54.7	7.01	1127.78
Master Protein	P36955	Pigment epithelium-derived factor OS=Homo sapiens GN=SERPINF1 PE=1 SV=4	SERPINF1	High	FALSE	0	173.491	56	26	3602	26	1	418	46.3	6.38	9275.57
Master Protein	Q9BTY2	Plasma alpha-L-fucosidase OS=Homo sapiens GN=FUCA2 PE=1 SV=2	FUCA2	High	FALSE	0	23.498	15	7	63	7	1	467	54	6.25	128.1
Master Protein	H0YAC1	Plasma kallikrein heavy chain (Fragment) OS=Homo sapiens GN=KLKB1 PE=3 SV=1	KLKB1	High	FALSE	0	207.541	50	32	3243	32	1	686	76.8	8.06	7854.36
Master Protein	P05154	Plasma serine protease inhibitor OS=Homo sapiens GN=SERPINA5 PE=1 SV=3	SERPINA5	High	FALSE	0	79.298	34	13	1008	13	1	406	45.6	9.26	2830.48
Master Protein	P05121	Plasminogen activator inhibitor 1 OS=Homo sapiens GN=SERPINE1 PE=1 SV=1	SERPINE1	High	FALSE	0.006	4.096	2	1	14	1	1	402	45	7.2	32.4
Master Protein	P00747	Plasminogen OS=Homo sapiens GN=PLG PE=1 SV=2	PLG	High	FALSE	0	521.025	70	56	9053	56	1	810	90.5	7.24	24584.82
Master Protein	P13796	Plastin-2 OS=Homo sapiens GN=LCP1 PE=1 SV=6	LCP1	High	FALSE	0	112.246	47	22	315	18	1	627	70.2	5.43	712.35
Master Protein	P13797	Plastin-3 OS=Homo sapiens GN=PLS3 PE=1 SV=4	PLS3	High	FALSE	0	21.054	8	5	69	1	1	630	70.8	5.6	165.65
Master Protein	P02775	Platelet basic protein OS=Homo sapiens GN=PPBP PE=1 SV=3	PPBP	High	FALSE	0	43.506	45	7	526	7	1	128	13.9	8.79	1350.71
Master Protein	P02776	Platelet factor 4 OS=Homo sapiens GN=PF4 PE=1 SV=2	PF4	High	FALSE	0	28.484	36	4	426	2	1	101	10.8	8.62	1382.63
Master Protein	P10720	Platelet factor 4 variant OS=Homo sapiens GN=PF4V1 PE=1 SV=1	PF4V1	High	FALSE	0	20.477	48	4	374	2	1	104	11.5	9.1	1247.22
Master Protein	P07359	Platelet glycoprotein Ib alpha chain OS=Homo sapiens GN=GP1BA PE=1 SV=2	GP1BA	High	FALSE	0	18.527	8	5	118	5	1	652	71.5	6.29	309.53
Master Protein	P40197	Platelet glycoprotein V OS=Homo sapiens GN=GP5 PE=1 SV=1	GP5	High	FALSE	0	19.373	10	5	31	5	1	560	60.9	9.63	78.12
Master Protein	Q13093	Platelet-activating factor acetylhydrolase OS=Homo sapiens GN=PLA2G7 PE=1 SV=1	PLA2G7	High	FALSE	0	27.012	17	7	60	7	1	441	50	7.56	130.52
Master Protein	P08567	Pleckstrin OS=Homo sapiens GN=PLEK PE=1 SV=3	PLEK	High	FALSE	0	28.665	22	4	45	4	1	350	40.1	8.28	83.77
Master Protein	Q8IUK5	Plexin domain-containing protein 1 OS=Homo sapiens GN=PLXDC1 PE=1 SV=2	PLXDC1	High	FALSE	0.005	4.69	2	1	7	1	1	500	55.7	5.85	6.47
Master Protein	Q6UX71	Plexin domain-containing protein 2 OS=Homo sapiens GN=PLXDC2 PE=1 SV=1	PLXDC2	High	FALSE	0	31.431	15	6	78	6	1	529	59.5	6.46	169.42
Master Protein	O43157	Plexin-B1 OS=Homo sapiens GN=PLXNB1 PE=1 SV=3	PLXNB1	High	FALSE	0.004	4.988	1	1	2	1	1	2135	232.2	5.49	6.84
Master Protein	O15031	Plexin-B2 OS=Homo sapiens GN=PLXNB2 PE=1 SV=3	PLXNB2	High	FALSE	0.004	6.193	2	2	5	2	1	1838	205	6.24	11.52
Master Protein	O00592	Podocalyxin OS=Homo sapiens GN=PODXL PE=1 SV=2	PODXL	High	FALSE	0	13.873	7	4	18	4	1	558	58.6	5.49	39.4
Master Protein	P15151	Poliovirus receptor OS=Homo sapiens GN=PVR PE=1 SV=2	PVR	High	FALSE	0	23.069	13	4	66	4	1	417	45.3	6.52	140.92
Master Protein	Q15365	Poly(rC)-binding protein 1 OS=Homo sapiens GN=PCBP1 PE=1 SV=2	PCBP1	High	FALSE	0	14.826	12	4	15	4	1	356	37.5	7.09	28.27
Master Protein	P0CG39	POTE ankyrin domain family member J OS=Homo sapiens GN=POTEJ PE=3 SV=1	POTEJ	High	FALSE	0	27.503	6	5	270	1	1	1038	117.3	5.97	817.49
Master Protein	P20742	Pregnancy zone protein OS=Homo sapiens GN=PZP PE=1 SV=4	PZP	High	FALSE	0	345.477	39	47	14319	36	1	1482	163.8	6.38	44702.33
Master Protein	Q9UHG3	Prenylcysteine oxidase 1 OS=Homo sapiens GN=PCYOX1 PE=1 SV=3	PCYOX1	High	FALSE	0	90.384	43	16	452	16	1	505	56.6	6.18	1052.42
Master Protein	Q6GTS8	Probable carboxypeptidase PM20D1 OS=Homo sapiens GN=PM20D1 PE=2 SV=3	PM20D1	High	FALSE	0.001	7.523	7	2	4	2	1	502	55.7	6.67	9.01
Master Protein	Q8IZF2	Probable G-protein coupled receptor 116 OS=Homo sapiens GN=GPR116 PE=1 SV=3	GPR116	High	FALSE	0	28.891	6	6	23	6	1	1346	149.4	6.65	38.2
Master Protein	Q15113	Procollagen C-endopeptidase enhancer 1 OS=Homo sapiens GN=PCOLCE PE=1 SV=2	PCOLCE	High	FALSE	0	30.685	23	7	113	7	1	449	47.9	7.43	282.62
Master Protein	P07737	Profilin-1 OS=Homo sapiens GN=PFN1 PE=1 SV=2	PFN1	High	FALSE	0	57.487	66	9	531	9	1	140	15	8.27	1348.59
Master Protein	P12273	Prolactin-inducible protein OS=Homo sapiens GN=PIP PE=1 SV=1	PIP	High	FALSE	0.006	3.715	10	1	3	1	1	146	16.6	8.05	4.15
Master Protein	H7BY64	Proline-rich acidic protein 1 OS=Homo sapiens GN=PRAP1 PE=4 SV=1	PRAP1	High	FALSE	0	8.118	10	2	6	2	1	310	35.1	5.91	13.23
Master Protein	Q07954	Prolow-density lipoprotein receptor-related protein 1 OS=Homo sapiens GN=LRP1 PE=1 SV=2	LRP1	High	FALSE	0	55.054	4	15	60	15	1	4544	504.3	5.39	131.19

Master Protein	Q12884	Prolyl endopeptidase FAP OS=Homo sapiens GN=FAP PE=1 SV=5	FAP	High	FALSE	0	20.11	8	6	105	5	1	760	87.7	6.65	184.35
Master Protein	P27918	Properdin OS=Homo sapiens GN=CFP PE=1 SV=2	CFP	High	FALSE	0	89.626	30	12	513	12	1	469	51.2	7.9	1322.42
Master Protein	Q8NBP7	Proprotein convertase subtilisin/kexin type 9 OS=Homo sapiens GN=PCSK9 PE=1 SV=3	PCSK9	High	FALSE	0	29.303	12	6	57	6	1	692	74.2	6.61	135.61
Master Protein	P41222	Prostaglandin-H2 D-isomerase OS=Homo sapiens GN=PTGDS PE=1 SV=1	PTGDS	High	FALSE	0	26.056	17	2	103	2	1	190	21	7.8	228.81
Master Protein	G3V5Z7	Proteasome subunit alpha type OS=Homo sapiens GN=PSMA6 PE=1 SV=1	PSMA6	High	FALSE	0	15.693	17	4	13	4	1	252	28.1	6.76	26.19
Master Protein	P25787	Proteasome subunit alpha type-2 OS=Homo sapiens GN=PSMA2 PE=1 SV=2	PSMA2	High	FALSE	0	13.047	24	3	6	3	1	234	25.9	7.43	9.08
Master Protein	P25789	Proteasome subunit alpha type-4 OS=Homo sapiens GN=PSMA4 PE=1 SV=1	PSMA4	High	FALSE	0	9.04	15	3	12	3	1	261	29.5	7.72	21.59
Master Protein	P28066	Proteasome subunit alpha type-5 OS=Homo sapiens GN=PSMA5 PE=1 SV=3	PSMA5	High	FALSE	0.005	4.594	10	2	3	2	1	241	26.4	4.79	5.2
Master Protein	O14818	Proteasome subunit alpha type-7 OS=Homo sapiens GN=PSMA7 PE=1 SV=1	PSMA7	High	FALSE	0	15.221	20	4	12	4	1	248	27.9	8.46	24.77
Master Protein	P49721	Proteasome subunit beta type-2 OS=Homo sapiens GN=PSMB2 PE=1 SV=1	PSMB2	High	FALSE	0.004	5.517	9	2	6	2	1	201	22.8	7.02	8.19
Master Protein	P49720	Proteasome subunit beta type-3 OS=Homo sapiens GN=PSMB3 PE=1 SV=2	PSMB3	High	FALSE	0.007	4.28	8	1	3	1	1	205	22.9	6.55	6.32
Master Protein	P28070	Proteasome subunit beta type-4 OS=Homo sapiens GN=PSMB4 PE=1 SV=4	PSMB4	High	FALSE	0.006	4.008	4	1	12	1	1	264	29.2	5.97	32.54
Master Protein	P28072	Proteasome subunit beta type-6 OS=Homo sapiens GN=PSMB6 PE=1 SV=4	PSMB6	High	FALSE	0.006	3.972	5	1	11	1	1	239	25.3	4.92	25.29
Master Protein	P28065	Proteasome subunit beta type-9 OS=Homo sapiens GN=PSMB9 PE=1 SV=2	PSMB9	High	FALSE	0.006	3.987	5	1	3	1	1	219	23.3	5.03	8.93
Master Protein	P02760	Protein AMBP OS=Homo sapiens GN=AMBP PE=1 SV=1	AMBP	High	FALSE	0	236.627	56	20	4636	20	1	352	39	6.25	12950.24
Master Protein	K7ER74	Protein APOC4-APOC2 OS=Homo sapiens GN=APOC4-APOC2 PE=4 SV=1	APOC4-APOC2	High	FALSE	0	90.427	39	9	4380	7	1	178	20	6.64	10447.68
Master Protein	H7BZJ3	Protein disulfide-isomerase A3 (Fragment) OS=Homo sapiens GN=PDIA3 PE=1 SV=1	PDIA3	High	FALSE	0	17.447	41	5	29	1	1	123	13.5	7.3	61.13
Master Protein	P30101	Protein disulfide-isomerase A3 OS=Homo sapiens GN=PDIA3 PE=1 SV=4	PDIA3	High	FALSE	0	55.325	31	14	113	10	1	505	56.7	6.35	260.59
Master Protein	P13667	Protein disulfide-isomerase A4 OS=Homo sapiens GN=PDIA4 PE=1 SV=2	PDIA4	High	FALSE	0	10.6	6	3	6	3	1	645	72.9	5.07	14.34
Master Protein	Q14554	Protein disulfide-isomerase A5 OS=Homo sapiens GN=PDIA5 PE=1 SV=1	PDIA5	High	FALSE	0.001	6.98	4	2	2	2	1	519	59.6	7.91	4.6
Master Protein	P07237	Protein disulfide-isomerase OS=Homo sapiens GN=P4HB PE=1 SV=3	P4HB	High	FALSE	0	47.2	29	11	87	11	1	508	57.1	4.87	177.87
Master Protein	Q99497	Protein DJ-1 OS=Homo sapiens GN=PARK7 PE=1 SV=2	PARK7	High	FALSE	0	19.03	24	4	36	4	1	189	19.9	6.79	94.48
Master Protein	Q9NUQ9	Protein FAM49B OS=Homo sapiens GN=FAM49B PE=1 SV=1	FAM49B	High	FALSE	0.003	5.922	7	2	2	2	1	324	36.7	6.06	4.71
Master Protein	A0A087WW89	Protein IGHV3-72 OS=Homo sapiens GN=IGHV3-72 PE=4 SV=1	IGHV3-72	High	FALSE	0	18.046	30	2	59	2	1	101	11.2	7.97	229.48
Master Protein	A0A075B7B8	Protein IGHV3OR16-12 (Fragment) OS=Homo sapiens GN=IGHV3OR16-12 PE=4 SV=1	IGHV3OR16-12	High	FALSE	0.005	4.579	9	1	12	1	1	117	12.9	6.51	35.97
Master Protein	A0A087WTK4	Protein IGKV1-16 OS=Homo sapiens GN=IGKV1-16 PE=4 SV=1	IGKV1-16	High	FALSE	0	17.936	35	2	24	1	1	97	10.4	7.96	60.3
Master Protein	A0A087X0N5	Protein IGKV1-17 OS=Homo sapiens GN=IGKV1-17 PE=4 SV=1	IGKV1-17	High	FALSE	0	20.282	34	2	27	1	1	97	10.5	8.5	70.4
Master Protein	A0A075B6R9	Protein IGKV2D-24 (Fragment) OS=Homo sapiens GN=IGKV2D-24 PE=4 SV=1	IGKV2D-24	High	FALSE	0.007	4.293	11	1	7	1	1	120	13.1	8.87	21.33
Master Protein	A0A075B6I8	Protein IGLV1-47 (Fragment) OS=Homo sapiens GN=IGLV1-47 PE=4 SV=1	IGLV1-47	High	FALSE	0.007	4.188	11	1	9	1	1	117	12.3	5.91	28.21
Master Protein	A0A075B6J8	Protein IGLV3-19 (Fragment) OS=Homo sapiens GN=IGLV3-19 PE=4 SV=1	IGLV3-19	High	FALSE	0.001	7.603	17	2	15	2	1	112	12	4.96	36.22
Master Protein	A0A075B6J3	Protein IGLV3-27 (Fragment) OS=Homo sapiens GN=IGLV3-27 PE=4 SV=1	IGLV3-27	High	FALSE	0.008	3.294	10	1	1	1	1	113	12.2	5.01	0

Master Protein	A0A075B6I9	Protein IGLV7-46 (Fragment) OS=Homo sapiens GN=IGLV7-46 PE=4 SV=2	IGLV7-46	High	FALSE	0.008	3.279	8	1	33	1	1	117	12.5	7.2	58.06
Master Protein	Q9BUN1	Protein MENT OS=Homo sapiens GN=MENT PE=2 SV=1	MENT	High	FALSE	0	8.148	7	2	27	2	1	341	36.7	8.59	63.77
Master Protein	P26447	Protein S100-A4 OS=Homo sapiens GN=S100A4 PE=1 SV=1	S100A4	High	FALSE	0.005	4.777	20	2	4	2	1	101	11.7	6.11	6.65
Master Protein	P31151	Protein S100-A7 OS=Homo sapiens GN=S100A7 PE=1 SV=4	S100A7	High	FALSE	0.006	3.691	11	1	2	1	1	101	11.5	6.77	4.79
Master Protein	P05109	Protein S100-A8 OS=Homo sapiens GN=S100A8 PE=1 SV=1	S100A8	High	FALSE	0	14	25	3	41	3	1	93	10.8	7.03	108.57
Master Protein	P06702	Protein S100-A9 OS=Homo sapiens GN=S100A9 PE=1 SV=1	S100A9	High	FALSE	0	25.218	45	4	72	4	1	114	13.2	6.13	198.79
Master Protein	P25815	Protein S100-P OS=Homo sapiens GN=S100P PE=1 SV=2	S100P	High	FALSE	0.007	3.838	14	1	2	1	1	95	10.4	4.88	3.81
Master Protein	A0A096LPE2	Protein SAA2-SAA4 OS=Homo sapiens GN=SAA2-SAA4 PE=4 SV=1	SAA2-SAA4	High	FALSE	0	46.356	44	9	1428	7	1	208	23.3	8.98	3393.16
Master Protein	G3V2W1	Protein Z-dependent protease inhibitor OS=Homo sapiens GN=SERPINA10 PE=3 SV=1	SERPINA10	High	FALSE	0	77.93	36	14	608	14	1	484	55.1	7.64	1288.71
Master Protein	Q92954	Proteoglycan 4 OS=Homo sapiens GN=PRG4 PE=1 SV=2	PRG4	High	FALSE	0	75.651	10	12	497	12	1	1404	151	9.5	1242.02
Master Protein	P00734	Prothrombin OS=Homo sapiens GN=F2 PE=1 SV=2	F2	High	FALSE	0	500.47	64	41	10585	41	1	622	70	5.9	33307.23
Master Protein	Q9HCL0	Protocadherin-18 OS=Homo sapiens GN=PCDH18 PE=2 SV=3	PCDH18	High	FALSE	0.007	4.232	1	1	5	1	1	1135	126.1	5.15	9.82
Master Protein	D6W5L6	Pulmonary surfactant-associated protein B OS=Homo sapiens GN=SFTPB PE=4 SV=1	SFTPB	High	FALSE	0	20.569	13	4	21	4	1	393	43.3	5.74	48.89
Master Protein	P00491	Purine nucleoside phosphorylase OS=Homo sapiens GN=PNP PE=1 SV=2	PNP	High	FALSE	0	35.83	31	7	37	7	1	289	32.1	6.95	108.59
Master Protein	P55786	Puromycin-sensitive aminopeptidase OS=Homo sapiens GN=NPEPPS PE=1 SV=2	NPEPPS	High	FALSE	0	9.22	3	2	10	2	1	919	103.2	5.72	23.63
Master Protein	P14618	Pyruvate kinase PKM OS=Homo sapiens GN=PKM PE=1 SV=4	PKM	High	FALSE	0	83.288	42	17	207	17	1	531	57.9	7.84	565.27
Master Protein	P31150	Rab GDP dissociation inhibitor alpha OS=Homo sapiens GN=GDI1 PE=1 SV=2	GDI1	High	FALSE	0	23.648	16	6	38	3	1	447	50.6	5.14	52.07
Master Protein	P50395	Rab GDP dissociation inhibitor beta OS=Homo sapiens GN=GDI2 PE=1 SV=2	GDI2	High	FALSE	0	46.725	33	11	59	8	1	445	50.6	6.47	105.9
Master Protein	Q13576	Ras GTPase-activating-like protein IQGAP2 OS=Homo sapiens GN=IQGAP2 PE=1 SV=4	IQGAP2	High	FALSE	0.006	4.36	1	1	4	1	1	1575	180.5	5.64	4.74
Master Protein	Q15404	Ras suppressor protein 1 OS=Homo sapiens GN=RSU1 PE=1 SV=3	RSU1	High	FALSE	0	31.693	30	7	105	7	1	277	31.5	8.65	229.27
Master Protein	P15153	Ras-related C3 botulinum toxin substrate 2 OS=Homo sapiens GN=RAC2 PE=1 SV=1	RAC2	High	FALSE	0.001	6.928	11	2	9	2	1	192	21.4	7.61	18.55
Master Protein	P61026	Ras-related protein Rab-10 OS=Homo sapiens GN=RAB10 PE=1 SV=1	RAB10	High	FALSE	0	16.182	17	3	41	1	1	200	22.5	8.38	94.98
Master Protein	Q15907	Ras-related protein Rab-11B OS=Homo sapiens GN=RAB11B PE=1 SV=4	RAB11B	High	FALSE	0	17.337	25	5	31	5	1	218	24.5	5.94	51.23
Master Protein	P61106	Ras-related protein Rab-14 OS=Homo sapiens GN=RAB14 PE=1 SV=4	RAB14	High	FALSE	0	8.355	9	2	26	1	1	215	23.9	6.21	67.1
Master Protein	Q9H0U4	Ras-related protein Rab-1B OS=Homo sapiens GN=RAB1B PE=1 SV=1	RAB1B	High	FALSE	0	28.153	34	6	59	4	1	201	22.2	5.73	136.71
Master Protein	O00194	Ras-related protein Rab-27B OS=Homo sapiens GN=RAB27B PE=1 SV=4	RAB27B	High	FALSE	0	10.546	18	3	7	3	1	218	24.6	5.52	13.37
Master Protein	Q9NRW1	Ras-related protein Rab-6B OS=Homo sapiens GN=RAB6B PE=1 SV=1	RAB6B	High	FALSE	0	11.263	15	3	23	2	1	208	23.4	5.53	60.24
Master Protein	P51149	Ras-related protein Rab-7a OS=Homo sapiens GN=RAB7A PE=1 SV=1	RAB7A	High	FALSE	0	10.211	16	3	8	3	1	207	23.5	6.7	11.19
Master Protein	P61006	Ras-related protein Rab-8A OS=Homo sapiens GN=RAB8A PE=1 SV=1	RAB8A	High	FALSE	0	12.539	17	3	39	1	1	207	23.7	9.07	90.68
Master Protein	P61224	Ras-related protein Rap-1b OS=Homo sapiens GN=RAP1B PE=1 SV=1	RAP1B	High	FALSE	0	32.098	53	8	60	8	1	184	20.8	5.78	147.82
Master Protein	A0A087WVC6	Receptor-type tyrosine-protein phosphatase eta OS=Homo sapiens GN=PTPRJ PE=4 SV=1	PTPRJ	High	FALSE	0	39.837	8	8	78	8	1	1342	146.5	5.62	218.29
Master Protein	P10586	Receptor-type tyrosine-protein phosphatase F OS=Homo sapiens GN=PTPRF PE=1 SV=2	PTPRF	High	FALSE	0	46.004	7	11	75	11	1	1907	212.7	6.3	166.46
Master Protein	P23470	Receptor-type tyrosine-protein phosphatase gamma OS=Homo sapiens GN=PTPRG PE=1 SV=4	PTPRG	High	FALSE	0	26.01	4	5	61	5	1	1445	161.9	6.42	163.28
Master Protein	Q13332	Receptor-type tyrosine-protein phosphatase S OS=Homo sapiens GN=PTPRS PE=1 SV=3	PTPRS	High	FALSE	0	34.533	6	8	36	8	1	1948	216.9	6.46	94.6
Master Protein	J3KQ66	Reelin OS=Homo sapiens GN=RELN PE=4 SV=1	RELN	High	FALSE	0	14.859	2	4	9	4	1	3460	388.2	5.88	18.25
Master Protein	P00352	Retinal dehydrogenase 1 OS=Homo sapiens GN=ALDH1A1 PE=1 SV=2	ALDH1A1	High	FALSE	0	19.366	13	5	15	5	1	501	54.8	6.73	34.19

Master Protein	Q99969	Retinoic acid receptor responder protein 2 OS=Homo sapiens GN=RARRES2 PE=1 SV=1	RARRES2	High	FALSE	0	16.443	21	3	29	3	1	163	18.6	9.09	96.47
Master Protein	P02753	Retinol-binding protein 4 OS=Homo sapiens GN=RBP4 PE=1 SV=3	RBP4	High	FALSE	0	123.12	71	12	4211	12	1	201	23	6.07	10278.62
Master Protein	O95980	Reversion-inducing cysteine-rich protein with Kazal motifs OS=Homo sapiens GN=RECK PE=1 SV=1	RECK	High	FALSE	0.001	6.986	2	2	3	2	1	971	106.4	6.74	6.91
Master Protein	J3QQX2	Rho GDP-dissociation inhibitor 1 OS=Homo sapiens GN=ARHGDIA PE=1 SV=1	ARHGDIA	High	FALSE	0	22.508	18	4	24	4	1	235	25.8	7.44	64
Master Protein	P52566	Rho GDP-dissociation inhibitor 2 OS=Homo sapiens GN=ARHGDIB PE=1 SV=3	ARHGDIB	High	FALSE	0	24.918	39	5	39	5	1	201	23	5.21	99.76
Master Protein	Q07960	Rho GTPase-activating protein 1 OS=Homo sapiens GN=ARHGAP1 PE=1 SV=1	ARHGAP1	High	FALSE	0	15.589	11	4	22	4	1	439	50.4	6.29	47.97
Master Protein	Q8N392	Rho GTPase-activating protein 18 OS=Homo sapiens GN=ARHGAP18 PE=1 SV=3	ARHGAP18	High	FALSE	0.005	4.714	3	2	3	2	1	663	74.9	6.44	1.62
Master Protein	P34096	Ribonuclease 4 OS=Homo sapiens GN=RNASE4 PE=1 SV=3	RNASE4	High	FALSE	0	19.181	25	4	19	4	1	147	16.8	9.03	44.42
Master Protein	P13489	Ribonuclease inhibitor OS=Homo sapiens GN=RNH1 PE=1 SV=2	RNH1	High	FALSE	0.001	6.985	6	2	7	2	1	461	49.9	4.82	18.34
Master Protein	Q8WZ75	Roundabout homolog 4 OS=Homo sapiens GN=ROBO4 PE=1 SV=1	ROBO4	High	FALSE	0	26.804	7	6	54	6	1	1007	107.4	6.64	131.05
Master Protein	B1AVU8	Saposin-D OS=Homo sapiens GN=PSAP PE=1 SV=1	PSAP	High	FALSE	0	14.017	7	4	20	4	1	559	61.7	5.22	44.43
Master Protein	Q13103	Secreted phosphoprotein 24 OS=Homo sapiens GN=SPP2 PE=1 SV=1	SPP2	High	FALSE	0	21.18	22	4	21	4	1	211	24.3	8.32	46.17
Master Protein	Q96QR1	Secretoglobin family 3A member 1 OS=Homo sapiens GN=SCGB3A1 PE=1 SV=2	SCGB3A1	High	FALSE	0.001	6.695	32	2	5	2	1	104	10.1	8.32	7.27
Master Protein	P49908	Selenoprotein P OS=Homo sapiens GN=SEPP1 PE=1 SV=3	SEPP1	High	FALSE	0	26.342	14	6	422	6	1	381	43.2	7.87	954.07
Master Protein	J3KNP4	Semaphorin-4B OS=Homo sapiens GN=SEMA4B PE=1 SV=1	SEMA4B	High	FALSE	0	13.787	5	4	16	4	1	837	92.7	7.05	25.57
Master Protein	E7EPK1	Septin-7 OS=Homo sapiens GN=SEPT7 PE=1 SV=2	SEPT7	High	FALSE	0	9.631	7	3	22	2	1	437	50.7	8.63	43.56
Master Protein	O95747	Serine/threonine-protein kinase OSR1 OS=Homo sapiens GN=OXSR1 PE=1 SV=1	OXSR1	High	FALSE	0.005	4.477	3	1	1	1	1	527	58	6.43	2.53
Master Protein	P02787	Serotransferrin OS=Homo sapiens GN=TF PE=1 SV=3	TF	High	FALSE	0	508.892	72	59	9875	59	1	698	77	7.12	28000.78
Master Protein	Q86U17	Serpin A11 OS=Homo sapiens GN=SERPINA11 PE=2 SV=2	SERPINA11	High	FALSE	0	41.02	24	10	97	10	1	422	47	7.68	256.69
Master Protein	A0A087X1N8	Serpin B6 OS=Homo sapiens GN=SERPINB6 PE=1 SV=1	SERPINB6	High	FALSE	0.004	4.965	5	2	2	2	1	395	44.8	5.68	2.24
Master Protein	P50453	Serpin B9 OS=Homo sapiens GN=SERPINB9 PE=1 SV=1	SERPINB9	High	FALSE	0.006	3.74	4	1	9	1	1	376	42.4	5.86	18.48
Master Protein	B7WNR0	Serum albumin OS=Homo sapiens GN=ALB PE=1 SV=1	ALB	High	FALSE	0	407.525	80	49	8170	1	1	494	56.2	7.14	23409.58
Master Protein	P0DJI8	Serum amyloid A-1 protein OS=Homo sapiens GN=SAA1 PE=1 SV=1	SAA1	High	FALSE	0	38.373	46	7	75	3	1	122	13.5	6.79	222.53
Master Protein	P0DJI9	Serum amyloid A-2 protein OS=Homo sapiens GN=SAA2 PE=1 SV=1	SAA2	High	FALSE	0	22.098	41	5	36	1	1	122	13.5	9.14	91.85
Master Protein	P02743	Serum amyloid P-component OS=Homo sapiens GN=APCS PE=1 SV=2	APCS	High	FALSE	0	48.716	35	10	1881	10	1	223	25.4	6.54	4802.92
Master Protein	O95810	Serum deprivation-response protein OS=Homo sapiens GN=SDPR PE=1 SV=3	SDPR	High	FALSE	0	36.287	28	9	78	9	1	425	47.1	5.21	186.97
Master Protein	P27169	Serum paraoxonase/arylesterase 1 OS=Homo sapiens GN=PON1 PE=1 SV=3	PON1	High	FALSE	0	131.167	62	16	1726	14	1	355	39.7	5.22	4457.92
Master Protein	Q15166	Serum paraoxonase/lactonase 3 OS=Homo sapiens GN=PON3 PE=1 SV=3	PON3	High	FALSE	0	72.694	42	11	357	9	1	354	39.6	5.41	814.99
Master Protein	P04278	Sex hormone-binding globulin OS=Homo sapiens GN=SHBG PE=1 SV=2	SHBG	High	FALSE	0	96.033	48	12	691	12	1	402	43.8	6.71	1774.99
Master Protein	P10768	S-formylglutathione hydrolase OS=Homo sapiens GN=ESD PE=1 SV=2	ESD	High	FALSE	0.007	4.153	6	1	6	1	1	282	31.4	7.02	8.03
Master Protein	A0A087WV23	SH3 domain-binding glutamic acid-rich-like protein 3 OS=Homo sapiens GN=SH3BGRL3 PE=1 SV=1	SH3BGRL3	High	FALSE	0.007	4.156	4	1	14	1	1	226	23.8	9.09	34.05
Master Protein	O75368	SH3 domain-binding glutamic acid-rich-like protein OS=Homo sapiens GN=SH3BGRL PE=1 SV=1	SH3BGRL	High	FALSE	0	9.572	31	3	10	3	1	114	12.8	5.25	14.48
Master Protein	A8MU27	Small ubiquitin-related modifier 3 OS=Homo sapiens GN=SUMO3 PE=1 SV=1	SUMO3	High	FALSE	0.007	3.915	8	1	3	1	1	147	16.9	9.67	7.08
Master Protein	A1L4H1	Soluble scavenger receptor cysteine-rich domain-containing protein SSC5D	SSC5D	High	FALSE	0.001	7.077	1	2	15	2	1	1573	165.6	6.13	39.47

		OS=Homo sapiens GN=SSC5D PE=2 SV=3														
Master Protein	P30626	Sorcin OS=Homo sapiens GN=SRI PE=1 SV=1	SRI	High	FALSE	0.008	3.36	6	1	9	1	1	198	21.7	5.59	14.09
Master Protein	D3DQH8	SPARC OS=Homo sapiens GN=SPARC PE=4 SV=1	SPARC	High	FALSE	0	22.154	17	5	20	5	1	315	36	4.88	55.15
Master Protein	Q14515	SPARC-like protein 1 OS=Homo sapiens GN=SPARCL1 PE=1 SV=2	SPARCL1	High	FALSE	0	18.102	6	3	19	3	1	664	75.2	4.81	40.53
Master Protein	O75563	Src kinase-associated phosphoprotein 2 OS=Homo sapiens GN=SKAP2 PE=1 SV=1	SKAP2	High	FALSE	0.003	5.87	6	2	5	2	1	359	41.2	4.69	5.79
Master Protein	Q14247	Src substrate cortactin OS=Homo sapiens GN=CTTN PE=1 SV=2	CTTN	High	FALSE	0.004	5.689	5	2	3	2	1	550	61.5	5.4	1.65
Master Protein	Q9NY15	Stabilin-1 OS=Homo sapiens GN=STAB1 PE=1 SV=3	STAB1	High	FALSE	0.001	6.549	1	2	2	2	1	2570	275.3	6.49	4.3
Master Protein	O00391	Sulfhydryl oxidase 1 OS=Homo sapiens GN=QSOX1 PE=1 SV=3	QSOX1	High	FALSE	0	112.71	26	18	479	18	1	747	82.5	8.92	1319.94
Master Protein	P04179	Superoxide dismutase [Mn], mitochondrial OS=Homo sapiens GN=SOD2 PE=1 SV=2	SOD2	High	FALSE	0.006	4.417	6	1	2	1	1	222	24.7	8.25	6.42
Master Protein	Q8TER0	Sushi, nidogen and EGF-like domain-containing protein 1 OS=Homo sapiens GN=SNED1 PE=2 SV=2	SNED1	High	FALSE	0	8.339	2	3	7	3	1	1413	152.1	6.98	9.91
Master Protein	Q4LDE5	Sushi, von Willebrand factor type A, EGF and pentraxin domain-containing protein 1 OS=Homo sapiens GN=SVEP1 PE=1 SV=3	SVEP1	High	FALSE	0.001	7.626	1	2	6	2	1	3571	389.9	5.5	14.36
Master Protein	Q9Y490	Talin-1 OS=Homo sapiens GN=TLN1 PE=1 SV=3	TLN1	High	FALSE	0	609.867	51	87	1712	87	1	2541	269.6	6.07	4511.52
Master Protein	D3YTG3	Target of Nesh-SH3 OS=Homo sapiens GN=ABI3BP PE=4 SV=1	ABI3BP	High	FALSE	0	19.668	3	5	93	5	1	1777	195.2	9.73	220.73
Master Protein	P17987	T-complex protein 1 subunit alpha OS=Homo sapiens GN=TCP1 PE=1 SV=1	TCP1	High	FALSE	0.007	4.256	2	1	2	1	1	556	60.3	6.11	5.56
Master Protein	P50991	T-complex protein 1 subunit delta OS=Homo sapiens GN=CCT4 PE=1 SV=4	CCT4	High	FALSE	0.001	7.176	4	2	3	2	1	539	57.9	7.83	6.64
Master Protein	P50990	T-complex protein 1 subunit theta OS=Homo sapiens GN=CCT8 PE=1 SV=4	CCT8	High	FALSE	0	8.085	4	2	10	2	1	548	59.6	5.6	22.57
Master Protein	P24821	Tenascin OS=Homo sapiens GN=TNC PE=1 SV=3	TNC	High	FALSE	0	57.205	9	14	92	14	1	2201	240.7	4.89	245.68
Master Protein	Q9UQP3	Tenascin-N OS=Homo sapiens GN=TNN PE=1 SV=2	TNN	High	FALSE	0.006	4.019	1	1	4	1	1	1299	143.9	5.63	5.85
Master Protein	P22105	Tenascin-X OS=Homo sapiens GN=TNXB PE=1 SV=3	TNXB	High	FALSE	0	144.652	12	30	376	30	1	4289	464	5.34	934.76
Master Protein	P05452	Tetranectin OS=Homo sapiens GN=CLEC3B PE=1 SV=3	CLEC3B	High	FALSE	0	55.202	50	9	1128	9	1	202	22.5	5.67	2828.55
Master Protein	G8JLH6	Tetraspanin (Fragment) OS=Homo sapiens GN=CD9 PE=1 SV=1	CD9	High	FALSE	0.005	4.455	4	1	9	1	1	228	25.4	6.52	28.21
Master Protein	P10599	Thioredoxin OS=Homo sapiens GN=TXN PE=1 SV=3	TXN	High	FALSE	0	7.837	21	2	26	2	1	105	11.7	4.92	84.6
Master Protein	O43396	Thioredoxin-like protein 1 OS=Homo sapiens GN=TXNL1 PE=1 SV=3	TXNL1	High	FALSE	0.001	7.483	8	2	11	2	1	289	32.2	4.96	19.33
Master Protein	P07996	Thrombospondin-1 OS=Homo sapiens GN=THBS1 PE=1 SV=2	THBS1	High	FALSE	0	125.705	25	22	425	22	1	1170	129.3	4.94	1075.2
Master Protein	P35443	Thrombospondin-4 OS=Homo sapiens GN=THBS4 PE=1 SV=2	THBS4	High	FALSE	0	59.301	14	10	78	8	1	961	105.8	4.68	234.88
Master Protein	P19971	Thymidine phosphorylase OS=Homo sapiens GN=TYMP PE=1 SV=2	TYMP	High	FALSE	0	19.764	17	6	13	6	1	482	49.9	5.53	22.01
Master Protein	Q9UKU6	Thyrotropin-releasing hormone-degrading ectoenzyme OS=Homo sapiens GN=TRHDE PE=2 SV=1	TRHDE	High	FALSE	0	13.982	5	4	12	4	1	1024	116.9	6.99	15.37
Master Protein	P05543	Thyroxine-binding globulin OS=Homo sapiens GN=SERPINA7 PE=1 SV=2	SERPINA7	High	FALSE	0	129.489	42	16	2373	16	1	415	46.3	6.3	5513.24
Master Protein	P04066	Tissue alpha-L-fucosidase OS=Homo sapiens GN=FUCA1 PE=1 SV=4	FUCA1	High	FALSE	0	16.11	11	4	48	4	1	466	53.7	6.84	113.22
Master Protein	P10646	Tissue factor pathway inhibitor OS=Homo sapiens GN=TFPI PE=1 SV=1	TFPI	High	FALSE	0.005	4.655	7	1	4	1	1	304	35	8.25	6.93
Master Protein	P37837	Transaldolase OS=Homo sapiens GN=TALDO1 PE=1 SV=2	TALDO1	High	FALSE	0	22.433	19	6	49	6	1	337	37.5	6.81	106.15
Master Protein	P02786	Transferrin receptor protein 1 OS=Homo sapiens GN=TFRC PE=1 SV=2	TFRC	High	FALSE	0	63.833	25	17	165	17	1	760	84.8	6.61	394.29
Master Protein	Q03167	Transforming growth factor beta receptor type 3 OS=Homo sapiens GN=TGFBR3 PE=1 SV=3	TGFBR3	High	FALSE	0	17.663	5	3	15	3	1	851	93.4	5.71	24.73
Master Protein	P01137	Transforming growth factor beta-1 OS=Homo sapiens GN=TGFB1 PE=1 SV=2	TGFB1	High	FALSE	0	10.216	10	3	5	3	1	390	44.3	8.53	11.12
Master Protein	Q15582	Transforming growth factor-beta-induced protein ig-h3 OS=Homo sapiens GN=TGFBI PE=1 SV=1	TGFBI	High	FALSE	0	124.761	35	19	708	19	1	683	74.6	7.71	1614.22

Master Protein	P55072	Transitional endoplasmic reticulum ATPase OS=Homo sapiens GN=VCP PE=1 SV=4	VCP	High	FALSE	0	49.714	18	12	60	12	1	806	89.3	5.26	139.69
Master Protein	Q66K66	Transmembrane protein 198 OS=Homo sapiens GN=TMEM198 PE=1 SV=1	TMEM198	High	FALSE	0.006	4.082	3	1	16	1	1	360	39.4	9.92	27.2
Master Protein	P02766	Transthyretin OS=Homo sapiens GN=TTR PE=1 SV=1	TTR	High	FALSE	0	155.028	80	12	10390	3	1	147	15.9	5.76	38666.17
Master Protein	A0A087WV45	Transthyretin OS=Homo sapiens GN=TTR PE=4 SV=1	TTR	High	FALSE	0	121.031	78	10	7702	1	1	139	15.1	5.76	28341.96
Master Protein	Q86YW5	Trem-like transcript 1 protein OS=Homo sapiens GN=TREML1 PE=1 SV=2	TREML1	High	FALSE	0	8.156	10	2	21	2	1	311	32.7	6.05	48.87
Master Protein	P60174	Triosephosphate isomerase OS=Homo sapiens GN=TPI1 PE=1 SV=3	TPI1	High	FALSE	0	63.272	51	9	155	9	1	286	30.8	5.92	410.17
Master Protein	B7Z596	Tropomyosin alpha-1 chain OS=Homo sapiens GN=TPM1 PE=1 SV=1	TPM1	High	FALSE	0	22.995	19	6	71	2	1	275	31.7	4.89	154.69
Master Protein	P67936	Tropomyosin alpha-4 chain OS=Homo sapiens GN=TPM4 PE=1 SV=3	TPM4	High	FALSE	0	56.83	35	11	213	7	1	248	28.5	4.69	521.98
Master Protein	P35030	Trypsin-3 OS=Homo sapiens GN=PRSS3 PE=1 SV=2	PRSS3	High	FALSE	0.007	4.293	4	1	9	1	1	304	32.5	7.49	22.01
Master Protein	P68363	Tubulin alpha-1B chain OS=Homo sapiens GN=TUBA1B PE=1 SV=1	TUBA1B	High	FALSE	0	36.552	27	9	54	2	1	451	50.1	5.06	127.64
Master Protein	P68366	Tubulin alpha-4A chain OS=Homo sapiens GN=TUBA4A PE=1 SV=1	TUBA4A	High	FALSE	0	35.802	26	9	67	2	1	448	49.9	5.06	155.44
Master Protein	P07437	Tubulin beta chain OS=Homo sapiens GN=TUBB PE=1 SV=2	TUBB	High	FALSE	0	23.331	16	5	39	3	1	444	49.6	4.89	70.39
Master Protein	Q9H4B7	Tubulin beta-1 chain OS=Homo sapiens GN=TUBB1 PE=1 SV=1	TUBB1	High	FALSE	0	30.551	18	7	50	5	1	451	50.3	5.17	110.42
Master Protein	Q6IBS0	Twinfilin-2 OS=Homo sapiens GN=TWF2 PE=1 SV=2	TWF2	High	FALSE	0	18.832	15	3	15	3	1	349	39.5	6.84	39.63
Master Protein	Q12866	Tyrosine-protein kinase Mer OS=Homo sapiens GN=MERTK PE=1 SV=2	MERTK	High	FALSE	0.003	5.822	2	2	2	2	1	999	110.2	5.8	1.61
Master Protein	P35590	Tyrosine-protein kinase receptor Tie-1 OS=Homo sapiens GN=TIE1 PE=1 SV=1	TIE1	High	FALSE	0	8.597	2	3	16	3	1	1138	125	7.03	32.71
Master Protein	P30530	Tyrosine-protein kinase receptor UFO OS=Homo sapiens GN=AXL PE=1 SV=3	AXL	High	FALSE	0	8.248	3	2	15	2	1	894	98.3	5.43	38.75
Master Protein	P29074	Tyrosine-protein phosphatase non-receptor type 4 OS=Homo sapiens GN=PTPN4 PE=1 SV=1	PTPN4	High	FALSE	0.009	3.265	2	1	100	1	1	926	105.8	7.44	103.42
Master Protein	P54577	Tyrosine--tRNA ligase, cytoplasmic OS=Homo sapiens GN=YARS PE=1 SV=4	YARS	High	FALSE	0.004	6.003	4	2	6	2	1	528	59.1	7.05	3.94
Master Protein	P54578	Ubiquitin carboxyl-terminal hydrolase 14 OS=Homo sapiens GN=USP14 PE=1 SV=3	USP14	High	FALSE	0.005	4.54	3	1	7	1	1	494	56	5.3	22.12
Master Protein	P62979	Ubiquitin-40S ribosomal protein S27a OS=Homo sapiens GN=RPS27A PE=1 SV=2	RPS27A	High	FALSE	0	16.619	30	4	108	4	1	156	18	9.64	301.97
Master Protein	P61086	Ubiquitin-conjugating enzyme E2 K OS=Homo sapiens GN=UBE2K PE=1 SV=3	UBE2K	High	FALSE	0.007	4.201	6	1	4	1	1	200	22.4	5.44	10.01
Master Protein	P68036	Ubiquitin-conjugating enzyme E2 L3 OS=Homo sapiens GN=UBE2L3 PE=1 SV=1	UBE2L3	High	FALSE	0	16.224	31	4	41	3	1	154	17.9	8.51	53.45
Master Protein	P61088	Ubiquitin-conjugating enzyme E2 N OS=Homo sapiens GN=UBE2N PE=1 SV=1	UBE2N	High	FALSE	0	12.242	29	4	12	4	1	152	17.1	6.57	12.31
Master Protein	P61960	Ubiquitin-fold modifier 1 OS=Homo sapiens GN=UFM1 PE=1 SV=1	UFM1	High	FALSE	0.006	4.36	18	1	2	1	1	85	9.1	9.31	2.15
Master Protein	P22314	Ubiquitin-like modifier-activating enzyme 1 OS=Homo sapiens GN=UBA1 PE=1 SV=3	UBA1	High	FALSE	0	31.454	9	7	30	7	1	1058	117.8	5.76	63.9
Master Protein	P41226	Ubiquitin-like modifier-activating enzyme 7 OS=Homo sapiens GN=UBA7 PE=1 SV=2	UBA7	High	FALSE	0	16.564	5	4	16	4	1	1012	111.6	6.04	33.63
Master Protein	Q9NY97	UDP-GlcNAc:betaGal beta-1,3-N-acetylglucosaminyltransferase 2 OS=Homo sapiens GN=B3GNT2 PE=1 SV=2	B3GNT2	High	FALSE	0	8.307	9	3	16	3	1	397	46	8.54	24.94
Master Protein	X6RBG4	Uromodulin OS=Homo sapiens GN=UMOD PE=4 SV=1	UMOD	High	FALSE	0	15.252	7	4	28	4	1	689	75.6	5.87	61.3
Master Protein	E7EUC7	UTP--glucose-1-phosphate uridylyltransferase OS=Homo sapiens GN=UGP2 PE=1 SV=1	UGP2	High	FALSE	0	26.649	19	8	32	8	1	517	57.8	8.13	53.53
Master Protein	P54727	UV excision repair protein RAD23 homolog B OS=Homo sapiens GN=RAD23B PE=1 SV=1	RAD23B	High	FALSE	0.001	7.484	6	3	6	3	1	409	43.1	4.84	9.94
Master Protein	P19320	Vascular cell adhesion protein 1 OS=Homo sapiens GN=VCAM1 PE=1 SV=1	VCAM1	High	FALSE	0	70.387	26	16	117	16	1	739	81.2	5.22	295.56
Master Protein	P35916	Vascular endothelial growth factor receptor 3 OS=Homo sapiens GN=FLT4 PE=1 SV=3	FLT4	High	FALSE	0	18.458	4	3	39	3	1	1363	152.7	6.3	122.08

Master Protein	P50552	Vasodilator-stimulated phosphoprotein OS=Homo sapiens GN=VASP PE=1 SV=3	VASP	High	FALSE	0	17.571	9	4	20	4	1	380	39.8	8.94	72.3
Master Protein	Q6EMK4	Vasorin OS=Homo sapiens GN=VASN PE=1 SV=1	VASN	High	FALSE	0	42.641	12	6	208	6	1	673	71.7	7.39	602.06
Master Protein	Q12907	Vesicular integral-membrane protein VIP36 OS=Homo sapiens GN=LMAN2 PE=1 SV=1	LMAN2	High	FALSE	0	7.708	6	2	23	2	1	356	40.2	6.95	59.02
Master Protein	P18206	Vinculin OS=Homo sapiens GN=VCL PE=1 SV=4	VCL	High	FALSE	0	332.427	54	50	1284	50	1	1134	123.7	5.66	3109.85
Master Protein	D6RF35	Vitamin D-binding protein OS=Homo sapiens GN=GC PE=1 SV=1	GC	High	FALSE	0	421.681	80	51	15170	3	1	476	53	5.52	41977.46
Master Protein	P07225	Vitamin K-dependent protein S OS=Homo sapiens GN=PROS1 PE=1 SV=1	PROS1	High	FALSE	0	145.825	36	22	1138	22	1	676	75.1	5.67	3177.58
Master Protein	P04004	Vitronectin OS=Homo sapiens GN=VTN PE=1 SV=1	VTN	High	FALSE	0	191.395	43	23	5096	21	1	478	54.3	5.8	14365.28
Master Protein	P54289	Voltage-dependent calcium channel subunit alpha-2/delta-1 OS=Homo sapiens GN=CACNA2D1 PE=1 SV=3	CACNA2D1	High	FALSE	0	54.176	15	14	72	14	1	1103	124.5	5.27	152.46
Master Protein	P04275	von Willebrand factor OS=Homo sapiens GN=VWF PE=1 SV=4	VWF	High	FALSE	0	182.195	17	40	382	40	1	2813	309.1	5.48	894.39
Master Protein	P38606	V-type proton ATPase catalytic subunit A OS=Homo sapiens GN=ATP6V1A PE=1 SV=2	ATP6V1A	High	FALSE	0.007	3.916	2	1	1	1	1	617	68.3	5.52	2.21
Master Protein	O75083	WD repeat-containing protein 1 OS=Homo sapiens GN=WDR1 PE=1 SV=4	WDR1	High	FALSE	0	67.101	27	15	202	15	1	606	66.2	6.65	430.8
Master Protein	O43895	Xaa-Pro aminopeptidase 2 OS=Homo sapiens GN=XPNPEP2 PE=1 SV=3	XPNPEP2	High	FALSE	0	11.558	5	3	15	3	1	674	75.6	6.04	35.18
Master Protein	P12955	Xaa-Pro dipeptidase OS=Homo sapiens GN=PEPD PE=1 SV=3	PEPD	High	FALSE	0	44.468	21	10	167	10	1	493	54.5	6	372.54
Master Protein	P25311	Zinc-alpha-2-glycoprotein OS=Homo sapiens GN=AZGP1 PE=1 SV=2	AZGP1	High	FALSE	0	215.318	69	28	3819	28	1	298	34.2	6.05	9934.51
Master Protein	Q15942	Zyxin OS=Homo sapiens GN=ZYX PE=1 SV=1	ZYX	High	FALSE	0	35.845	23	8	88	8	1	572	61.2	6.67	203.92
Master Protein	Q9Y2I7	1-phosphatidylinositol 3-phosphate 5-kinase OS=Homo sapiens GN=PIKFYVE PE=1 SV=3	PIKFYVE	Medium	FALSE	0.013	2.923	1	1	26	1	1	2098	237	6.7	43.99
Master Protein	Q6P2Q0	ABCB9 protein OS=Homo sapiens GN=ABCB9 PE=2 SV=1	ABCB9	Medium	FALSE	0.038	2.204	1	1	107	1	1	769	84.7	7.93	154.03
Master Protein	P23526	Adenosylhomocysteinase OS=Homo sapiens GN=AHCY PE=1 SV=4	AHCY	Medium	FALSE	0.036	2.318	2	1	1	1	1	432	47.7	6.34	2.16
Master Protein	P05186	Alkaline phosphatase, tissue-nonspecific isozyme OS=Homo sapiens GN=ALPL PE=1 SV=4	ALPL	Medium	FALSE	0.036	2.308	3	1	2	1	1	524	57.3	6.67	0
Master Protein	Q96IU4	Alpha/beta hydrolase domain-containing protein 14B OS=Homo sapiens GN=ABHD14B PE=1 SV=1	ABHD14B	Medium	FALSE	0.013	2.875	5	1	3	1	1	210	22.3	6.4	4.06
Master Protein	Q09328	Alpha-1,6-mannosylglycoprotein 6-beta-N-acetylglucosaminyltransferase A OS=Homo sapiens GN=MGAT5 PE=1 SV=1	MGAT5	Medium	FALSE	0.036	2.247	1	1	8	1	1	741	84.5	8.12	16.84
Master Protein	J3KQL8	Apolipoprotein L2 OS=Homo sapiens GN=APOL2 PE=1 SV=2	APOL2	Medium	FALSE	0.046	2.051	2	1	10	1	1	449	48.9	6	22.6
Master Protein	P18054	Arachidonate 12-lipoxygenase, 12S-type OS=Homo sapiens GN=ALOX12 PE=1 SV=4	ALOX12	Medium	FALSE	0.035	2.339	2	1	1	1	1	663	75.6	6.21	2.05
Master Protein	Q96CX2	BTB/POZ domain-containing protein KCTD12 OS=Homo sapiens GN=KCTD12 PE=1 SV=1	KCTD12	Medium	FALSE	0.017	2.584	4	1	2	1	1	325	35.7	5.64	0
Master Protein	P15085	Carboxypeptidase A1 OS=Homo sapiens GN=CPA1 PE=1 SV=2	CPA1	Medium	FALSE	0.015	2.686	4	1	2	1	1	419	47.1	5.76	3.49
Master Protein	X6R8A1	Carboxypeptidase OS=Homo sapiens GN=CTSA PE=1 SV=1	CTSA	Medium	FALSE	0.015	2.729	3	1	3	1	1	498	56.2	6.61	1.78
Master Protein	P31944	Caspase-14 OS=Homo sapiens GN=CASP14 PE=1 SV=2	CASP14	Medium	FALSE	0.015	2.763	3	1	3	1	1	242	27.7	5.58	4.01
Master Protein	Q6ZRH7	Cation channel sperm-associated protein subunit gamma OS=Homo sapiens GN=CATSPERG PE=2 SV=3	CATSPERG	Medium	FALSE	0.029	2.441	3	1	1	1	1	1159	132.9	6.37	0
Master Protein	Q9Y696	Chloride intracellular channel protein 4 OS=Homo sapiens GN=CLIC4 PE=1 SV=4	CLIC4	Medium	FALSE	0.013	2.881	3	1	3	1	1	253	28.8	5.59	6.06
Master Protein	Q8NDM7	Cilia- and flagella-associated protein 43 OS=Homo sapiens GN=CFAP43 PE=2 SV=3	CFAP43	Medium	FALSE	0.046	2.054	1	1	1	1	1	1665	191.9	5.99	0
Master Protein	O00748	Cocaine esterase OS=Homo sapiens GN=CES2 PE=1 SV=1	CES2	Medium	FALSE	0.013	2.899	2	1	6	1	1	559	61.8	6.1	1.92
Master Protein	P08123	Collagen alpha-2(I) chain OS=Homo sapiens GN=COL1A2 PE=1 SV=7	COL1A2	Medium	FALSE	0.013	2.858	1	1	3	1	1	1366	129.2	8.95	3.53
Master Protein	J3KNI1	Conserved oligomeric Golgi complex subunit 4 OS=Homo sapiens GN=COG4 PE=1 SV=1	COG4	Medium	FALSE	0.029	2.436	1	1	1	1	1	789	89.4	5.21	0
Master Protein	P22528	Cornifin-B OS=Homo sapiens GN=SPRR1B PE=1 SV=2	SPRR1B	Medium	FALSE	0.028	2.49	9	1	1	1	1	89	9.9	8.48	0

Master Protein	P46109	Crk-like protein OS=Homo sapiens GN=CRKL PE=1 SV=1	CRKL	Medium	FALSE	0.026	2.555	4	1	2	1	1	303	33.8	6.74	1.89
Master Protein	Q70SY1	Cyclic AMP-responsive element-binding protein 3-like protein 2 OS=Homo sapiens GN=CREB3L2 PE=1 SV=3	CREB3L2	Medium	FALSE	0.04	2.176	2	1	9	1	1	520	57.4	5.43	10.68
Master Protein	G3V5T9	Cyclin-dependent kinase 2 OS=Homo sapiens GN=CDK2 PE=1 SV=1	CDK2	Medium	FALSE	0.011	3.178	5	1	43	1	1	346	39.2	8.62	1.63
Master Protein	P09417	Dihydropteridine reductase OS=Homo sapiens GN=QDPR PE=1 SV=2	QDPR	Medium	FALSE	0.015	2.667	5	1	1	1	1	244	25.8	7.37	1.89
Master Protein	M0R2J8	Doublecortin domain-containing protein 1 OS=Homo sapiens GN=DCDC1 PE=4 SV=1	DCDC1	Medium	FALSE	0.041	2.12	0	1	703	1	1	1783	200.5	8.9	463.27
Master Protein	Q8TD57	Dynein heavy chain 3, axonemal OS=Homo sapiens GN=DNAH3 PE=2 SV=1	DNAH3	Medium	FALSE	0.036	2.299	0	1	11	1	1	4116	470.5	6.43	2.44
Master Protein	P24534	Elongation factor 1-beta OS=Homo sapiens GN=EEF1B2 PE=1 SV=3	EEF1B2	Medium	FALSE	0.017	2.641	4	1	1	1	1	225	24.7	4.67	1.76
Master Protein	B5ME19	Eukaryotic translation initiation factor 3 subunit C-like protein OS=Homo sapiens GN=EIF3CL PE=3 SV=1	EIF3CL	Medium	FALSE	0.04	2.136	1	1	4	1	1	914	105.4	5.64	7.41
Master Protein	P14324	Farnesyl pyrophosphate synthase OS=Homo sapiens GN=FDPS PE=1 SV=4	FDPS	Medium	FALSE	0.017	2.616	2	1	1	1	1	419	48.2	6.15	1.98
Master Protein	P02794	Ferritin heavy chain OS=Homo sapiens GN=FTH1 PE=1 SV=2	FTH1	Medium	FALSE	0.029	2.432	4	1	2	1	1	183	21.2	5.55	1.64
Master Protein	Q86WI1	Fibrocystin-L OS=Homo sapiens GN=PKHD1L1 PE=2 SV=2	PKHD1L1	Medium	FALSE	0.03	2.394	0	1	1	1	1	4243	465.4	6.11	1.9
Master Protein	Q6PIW4	Fidgetin-like protein 1 OS=Homo sapiens GN=FIGNL1 PE=1 SV=2	FIGNL1	Medium	FALSE	0.015	2.692	2	1	2	1	1	674	74	7.85	3.58
Master Protein	Q12841	Follistatin-related protein 1 OS=Homo sapiens GN=FSTL1 PE=1 SV=1	FSTL1	Medium	FALSE	0.013	2.835	3	1	15	1	1	308	35	5.52	24.35
Master Protein	Q9H479	Fructosamine-3-kinase OS=Homo sapiens GN=FN3K PE=1 SV=1	FN3K	Medium	FALSE	0.026	2.556	4	1	1	1	1	309	35.1	7.55	0
Master Protein	Q13630	GDP-L-fucose synthase OS=Homo sapiens GN=TSTA3 PE=1 SV=1	TSTA3	Medium	FALSE	0.015	2.734	4	1	1	1	1	321	35.9	6.6	1.98
Master Protein	P07203	Glutathione peroxidase 1 OS=Homo sapiens GN=GPX1 PE=1 SV=4	GPX1	Medium	FALSE	0.027	2.547	5	1	3	1	1	203	22.1	6.55	4.02
Master Protein	P02724	Glycophorin-A OS=Homo sapiens GN=GYPA PE=1 SV=2	GYPA	Medium	FALSE	0.029	2.408	20	1	4	1	1	150	16.3	5.47	2.01
Master Protein	P48723	Heat shock 70 kDa protein 13 OS=Homo sapiens GN=HSPA13 PE=1 SV=1	HSPA13	Medium	FALSE	0.013	2.937	2	1	1	1	1	471	51.9	5.76	0
Master Protein	Q96RW7	Hemicentin-1 OS=Homo sapiens GN=HMCN1 PE=1 SV=2	HMCN1	Medium	FALSE	0.017	2.643	0	1	6	1	1	5635	613	6.49	0
Master Protein	Q4G0P3	Hydrocephalus-inducing protein homolog OS=Homo sapiens GN=HYDIN PE=1 SV=3	HYDIN	Medium	FALSE	0.036	2.28	0	1	189	1	1	5121	575.5	6.06	15.17
Master Protein	P01824	Ig heavy chain V-II region WAH OS=Homo sapiens PE=1 SV=1	IGHV4-39	Medium	FALSE	0.036	2.293	7	1	1	1	1	129	14.1	8.46	2.21
Master Protein	P06316	Ig lambda chain V-I region BL2 OS=Homo sapiens PE=2 SV=1	IGLV1-51	Medium	FALSE	0.017	2.646	6	1	2	1	1	130	13.6	7.77	1.93
Master Protein	P05231	Interleukin-6 OS=Homo sapiens GN=IL6 PE=1 SV=1	IL6	Medium	FALSE	0.036	2.266	5	1	2	1	1	212	23.7	6.57	0
Master Protein	P05089-2	Isoform 2 of Arginase-1 OS=Homo sapiens GN=ARG1	ARG1	Medium	FALSE	0.04	2.187	3	1	2	1	1	330	35.6	7.21	3.9
Master Protein	Q14839-2	Isoform 2 of Chromodomain-helicase-DNA-binding protein 4 OS=Homo sapiens GN=CHD4	CHD4	Medium	FALSE	0.035	2.337	1	1	23	1	1	1940	220.7	5.97	19.43
Master Protein	Q8N0W4-2	Isoform 2 of Neuroligin-4, X-linked OS=Homo sapiens GN=NLGN4X	NLGN4X	Medium	FALSE	0.047	2.061	2	1	3	1	1	836	94	6.05	4.64
Master Protein	P62136-2	Isoform 2 of Serine/threonine-protein phosphatase PP1-alpha catalytic subunit OS=Homo sapiens GN=PPP1CA	PPP1CA	Medium	FALSE	0.035	2.338	3	1	2	1	1	341	38.6	6.62	3.43
Master Protein	P59998-3	Isoform 3 of Actin-related protein 2/3 complex subunit 4 OS=Homo sapiens GN=ARPC4	ARPC4	Medium	FALSE	0.013	2.94	4	1	15	1	1	187	21.6	8.59	22.25
Master Protein	Q8WWM7-3	Isoform 3 of Ataxin-2-like protein OS=Homo sapiens GN=ATXN2L	ATXN2L	Medium	FALSE	0.045	2.083	1	1	18	1	1	1097	115.5	8.66	43.42
Master Protein	P36268-3	Isoform 3 of Inactive gamma-glutamyltranspeptidase 2 OS=Homo sapiens GN=GGT2	GGT2	Medium	FALSE	0.047	2.055	2	1	1	1	1	574	62.1	7.33	2.09
Master Protein	Q9UNZ2-5	Isoform 3 of NSFL1 cofactor p47 OS=Homo sapiens GN=NSFL1C	NSFL1C	Medium	FALSE	0.011	3.08	4	1	1	1	1	372	40.8	5.15	1.93
Master Protein	P23467-3	Isoform 3 of Receptor-type tyrosine-protein phosphatase beta OS=Homo sapiens GN=PTPRB	PTPRB	Medium	FALSE	0.013	2.85	0	1	2	1	1	2215	249	7.74	1.73
Master Protein	Q13596-3	Isoform 3 of Sorting nexin-1 OS=Homo sapiens GN=SNX1	SNX1	Medium	FALSE	0.047	2.036	2	1	1	1	1	557	63	5.48	1.68
Master Protein	Q14766-4	Isoform 4 of Latent-transforming growth factor beta-binding protein 1 OS=Homo sapiens GN=LTBP1	LTBP1	Medium	FALSE	0.011	3.111	1	1	3	1	1	1722	186.7	5.96	1.87
Master Protein	P53990-5	Isoform 5 of IST1 homolog OS=Homo sapiens GN=IST1	IST1	Medium	FALSE	0.043	2.097	2	1	1	1	1	379	41.5	5.57	1.71

Master Protein	Q92823-5	Isoform 5 of Neuronal cell adhesion molecule OS=Homo sapiens GN=NRCAM	NRCAM	Medium	FALSE	0.029	2.402	1	1	1	1	1	1308	144.3	5.67	2.48
Master Protein	P21583	Kit ligand OS=Homo sapiens GN=KITLG PE=1 SV=1	KITLG	Medium	FALSE	0.017	2.637	3	1	10	1	1	273	30.9	6.14	12.18
Master Protein	Q86WA8	Lon protease homolog 2, peroxisomal OS=Homo sapiens GN=LONP2 PE=1 SV=1	LONP2	Medium	FALSE	0.036	2.253	2	1	1	1	1	852	94.6	7.3	0
Master Protein	Q9H8J5	MANSC domain-containing protein 1 OS=Homo sapiens GN=MANSC1 PE=2 SV=1	MANSC1	Medium	FALSE	0.036	2.281	2	1	2	1	1	431	46.8	6.54	2.28
Master Protein	Q96HR3	Mediator of RNA polymerase II transcription subunit 30 OS=Homo sapiens GN=MED30 PE=1 SV=1	MED30	Medium	FALSE	0.013	2.823	5	1	14	1	1	178	20.3	8.27	20.24
Master Protein	Q15555	Microtubule-associated protein RP/EB family member 2 OS=Homo sapiens GN=MAPRE2 PE=1 SV=1	MAPRE2	Medium	FALSE	0.046	2.08	2	1	4	1	1	327	37	5.57	1.69
Master Protein	P28482	Mitogen-activated protein kinase 1 OS=Homo sapiens GN=MAPK1 PE=1 SV=3	MAPK1	Medium	FALSE	0.048	2.009	2	1	62	1	1	360	41.4	6.98	121.01
Master Protein	Q9H7P6	Multivesicular body subunit 12B OS=Homo sapiens GN=MVB12B PE=1 SV=2	MVB12B	Medium	FALSE	0.045	2.066	3	1	27	1	1	319	35.6	8.15	16.35
Master Protein	P35579	Myosin-9 OS=Homo sapiens GN=MYH9 PE=1 SV=4	MYH9	Medium	FALSE	0.028	2.5	1	1	10	1	1	1960	226.4	5.6	0
Master Protein	Q86SF2	N-acetylgalactosaminyltransferase 7 OS=Homo sapiens GN=GALNT7 PE=1 SV=1	GALNT7	Medium	FALSE	0.015	2.688	2	1	4	1	1	657	75.3	7.11	7.71
Master Protein	Q09666	Neuroblast differentiation-associated protein AHNAK OS=Homo sapiens GN=AHNAK PE=1 SV=2	AHNAK	Medium	FALSE	0.036	2.36	0	1	9	1	1	5890	628.7	6.15	16.81
Master Protein	P61970	Nuclear transport factor 2 OS=Homo sapiens GN=NUTF2 PE=1 SV=1	NUTF2	Medium	FALSE	0.014	2.725	6	1	1	1	1	127	14.5	5.38	2.05
Master Protein	Q02818	Nucleobindin-1 OS=Homo sapiens GN=NUCB1 PE=1 SV=4	NUCB1	Medium	FALSE	0.036	2.356	2	1	1	1	1	461	53.8	5.25	0
Master Protein	A0A087WV17	Osteoclast-associated immunoglobulin-like receptor OS=Homo sapiens GN=OSCAR PE=4 SV=1	OSCAR	Medium	FALSE	0.011	2.955	3	1	2	1	1	286	30.8	6.52	5.57
Master Protein	P10451	Osteopontin OS=Homo sapiens GN=SPP1 PE=1 SV=1	SPP1	Medium	FALSE	0.017	2.596	3	1	3	1	1	314	35.4	4.58	7.07
Master Protein	P78562	Phosphate-regulating neutral endopeptidase OS=Homo sapiens GN=PHEX PE=1 SV=1	PHEX	Medium	FALSE	0.047	2.031	3	1	2	1	1	749	86.4	8.76	0
Master Protein	P78356	Phosphatidylinositol 5-phosphate 4-kinase type-2 beta OS=Homo sapiens GN=PIP4K2B PE=1 SV=1	PIP4K2B	Medium	FALSE	0.011	3.015	2	1	2	1	1	416	47.3	7.33	2.06
Master Protein	P35558	Phosphoenolpyruvate carboxykinase, cytosolic [GTP] OS=Homo sapiens GN=PCK1 PE=1 SV=3	PCK1	Medium	FALSE	0.048	2.019	1	1	12	1	1	622	69.2	6.14	0
Master Protein	Q9NRG1	Phosphoribosyltransferase domain-containing protein 1 OS=Homo sapiens GN=PRTFDC1 PE=1 SV=1	PRTFDC1	Medium	FALSE	0.036	2.256	3	1	2	1	1	225	25.7	6.15	0
Master Protein	O75915	PRA1 family protein 3 OS=Homo sapiens GN=ARL6IP5 PE=1 SV=1	ARL6IP5	Medium	FALSE	0.048	2.019	10	1	13	1	1	188	21.6	9.77	0
Master Protein	Q86YR7	Probable guanine nucleotide exchange factor MCF2L2 OS=Homo sapiens GN=MCF2L2 PE=2 SV=3	MCF2L2	Medium	FALSE	0.029	2.437	1	1	2	1	1	1114	126.9	6.42	0
Master Protein	P20618	Proteasome subunit beta type-1 OS=Homo sapiens GN=PSMB1 PE=1 SV=2	PSMB1	Medium	FALSE	0.013	2.914	4	1	1	1	1	241	26.5	8.13	1.88
Master Protein	A0A075B6I0	Protein IGLV8-61 (Fragment) OS=Homo sapiens GN=IGLV8-61 PE=4 SV=2	IGLV8-61	Medium	FALSE	0.011	2.979	7	1	3	1	1	123	12.9	4.55	5.94
Master Protein	Q9UNF0	Protein kinase C and casein kinase substrate in neurons protein 2 OS=Homo sapiens GN=PACSIN2 PE=1 SV=2	PACSIN2	Medium	FALSE	0.013	2.928	2	1	3	1	1	486	55.7	5.2	5.96
Master Protein	P31949	Protein S100-A11 OS=Homo sapiens GN=S100A11 PE=1 SV=2	S100A11	Medium	FALSE	0.011	3.02	9	1	1	1	1	105	11.7	7.12	2.02
Master Protein	P06703	Protein S100-A6 OS=Homo sapiens GN=S100A6 PE=1 SV=1	S100A6	Medium	FALSE	0.015	2.744	9	1	24	1	1	90	10.2	5.48	46.83
Master Protein	H7BY58	Protein-L-isoaspartate O-methyltransferase OS=Homo sapiens GN=PCMT1 PE=1 SV=1	PCMT1	Medium	FALSE	0.027	2.551	3	1	1	1	1	286	30.3	6.73	2.93
Master Protein	P16109	P-selectin OS=Homo sapiens GN=SELP PE=1 SV=3	SELP	Medium	FALSE	0.043	2.109	1	1	2	1	1	830	90.8	6.6	1.95
Master Protein	Q86UN3	Reticulon-4 receptor-like 2 OS=Homo sapiens GN=RTN4RL2 PE=1 SV=1	RTN4RL2	Medium	FALSE	0.036	2.355	3	1	2	1	1	420	46.1	7.62	1.8
Master Protein	A0A087WZM2	Ribonuclease T2 OS=Homo sapiens GN=RNASET2 PE=1 SV=1	RNASET2	Medium	FALSE	0.013	2.933	3	1	1	1	1	306	34.9	7.11	1.84
Master Protein	Q13275	Semaphorin-3F OS=Homo sapiens GN=SEMA3F PE=2 SV=2	SEMA3F	Medium	FALSE	0.015	2.768	2	1	1	1	1	785	88.3	8.27	2.05
Master Protein	Q9NS98	Semaphorin-3G OS=Homo sapiens GN=SEMA3G PE=2 SV=1	SEMA3G	Medium	FALSE	0.034	2.365	1	1	1	1	1	782	86.6	7.78	2.06

Master Protein	A6NMB1	Sialic acid-binding Ig-like lectin 16 OS=Homo sapiens GN=SIGLEC16 PE=2 SV=3	SIGLEC16	Medium	FALSE	0.04	2.157	1	1	239	1	1	481	53	9.03	447.88
Master Protein	O43699	Sialic acid-binding Ig-like lectin 6 OS=Homo sapiens GN=SIGLEC6 PE=1 SV=2	SIGLEC6	Medium	FALSE	0.038	2.203	6	1	2	1	1	453	49.9	7.15	4.46
Master Protein	Q8TAQ9	SUN domain-containing protein 3 OS=Homo sapiens GN=SUN3 PE=2 SV=4	SUN3	Medium	FALSE	0.036	2.268	2	1	77	1	1	357	40.5	7.83	172.31
Master Protein	P00441	Superoxide dismutase [Cu-Zn] OS=Homo sapiens GN=SOD1 PE=1 SV=2	SOD1	Medium	FALSE	0.017	2.616	9	1	1	1	1	154	15.9	6.13	1.81
Master Protein	Q9BYX2	TBC1 domain family member 2A OS=Homo sapiens GN=TBC1D2 PE=1 SV=3	TBC1D2	Medium	FALSE	0.048	2.012	1	1	485	1	1	928	105.3	6.58	793.28
Master Protein	O95881	Thioredoxin domain-containing protein 12 OS=Homo sapiens GN=TXNDC12 PE=1 SV=1	TXNDC12	Medium	FALSE	0.015	2.781	5	1	1	1	1	172	19.2	5.4	1.72
Master Protein	Q9BRA2	Thioredoxin domain-containing protein 17 OS=Homo sapiens GN=TXNDC17 PE=1 SV=1	TXNDC17	Medium	FALSE	0.013	2.855	7	1	2	1	1	123	13.9	5.52	2.26
Master Protein	H3BP77	Thymidine kinase 2, mitochondrial OS=Homo sapiens GN=TK2 PE=4 SV=1	TK2	Medium	FALSE	0.047	2.04	2	1	10	1	1	282	32.9	8.46	20.59
Master Protein	Q12986	Transcriptional repressor NF-X1 OS=Homo sapiens GN=NFX1 PE=1 SV=2	NFX1	Medium	FALSE	0.029	2.421	1	1	714	1	1	1120	124.3	8.24	589.76
Master Protein	Q14956	Transmembrane glycoprotein NMB OS=Homo sapiens GN=GPNMB PE=1 SV=2	GPNMB	Medium	FALSE	0.027	2.493	2	1	10	1	1	572	63.9	6.64	18.02
Master Protein	P45974	Ubiquitin carboxyl-terminal hydrolase 5 OS=Homo sapiens GN=USP5 PE=1 SV=2	USP5	Medium	FALSE	0.011	3.177	1	1	2	1	1	858	95.7	5.03	3.97
Master Protein	Q9NYU2	UDP-glucose:glycoprotein glucosyltransferase 1 OS=Homo sapiens GN=UGGT1 PE=1 SV=3	UGGT1	Medium	FALSE	0.026	2.564	1	1	1	1	1	1555	177.1	5.63	0
Master Protein	Q96KN7	X-linked retinitis pigmentosa GTPase regulator-interacting protein 1 OS=Homo sapiens GN=RPGRIP1 PE=1 SV=2	RPGRIP1	Medium	FALSE	0.017	2.612	2	1	1	1	1	1286	146.6	5.68	0

† MS information on 902 quantified proteins is listed.

Abbreviations

MDD: major depressive disorder, BD: bipolar disorder, SCZ: schizophrenia, HC: healthy control

Table S13. List of differentially expressed proteins (DEPs) derived from proteomic profiling data for four groups (SCZ, BD, MDD, and HC)†

MDD vs BD vs HC

Accessions	Gene symbols	Protein names	ANOVA Significant	Clusters	-Log ANOVA p value	ANOVA P-value
C9JIF9	APEH	Acylamino-acid-releasing enzyme OS=Homo sapiens GN=APEH PE=1 SV=1	+	Cluster 1	1.477	0.033
P53990-5	IST1	Isoform 5 of IST1 homolog OS=Homo sapiens GN=IST1	+	Cluster 1	1.712	0.019
P02792	FTL	Ferritin light chain OS=Homo sapiens GN=FTL PE=1 SV=2	+	Cluster 1	2.999	0.001
Q9UJJ9	GNPTG	N-acetylglucosamine-1-phosphotransferase subunit gamma OS=Homo sapiens GN=GNPTG PE=1 SV=1	+	Cluster 1	1.426	0.038
P23142	FBLN1	Fibulin-1 OS=Homo sapiens GN=FBLN1 PE=1 SV=4	+	Cluster 1	1.438	0.036
P35916	FLT4	Vascular endothelial growth factor receptor 3 OS=Homo sapiens GN=FLT4 PE=1 SV=3	+	Cluster 1	1.891	0.013
P04278	SHBG	Sex hormone-binding globulin OS=Homo sapiens GN=SHBG PE=1 SV=2	+	Cluster 1	2.214	0.006
P02774-3	GC	Isoform 3 of Vitamin D-binding protein OS=Homo sapiens GN=GC	+	Cluster 1	2.603	0.002
P02765	AHSG	Alpha-2-HS-glycoprotein OS=Homo sapiens GN=AHSG PE=1 SV=1	+	Cluster 1	1.868	0.014
Q06033	ITIH3	Inter-alpha-trypsin inhibitor heavy chain H3 OS=Homo sapiens GN=ITIH3 PE=1 SV=2	+	Cluster 1	1.715	0.019
P01023	A2M	Alpha-2-macroglobulin OS=Homo sapiens GN=A2M PE=1 SV=3	+	Cluster 1	2.087	0.008
P24298	GPT	Alanine aminotransferase 1 OS=Homo sapiens GN=GPT PE=1 SV=3	+	Cluster 1	1.433	0.037
P19823	ITIH2	Inter-alpha-trypsin inhibitor heavy chain H2 OS=Homo sapiens GN=ITIH2 PE=1 SV=2	+	Cluster 1	3.505	< 0.001
P05160	F13B	Coagulation factor XIII B chain OS=Homo sapiens GN=F13B PE=1 SV=3	+	Cluster 1	2.660	0.002
Q70J99-3	UNC13D	Isoform 3 of Protein unc-13 homolog D OS=Homo sapiens GN=UNC13D	+	Cluster 1	2.216	0.006
Q13790	APOF	Apolipoprotein F OS=Homo sapiens GN=APOF PE=1 SV=2	+	Cluster 2	1.963	0.011
Q16706	MAN2A1	Alpha-mannosidase 2 OS=Homo sapiens GN=MAN2A1 PE=1 SV=2	+	Cluster 2	1.546	0.028
P02749	APOH	Beta-2-glycoprotein 1 OS=Homo sapiens GN=APOH PE=1 SV=3	+	Cluster 2	1.637	0.023
Q6UX71	PLXDC2	Plexin domain-containing protein 2 OS=Homo sapiens GN=PLXDC2 PE=1 SV=1	+	Cluster 2	1.505	0.031
P55268	LAMB2	Laminin subunit beta-2 OS=Homo sapiens GN=LAMB2 PE=1 SV=2	+	Cluster 2	2.577	0.003
Q96KN2	CNDP1	Beta-Ala-His dipeptidase OS=Homo sapiens GN=CNDP1 PE=1 SV=4	+	Cluster 2	2.456	0.003
P35908	KRT2	Keratin, type II cytoskeletal 2 epidermal OS=Homo sapiens GN=KRT2 PE=1 SV=2	+	Cluster 2	1.651	0.022
P02787	TF	Serotransferrin OS=Homo sapiens GN=TF PE=1 SV=3	+	Cluster 2	2.707	0.002
P04264	KRT1	Keratin, type II cytoskeletal 1 OS=Homo sapiens GN=KRT1 PE=1 SV=6	+	Cluster 2	4.689	< 0.001
P13645	KRT10	Keratin, type I cytoskeletal 10 OS=Homo sapiens GN=KRT10 PE=1 SV=6	+	Cluster 2	5.036	< 0.001
P01019	AGT	Angiotensinogen OS=Homo sapiens GN=AGT PE=1 SV=1	+	Cluster 2	2.110	0.008
P01889	HLA-B	HLA class I histocompatibility antigen, B-7 alpha chain OS=Homo sapiens GN=HLA-B PE=1 SV=3	+	Cluster 2	1.380	0.042
V9GYM3	APOA2	Apolipoprotein A-II OS=Homo sapiens GN=APOA2 PE=1 SV=1	+	Cluster 2	2.105	0.008
Q9Y251	HPSE	Heparanase OS=Homo sapiens GN=HPSE PE=1 SV=2	+	Cluster 2	1.419	0.038
P12273	PIP	Prolactin-inducible protein OS=Homo sapiens GN=PIP PE=1 SV=1	+	Cluster 2	1.655	0.022
Q96CX2	KCTD12	BTB/POZ domain-containing protein KCTD12 OS=Homo sapiens GN=KCTD12 PE=1 SV=1	+	Cluster 2	1.711	0.019
P04070-2	PROC	Isoform 2 of Vitamin K-dependent protein C OS=Homo sapiens GN=PROC	+	Cluster 2	2.063	0.009
G3XAI2	LAMB1	Laminin subunit beta-1 OS=Homo sapiens GN=LAMB1 PE=1 SV=1	+	Cluster 2	1.699	0.020
A0A087WVE2	FCN1	Ficolin-1 OS=Homo sapiens GN=FCN1 PE=4 SV=1	+	Cluster 2	2.991	0.001
B4DPQ0	C1R	Complement C1r subcomponent OS=Homo sapiens GN=C1R PE=2 SV=1	+	Cluster 2	4.254	< 0.001

MDD vs SCZ vs HC

Accessions	Gene symbols	Protein name	ANOVA Significant	Clusters	-Log ANOVA p value	ANOVA P-value
P06744-2	GPI	Isoform 2 of Glucose-6-phosphate isomerase OS=Homo sapiens GN=GPI	+	Cluster 1	1.605	0.025
P24666	ACP1	Low molecular weight phosphotyrosine protein phosphatase OS=Homo sapiens GN=ACP1 PE=1 SV=3	+	Cluster 1	1.366	0.043
Q03167	TGFBR3	Transforming growth factor beta receptor type 3 OS=Homo sapiens GN=TGFBR3 PE=1 SV=3	+	Cluster 1	1.304	0.050
Q07954	LRP1	Prolow-density lipoprotein receptor-related protein 1 OS=Homo sapiens GN=LRP1 PE=1 SV=2	+	Cluster 1	1.441	0.036
Q9UNW1	MINPP1	Multiple inositol polyphosphate phosphatase 1 OS=Homo sapiens GN=MINPP1 PE=1 SV=1	+	Cluster 1	1.501	0.032
Q96RD9	FCRL5	Fc receptor-like protein 5 OS=Homo sapiens GN=FCRL5 PE=1 SV=3	+	Cluster 1	2.151	0.007
P23470	PTPRG	Receptor-type tyrosine-protein phosphatase gamma OS=Homo sapiens GN=PTPRG PE=1 SV=4	+	Cluster 1	2.176	0.007
Q8WWM7-3	ATXN2L	Isoform 3 of Ataxin-2-like protein OS=Homo sapiens GN=ATXN2L	+	Cluster 1	1.639	0.023
P04196	HRG	Histidine-rich glycoprotein OS=Homo sapiens GN=HRG PE=1 SV=1	+	Cluster 1	1.842	0.014
O00194	RAB27B	Ras-related protein Rab-27B OS=Homo sapiens GN=RAB27B PE=1 SV=4	+	Cluster 1	1.786	0.016
P07358	C8B	Complement component C8 beta chain OS=Homo sapiens GN=C8B PE=1 SV=3	+	Cluster 1	1.953	0.011
P98160	HSPG2	Basement membrane-specific heparan sulfate proteoglycan core protein OS=Homo sapiens GN=HSPG2 PE=1 SV=4	+	Cluster 1	2.233	0.006
P06331		Ig heavy chain V-II region ARH-77 OS=Homo sapiens PE=4 SV=1	+	Cluster 1	1.905	0.012
Q15833-3	STXBP2	Isoform 3 of Syntaxin-binding protein 2 OS=Homo sapiens GN=STXBP2	+	Cluster 1	1.369	0.043
Q9NSB4	KRT82	Keratin, type II cuticular Hb2 OS=Homo sapiens GN=KRT82 PE=1 SV=3	+	Cluster 1	2.569	0.003
D6RE86	CP	Ceruloplasmin (Fragment) OS=Homo sapiens GN=CP PE=4 SV=3	+	Cluster 1	2.337	0.005
Q8NBP7	PCSK9	Proprotein convertase subtilisin/kexin type 9 OS=Homo sapiens GN=PCSK9 PE=1 SV=3	+	Cluster 1	4.285	< 0.001
P42574	CASP3	Caspase-3 OS=Homo sapiens GN=CASP3 PE=1 SV=2	+	Cluster 1	2.361	0.004
P10646	TFPI	Tissue factor pathway inhibitor OS=Homo sapiens GN=TFPI PE=1 SV=1	+	Cluster 1	2.043	0.009
A0A087WYC5	IGHG1	Ig gamma-1 chain C region OS=Homo sapiens GN=IGHG1 PE=1 SV=1	+	Cluster 1	2.594	0.003
O75636	FCN3	Ficolin-3 OS=Homo sapiens GN=FCN3 PE=1 SV=2	+	Cluster 1	1.673	0.021
P23280-2	CA6	Isoform 2 of Carbonic anhydrase 6 OS=Homo sapiens GN=CA6	+	Cluster 1	1.567	0.027
P51148-2	RAB5C	Isoform 2 of Ras-related protein Rab-5C OS=Homo sapiens GN=RAB5C	+	Cluster 1	1.352	0.044
P07225	PROS1	Vitamin K-dependent protein S OS=Homo sapiens GN=PROS1 PE=1 SV=1	+	Cluster 1	2.214	0.006
P05186	ALPL	Alkaline phosphatase, tissue-nonspecific isozyme OS=Homo sapiens GN=ALPL PE=1 SV=4	+	Cluster 1	1.822	0.015
P02654	APOC1	Apolipoprotein C-I OS=Homo sapiens GN=APOC1 PE=1 SV=1	+	Cluster 1	1.320	0.048
Q13508	ART3	Ecto-ADP-ribosyltransferase 3 OS=Homo sapiens GN=ART3 PE=1 SV=2	+	Cluster 2	1.612	0.024
P12955	PEPD	Xaa-Pro dipeptidase OS=Homo sapiens GN=PEPD PE=1 SV=3	+	Cluster 2	2.369	0.004
Q9Y2I7	PIKFYVE	1-phosphatidylinositol 3-phosphate 5-kinase OS=Homo sapiens GN=PIKFYVE PE=1 SV=3	+	Cluster 2	3.256	< 0.001
Q15555	MAPRE2	Microtubule-associated protein RP/EB family member 2 OS=Homo sapiens GN=MAPRE2 PE=1 SV=1	+	Cluster 2	1.574	0.027
O00748	CES2	Cocaine esterase OS=Homo sapiens GN=CES2 PE=1 SV=1	+	Cluster 2	2.056	0.009
P02774-3	GC	Isoform 3 of Vitamin D-binding protein OS=Homo sapiens GN=GC	+	Cluster 2	2.921	0.001
P04278	SHBG	Sex hormone-binding globulin OS=Homo sapiens GN=SHBG PE=1 SV=2	+	Cluster 2	3.340	< 0.001
P02533	KRT14	Keratin, type I cytoskeletal 14 OS=Homo sapiens GN=KRT14 PE=1 SV=4	+	Cluster 2	2.096	0.008
M0R2J8	DCDC1	Doublecortin domain-containing protein 1 OS=Homo sapiens GN=DCDC1 PE=4 SV=1	+	Cluster 2	1.515	0.031

BD vs SCZ vs HC

Accessions	Gene symbol	Protein name	ANOVA Significant	Clusters	-Log ANOVA p value	ANOVA P-value
P16070	CD44	CD44 antigen OS=Homo sapiens GN=CD44 PE=1 SV=3	+	Cluster 1	1.554	0.028
A0A075B6R9	IGKV2D-24	Protein IGKV2D-24 (Fragment) OS=Homo sapiens GN=IGKV2D-24 PE=4 SV=1	+	Cluster 1	2.633	0.002
G8JLH6	CD9	Tetraspanin (Fragment) OS=Homo sapiens GN=CD9 PE=1 SV=1	+	Cluster 1	2.048	0.009
P13727	PRG2	Bone marrow proteoglycan OS=Homo sapiens GN=PRG2 PE=1 SV=2	+	Cluster 1	1.475	0.033
Q14624	ITIH4	Inter-alpha-trypsin inhibitor heavy chain H4 OS=Homo sapiens GN=ITIH4 PE=1 SV=4	+	Cluster 1	1.780	0.017
P02787	TF	Serotransferrin OS=Homo sapiens GN=TF PE=1 SV=3	+	Cluster 1	4.242	< 0.001
P04075-2	ALDOA	Isoform 2 of Fructose-bisphosphate aldolase A OS=Homo sapiens GN=ALDOA	+	Cluster 1	2.561	0.003
A0A075B6I8	IGLV1-47	Protein IGLV1-47 (Fragment) OS=Homo sapiens GN=IGLV1-47 PE=4 SV=1	+	Cluster 1	3.703	< 0.001
Q9BUN1	MENT	Protein MENT OS=Homo sapiens GN=MENT PE=2 SV=1	+	Cluster 1	1.437	0.037
B7ZKJ8	ITIH4	ITIH4 protein OS=Homo sapiens GN=ITIH4 PE=1 SV=1	+	Cluster 1	2.452	0.004
Q6UX71	PLXDC2	Plexin domain-containing protein 2 OS=Homo sapiens GN=PLXDC2 PE=1 SV=1	+	Cluster 1	1.692	0.020
Q9HBW9	ELTD1	EGF, latrophilin and seven transmembrane domain-containing protein 1 OS=Homo sapiens GN=ELTD1 PE=1 SV=3	+	Cluster 1	2.317	0.005
P13598	ICAM2	Intercellular adhesion molecule 2 OS=Homo sapiens GN=ICAM2 PE=1 SV=2	+	Cluster 1	2.475	0.003
Q8TD57	DNAH3	Dynein heavy chain 3, axonemal OS=Homo sapiens GN=DNAH3 PE=2 SV=1	+	Cluster 1	2.758	0.002
Q96KN2	CNDP1	Beta-Ala-His dipeptidase OS=Homo sapiens GN=CNDP1 PE=1 SV=4	+	Cluster 1	2.992	0.001
P11597	CETP	Cholesteryl ester transfer protein OS=Homo sapiens GN=CETP PE=1 SV=2	+	Cluster 1	2.609	0.002
Q9H8L6	MMRN2	Multimerin-2 OS=Homo sapiens GN=MMRN2 PE=1 SV=2	+	Cluster 1	3.240	< 0.001
Q9NZP8	C1RL	Complement C1r subcomponent-like protein OS=Homo sapiens GN=C1RL PE=1 SV=2	+	Cluster 1	2.757	0.002
Q8IUK5	PLXDC1	Plexin domain-containing protein 1 OS=Homo sapiens GN=PLXDC1 PE=1 SV=2	+	Cluster 1	1.597	0.025
Q15833-3	STXBP2	Isoform 3 of Syntaxin-binding protein 2 OS=Homo sapiens GN=STXBP2	+	Cluster 1	1.335	0.046
P22352	GPX3	Glutathione peroxidase 3 OS=Homo sapiens GN=GPX3 PE=1 SV=2	+	Cluster 1	1.365	0.043
P02647	APOA1	Apolipoprotein A-I OS=Homo sapiens GN=APOA1 PE=1 SV=1	+	Cluster 1	1.591	0.026
V9GYM3	APOA2	Apolipoprotein A-II OS=Homo sapiens GN=APOA2 PE=1 SV=1	+	Cluster 1	1.370	0.043
F5H6S5	C1RL	Complement C1r subcomponent-like protein (Fragment) OS=Homo sapiens GN=C1RL PE=4 SV=3	+	Cluster 1	1.473	0.034
P02774-3	GC	Isoform 3 of Vitamin D-binding protein OS=Homo sapiens GN=GC	+	Cluster 1	3.585	< 0.001
P27169	PON1	Serum paraoxonase/arylesterase 1 OS=Homo sapiens GN=PON1 PE=1 SV=3	+	Cluster 1	1.383	0.041
P04278	SHBG	Sex hormone-binding globulin OS=Homo sapiens GN=SHBG PE=1 SV=2	+	Cluster 1	3.984	< 0.001
P13667	PDIA4	Protein disulfide-isomerase A4 OS=Homo sapiens GN=PDIA4 PE=1 SV=2	+	Cluster 1	3.942	< 0.001
P08185	SERPINA6	Corticosteroid-binding globulin OS=Homo sapiens GN=SERPINA6 PE=1 SV=1	+	Cluster 1	2.539	0.003
Q9H7P6	MVB12B	Multivesicular body subunit 12B OS=Homo sapiens GN=MVB12B PE=1 SV=2	+	Cluster 1	2.730	0.002
P01023	A2M	Alpha-2-macroglobulin OS=Homo sapiens GN=A2M PE=1 SV=3	+	Cluster 1	4.694	< 0.001
P02792	FTL	Ferritin light chain OS=Homo sapiens GN=FTL PE=1 SV=2	+	Cluster 1	2.326	0.005
P01717		Ig lambda chain V-IV region Hil OS=Homo sapiens PE=1 SV=1	+	Cluster 1	2.244	0.006
P0C0L4	C4A	Complement C4-A OS=Homo sapiens GN=C4A PE=1 SV=2	+	Cluster 1	1.483	0.033
Q7Z3B1	NEGR1	Neuronal growth regulator 1 OS=Homo sapiens GN=NEGR1 PE=1 SV=3	+	Cluster 1	1.782	0.017

P02786	TFRC	Transferrin receptor protein 1 OS=Homo sapiens GN=TFRC PE=1 SV=2	+	Cluster 2	2.304	0.005	P01765		Ig heavy chain V-III region TIL OS=Homo sapiens PE=1 SV=1	+	Cluster 2	1.709	0.020	P02533	KRT14	Keratin, type I cytoskeletal 14 OS=Homo sapiens GN=KRT14 PE=1 SV=4	+	Cluster 1	2.228	0.006
O95445	APOM	Apolipoprotein M OS=Homo sapiens GN=APOM PE=1 SV=2	+	Cluster 2	1.620	0.024	P04075-2	ALDOA	Isoform 2 of Fructose-bisphosphate aldolase A OS=Homo sapiens GN=ALDOA	+	Cluster 2	3.105	< 0.001	P12955	PEPD	Xaa-Pro dipeptidase OS=Homo sapiens GN=PEPD PE=1 SV=3	+	Cluster 1	1.608	0.025
P16035	TIMP2	Metalloproteinase inhibitor 2 OS=Homo sapiens GN=TIMP2 PE=1 SV=2	+	Cluster 2	2.385	0.004	A0A075B6I0	IGLV8-61	Protein IGLV8-61 (Fragment) OS=Homo sapiens GN=IGLV8-61 PE=4 SV=2	+	Cluster 2	2.446	0.004	Q06033	ITIH3	Inter-alpha-trypsin inhibitor heavy chain H3 OS=Homo sapiens GN=ITIH3 PE=1 SV=2	+	Cluster 1	2.208	0.006
P02747	C1QC	Complement C1q subcomponent subunit C OS=Homo sapiens GN=C1QC PE=1 SV=3	+	Cluster 2	2.645	0.002	Q06033	ITIH3	Inter-alpha-trypsin inhibitor heavy chain H3 OS=Homo sapiens GN=ITIH3 PE=1 SV=2	+	Cluster 2	2.327	0.005	M0R2J8	DCDC1	Doublecortin domain-containing protein 1 OS=Homo sapiens GN=DCDC1 PE=4 SV=1	+	Cluster 1	2.813	0.002
P13667	PDIA4	Protein disulfide-isomerase A4 OS=Homo sapiens GN=PDIA4 PE=1 SV=2	+	Cluster 2	2.602	0.003	P11597	CETP	Cholesteryl ester transfer protein OS=Homo sapiens GN=CETP PE=1 SV=2	+	Cluster 2	3.670	< 0.001	P00533	EGFR	Epidermal growth factor receptor OS=Homo sapiens GN=EGFR PE=1 SV=2	+	Cluster 1	2.358	0.004
P05019	IGF1	Insulin-like growth factor I OS=Homo sapiens GN=IGF1 PE=1 SV=1	+	Cluster 2	2.274	0.005	Q9H8L6	MMRN2	Multimerin-2 OS=Homo sapiens GN=MMRN2 PE=1 SV=2	+	Cluster 2	3.653	< 0.001	B4DPQ0	C1R	Complement C1r subcomponent OS=Homo sapiens GN=C1R PE=2 SV=1	+	Cluster 1	5.107	< 0.001
Q8WZ75	ROBO4	Roundabout homolog 4 OS=Homo sapiens GN=ROBO4 PE=1 SV=1	+	Cluster 3	1.332	0.047	P19823	ITIH2	Inter-alpha-trypsin inhibitor heavy chain H2 OS=Homo sapiens GN=ITIH2 PE=1 SV=2	+	Cluster 2	2.697	0.002	P02786	TFRC	Transferrin receptor protein 1 OS=Homo sapiens GN=TFRC PE=1 SV=2	+	Cluster 1	2.479	0.003
F5H2F4	MTHFD1	C-1-tetrahydrofolate synthase, cytoplasmic OS=Homo sapiens GN=MTHFD1 PE=1 SV=1	+	Cluster 3	1.641	0.023	P02787	TF	Serotransferrin OS=Homo sapiens GN=TF PE=1 SV=3	+	Cluster 2	4.114	< 0.001	O95445	APOM	Apolipoprotein M OS=Homo sapiens GN=APOM PE=1 SV=2	+	Cluster 1	2.672	0.002
Q02818	NUCB1	Nucleobindin-1 OS=Homo sapiens GN=NUCB1 PE=1 SV=4	+	Cluster 3	1.399	0.040	Q14624	ITIH4	Inter-alpha-trypsin inhibitor heavy chain H4 OS=Homo sapiens GN=ITIH4 PE=1 SV=4	+	Cluster 2	2.403	0.004	Q16270	IGFBP7	Insulin-like growth factor-binding protein 7 OS=Homo sapiens GN=IGFBP7 PE=1 SV=1	+	Cluster 2	1.638	0.023
O14498	ISLR	Immunoglobulin superfamily containing leucine-rich repeat protein OS=Homo sapiens GN=ISLR PE=1 SV=1	+	Cluster 3	1.802	0.016	Q9HBW9	ELTD1	EGF, latrophilin and seven transmembrane domain-containing protein 1 OS=Homo sapiens GN=ELTD1 PE=1 SV=3	+	Cluster 2	2.328	0.005	A0A075B7B8	IGHV3OR16-12	Protein IGHV3OR16-12 (Fragment) OS=Homo sapiens GN=IGHV3OR16-12 PE=4 SV=1	+	Cluster 2	1.528	0.030
P35527	KRT9	Keratin, type I cytoskeletal 9 OS=Homo sapiens GN=KRT9 PE=1 SV=3	+	Cluster 3	2.144	0.007	P01023	A2M	Alpha-2-macroglobulin OS=Homo sapiens GN=A2M PE=1 SV=3	+	Cluster 2	5.316	< 0.001	P04275	VWF	von Willebrand factor OS=Homo sapiens GN=VWF PE=1 SV=4	+	Cluster 2	1.848	0.014
P02144	MB	Myoglobin OS=Homo sapiens GN=MB PE=1 SV=2	+	Cluster 3	3.882	< 0.001	A0A075B6I8	IGLV1-47	Protein IGLV1-47 (Fragment) OS=Homo sapiens GN=IGLV1-47 PE=4 SV=1	+	Cluster 2	3.908	< 0.001	O00187	MASP2	Mannan-binding lectin serine protease 2 OS=Homo sapiens GN=MASP2 PE=1 SV=4	+	Cluster 2	1.839	0.015
A0A087WXI5	CDH1	Cadherin-1 OS=Homo sapiens GN=CDH1 PE=1 SV=1	+	Cluster 3	2.039	0.009	B7ZKJ8	ITIH4	ITIH4 protein OS=Homo sapiens GN=ITIH4 PE=1 SV=1	+	Cluster 2	3.060	< 0.001	Q08830	FGL1	Fibrinogen-like protein 1 OS=Homo sapiens GN=FGL1 PE=1 SV=3	+	Cluster 2	1.552	0.028
P29622	SERPINA4	Kallistatin OS=Homo sapiens GN=SERPINA4 PE=1 SV=3	+	Cluster 3	2.450	0.004	Q8TD57	DNAH3	Dynein heavy chain 3, axonemal OS=Homo sapiens GN=DNAH3 PE=2 SV=1	+	Cluster 2	3.672	< 0.001	P24298	GPT	Alanine aminotransferase 1 OS=Homo sapiens GN=GPT PE=1 SV=3	+	Cluster 2	2.110	0.008
P12109	COL6A1	Collagen alpha-1(VI) chain OS=Homo sapiens GN=COL6A1 PE=1 SV=3	+	Cluster 3	1.571	0.027	P02792	FTL	Ferritin light chain OS=Homo sapiens GN=FTL PE=1 SV=2	+	Cluster 2	2.996	0.001	P02765	AHSG	Alpha-2-HS-glycoprotein OS=Homo sapiens GN=AHSG PE=1 SV=1	+	Cluster 2	1.984	0.010
P13929	ENO3	Beta-enolase OS=Homo sapiens GN=ENO3 PE=1 SV=5	+	Cluster 3	2.305	0.005	P13489	RNH1	Ribonuclease inhibitor OS=Homo sapiens GN=RNH1 PE=1 SV=2	+	Cluster 2	2.344	0.005	P02671	FGA	Fibrinogen alpha chain OS=Homo sapiens GN=FGA PE=1 SV=2	+	Cluster 2	3.296	< 0.001
A0A087WYJ9	IGHM	Ig mu chain C region OS=Homo sapiens GN=IGHM PE=1 SV=1	+	Cluster 3	1.587	0.026	G8JLH6	CD9	Tetraspanin (Fragment) OS=Homo sapiens GN=CD9 PE=1 SV=1	+	Cluster 2	2.002	0.010	P39060	COL18A1	Collagen alpha-1(XVIII) chain OS=Homo sapiens GN=COL18A1 PE=1 SV=5	+	Cluster 2	1.677	0.021
P26447	S100A4	Protein S100-A4 OS=Homo sapiens GN=S100A4 PE=1 SV=1	+	Cluster 3	1.358	0.044	P13727	PRG2	Bone marrow proteoglycan OS=Homo sapiens GN=PRG2 PE=1 SV=2	+	Cluster 2	2.065	0.009	Q12986	NFX1	Transcriptional repressor NF-X1 OS=Homo sapiens GN=NFX1 PE=1 SV=2	+	Cluster 2	1.341	0.046
P35858-2	IGFALS	Isoform 2 of Insulin-like growth factor-binding protein complex acid labile subunit OS=Homo sapiens GN=IGFALS	+	Cluster 3	1.425	0.038	P16070	CD44	CD44 antigen OS=Homo sapiens GN=CD44 PE=1 SV=3	+	Cluster 2	1.742	0.018	P02747	C1QC	Complement C1q subcomponent subunit C OS=Homo sapiens GN=C1QC PE=1 SV=3	+	Cluster 2	2.420	0.004
P43652	AFM	Afamin OS=Homo sapiens GN=AFM PE=1 SV=1	+	Cluster 3	1.873	0.013	A0A075B6R9	IGKV2D-24	Protein IGKV2D-24 (Fragment) OS=Homo sapiens GN=IGKV2D-24 PE=4 SV=1	+	Cluster 2	2.363	0.004	P02675	FGB	Fibrinogen beta chain OS=Homo sapiens GN=FGB PE=1 SV=2	+	Cluster 2	1.418	0.038
A0A087WX49	IGLL5	Immunoglobulin lambda-like polypeptide 5 OS=Homo sapiens GN=IGLL5 PE=4 SV=1	+	Cluster 3	2.307	0.005	Q9BUN1	MENT	Protein MENT OS=Homo sapiens GN=MENT PE=2 SV=1	+	Cluster 2	2.046	0.009	P22897	MRC1	Macrophage mannose receptor 1 OS=Homo sapiens GN=MRC1 PE=1 SV=1	+	Cluster 2	1.706	0.020
P80108	GPLD1	Phosphatidylinositol-glycan-specific phospholipase D OS=Homo sapiens GN=GPLD1 PE=1 SV=3	+	Cluster 3	2.687	0.002	P13598	ICAM2	Intercellular adhesion molecule 2 OS=Homo sapiens GN=ICAM2 PE=1 SV=2	+	Cluster 2	1.741	0.018	P05160	F13B	Coagulation factor XIII B chain OS=Homo sapiens GN=F13B PE=1 SV=3	+	Cluster 2	3.272	< 0.001
P11217	PYGM	Glycogen phosphorylase, muscle form OS=Homo sapiens GN=PYGM PE=1 SV=6	+	Cluster 3	3.524	< 0.001	Q14974	KPNB1	Importin subunit beta-1 OS=Homo sapiens GN=KPNB1 PE=1 SV=2	+	Cluster 2	1.584	0.026	Q9BXR6	CFHR5	Complement factor H-related protein 5 OS=Homo sapiens GN=CFHR5 PE=1 SV=1	+	Cluster 2	2.105	0.008
P06732	CKM	Creatine kinase M-type OS=Homo sapiens GN=CKM PE=1 SV=2	+	Cluster 3	4.790	< 0.001	K4DIA0	ICOSLG	ICOS ligand OS=Homo sapiens GN=ICOSLG PE=4 SV=1	+	Cluster 2	1.369	0.043	P02679-2	FGG	Isoform Gamma-A of Fibrinogen gamma chain OS=Homo sapiens GN=FGG	+	Cluster 2	2.543	0.003
A0A075B6L0	IGLC3	Ig lambda-3 chain C regions (Fragment) OS=Homo sapiens GN=IGLC3 PE=4 SV=2	+	Cluster 3	2.112	0.008	P02786	TFRC	Transferrin receptor protein 1 OS=Homo sapiens GN=TFRC PE=1 SV=2	+	Cluster 2	2.141	0.007	P02749	APOH	Beta-2-glycoprotein 1 OS=Homo sapiens GN=APOH PE=1 SV=3	+	Cluster 2	2.351	0.004
A0A087X130	IGKC	Ig kappa chain C region OS=Homo sapiens GN=IGKC PE=4 SV=1	+	Cluster 3	2.861	0.001	O95445	APOM	Apolipoprotein M OS=Homo sapiens GN=APOM PE=1 SV=2	+	Cluster 2	1.960	0.011	P53990-5	IST1	Isoform 5 of IST1 homolog OS=Homo sapiens GN=IST1	+	Cluster 2	2.132	0.007
P69891	HBG1	Hemoglobin subunit gamma-1 OS=Homo sapiens GN=HBG1 PE=1 SV=2	+	Cluster 3	2.141	0.007	P02647	APOA1	Apolipoprotein A-I OS=Homo sapiens GN=APOA1 PE=1 SV=1	+	Cluster 2	1.491	0.032	Q16610	ECM1	Extracellular matrix protein 1 OS=Homo sapiens GN=ECM1 PE=1 SV=2	+	Cluster 2	2.332	0.005
O75915	ARL6IP5	PRA1 family protein 3 OS=Homo sapiens GN=ARL6IP5 PE=1 SV=1	+	Cluster 3	1.371	0.043	P01717		Ig lambda chain V-IV region Hil OS=Homo sapiens PE=1 SV=1	+	Cluster 2	1.561	0.027	C9JPV4	SERPINF2	Alpha-2-antiplasmin (Fragment) OS=Homo sapiens GN=SERPINF2 PE=3 SV=1	+	Cluster 2	2.750	0.002
O15394	NCAM2	Neural cell adhesion molecule 2 OS=Homo sapiens GN=NCAM2 PE=1 SV=2	+	Cluster 3	2.728	0.002	G3XAK1	MST1	Hepatocyte growth factor-like protein alpha chain OS=Homo sapiens GN=MST1 PE=3 SV=1	+	Cluster 2	1.672	0.021	P19823	ITIH2	Inter-alpha-trypsin inhibitor heavy chain H2 OS=Homo sapiens GN=ITIH2 PE=1 SV=2	+	Cluster 2	3.295	< 0.001
P23526	AHCY	Adenosylhomocysteinase OS=Homo sapiens GN=AHCY PE=1 SV=4	+	Cluster 3	1.367	0.043	P08697	SERPINF2	Alpha-2-antiplasmin OS=Homo sapiens GN=SERPINF2 PE=1 SV=3	+	Cluster 2	1.308	0.049	A0A087WVE2	FCN1	Ficolin-1 OS=Homo sapiens GN=FCN1 PE=4 SV=1	+	Cluster 2	2.718	0.002
Q15084-2	PDIA6	Isoform 2 of Protein disulfide-isomerase A6 OS=Homo sapiens GN=PDIA6	+	Cluster 3	1.664	0.022	Q9H7P6	MVB12B	Multivesicular body subunit 12B OS=Homo sapiens GN=MVB12B PE=1 SV=2	+	Cluster 2	2.088	0.008	P19827	ITIH1	Inter-alpha-trypsin inhibitor heavy chain H1 OS=Homo sapiens GN=ITIH1 PE=1 SV=3	+	Cluster 2	1.364	0.043
P01137	TGFB1	Transforming growth factor beta-1 OS=Homo sapiens GN=TGFB1 PE=1 SV=2	+	Cluster 3	1.423	0.038	Q96KN2	CNDP1	Beta-Ala-His dipeptidase OS=Homo sapiens GN=CNDP1 PE=1 SV=4	+	Cluster 2	2.476	0.003	P04070-2	PROC	Isoform 2 of Vitamin K-dependent protein C OS=Homo sapiens GN=PROC	+	Cluster 2	1.614	0.024
P49641	MAN2A2	Alpha-mannosidase 2x OS=Homo sapiens GN=MAN2A2 PE=2 SV=3	+	Cluster 4	1.311	0.049	P13667	PDIA4	Protein disulfide-isomerase A4 OS=Homo sapiens GN=PDIA4 PE=1 SV=2	+	Cluster 2	3.834	< 0.001	Q09666	AHNAK	Neuroblast differentiation-associated protein AHNAK OS=Homo sapiens GN=AHNAK PE=1 SV=2	+	Cluster 2	1.381	0.042
P19652	ORM2	Alpha-1-acid glycoprotein 2 OS=Homo sapiens GN=ORM2 PE=1 SV=2	+	Cluster 4	1.446	0.036	B4DPQ0	C1R	Complement C1r subcomponent OS=Homo sapiens GN=C1R PE=2 SV=1	+	Cluster 2	4.731	< 0.001	C9JF17	APOD	Apolipoprotein D (Fragment) OS=Homo sapiens GN=APOD PE=4 SV=1	+	Cluster 2	1.330	0.047
P01591	IGJ	Immunoglobulin J chain OS=Homo sapiens GN=IGJ PE=1 SV=4	+	Cluster 4	1.450	0.035	P08185	SERPINA6	Corticosteroid-binding globulin OS=Homo sapiens GN=SERPINA6 PE=1 SV=1	+	Cluster 2	1.799	0.016	Q70J99-3	UNC13D	Isoform 3 of Protein unc-13 homolog D OS=Homo sapiens GN=UNC13D	+	Cluster 2	1.929	0.012
Q14766-4	LTBP1	Isoform 4 of Latent-transforming growth factor beta-binding protein 1 OS=Homo sapiens GN=LTBP1	+	Cluster 4	1.658	0.022	Q9BXR6	CFHR5	Complement factor H-related protein 5 OS=Homo sapiens GN=CFHR5 PE=1 SV=1	+	Cluster 3	1.898	0.013	P16035	TIMP2	Metalloproteinase inhibitor 2 OS=Homo sapiens GN=TIMP2 PE=1 SV=2	+	Cluster 2	2.035	0.009
Q9NZK5	CECR1	Adenosine deaminase CECR1 OS=Homo sapiens GN=CECR1 PE=1 SV=2	+	Cluster 4	1.459	0.035	M0QXZ6	GMFG	Glia maturation factor gamma (Fragment) OS=Homo sapiens GN=GMFG PE=4 SV=1	+	Cluster 3	1.483	0.033	P00450	CP	Ceruloplasmin OS=Homo sapiens GN=CP PE=1 SV=1	+	Cluster 2	1.543	0.029
P18669	PGAM1	Phosphoglycerate mutase 1 OS=Homo sapiens GN=PGAM1 PE=1 SV=2	+	Cluster 4	2.114	0.008	O75563	SKAP2	Src kinase-associated phosphoprotein 2 OS=Homo sapiens GN=SKAP2 PE=1 SV=1	+	Cluster 3	1.976	0.011	Q13790	APOF	Apolipoprotein F OS=Homo sapiens GN=APOF PE=1 SV=2	+	Cluster 2	1.839	0.014
P05362	ICAM1	Intercellular adhesion molecule 1 OS=Homo sapiens GN=ICAM1 PE=1 SV=2	+	Cluster 4	1.675	0.021	P02144	MB	Myoglobin OS=Homo sapiens GN=MB PE=1 SV=2	+	Cluster 3	1.387	0.041	P81605-2	DCD	Isoform 2 of Dermcidin OS=Homo sapiens GN=DCD	+	Cluster 3	1.699	0.020
P05109	S100A8	Protein S100-A8 OS=Homo sapiens GN=S100A8 PE=1 SV=1	+	Cluster 4	1.841	0.014	Q16610	ECM1	Extracellular matrix protein 1 OS=Homo sapiens GN=ECM1 PE=1 SV=2	+	Cluster 3	2.426	0.004	P05452	CLEC3B	Tetranectin OS=Homo sapiens GN=CLEC3B PE=1 SV=3	+	Cluster 3	2.506	0.003

P42574	CASP3	Caspase-3 OS=Homo sapiens GN=CASP3 PE=1 SV=2	+	Cluster 4	1.343	0.045	A0A087WXI5	CDH1	Cadherin-1 OS=Homo sapiens GN=CDH1 PE=1 SV=1	+	Cluster 3	1.360	0.044	P43652	AFM	Afamin OS=Homo sapiens GN=AFM PE=1 SV=1	+	Cluster 3	1.784	0.016
Q05682	CALD1	Caldesmon OS=Homo sapiens GN=CALD1 PE=1 SV=3	+	Cluster 4	2.590	0.003	P09104	ENO2	Gamma-enolase OS=Homo sapiens GN=ENO2 PE=1 SV=3	+	Cluster 3	2.255	0.006	P02788	LTF	Lactotransferrin OS=Homo sapiens GN=LTF PE=1 SV=6	+	Cluster 3	1.688	0.020
P23470	PTPRG	Receptor-type tyrosine-protein phosphatase gamma OS=Homo sapiens GN=PTPRG PE=1 SV=4	+	Cluster 4	1.418	0.038	P02679-2	FGG	Isoform Gamma-A of Fibrinogen gamma chain OS=Homo sapiens GN=FGG	+	Cluster 3	3.995	< 0.001	E9PK25	CFL1	Cofilin-1 OS=Homo sapiens GN=CFL1 PE=1 SV=1	+	Cluster 3	2.418	0.004
P0C0L5	C4B	Complement C4-B OS=Homo sapiens GN=C4B PE=1 SV=2	+	Cluster 4	1.863	0.014	P36980	CFHR2	Complement factor H-related protein 2 OS=Homo sapiens GN=CFHR2 PE=1 SV=1	+	Cluster 3	3.014	< 0.001	A0A087X130	IGKC	Ig kappa chain C region OS=Homo sapiens GN=IGKC PE=4 SV=1	+	Cluster 3	3.347	< 0.001
P15531-2	NME1	Isoform 2 of Nucleoside diphosphate kinase A OS=Homo sapiens GN=NME1	+	Cluster 4	3.039	< 0.001	P0DJI9	SAA2	Serum amyloid A-2 protein OS=Homo sapiens GN=SAA2 PE=1 SV=1	+	Cluster 3	4.068	< 0.001	P55786	NPEPPS	Puromycin-sensitive aminopeptidase OS=Homo sapiens GN=NPEPPS PE=1 SV=2	+	Cluster 3	3.116	< 0.001
Q07960	ARHGAP1	Rho GTPase-activating protein 1 OS=Homo sapiens GN=ARHGAP1 PE=1 SV=1	+	Cluster 4	2.176	0.007	P02760	AMBP	Protein AMBP OS=Homo sapiens GN=AMBP PE=1 SV=1	+	Cluster 3	4.136	< 0.001	Q9NUQ9	FAM49B	Protein FAM49B OS=Homo sapiens GN=FAM49B PE=1 SV=1	+	Cluster 3	1.534	0.029
Q8IZF2	GPR116	Probable G-protein coupled receptor 116 OS=Homo sapiens GN=GPR116 PE=1 SV=3	+	Cluster 4	1.839	0.014	Q8NI99	ANGPTL6	Angiopoietin-related protein 6 OS=Homo sapiens GN=ANGPTL6 PE=1 SV=1	+	Cluster 3	1.460	0.035	H0YMD1	LDLR	Low-density lipoprotein receptor OS=Homo sapiens GN=LDLR PE=1 SV=1	+	Cluster 3	1.781	0.017
P61088	UBE2N	Ubiquitin-conjugating enzyme E2 N OS=Homo sapiens GN=UBE2N PE=1 SV=1	+	Cluster 4	3.147	< 0.001	Q8WZ75	ROBO4	Roundabout homolog 4 OS=Homo sapiens GN=ROBO4 PE=1 SV=1	+	Cluster 3	1.448	0.036	P04745	AMY1A	Alpha-amylase 1 OS=Homo sapiens GN=AMY1A PE=1 SV=2	+	Cluster 3	2.066	0.009
P01880-2	IGHD	Isoform 2 of Ig delta chain C region OS=Homo sapiens GN=IGHD	+	Cluster 4	2.871	0.001	C9JU00	FGG	Fibrinogen gamma chain (Fragment) OS=Homo sapiens GN=FGG PE=1 SV=1	+	Cluster 3	1.420	0.038	O95897	OLFM2	Noelin-2 OS=Homo sapiens GN=OLFM2 PE=2 SV=2	+	Cluster 3	1.827	0.015
P20851	C4BPB	C4b-binding protein beta chain OS=Homo sapiens GN=C4BPB PE=1 SV=1	+	Cluster 4	3.192	< 0.001	P07437	TUBB	Tubulin beta chain OS=Homo sapiens GN=TUBB PE=1 SV=2	+	Cluster 3	1.593	0.026	Q14623	IHH	Indian hedgehog protein OS=Homo sapiens GN=IHH PE=1 SV=4	+	Cluster 3	1.396	0.040
P16520	GNB3	Guanine nucleotide-binding protein G(I)/G(S)/G(T) subunit beta-3 OS=Homo sapiens GN=GNB3 PE=1 SV=1	+	Cluster 4	2.095	0.008	F5H2F4	MTHFD1	C-1-tetrahydrofolate synthase, cytoplasmic OS=Homo sapiens GN=MTHFD1 PE=1 SV=1	+	Cluster 3	1.988	0.010	P02766	TTR	Transthyretin OS=Homo sapiens GN=TTR PE=1 SV=1	+	Cluster 3	1.324	0.047
O14745	SLC9A3R1	Na(+)/H(+) exchange regulatory cofactor NHE-RF1 OS=Homo sapiens GN=SLC9A3R1 PE=1 SV=4	+	Cluster 4	3.180	< 0.001	Q92954	PRG4	Proteoglycan 4 OS=Homo sapiens GN=PRG4 PE=1 SV=2	+	Cluster 3	2.094	0.008	Q9UBG0	MRC2	C-type mannose receptor 2 OS=Homo sapiens GN=MRC2 PE=1 SV=2	+	Cluster 3	1.407	0.039
K7ELL7	PRKCSH	Glucosidase 2 subunit beta OS=Homo sapiens GN=PRKCSH PE=1 SV=1	+	Cluster 4	2.363	0.004	E7ETH0	CFI	Complement factor I light chain OS=Homo sapiens GN=CFI PE=3 SV=1	+	Cluster 3	1.456	0.035	Q9Y6Z7	COLEC10	Collectin-10 OS=Homo sapiens GN=COLEC10 PE=2 SV=2	+	Cluster 3	1.676	0.021
P18206	VCL	Vinculin OS=Homo sapiens GN=VCL PE=1 SV=4	+	Cluster 4	4.820	< 0.001	P48506	GCLC	Glutamate--cysteine ligase catalytic subunit OS=Homo sapiens GN=GCLC PE=1 SV=2	+	Cluster 3	1.324	0.047	Q8WWM7-3	ATXN2L	Isoform 3 of Ataxin-2-like protein OS=Homo sapiens GN=ATXN2L	+	Cluster 3	2.365	0.004
P68036	UBE2L3	Ubiquitin-conjugating enzyme E2 L3 OS=Homo sapiens GN=UBE2L3 PE=1 SV=1	+	Cluster 4	4.616	< 0.001	P01024	C3	Complement C3 OS=Homo sapiens GN=C3 PE=1 SV=2	+	Cluster 3	1.614	0.024	P80108	GPLD1	Phosphatidylinositol-glycan-specific phospholipase D OS=Homo sapiens GN=GPLD1 PE=1 SV=3	+	Cluster 3	1.490	0.032
P02775	PPBP	Platelet basic protein OS=Homo sapiens GN=PPBP PE=1 SV=3	+	Cluster 4	3.650	< 0.001	P09960	LTA4H	Leukotriene A-4 hydrolase OS=Homo sapiens GN=LTA4H PE=1 SV=2	+	Cluster 3	1.338	0.046	P13929	ENO3	Beta-enolase OS=Homo sapiens GN=ENO3 PE=1 SV=5	+	Cluster 3	2.226	0.006
P61204	ARF3	ADP-ribosylation factor 3 OS=Homo sapiens GN=ARF3 PE=1 SV=2	+	Cluster 4	2.852	0.001	P06703	S100A6	Protein S100-A6 OS=Homo sapiens GN=S100A6 PE=1 SV=1	+	Cluster 3	1.464	0.034	P08571	CD14	Monocyte differentiation antigen CD14 OS=Homo sapiens GN=CD14 PE=1 SV=2	+	Cluster 3	1.840	0.014
D6RE86	CP	Ceruloplasmin (Fragment) OS=Homo sapiens GN=CP PE=4 SV=3	+	Cluster 4	2.575	0.003	P09493-8	TPM1	Isoform 8 of Tropomyosin alpha-1 chain OS=Homo sapiens GN=TPM1	+	Cluster 3	1.748	0.018	P29622	SERPINA4	Kallistatin OS=Homo sapiens GN=SERPINA4 PE=1 SV=3	+	Cluster 3	3.258	< 0.001
O15143	ARPC1B	Actin-related protein 2/3 complex subunit 1B OS=Homo sapiens GN=ARPC1B PE=1 SV=3	+	Cluster 4	2.927	0.001	P23526	AHCY	Adenosylhomocysteinase OS=Homo sapiens GN=AHCY PE=1 SV=4	+	Cluster 3	1.999	0.010	Q8NBP7	PCSK9	Proprotein convertase subtilisin/kexin type 9 OS=Homo sapiens GN=PCSK9 PE=1 SV=3	+	Cluster 3	3.537	< 0.001
P10646	TFPI	Tissue factor pathway inhibitor OS=Homo sapiens GN=TFPI PE=1 SV=1	+	Cluster 4	1.461	0.035	O75915	ARL6IP5	PRA1 family protein 3 OS=Homo sapiens GN=ARL6IP5 PE=1 SV=1	+	Cluster 3	1.661	0.022	P29401-2	TKT	Isoform 2 of Transketolase OS=Homo sapiens GN=TKT	+	Cluster 3	2.851	0.001
D3DQH8	SPARC	SPARC OS=Homo sapiens GN=SPARC PE=4 SV=1	+	Cluster 4	1.572	0.027	K7ELJ7	CAPNS1	Calpain small subunit 1 OS=Homo sapiens GN=CAPNS1 PE=1 SV=1	+	Cluster 3	1.706	0.020	P02144	MB	Myoglobin OS=Homo sapiens GN=MB PE=1 SV=2	+	Cluster 3	4.116	< 0.001
Q8N6C8-3	LILRA3	Isoform 3 of Leukocyte immunoglobulin-like receptor subfamily A member 3 OS=Homo sapiens GN=LILRA3	+	Cluster 4	2.457	0.003	P01009	SERPINA1	Alpha-1-antitrypsin OS=Homo sapiens GN=SERPINA1 PE=1 SV=3	+	Cluster 3	2.464	0.003	P11217	PYGM	Glycogen phosphorylase, muscle form OS=Homo sapiens GN=PYGM PE=1 SV=6	+	Cluster 3	4.085	< 0.001
P00390	GSR	Glutathione reductase, mitochondrial OS=Homo sapiens GN=GSR PE=1 SV=2	+	Cluster 4	2.673	0.002	P01137	TGFB1	Transforming growth factor beta-1 OS=Homo sapiens GN=TGFB1 PE=1 SV=2	+	Cluster 3	2.392	0.004	P01876	IGHA1	Ig alpha-1 chain C region OS=Homo sapiens GN=IGHA1 PE=1 SV=2	+	Cluster 3	1.314	0.048
Q15691	MAPRE1	Microtubule-associated protein RP/EB family member 1 OS=Homo sapiens GN=MAPRE1 PE=1 SV=3	+	Cluster 4	1.922	0.012	P00739-2	HPR	Isoform 2 of Haptoglobin-related protein OS=Homo sapiens GN=HPR	+	Cluster 3	2.001	0.010	P54802	NAGLU	Alpha-N-acetylglucosaminidase OS=Homo sapiens GN=NAGLU PE=1 SV=2	+	Cluster 3	2.175	0.007
Q9NSB4	KRT82	Keratin, type II cuticular Hb2 OS=Homo sapiens GN=KRT82 PE=1 SV=3	+	Cluster 4	2.224	0.006	Q6GTS8	PM20D1	Probable carboxypeptidase PM20D1 OS=Homo sapiens GN=PM20D1 PE=2 SV=3	+	Cluster 3	2.652	0.002	O15394	NCAM2	Neural cell adhesion molecule 2 OS=Homo sapiens GN=NCAM2 PE=1 SV=2	+	Cluster 3	2.889	0.001
P54577	YARS	Tyrosine--tRNA ligase, cytoplasmic OS=Homo sapiens GN=YARS PE=1 SV=4	+	Cluster 4	1.503	0.031	P69891	HBG1	Hemoglobin subunit gamma-1 OS=Homo sapiens GN=HBG1 PE=1 SV=2	+	Cluster 3	4.332	< 0.001	Q15063	POSTN	Periostin OS=Homo sapiens GN=POSTN PE=1 SV=2	+	Cluster 3	1.626	0.024
P13489	RNH1	Ribonuclease inhibitor OS=Homo sapiens GN=RNH1 PE=1 SV=2	+	Cluster 4	1.827	0.015	F6TLX2	GLOD4	Glyoxalase domain-containing protein 4 OS=Homo sapiens GN=GLOD4 PE=1 SV=1	+	Cluster 3	3.087	< 0.001	Q76LX8	ADAMTS13	A disintegrin and metalloproteinase with thrombospondin motifs 13 OS=Homo sapiens GN=ADAMTS13 PE=1 SV=1	+	Cluster 3	1.378	0.042
P07741	APRT	Adenine phosphoribosyltransferase OS=Homo sapiens GN=APRT PE=1 SV=2	+	Cluster 4	1.704	0.020	A0A087WX49	IGLL5	Immunoglobulin lambda-like polypeptide 5 OS=Homo sapiens GN=IGLL5 PE=4 SV=1	+	Cluster 3	3.873	< 0.001	P00742	F10	Coagulation factor X OS=Homo sapiens GN=F10 PE=1 SV=2	+	Cluster 3	1.663	0.022
O00194	RAB27B	Ras-related protein Rab-27B OS=Homo sapiens GN=RAB27B PE=1 SV=4	+	Cluster 4	1.618	0.024	A0A075B6L0	IGLC3	Ig lambda-3 chain C regions (Fragment) OS=Homo sapiens GN=IGLC3 PE=4 SV=2	+	Cluster 3	4.445	< 0.001	P06331		Ig heavy chain V-II region ARH-77 OS=Homo sapiens PE=4 SV=1	+	Cluster 3	1.492	0.032
Q07954	LRP1	Prolow-density lipoprotein receptor-related protein 1 OS=Homo sapiens GN=LRP1 PE=1 SV=2	+	Cluster 4	1.490	0.032	A0A087WYJ9	IGHM	Ig mu chain C region OS=Homo sapiens GN=IGHM PE=1 SV=1	+	Cluster 3	2.369	0.004	A0A075B6I0	IGLV8-61	Protein IGLV8-61 (Fragment) OS=Homo sapiens GN=IGLV8-61 PE=4 SV=2	+	Cluster 3	2.295	0.005
Q9UNW1	MINPP1	Multiple inositol polyphosphate phosphatase 1 OS=Homo sapiens GN=MINPP1 PE=1 SV=1	+	Cluster 4	1.388	0.041	A0A075B6I9	IGLV7-46	Protein IGLV7-46 (Fragment) OS=Homo sapiens GN=IGLV7-46 PE=4 SV=2	+	Cluster 3	4.415	< 0.001	Q9Y2I7	PIKFYVE	1-phosphatidylinositol 3-phosphate 5-kinase OS=Homo sapiens GN=PIKFYVE PE=1 SV=3	+	Cluster 3	3.732	< 0.001
P35590	TIE1	Tyrosine-protein kinase receptor Tie-1 OS=Homo sapiens GN=TIE1 PE=1 SV=1	+	Cluster 4	2.497	0.003	P00738	HP	Haptoglobin OS=Homo sapiens GN=HP PE=1 SV=1	+	Cluster 3	3.175	< 0.001	P01765		Ig heavy chain V-III region TIL OS=Homo sapiens PE=1 SV=1	+	Cluster 3	2.844	0.001
Q96RD9	FCRL5	Fc receptor-like protein 5 OS=Homo sapiens GN=FCRL5 PE=1 SV=3	+	Cluster 4	1.820	0.015	P22891-2	PROZ	Isoform 2 of Vitamin K-dependent protein Z OS=Homo sapiens GN=PROZ	+	Cluster 3	2.629	0.002	P04264	KRT1	Keratin, type II cytoskeletal 1 OS=Homo sapiens GN=KRT1 PE=1 SV=6	+	Cluster 3	4.566	< 0.001
P37235	HPCAL1	Hippocalcin-like protein 1 OS=Homo sapiens GN=HPCAL1 PE=1 SV=3	+	Cluster 4	1.696	0.020	P43652	AFM	Afamin OS=Homo sapiens GN=AFM PE=1 SV=1	+	Cluster 3	1.318	0.048	P06732	CKM	Creatine kinase M-type OS=Homo sapiens GN=CKM PE=1 SV=2	+	Cluster 3	4.218	< 0.001
P54727	RAD23B	UV excision repair protein RAD23 homolog B OS=Homo sapiens GN=RAD23B PE=1 SV=1	+	Cluster 4	1.720	0.019	P01011	SERPINA3	Alpha-1-antichymotrypsin OS=Homo sapiens GN=SERPINA3 PE=1 SV=2	+	Cluster 3	1.790	0.016	P35527	KRT9	Keratin, type I cytoskeletal 9 OS=Homo sapiens GN=KRT9 PE=1 SV=3	+	Cluster 3	3.788	< 0.001
P07738	BPGM	Bisphosphoglycerate mutase OS=Homo sapiens GN=BPGM PE=1 SV=2	+	Cluster 4	1.465	0.034	P10599	TXN	Thioredoxin OS=Homo sapiens GN=TXN PE=1 SV=3	+	Cluster 3	1.701	0.020	P13645	KRT10	Keratin, type I cytoskeletal 10 OS=Homo sapiens GN=KRT10 PE=1 SV=6	+	Cluster 3	2.893	0.001
P12955	PEPD	Xaa-Pro dipeptidase OS=Homo sapiens GN=PEPD PE=1 SV=3	+	Cluster 4	1.349	0.045	P01703		Ig lambda chain V-I region NEWM OS=Homo sapiens PE=1 SV=1	+	Cluster 3	1.492	0.032	P35858-2	IGFALS	Isoform 2 of Insulin-like growth factor-binding protein complex acid labile subunit OS=Homo sapiens GN=IGFALS	+	Cluster 3	2.762	0.002
P10644	PRKAR1A	cAMP-dependent protein kinase type I-alpha regulatory subunit OS=Homo sapiens GN=PRKAR1A PE=1 SV=1	+	Cluster 4	2.183	0.007	O75023-3	LILRB5	Isoform 3 of Leukocyte immunoglobulin-like receptor subfamily B member 5 OS=Homo sapiens GN=LILRB5	+	Cluster 3	2.549	0.003	P07225	PROS1	Vitamin K-dependent protein S OS=Homo sapiens GN=PROS1 PE=1 SV=1	+	Cluster 3	1.852	0.014
P51148-2	RAB5C	Isoform 2 of Ras-related protein Rab-5C OS=Homo sapiens GN=RAB5C	+	Cluster 4	1.547	0.028	P05231	IL6	Interleukin-6 OS=Homo sapiens GN=IL6 PE=1 SV=1	+	Cluster 3	1.596	0.025	Q96KN7	RPGRIP1	X-linked retinitis pigmentosa GTPase regulator-interacting protein 1 OS=Homo sapiens GN=RPGRIP1 PE=1 SV=2	+	Cluster 3	1.318	0.048
P32754	HPD	4-hydroxyphenylpyruvate dioxygenase OS=Homo sapiens GN=HPD PE=1 SV=2	+	Cluster 4	1.342	0.045	Q02985	CFHR3	Complement factor H-related protein 3 OS=Homo sapiens GN=CFHR3 PE=1 SV=2	+	Cluster 3	2.833	0.001	Q13596-3	SNX1	Isoform 3 of Sorting nexin-1 OS=Homo sapiens GN=SNX1	+	Cluster 4	1.481	0.033

A0A075B6J8	IGLV3-19	Protein IGLV3-19 (Fragment) OS=Homo sapiens GN=IGLV3-19 PE=4 SV=1	+	Cluster 4	1.392	0.041	Q13630	TSTA3	GDP-L-fucose synthase OS=Homo sapiens GN=TSTA3 PE=1 SV=1	+	Cluster 3	1.471	0.034	P26992	CNTFR	Ciliary neurotrophic factor receptor subunit alpha OS=Homo sapiens GN=CNTFR PE=1 SV=2	+	Cluster 4	1.540	0.029
P22891-2	PROZ	Isoform 2 of Vitamin K-dependent protein Z OS=Homo sapiens GN=PROZ	+	Cluster 4	1.314	0.049	P26447	S100A4	Protein S100-A4 OS=Homo sapiens GN=S100A4 PE=1 SV=1	+	Cluster 3	1.603	0.025	P08107	HSPA1A	Heat shock 70 kDa protein 1A/1B OS=Homo sapiens GN=HSPA1A PE=1 SV=5	+	Cluster 4	1.615	0.024
Q03167	TGFBR3	Transforming growth factor beta receptor type 3 OS=Homo sapiens GN=TGFBR3 PE=1 SV=3	+	Cluster 4	1.442	0.036	A8MU27	SUMO3	Small ubiquitin-related modifier 3 OS=Homo sapiens GN=SUMO3 PE=1 SV=1	+	Cluster 3	2.194	0.006	P12109	COL6A1	Collagen alpha-1(VI) chain OS=Homo sapiens GN=COL6A1 PE=1 SV=3	+	Cluster 4	1.601	0.025
P06744-2	GPI	Isoform 2 of Glucose-6-phosphate isomerase OS=Homo sapiens GN=GPI	+	Cluster 4	1.431	0.037	Q6IBS0	TWF2	Twinfilin-2 OS=Homo sapiens GN=TWF2 PE=1 SV=2	+	Cluster 3	1.517	0.030	E7ETH0	CFI	Complement factor I light chain OS=Homo sapiens GN=CFI PE=3 SV=1	+	Cluster 4	1.597	0.025
P06737	PYGL	Glycogen phosphorylase, liver form OS=Homo sapiens GN=PYGL PE=1 SV=4	+	Cluster 4	1.520	0.030	P05121	SERPINE1	Plasminogen activator inhibitor 1 OS=Homo sapiens GN=SERPINE1 PE=1 SV=1	+	Cluster 4	1.453	0.035	Q5T9B7	AK1	Adenylate kinase isoenzyme 1 OS=Homo sapiens GN=AK1 PE=1 SV=1	+	Cluster 4	1.454	0.035
P14324	FDPS	Farnesyl pyrophosphate synthase OS=Homo sapiens GN=FDPS PE=1 SV=4	+	Cluster 4	1.766	0.017	Q9P232	CNTN3	Contactin-3 OS=Homo sapiens GN=CNTN3 PE=1 SV=3	+	Cluster 4	1.500	0.032	P14324	FDPS	Farnesyl pyrophosphate synthase OS=Homo sapiens GN=FDPS PE=1 SV=4	+	Cluster 4	1.751	0.018
P11766	ADH5	Alcohol dehydrogenase class-3 OS=Homo sapiens GN=ADH5 PE=1 SV=4	+	Cluster 4	1.303	0.050	Q9NUQ9	FAM49B	Protein FAM49B OS=Homo sapiens GN=FAM49B PE=1 SV=1	+	Cluster 4	1.320	0.048	P05062	ALDOB	Fructose-bisphosphate aldolase B OS=Homo sapiens GN=ALDOB PE=1 SV=2	+	Cluster 4	1.864	0.014
P27918	CFP	Properdin OS=Homo sapiens GN=CFP PE=1 SV=2	+	Cluster 4	1.430	0.037	P00558	PGK1	Phosphoglycerate kinase 1 OS=Homo sapiens GN=PGK1 PE=1 SV=3	+	Cluster 4	1.343	0.045	P23141-2	CES1	Isoform 2 of Liver carboxylesterase 1 OS=Homo sapiens GN=CES1	+	Cluster 4	1.614	0.024
P00918	CA2	Carbonic anhydrase 2 OS=Homo sapiens GN=CA2 PE=1 SV=2	+	Cluster 4	1.397	0.040	O60888-2	CUTA	Isoform A of Protein CutA OS=Homo sapiens GN=CUTA	+	Cluster 4	1.557	0.028	P35590	TIE1	Tyrosine-protein kinase receptor Tie-1 OS=Homo sapiens GN=TIE1 PE=1 SV=1	+	Cluster 4	1.414	0.039
A0A087WYC5	IGHG1	Ig gamma-1 chain C region OS=Homo sapiens GN=IGHG1 PE=1 SV=1	+	Cluster 4	1.331	0.047	P63104	YWHAZ	14-3-3 protein zeta/delta OS=Homo sapiens GN=YWHAZ PE=1 SV=1	+	Cluster 4	1.961	0.011	P01703		Ig lambda chain V-I region NEWM OS=Homo sapiens PE=1 SV=1	+	Cluster 4	1.560	0.028
P37837	TALDO1	Transaldolase OS=Homo sapiens GN=TALDO1 PE=1 SV=2	+	Cluster 4	1.521	0.030	P12931-2	SRC	Isoform 2 of Proto-oncogene tyrosine-protein kinase Src OS=Homo sapiens GN=SRC	+	Cluster 4	2.096	0.008	Q96G03	PGM2	Phosphoglucomutase-2 OS=Homo sapiens GN=PGM2 PE=1 SV=4	+	Cluster 4	1.613	0.024
P24534	EEF1B2	Elongation factor 1-beta OS=Homo sapiens GN=EEF1B2 PE=1 SV=3	+	Cluster 4	1.383	0.041	P04406	GAPDH	Glyceraldehyde-3-phosphate dehydrogenase OS=Homo sapiens GN=GAPDH PE=1 SV=3	+	Cluster 4	1.408	0.039	Q12805	EFEMP1	EGF-containing fibulin-like extracellular matrix protein 1 OS=Homo sapiens GN=EFEMP1 PE=1 SV=2	+	Cluster 4	2.393	0.004
P08107	HSPA1A	Heat shock 70 kDa protein 1A/1B OS=Homo sapiens GN=HSPA1A PE=1 SV=5	+	Cluster 4	1.411	0.039	O75083	WDR1	WD repeat-containing protein 1 OS=Homo sapiens GN=WDR1 PE=1 SV=4	+	Cluster 4	1.703	0.020	P36980	CFHR2	Complement factor H-related protein 2 OS=Homo sapiens GN=CFHR2 PE=1 SV=1	+	Cluster 4	4.119	< 0.001
P68363	TUBA1B	Tubulin alpha-1B chain OS=Homo sapiens GN=TUBA1B PE=1 SV=1	+	Cluster 4	1.731	0.019	P54727	RAD23B	UV excision repair protein RAD23 homolog B OS=Homo sapiens GN=RAD23B PE=1 SV=1	+	Cluster 4	1.646	0.023	Q9UJJ9	GNPTG	N-acetylglucosamine-1-phosphotransferase subunit gamma OS=Homo sapiens GN=GNPTG PE=1 SV=1	+	Cluster 4	1.536	0.029
O15144	ARPC2	Actin-related protein 2/3 complex subunit 2 OS=Homo sapiens GN=ARPC2 PE=1 SV=1	+	Cluster 4	1.655	0.022	P52907	CAPZA1	F-actin-capping protein subunit alpha-1 OS=Homo sapiens GN=CAPZA1 PE=1 SV=3	+	Cluster 4	1.493	0.032	P14543	NID1	Nidogen-1 OS=Homo sapiens GN=NID1 PE=1 SV=3	+	Cluster 4	1.741	0.018
Q9P232	CNTN3	Contactin-3 OS=Homo sapiens GN=CNTN3 PE=1 SV=3	+	Cluster 4	1.340	0.046	Q8N6C8-3	LILRA3	Isoform 3 of Leukocyte immunoglobulin-like receptor subfamily A member 3 OS=Homo sapiens GN=LILRA3	+	Cluster 4	2.345	0.005	Q9HCU0	CD248	Endosialin OS=Homo sapiens GN=CD248 PE=1 SV=1	+	Cluster 4	2.461	0.003
P02654	APOC1	Apolipoprotein C-I OS=Homo sapiens GN=APOC1 PE=1 SV=1	+	Cluster 4	1.459	0.035	Q12841	FSTL1	Follistatin-related protein 1 OS=Homo sapiens GN=FSTL1 PE=1 SV=1	+	Cluster 4	1.730	0.019	P36955	SERPINF1	Pigment epithelium-derived factor OS=Homo sapiens GN=SERPINF1 PE=1 SV=4	+	Cluster 4	1.428	0.037
O75636	FCN3	Ficolin-3 OS=Homo sapiens GN=FCN3 PE=1 SV=2	+	Cluster 4	1.775	0.017	P35590	TIE1	Tyrosine-protein kinase receptor Tie-1 OS=Homo sapiens GN=TIE1 PE=1 SV=1	+	Cluster 4	1.376	0.042	P19652	ORM2	Alpha-1-acid glycoprotein 2 OS=Homo sapiens GN=ORM2 PE=1 SV=2	+	Cluster 4	2.520	0.003
P07225	PROS1	Vitamin K-dependent protein S OS=Homo sapiens GN=PROS1 PE=1 SV=1	+	Cluster 4	1.497	0.032	P86U17	SERPINA11	Serpin A11 OS=Homo sapiens GN=SERPINA11 PE=2 SV=2	+	Cluster 4	1.517	0.030	P04083	ANXA1	Annexin A1 OS=Homo sapiens GN=ANXA1 PE=1 SV=2	+	Cluster 4	2.085	0.008
O60888-2	CUTA	Isoform A of Protein CutA OS=Homo sapiens GN=CUTA	+	Cluster 4	1.559	0.028	H0YMD1	LDLR	Low-density lipoprotein receptor OS=Homo sapiens GN=LDLR PE=1 SV=1	+	Cluster 4	1.585	0.026	P22692	IGFBP4	Insulin-like growth factor-binding protein 4 OS=Homo sapiens GN=IGFBP4 PE=1 SV=2	+	Cluster 4	2.625	0.002
P04406	GAPDH	Glyceraldehyde-3-phosphate dehydrogenase OS=Homo sapiens GN=GAPDH PE=1 SV=3	+	Cluster 4	1.552	0.028	Q14623	IHH	Indian hedgehog protein OS=Homo sapiens GN=IHH PE=1 SV=4	+	Cluster 4	2.448	0.004	O75563	SKAP2	Src kinase-associated phosphoprotein 2 OS=Homo sapiens GN=SKAP2 PE=1 SV=1	+	Cluster 4	1.917	0.012
P52907	CAPZA1	F-actin-capping protein subunit alpha-1 OS=Homo sapiens GN=CAPZA1 PE=1 SV=3	+	Cluster 4	1.484	0.033	P18669	PGAM1	Phosphoglycerate mutase 1 OS=Homo sapiens GN=PGAM1 PE=1 SV=2	+	Cluster 4	2.724	0.002	P32754	HPD	4-hydroxyphenylpyruvate dioxygenase OS=Homo sapiens GN=HPD PE=1 SV=2	+	Cluster 4	3.025	< 0.001
P58546	MTPN	Myotrophin OS=Homo sapiens GN=MTPN PE=1 SV=2	+	Cluster 4	2.736	0.002	O95897	OLFM2	Noelin-2 OS=Homo sapiens GN=OLFM2 PE=2 SV=2	+	Cluster 4	1.594	0.025	P20774	OGN	Mimecan OS=Homo sapiens GN=OGN PE=1 SV=1	+	Cluster 4	1.678	0.021
P40197	GP5	Platelet glycoprotein V OS=Homo sapiens GN=GP5 PE=1 SV=1	+	Cluster 4	2.364	0.004	Q15691	MAPRE1	Microtubule-associated protein RP/EB family member 1 OS=Homo sapiens GN=MAPRE1 PE=1 SV=3	+	Cluster 4	2.003	0.010	K7ELL7	PRKCSH	Glucosidase 2 subunit beta OS=Homo sapiens GN=PRKCSH PE=1 SV=1	+	Cluster 4	1.686	0.021
P32119	PRDX2	Peroxiredoxin-2 OS=Homo sapiens GN=PRDX2 PE=1 SV=5	+	Cluster 4	2.495	0.003	Q9Y6Z7	COLEC10	Collectin-10 OS=Homo sapiens GN=COLEC10 PE=2 SV=2	+	Cluster 4	1.836	0.015	P07437	TUBB	Tubulin beta chain OS=Homo sapiens GN=TUBB PE=1 SV=2	+	Cluster 4	1.845	0.014
P62979	RPS27A	Ubiquitin-40S ribosomal protein S27a OS=Homo sapiens GN=RPS27A PE=1 SV=2	+	Cluster 4	2.828	0.001	P29401-2	TKT	Isoform 2 of Transketolase OS=Homo sapiens GN=TKT	+	Cluster 4	2.244	0.006	Q9NY15	STAB1	Stabilin-1 OS=Homo sapiens GN=STAB1 PE=1 SV=3	+	Cluster 4	1.537	0.029
P00451	F8	Coagulation factor VIII OS=Homo sapiens GN=F8 PE=1 SV=1	+	Cluster 4	2.786	0.002	P00451	F8	Coagulation factor VIII OS=Homo sapiens GN=F8 PE=1 SV=1	+	Cluster 4	1.768	0.017	P00739-2	HPR	Isoform 2 of Haptoglobin-related protein OS=Homo sapiens GN=HPR	+	Cluster 4	2.782	0.002
P01033	TIMP1	Metalloproteinase inhibitor 1 OS=Homo sapiens GN=TIMP1 PE=1 SV=1	+	Cluster 4	1.899	0.013	P00390	GSR	Glutathione reductase, mitochondrial OS=Homo sapiens GN=GSR PE=1 SV=2	+	Cluster 4	2.073	0.008	K7ELJ7	CAPNS1	Calpain small subunit 1 OS=Homo sapiens GN=CAPNS1 PE=1 SV=1	+	Cluster 4	1.969	0.011
P11021	HSPA5	78 kDa glucose-regulated protein OS=Homo sapiens GN=HSPA5 PE=1 SV=2	+	Cluster 4	2.154	0.007	P01880-2	IGHD	Isoform 2 of Ig delta chain C region OS=Homo sapiens GN=IGHD	+	Cluster 4	2.734	0.002	P04040	CAT	Catalase OS=Homo sapiens GN=CAT PE=1 SV=3	+	Cluster 4	2.767	0.002
P50552	VASP	Vasodilator-stimulated phosphoprotein OS=Homo sapiens GN=VASP PE=1 SV=3	+	Cluster 4	1.627	0.024	P12109	COL6A1	Collagen alpha-1(VI) chain OS=Homo sapiens GN=COL6A1 PE=1 SV=3	+	Cluster 4	2.189	0.006	P16520	GNB3	Guanine nucleotide-binding protein G(I)/G(S)/G(T) subunit beta-3 OS=Homo sapiens GN=GNB3 PE=1 SV=1	+	Cluster 4	2.242	0.006
Q9HCU0	CD248	Endosialin OS=Homo sapiens GN=CD248 PE=1 SV=1	+	Cluster 4	1.888	0.013	P27918	CFP	Properdin OS=Homo sapiens GN=CFP PE=1 SV=2	+	Cluster 4	1.821	0.015	P09960	LTA4H	Leukotriene A-4 hydrolase OS=Homo sapiens GN=LTA4H PE=1 SV=2	+	Cluster 4	1.943	0.011
Q15555	MAPRE2	Microtubule-associated protein RP/EB family member 2 OS=Homo sapiens GN=MAPRE2 PE=1 SV=1	+	Cluster 4	2.075	0.008	P00918	CA2	Carbonic anhydrase 2 OS=Homo sapiens GN=CA2 PE=1 SV=2	+	Cluster 4	2.280	0.005	P07741	APRT	Adenine phosphoribosyltransferase OS=Homo sapiens GN=APRT PE=1 SV=2	+	Cluster 4	1.762	0.017
Q9NZN3	EHD3	EH domain-containing protein 3 OS=Homo sapiens GN=EHD3 PE=1 SV=2	+	Cluster 4	2.434	0.004	P04040	CAT	Catalase OS=Homo sapiens GN=CAT PE=1 SV=3	+	Cluster 4	2.743	0.002	P22891-2	PROZ	Isoform 2 of Vitamin K-dependent protein Z OS=Homo sapiens GN=PROZ	+	Cluster 4	2.880	0.001
P12931-2	SRC	Isoform 2 of Proto-oncogene tyrosine-protein kinase Src OS=Homo sapiens GN=SRC	+	Cluster 4	1.916	0.012	P02008	HBZ	Hemoglobin subunit zeta OS=Homo sapiens GN=HBZ PE=1 SV=2	+	Cluster 4	2.928	0.001	P00738	HP	Haptoglobin OS=Homo sapiens GN=HP PE=1 SV=1	+	Cluster 4	3.248	< 0.001
P00558	PGK1	Phosphoglycerate kinase 1 OS=Homo sapiens GN=PGK1 PE=1 SV=3	+	Cluster 4	1.356	0.044	P19652	ORM2	Alpha-1-acid glycoprotein 2 OS=Homo sapiens GN=ORM2 PE=1 SV=2	+	Cluster 4	2.819	0.002	P31939	ATIC	Bifunctional purine biosynthesis protein PURH OS=Homo sapiens GN=ATIC PE=1 SV=3	+	Cluster 4	3.635	< 0.001
O75083	WDR1	WD repeat-containing protein 1 OS=Homo sapiens GN=WDR1 PE=1 SV=4	+	Cluster 4	1.591	0.026	P01591	IGJ	Immunoglobulin J chain OS=Homo sapiens GN=IGJ PE=1 SV=4	+	Cluster 4	3.188	< 0.001	P09493-8	TPM1	Isoform 8 of Tropomyosin alpha-1 chain OS=Homo sapiens GN=TPM1	+	Cluster 4	2.440	0.004
P63104	YWHAZ	14-3-3 protein zeta/delta OS=Homo sapiens GN=YWHAZ PE=1 SV=1	+	Cluster 4	1.892	0.013	P16930	FAH	Fumarylacetoacetase OS=Homo sapiens GN=FAH PE=1 SV=2	+	Cluster 4	2.415	0.004	P00325	ADH1B	Alcohol dehydrogenase 1B OS=Homo sapiens GN=ADH1B PE=1 SV=2	+	Cluster 4	2.324	0.005
Q14247	CTTN	Src substrate cortactin OS=Homo sapiens GN=CTTN PE=1 SV=2	+	Cluster 4	2.387	0.004	P06727	APOA4	Apolipoprotein A-IV OS=Homo sapiens GN=APOA4 PE=1 SV=3	+	Cluster 4	1.336	0.046	P0DJI9	SAA2	Serum amyloid A-2 protein OS=Homo sapiens GN=SAA2 PE=1 SV=1	+	Cluster 4	3.303	< 0.001
Q9BS40	LXN	Latexin OS=Homo sapiens GN=LXN PE=1 SV=2	+	Cluster 4	1.719	0.019	P31939	ATIC	Bifunctional purine biosynthesis protein PURH OS=Homo sapiens GN=ATIC PE=1 SV=3	+	Cluster 4	1.549	0.028	Q6IBS0	TWF2	Twinfilin-2 OS=Homo sapiens GN=TWF2 PE=1 SV=2	+	Cluster 4	1.750	0.018
A0A075B6I0	IGLV8-61	Protein IGLV8-61 (Fragment) OS=Homo sapiens GN=IGLV8-61 PE=4 SV=2	+	Cluster 4	1.967	0.011	P22692	IGFBP4	Insulin-like growth factor-binding protein 4 OS=Homo sapiens GN=IGFBP4 PE=1 SV=2	+	Cluster 4	1.667	0.022	Q05682	CALD1	Caldesmon OS=Homo sapiens GN=CALD1 PE=1 SV=3	+	Cluster 4	2.929	0.001

A0A087WXC9	MADCAM1	Mucosal addressin cell adhesion molecule 1 OS=Homo sapiens GN=MADCAM1 PE=4 SV=1	+	Cluster 4	1.770	0.017	P13716-2	ALAD	Isoform 2 of Delta-aminolevulinic acid dehydratase OS=Homo sapiens GN=ALAD	+	Cluster 4	2.453	0.004	A0A075B6I9	IGLV7-46	Protein IGLV7-46 (Fragment) OS=Homo sapiens GN=IGLV7-46 PE=4 SV=2	+	Cluster 4	2.264	0.005
Q9Y2I7	PIKFYVE	1-phosphatidylinositol 3-phosphate 5-kinase OS=Homo sapiens GN=PIKFYVE PE=1 SV=3	+	Cluster 4	3.384	< 0.001	P00325	ADH1B	Alcohol dehydrogenase 1B OS=Homo sapiens GN=ADH1B PE=1 SV=2	+	Cluster 4	1.688	0.021	O15143	ARPC1B	Actin-related protein 2/3 complex subunit 1B OS=Homo sapiens GN=ARPC1B PE=1 SV=3	+	Cluster 4	2.785	0.002
Q9NUQ9	FAM49B	Protein FAM49B OS=Homo sapiens GN=FAM49B PE=1 SV=1	+	Cluster 4	2.901	0.001	P10451	SPP1	Osteopontin OS=Homo sapiens GN=SPP1 PE=1 SV=1	+	Cluster 4	1.509	0.031	J3KQE5	RAN	GTP-binding nuclear protein Ran (Fragment) OS=Homo sapiens GN=RAN PE=1 SV=1	+	Cluster 4	1.818	0.015
P06727	APOA4	Apolipoprotein A-IV OS=Homo sapiens GN=APOA4 PE=1 SV=3	+	Cluster 4	2.038	0.009	P07741	APRT	Adenine phosphoribosyltransferase OS=Homo sapiens GN=APRT PE=1 SV=2	+	Cluster 4	1.485	0.033	P01024	C3	Complement C3 OS=Homo sapiens GN=C3 PE=1 SV=2	+	Cluster 4	2.002	0.010
P11908-2	PRPS2	Isoform 2 of Ribose-phosphate pyrophosphokinase 2 OS=Homo sapiens GN=PRPS2	+	Cluster 4	1.874	0.013	P24534	EEF1B2	Elongation factor 1-beta OS=Homo sapiens GN=EEF1B2 PE=1 SV=3	+	Cluster 4	1.350	0.045	P61204	ARF3	ADP-ribosylation factor 3 OS=Homo sapiens GN=ARF3 PE=1 SV=2	+	Cluster 4	3.026	< 0.001
P25786-2	PSMA1	Isoform Long of Proteasome subunit alpha type-1 OS=Homo sapiens GN=PSMA1	+	Cluster 4	1.410	0.039	P10644	PRKAR1A	cAMP-dependent protein kinase type I-alpha regulatory subunit OS=Homo sapiens GN=PRKAR1A PE=1 SV=1	+	Cluster 4	1.797	0.016	P06703	S100A6	Protein S100-A6 OS=Homo sapiens GN=S100A6 PE=1 SV=1	+	Cluster 4	2.119	0.008
O95810	SDPR	Serum deprivation-response protein OS=Homo sapiens GN=SDPR PE=1 SV=3	+	Cluster 4	1.396	0.040	P05109	S100A8	Protein S100-A8 OS=Homo sapiens GN=S100A8 PE=1 SV=1	+	Cluster 4	1.987	0.010	P10644	PRKAR1A	cAMP-dependent protein kinase type I-alpha regulatory subunit OS=Homo sapiens GN=PRKAR1A PE=1 SV=1	+	Cluster 4	2.010	0.010
P02753	RBP4	Retinol-binding protein 4 OS=Homo sapiens GN=RBP4 PE=1 SV=3	+	Cluster 4	2.434	0.004	Q15848	ADIPOQ	Adiponectin OS=Homo sapiens GN=ADIPOQ PE=1 SV=1	+	Cluster 4	1.561	0.027	O43866	CD5L	CD5 antigen-like OS=Homo sapiens GN=CD5L PE=1 SV=1	+	Cluster 4	1.560	0.028
Q9Y6Z7	COLEC10	Collectin-10 OS=Homo sapiens GN=COLEC10 PE=2 SV=2	+	Cluster 4	2.692	0.002	P37837	TALDO1	Transaldolase OS=Homo sapiens GN=TALDO1 PE=1 SV=2	+	Cluster 4	1.854	0.014	Q02985	CFHR3	Complement factor H-related protein 3 OS=Homo sapiens GN=CFHR3 PE=1 SV=2	+	Cluster 4	2.548	0.003
H0YMD1	LDLR	Low-density lipoprotein receptor OS=Homo sapiens GN=LDLR PE=1 SV=1	+	Cluster 4	1.823	0.015	P06737	PYGL	Glycogen phosphorylase, liver form OS=Homo sapiens GN=PYGL PE=1 SV=4	+	Cluster 4	2.235	0.006	P01591	IGJ	Immunoglobulin J chain OS=Homo sapiens GN=IGJ PE=1 SV=4	+	Cluster 4	3.082	< 0.001
Q8N392	ARHGAP18	Rho GTPase-activating protein 18 OS=Homo sapiens GN=ARHGAP18 PE=1 SV=3	+	Cluster 4	1.693	0.020	P50453	SERPINB9	Serpin B9 OS=Homo sapiens GN=SERPINB9 PE=1 SV=1	+	Cluster 4	1.744	0.018	P50552	VASP	Vasodilator-stimulated phosphoprotein OS=Homo sapiens GN=VASP PE=1 SV=3	+	Cluster 4	2.294	0.005
B4DUT8	CNN2	Calponin OS=Homo sapiens GN=CNN2 PE=1 SV=1	+	Cluster 4	1.500	0.032	A0A075B6J3	IGLV3-27	Protein IGLV3-27 (Fragment) OS=Homo sapiens GN=IGLV3-27 PE=4 SV=1	+	Cluster 4	1.354	0.044	P02760	AMBP	Protein AMBP OS=Homo sapiens GN=AMBP PE=1 SV=1	+	Cluster 4	4.309	< 0.001
P12814-3	ACTN1	Isoform 3 of Alpha-actinin-1 OS=Homo sapiens GN=ACTN1	+	Cluster 4	1.992	0.010	Q9UJJ9	GNPTG	N-acetylglucosamine-1-phosphotransferase subunit gamma OS=Homo sapiens GN=GNPTG PE=1 SV=1	+	Cluster 4	1.860	0.014	P0C0L5	C4B	Complement C4-B OS=Homo sapiens GN=C4B PE=1 SV=2	+	Cluster 4	3.250	< 0.001
P50990	CCT8	T-complex protein 1 subunit theta OS=Homo sapiens GN=CCT8 PE=1 SV=4	+	Cluster 4	2.063	0.009	A0A075B6J8	IGLV3-19	Protein IGLV3-19 (Fragment) OS=Homo sapiens GN=IGLV3-19 PE=4 SV=1	+	Cluster 4	1.971	0.011	P02775	PPBP	Platelet basic protein OS=Homo sapiens GN=PPBP PE=1 SV=3	+	Cluster 4	4.680	< 0.001
O60234	GMFG	Glia maturation factor gamma OS=Homo sapiens GN=GMFG PE=1 SV=1	+	Cluster 4	2.824	0.002	Q13201	MMRN1	Multimerin-1 OS=Homo sapiens GN=MMRN1 PE=1 SV=3	+	Cluster 4	1.621	0.024	P07738	BPGM	Bisphosphoglycerate mutase OS=Homo sapiens GN=BPGM PE=1 SV=2	+	Cluster 4	3.934	< 0.001
P14618	PKM	Pyruvate kinase PKM OS=Homo sapiens GN=PKM PE=1 SV=4	+	Cluster 4	1.890	0.013	G3V5Z7	PSMA6	Proteasome subunit alpha type OS=Homo sapiens GN=PSMA6 PE=1 SV=1	+	Cluster 4	1.921	0.012	P16930	FAH	Fumarylacetoacetase OS=Homo sapiens GN=FAH PE=1 SV=2	+	Cluster 4	3.377	< 0.001
Q14847-2	LASP1	Isoform 2 of LIM and SH3 domain protein 1 OS=Homo sapiens GN=LASP1	+	Cluster 4	2.487	0.003	Q7L9L4-2	MOB1B	Isoform 2 of MOB kinase activator 1B OS=Homo sapiens GN=MOB1B	+	Cluster 4	1.490	0.032	C9JU00	FGG	Fibrinogen gamma chain (Fragment) OS=Homo sapiens GN=FGG PE=1 SV=1	+	Cluster 4	2.240	0.006
Q9BR76	CORO1B	Coronin-1B OS=Homo sapiens GN=CORO1B PE=1 SV=1	+	Cluster 4	1.771	0.017	P29350-4	PTPN6	Isoform 4 of Tyrosine-protein phosphatase non-receptor type 6 OS=Homo sapiens GN=PTPN6	+	Cluster 4	2.925	0.001	P54577	YARS	Tyrosine--tRNA ligase, cytoplasmic OS=Homo sapiens GN=YARS PE=1 SV=4	+	Cluster 4	1.452	0.035
P36871	PGM1	Phosphoglucomutase-1 OS=Homo sapiens GN=PGM1 PE=1 SV=3	+	Cluster 4	4.264	< 0.001	P08107	HSPA1A	Heat shock 70 kDa protein 1A/1B OS=Homo sapiens GN=HSPA1A PE=1 SV=5	+	Cluster 4	2.273	0.005	P01009	SERPINA1	Alpha-1-antitrypsin OS=Homo sapiens GN=SERPINA1 PE=1 SV=3	+	Cluster 4	3.496	< 0.001
P29401-2	TKT	Isoform 2 of Transketolase OS=Homo sapiens GN=TKT	+	Cluster 4	3.077	< 0.001	P62993	GRB2	Growth factor receptor-bound protein 2 OS=Homo sapiens GN=GRB2 PE=1 SV=1	+	Cluster 4	1.425	0.038	Q9NRW1	RAB6B	Ras-related protein Rab-6B OS=Homo sapiens GN=RAB6B PE=1 SV=1	+	Cluster 4	3.081	< 0.001
P14780	MMP9	Matrix metalloproteinase-9 OS=Homo sapiens GN=MMP9 PE=1 SV=3	+	Cluster 4	2.399	0.004	Q14515	SPARCL1	SPARC-like protein 1 OS=Homo sapiens GN=SPARCL1 PE=1 SV=2	+	Cluster 4	1.366	0.043	P61106	RAB14	Ras-related protein Rab-14 OS=Homo sapiens GN=RAB14 PE=1 SV=4	+	Cluster 4	1.594	0.025
P60174	TPI1	Triosephosphate isomerase OS=Homo sapiens GN=TPI1 PE=1 SV=3	+	Cluster 4	2.557	0.003	Q14019	COTL1	Coactosin-like protein OS=Homo sapiens GN=COTL1 PE=1 SV=3	+	Cluster 4	2.055	0.009	P02008	HBZ	Hemoglobin subunit zeta OS=Homo sapiens GN=HBZ PE=1 SV=2	+	Cluster 4	2.395	0.004
O00151	PDLIM1	PDZ and LIM domain protein 1 OS=Homo sapiens GN=PDLIM1 PE=1 SV=4	+	Cluster 4	3.438	< 0.001	P35030	PRSS3	Trypsin-3 OS=Homo sapiens GN=PRSS3 PE=1 SV=2	+	Cluster 4	1.845	0.014	P05231	IL6	Interleukin-6 OS=Homo sapiens GN=IL6 PE=1 SV=1	+	Cluster 4	1.440	0.036
P06733	ENO1	Alpha-enolase OS=Homo sapiens GN=ENO1 PE=1 SV=2	+	Cluster 4	3.531	< 0.001	P40189	IL6ST	Interleukin-6 receptor subunit beta OS=Homo sapiens GN=IL6ST PE=1 SV=2	+	Cluster 4	1.648	0.022	Q15848	ADIPOQ	Adiponectin OS=Homo sapiens GN=ADIPOQ PE=1 SV=1	+	Cluster 4	1.381	0.042
Q8WWM7-3	ATXN2L	Isoform 3 of Ataxin-2-like protein OS=Homo sapiens GN=ATXN2L	+	Cluster 4	1.339	0.046	J3KQE5	RAN	GTP-binding nuclear protein Ran (Fragment) OS=Homo sapiens GN=RAN PE=1 SV=1	+	Cluster 4	2.300	0.005	G3V2W1	SERPINA10	Protein Z-dependent protease inhibitor OS=Homo sapiens GN=SERPINA10 PE=3 SV=1	+	Cluster 4	1.303	0.050
Q7L9L4-2	MOB1B	Isoform 2 of MOB kinase activator 1B OS=Homo sapiens GN=MOB1B	+	Cluster 4	1.666	0.022	P00352	ALDH1A1	Retinal dehydrogenase 1 OS=Homo sapiens GN=ALDH1A1 PE=1 SV=2	+	Cluster 4	2.960	0.001	Q86YW5	TREML1	Trem-like transcript 1 protein OS=Homo sapiens GN=TREML1 PE=1 SV=2	+	Cluster 4	2.737	0.002
P29350-4	PTPN6	Isoform 4 of Tyrosine-protein phosphatase non-receptor type 6 OS=Homo sapiens GN=PTPN6	+	Cluster 4	2.475	0.003	P01033	TIMP1	Metalloproteinase inhibitor 1 OS=Homo sapiens GN=TIMP1 PE=1 SV=1	+	Cluster 4	2.547	0.003	P50395	GDI2	Rab GDP dissociation inhibitor beta OS=Homo sapiens GN=GDI2 PE=1 SV=2	+	Cluster 4	2.475	0.003
O75874	IDH1	Isocitrate dehydrogenase [NADP] cytoplasmic OS=Homo sapiens GN=IDH1 PE=1 SV=2	+	Cluster 4	1.559	0.028	Q15084-2	PDIA6	Isoform 2 of Protein disulfide-isomerase A6 OS=Homo sapiens GN=PDIA6	+	Cluster 4	1.585	0.026	P13716-2	ALAD	Isoform 2 of Delta-aminolevulinic acid dehydratase OS=Homo sapiens GN=ALAD	+	Cluster 4	1.369	0.043
P06331		Ig heavy chain V-II region ARH-77 OS=Homo sapiens PE=4 SV=1	+	Cluster 4	1.442	0.036	O75874	IDH1	Isocitrate dehydrogenase [NADP] cytoplasmic OS=Homo sapiens GN=IDH1 PE=1 SV=2	+	Cluster 4	1.745	0.018	G3V5Z7	PSMA6	Proteasome subunit alpha type OS=Homo sapiens GN=PSMA6 PE=1 SV=1	+	Cluster 4	2.144	0.007
P40189	IL6ST	Interleukin-6 receptor subunit beta OS=Homo sapiens GN=IL6ST PE=1 SV=2	+	Cluster 4	1.593	0.026	P21333	FLNA	Filamin-A OS=Homo sapiens GN=FLNA PE=1 SV=4	+	Cluster 4	2.490	0.003	P06737	PYGL	Glycogen phosphorylase, liver form OS=Homo sapiens GN=PYGL PE=1 SV=4	+	Cluster 4	1.951	0.011
Q14019	COTL1	Coactosin-like protein OS=Homo sapiens GN=COTL1 PE=1 SV=3	+	Cluster 4	1.841	0.014	P15085	CPA1	Carboxypeptidase A1 OS=Homo sapiens GN=CPA1 PE=1 SV=2	+	Cluster 4	1.846	0.014	O14745	SLC9A3R1	Na(+)/H(+) exchange regulatory cofactor NHE-RF1 OS=Homo sapiens GN=SLC9A3R1 PE=1 SV=4	+	Cluster 4	2.403	0.004
P04040	CAT	Catalase OS=Homo sapiens GN=CAT PE=1 SV=3	+	Cluster 4	1.452	0.035	O00533-2	CHL1	Isoform 2 of Neural cell adhesion molecule L1-like protein OS=Homo sapiens GN=CHL1	+	Cluster 4	1.451	0.035	P40197	GP5	Platelet glycoprotein V OS=Homo sapiens GN=GP5 PE=1 SV=1	+	Cluster 4	2.072	0.008
E7EMB3	CALM2	Calmodulin OS=Homo sapiens GN=CALM2 PE=1 SV=1	+	Cluster 4	1.869	0.014	P30044	PRDX5	Peroxiredoxin-5, mitochondrial OS=Homo sapiens GN=PRDX5 PE=1 SV=4	+	Cluster 4	2.607	0.002	P08319-2	ADH4	Isoform 2 of Alcohol dehydrogenase 4 OS=Homo sapiens GN=ADH4	+	Cluster 4	1.348	0.045
P00352	ALDH1A1	Retinal dehydrogenase 1 OS=Homo sapiens GN=ALDH1A1 PE=1 SV=2	+	Cluster 4	2.483	0.003	Q01518	CAP1	Adenylyl cyclase-associated protein 1 OS=Homo sapiens GN=CAP1 PE=1 SV=5	+	Cluster 4	1.528	0.030	Q92954	PRG4	Proteoglycan 4 OS=Homo sapiens GN=PRG4 PE=1 SV=2	+	Cluster 4	2.238	0.006
Q96KP4	CNDP2	Cytosolic non-specific dipeptidase OS=Homo sapiens GN=CNDP2 PE=1 SV=2	+	Cluster 4	2.644	0.002	O14818	PSMA7	Proteasome subunit alpha type-7 OS=Homo sapiens GN=PSMA7 PE=1 SV=1	+	Cluster 4	1.619	0.024	Q07960	ARHGAP1	Rho GTPase-activating protein 1 OS=Homo sapiens GN=ARHGAP1 PE=1 SV=1	+	Cluster 4	2.837	0.001
P02042	HBD	Hemoglobin subunit delta OS=Homo sapiens GN=HBD PE=1 SV=2	+	Cluster 4	3.102	< 0.001	P07237	P4HB	Protein disulfide-isomerase OS=Homo sapiens GN=P4HB PE=1 SV=3	+	Cluster 4	1.639	0.023	P07900-2	HSP90AA1	Isoform 2 of Heat shock protein HSP 90-alpha OS=Homo sapiens GN=HSP90AA1	+	Cluster 4	3.360	< 0.001
P00915	CA1	Carbonic anhydrase 1 OS=Homo sapiens GN=CA1 PE=1 SV=2	+	Cluster 4	4.322	< 0.001	P07996	THBS1	Thrombospondin-1 OS=Homo sapiens GN=THBS1 PE=1 SV=2	+	Cluster 4	2.018	0.010	B4DUT8	CNN2	Calponin OS=Homo sapiens GN=CNN2 PE=1 SV=1	+	Cluster 4	3.480	< 0.001
P68032	ACTC1	Actin, alpha cardiac muscle 1 OS=Homo sapiens GN=ACTC1 PE=1 SV=1	+	Cluster 4	3.628	< 0.001	P61160-2	ACTR2	Isoform 2 of Actin-related protein 2 OS=Homo sapiens GN=ACTR2	+	Cluster 4	2.114	0.008	P11908-2	PRPS2	Isoform 2 of Ribose-phosphate pyrophosphokinase 2 OS=Homo sapiens GN=PRPS2	+	Cluster 4	3.506	< 0.001
Q86YW5	TREML1	Trem-like transcript 1 protein OS=Homo sapiens GN=TREML1 PE=1 SV=2	+	Cluster 4	2.561	0.003	Q9ULV4-3	CORO1C	Isoform 3 of Coronin-1C OS=Homo sapiens GN=CORO1C	+	Cluster 4	1.870	0.013	P48506	GCLC	Glutamate--cysteine ligase catalytic subunit OS=Homo sapiens GN=GCLC PE=1 SV=2	+	Cluster 4	2.938	0.001
A0A087WV23	SH3BGRL3	SH3 domain-binding glutamic acid-rich-like protein 3 OS=Homo sapiens GN=SH3BGRL3 PE=1 SV=1	+	Cluster 4	2.433	0.004	P67936	TPM4	Tropomyosin alpha-4 chain OS=Homo sapiens GN=TPM4 PE=1 SV=3	+	Cluster 4	2.343	0.005	P28065	PSMB9	Proteasome subunit beta type-9 OS=Homo sapiens GN=PSMB9 PE=1 SV=2	+	Cluster 4	1.639	0.023
P52566	ARHGDIB	Rho GDP-dissociation inhibitor 2 OS=Homo sapiens GN=ARHGDIB PE=1 SV=3	+	Cluster 4	3.336	< 0.001	P31946	YWHAB	14-3-3 protein beta/alpha OS=Homo sapiens GN=YWHAB PE=1 SV=3	+	Cluster 4	2.161	0.007	P05109	S100A8	Protein S100-A8 OS=Homo sapiens GN=S100A8 PE=1 SV=1	+	Cluster 4	1.661	0.022

P50395	GDI2	Rab GDP dissociation inhibitor beta OS=Homo sapiens GN=GDI2 PE=1 SV=2	+	Cluster 4	2.400	0.004	Q13642-4	FHL1	Isoform 4 of Four and a half LIM domains protein 1 OS=Homo sapiens GN=FHL1	+	Cluster 4	2.182	0.007	Q16394	EXT1	Exostosin-1 OS=Homo sapiens GN=EXT1 PE=1 SV=2	+	Cluster 4	1.577	0.026
P30041	PRDX6	Peroxiredoxin-6 OS=Homo sapiens GN=PRDX6 PE=1 SV=3	+	Cluster 4	3.113	< 0.001	P09211	GSTP1	Glutathione S-transferase P OS=Homo sapiens GN=GSTP1 PE=1 SV=2	+	Cluster 4	1.361	0.044	O15144	ARPC2	Actin-related protein 2/3 complex subunit 2 OS=Homo sapiens GN=ARPC2 PE=1 SV=1	+	Cluster 4	1.702	0.020
Q14623	IHH	Indian hedgehog protein OS=Homo sapiens GN=IHH PE=1 SV=4	+	Cluster 4	2.351	0.004	P55072	VCP	Transitional endoplasmic reticulum ATPase OS=Homo sapiens GN=VCP PE=1 SV=4	+	Cluster 4	1.329	0.047	A0A087WYJ9	IGHM	Ig mu chain C region OS=Homo sapiens GN=IGHM PE=1 SV=1	+	Cluster 4	2.752	0.002
P00338-3	LDHA	Isoform 3 of L-lactate dehydrogenase A chain OS=Homo sapiens GN=LDHA	+	Cluster 4	4.004	< 0.001	E7EMB3	CALM2	Calmodulin OS=Homo sapiens GN=CALM2 PE=1 SV=1	+	Cluster 4	2.251	0.006	P30740	SERPINB1	Leukocyte elastase inhibitor OS=Homo sapiens GN=SERPINB1 PE=1 SV=1	+	Cluster 4	3.012	< 0.001
P48059-3	LIMS1	Isoform 3 of LIM and senescent cell antigen-like-containing domain protein 1 OS=Homo sapiens GN=LIMS1	+	Cluster 4	4.553	< 0.001	P28070	PSMB4	Proteasome subunit beta type-4 OS=Homo sapiens GN=PSMB4 PE=1 SV=4	+	Cluster 4	1.585	0.026	F6TLX2	GLOD4	Glyoxalase domain-containing protein 4 OS=Homo sapiens GN=GLOD4 PE=1 SV=1	+	Cluster 4	1.502	0.031
E9PK25	CFL1	Cofilin-1 OS=Homo sapiens GN=CFL1 PE=1 SV=1	+	Cluster 4	2.780	0.002	A0A087WV23	SH3BGRL3	SH3 domain-binding glutamic acid-rich-like protein 3 OS=Homo sapiens GN=SH3BGRL3 PE=1 SV=1	+	Cluster 4	3.812	< 0.001	P14780	MMP9	Matrix metalloproteinase-9 OS=Homo sapiens GN=MMP9 PE=1 SV=3	+	Cluster 4	2.691	0.002
Q562R1	ACTBL2	Beta-actin-like protein 2 OS=Homo sapiens GN=ACTBL2 PE=1 SV=2	+	Cluster 4	3.457	< 0.001	P07738	BPGM	Bisphosphoglycerate mutase OS=Homo sapiens GN=BPGM PE=1 SV=2	+	Cluster 4	2.691	0.002	Q3ZCW2	LGALSL	Galectin-related protein OS=Homo sapiens GN=LGALSL PE=1 SV=2	+	Cluster 4	2.819	0.002
P23284	PPIB	Peptidyl-prolyl cis-trans isomerase B OS=Homo sapiens GN=PPIB PE=1 SV=2	+	Cluster 4	2.819	0.002	A0A087X130	IGKC	Ig kappa chain C region OS=Homo sapiens GN=IGKC PE=4 SV=1	+	Cluster 4	3.411	< 0.001	P06727	APOA4	Apolipoprotein A-IV OS=Homo sapiens GN=APOA4 PE=1 SV=3	+	Cluster 4	1.724	0.019
P30043	BLVRB	Flavin reductase (NADPH) OS=Homo sapiens GN=BLVRB PE=1 SV=3	+	Cluster 4	2.933	0.001	P49721	PSMB2	Proteasome subunit beta type-2 OS=Homo sapiens GN=PSMB2 PE=1 SV=1	+	Cluster 4	1.959	0.011	Q14847-2	LASP1	Isoform 2 of LIM and SH3 domain protein 1 OS=Homo sapiens GN=LASP1	+	Cluster 4	2.757	0.002
P07384	CAPN1	Calpain-1 catalytic subunit OS=Homo sapiens GN=CAPN1 PE=1 SV=1	+	Cluster 4	4.457	< 0.001	Q5T9B7	AK1	Adenylate kinase isoenzyme 1 OS=Homo sapiens GN=AK1 PE=1 SV=1	+	Cluster 4	3.982	< 0.001	P14618	PKM	Pyruvate kinase PKM OS=Homo sapiens GN=PKM PE=1 SV=4	+	Cluster 4	2.045	0.009
Q15942	ZYX	Zyxin OS=Homo sapiens GN=ZYX PE=1 SV=1	+	Cluster 4	3.171	< 0.001	P05362	ICAM1	Intercellular adhesion molecule 1 OS=Homo sapiens GN=ICAM1 PE=1 SV=2	+	Cluster 4	2.250	0.006	Q04760	GLO1	Lactoylglutathione lyase OS=Homo sapiens GN=GLO1 PE=1 SV=4	+	Cluster 4	1.688	0.021
Q9Y490	TLN1	Talin-1 OS=Homo sapiens GN=TLN1 PE=1 SV=3	+	Cluster 4	3.827	< 0.001	Q9BS26	ERP44	Endoplasmic reticulum resident protein 44 OS=Homo sapiens GN=ERP44 PE=1 SV=1	+	Cluster 4	2.695	0.002	Q13418	ILK	Integrin-linked protein kinase OS=Homo sapiens GN=ILK PE=1 SV=2	+	Cluster 4	3.056	< 0.001
P30626	SRI	Sorcin OS=Homo sapiens GN=SRI PE=1 SV=1	+	Cluster 4	2.648	0.002	Q8N392	ARHGAP18	Rho GTPase-activating protein 18 OS=Homo sapiens GN=ARHGAP18 PE=1 SV=3	+	Cluster 4	1.756	0.018	P26447	S100A4	Protein S100-A4 OS=Homo sapiens GN=S100A4 PE=1 SV=1	+	Cluster 4	2.503	0.003
P06753-2	TPM3	Isoform 2 of Tropomyosin alpha-3 chain OS=Homo sapiens GN=TPM3	+	Cluster 4	2.586	0.003	B4DUT8	CNN2	Calponin OS=Homo sapiens GN=CNN2 PE=1 SV=1	+	Cluster 4	2.107	0.008	P60174	TPI1	Triosephosphate isomerase OS=Homo sapiens GN=TPI1 PE=1 SV=3	+	Cluster 4	2.967	0.001
P59998-3	ARPC4	Isoform 3 of Actin-related protein 2/3 complex subunit 4 OS=Homo sapiens GN=ARPC4	+	Cluster 4	2.847	0.001	O95810	SDPR	Serum deprivation-response protein OS=Homo sapiens GN=SDPR PE=1 SV=3	+	Cluster 4	1.973	0.011	O00151	PDLIM1	PDZ and LIM domain protein 1 OS=Homo sapiens GN=PDLIM1 PE=1 SV=4	+	Cluster 4	4.101	< 0.001
O00299	CLIC1	Chloride intracellular channel protein 1 OS=Homo sapiens GN=CLIC1 PE=1 SV=4	+	Cluster 4	3.156	< 0.001	Q9HCU0	CD248	Endosialin OS=Homo sapiens GN=CD248 PE=1 SV=1	+	Cluster 4	2.598	0.003	P15531-2	NME1	Isoform 2 of Nucleoside diphosphate kinase A OS=Homo sapiens GN=NME1	+	Cluster 4	3.576	< 0.001
P61158	ACTR3	Actin-related protein 3 OS=Homo sapiens GN=ACTR3 PE=1 SV=3	+	Cluster 4	3.457	< 0.001	P60174	TPI1	Triosephosphate isomerase OS=Homo sapiens GN=TPI1 PE=1 SV=3	+	Cluster 4	2.963	0.001	P50990	CCT8	T-complex protein 1 subunit theta OS=Homo sapiens GN=CCT8 PE=1 SV=4	+	Cluster 4	2.313	0.005
P02776	PF4	Platelet factor 4 OS=Homo sapiens GN=PF4 PE=1 SV=2	+	Cluster 4	2.442	0.004	P50552	VASP	Vasodilator-stimulated phosphoprotein OS=Homo sapiens GN=VASP PE=1 SV=3	+	Cluster 4	2.312	0.005	O60234	GMFG	Glia maturation factor gamma OS=Homo sapiens GN=GMFG PE=1 SV=1	+	Cluster 4	3.184	< 0.001
Q13228-4	SELENBP1	Isoform 4 of Selenium-binding protein 1 OS=Homo sapiens GN=SELENBP1	+	Cluster 4	2.448	0.004	P32754	HPD	4-hydroxyphenylpyruvate dioxygenase OS=Homo sapiens GN=HPD PE=1 SV=2	+	Cluster 4	2.087	0.008	P12814-3	ACTN1	Isoform 3 of Alpha-actinin-1 OS=Homo sapiens GN=ACTN1	+	Cluster 4	2.087	0.008
Q9H0U4	RAB1B	Ras-related protein Rab-1B OS=Homo sapiens GN=RAB1B PE=1 SV=1	+	Cluster 4	3.505	< 0.001	P11908-2	PRPS2	Isoform 2 of Ribose-phosphate pyrophosphokinase 2 OS=Homo sapiens GN=PRPS2	+	Cluster 4	2.858	0.001	P63104	YWHAZ	14-3-3 protein zeta/delta OS=Homo sapiens GN=YWHAZ PE=1 SV=1	+	Cluster 4	1.321	0.048
P29966	MARCKS	Myristoylated alanine-rich C-kinase substrate OS=Homo sapiens GN=MARCKS PE=1 SV=4	+	Cluster 4	2.817	0.002	G3V2W1	SERPINA10	Protein Z-dependent protease inhibitor OS=Homo sapiens GN=SERPINA10 PE=3 SV=1	+	Cluster 4	2.060	0.009	O75083	WDR1	WD repeat-containing protein 1 OS=Homo sapiens GN=WDR1 PE=1 SV=4	+	Cluster 4	1.369	0.043
P0DJI8	SAA1	Serum amyloid A-1 protein OS=Homo sapiens GN=SAA1 PE=1 SV=1	+	Cluster 4	3.969	< 0.001	P40197	GP5	Platelet glycoprotein V OS=Homo sapiens GN=GP5 PE=1 SV=1	+	Cluster 4	2.118	0.008	Q9BYE9	CDHR2	Cadherin-related family member 2 OS=Homo sapiens GN=CDHR2 PE=1 SV=2	+	Cluster 4	1.921	0.012
P52209	PGD	6-phosphogluconate dehydrogenase, decarboxylating OS=Homo sapiens GN=PGD PE=1 SV=3	+	Cluster 4	3.294	< 0.001	Q9BYE9	CDHR2	Cadherin-related family member 2 OS=Homo sapiens GN=CDHR2 PE=1 SV=2	+	Cluster 4	1.627	0.024	P68363	TUBA1B	Tubulin alpha-1B chain OS=Homo sapiens GN=TUBA1B PE=1 SV=1	+	Cluster 4	1.733	0.018
P68366	TUBA4A	Tubulin alpha-4A chain OS=Homo sapiens GN=TUBA4A PE=1 SV=1	+	Cluster 4	4.108	< 0.001	Q96FW1-2	OTUB1	Isoform 2 of Ubiquitin thioesterase OTUB1 OS=Homo sapiens GN=OTUB1	+	Cluster 4	1.393	0.040	A0A087WXC9	MADCAM1	Mucosal addressin cell adhesion molecule 1 OS=Homo sapiens GN=MADCAM1 PE=4 SV=1	+	Cluster 4	1.592	0.026
P40925-3	MDH1	Isoform 3 of Malate dehydrogenase, cytoplasmic OS=Homo sapiens GN=MDH1	+	Cluster 4	2.913	0.001	Q14847-2	LASP1	Isoform 2 of LIM and SH3 domain protein 1 OS=Homo sapiens GN=LASP1	+	Cluster 4	2.514	0.003	J3KQ18	DDT	D-dopachrome decarboxylase OS=Homo sapiens GN=DDT PE=1 SV=1	+	Cluster 4	3.596	< 0.001
Q9NVA2-2	SEPT11	Isoform 2 of Septin-11 OS=Homo sapiens GN=SEPT11	+	Cluster 4	3.163	< 0.001	P14618	PKM	Pyruvate kinase PKM OS=Homo sapiens GN=PKM PE=1 SV=4	+	Cluster 4	1.788	0.016	P08238	HSP90AB1	Heat shock protein HSP 90-beta OS=Homo sapiens GN=HSP90AB1 PE=1 SV=4	+	Cluster 4	1.891	0.013
P30044	PRDX5	Peroxiredoxin-5, mitochondrial OS=Homo sapiens GN=PRDX5 PE=1 SV=4	+	Cluster 4	2.557	0.003	K7ER74	APOC4-APOC2	Protein APOC4-APOC2 OS=Homo sapiens GN=APOC4-APOC2 PE=4 SV=1	+	Cluster 4	3.192	< 0.001	Q14247	CTTN	Src substrate cortactin OS=Homo sapiens GN=CTTN PE=1 SV=2	+	Cluster 4	2.486	0.003
J3KQ32	OLA1	Obg-like ATPase 1 OS=Homo sapiens GN=OLA1 PE=1 SV=1	+	Cluster 4	2.945	0.001	Q07960	ARHGAP1	Rho GTPase-activating protein 1 OS=Homo sapiens GN=ARHGAP1 PE=1 SV=1	+	Cluster 4	2.890	0.001	Q9BS40	LXN	Latexin OS=Homo sapiens GN=LXN PE=1 SV=2	+	Cluster 4	1.914	0.012
P30740	SERPINB1	Leukocyte elastase inhibitor OS=Homo sapiens GN=SERPINB1 PE=1 SV=1	+	Cluster 4	3.106	< 0.001	O60234	GMFG	Glia maturation factor gamma OS=Homo sapiens GN=GMFG PE=1 SV=1	+	Cluster 4	3.243	< 0.001	P50991	CCT4	T-complex protein 1 subunit delta OS=Homo sapiens GN=CCT4 PE=1 SV=4	+	Cluster 4	1.347	0.045
P54578	USP14	Ubiquitin carboxyl-terminal hydrolase 14 OS=Homo sapiens GN=USP14 PE=1 SV=3	+	Cluster 4	3.432	< 0.001	P07900-2	HSP90AA1	Isoform 2 of Heat shock protein HSP 90-alpha OS=Homo sapiens GN=HSP90AA1	+	Cluster 4	3.807	< 0.001	P52907	CAPZA1	F-actin-capping protein subunit alpha-1 OS=Homo sapiens GN=CAPZA1 PE=1 SV=3	+	Cluster 4	2.170	0.007
P07900-2	HSP90AA1	Isoform 2 of Heat shock protein HSP 90-alpha OS=Homo sapiens GN=HSP90AA1	+	Cluster 4	3.522	< 0.001	Q9BR76	CORO1B	Coronin-1B OS=Homo sapiens GN=CORO1B PE=1 SV=1	+	Cluster 4	1.828	0.015	Q9NZN3	EHD3	EH domain-containing protein 3 OS=Homo sapiens GN=EHD3 PE=1 SV=2	+	Cluster 4	2.136	0.007
Q13418	ILK	Integrin-linked protein kinase OS=Homo sapiens GN=ILK PE=1 SV=2	+	Cluster 4	3.199	< 0.001	P50990	CCT8	T-complex protein 1 subunit theta OS=Homo sapiens GN=CCT8 PE=1 SV=4	+	Cluster 4	2.417	0.004	P12931-2	SRC	Isoform 2 of Proto-oncogene tyrosine-protein kinase Src OS=Homo sapiens GN=SRC	+	Cluster 4	1.539	0.029
P02741	CRP	C-reactive protein OS=Homo sapiens GN=CRP PE=1 SV=1	+	Cluster 4	4.278	< 0.001	P12814-3	ACTN1	Isoform 3 of Alpha-actinin-1 OS=Homo sapiens GN=ACTN1	+	Cluster 4	2.428	0.004	O75874	IDH1	Isocitrate dehydrogenase [NADP] cytoplasmic OS=Homo sapiens GN=IDH1 PE=1 SV=2	+	Cluster 4	1.469	0.034
P18085	ARF4	ADP-ribosylation factor 4 OS=Homo sapiens GN=ARF4 PE=1 SV=3	+	Cluster 4	3.220	< 0.001	P68363	TUBA1B	Tubulin alpha-1B chain OS=Homo sapiens GN=TUBA1B PE=1 SV=1	+	Cluster 4	2.171	0.007	Q562R1	ACTBL2	Beta-actin-like protein 2 OS=Homo sapiens GN=ACTBL2 PE=1 SV=2	+	Cluster 4	2.712	0.002
P07195	LDHB	L-lactate dehydrogenase B chain OS=Homo sapiens GN=LDHB PE=1 SV=2	+	Cluster 4	3.571	< 0.001	P50991	CCT4	T-complex protein 1 subunit delta OS=Homo sapiens GN=CCT4 PE=1 SV=4	+	Cluster 4	1.595	0.025	P41226	UBA7	Ubiquitin-like modifier-activating enzyme 7 OS=Homo sapiens GN=UBA7 PE=1 SV=2	+	Cluster 4	1.474	0.034
J3KQ18	DDT	D-dopachrome decarboxylase OS=Homo sapiens GN=DDT PE=1 SV=1	+	Cluster 4	3.498	< 0.001	Q9BS40	LXN	Latexin OS=Homo sapiens GN=LXN PE=1 SV=2	+	Cluster 4	2.064	0.009	P61160-2	ACTR2	Isoform 2 of Actin-related protein 2 OS=Homo sapiens GN=ACTR2	+	Cluster 4	2.208	0.006
Q15404	RSU1	Ras suppressor protein 1 OS=Homo sapiens GN=RSU1 PE=1 SV=3	+	Cluster 4	5.367	< 0.001	A0A087WXC9	MADCAM1	Mucosal addressin cell adhesion molecule 1 OS=Homo sapiens GN=MADCAM1 PE=4 SV=1	+	Cluster 4	1.437	0.037	Q15084-2	PDIA6	Isoform 2 of Protein disulfide-isomerase A6 OS=Homo sapiens GN=PDIA6	+	Cluster 4	2.496	0.003
P08567	PLEK	Pleckstrin OS=Homo sapiens GN=PLEK PE=1 SV=3	+	Cluster 4	5.353	< 0.001	P50395	GDI2	Rab GDP dissociation inhibitor beta OS=Homo sapiens GN=GDI2 PE=1 SV=2	+	Cluster 4	3.465	< 0.001	P15085	CPA1	Carboxypeptidase A1 OS=Homo sapiens GN=CPA1 PE=1 SV=2	+	Cluster 4	1.985	0.010
P78417	GSTO1	Glutathione S-transferase omega-1 OS=Homo sapiens GN=GSTO1 PE=1 SV=2	+	Cluster 4	5.038	< 0.001	Q14247	CTTN	Src substrate cortactin OS=Homo sapiens GN=CTTN PE=1 SV=2	+	Cluster 4	2.489	0.003	Q13228-4	SELENBP1	Isoform 4 of Selenium-binding protein 1 OS=Homo sapiens GN=SELENBP1	+	Cluster 4	1.922	0.012
P08758	ANXA5	Annexin A5 OS=Homo sapiens GN=ANXA5 PE=1 SV=2	+	Cluster 4	4.209	< 0.001	J3KQ18	DDT	D-dopachrome decarboxylase OS=Homo sapiens GN=DDT PE=1 SV=1	+	Cluster 4	3.311	< 0.001	Q9H0U4	RAB1B	Ras-related protein Rab-1B OS=Homo sapiens GN=RAB1B PE=1 SV=1	+	Cluster 4	2.682	0.002
P26038	MSN	Moesin OS=Homo sapiens GN=MSN PE=1 SV=3	+	Cluster 4	5.569	< 0.001	O15145	ARPC3	Actin-related protein 2/3 complex subunit 3 OS=Homo sapiens GN=ARPC3 PE=1 SV=3	+	Cluster 4	1.966	0.011	P09972	ALDOC	Fructose-bisphosphate aldolase C OS=Homo sapiens GN=ALDOC PE=1 SV=2	+	Cluster 4	3.335	< 0.001

Q9HBI1-2	PARVB	Isoform 2 of Beta-parvin OS=Homo sapiens GN=PARVB	+	Cluster 4	5.120	< 0.001	Q9NZN3	EHD3	EH domain-containing protein 3 OS=Homo sapiens GN=EHD3 PE=1 SV=2	+	Cluster 4	2.390	0.004	P61158	ACTR3	Actin-related protein 3 OS=Homo sapiens GN=ACTR3 PE=1 SV=3	+	Cluster 4	3.670	< 0.001
Q86UX7	FERMT3	Fermitin family homolog 3 OS=Homo sapiens GN=FERMT3 PE=1 SV=1	+	Cluster 4	7.928	< 0.001	P08758	ANXA5	Annexin A5 OS=Homo sapiens GN=ANXA5 PE=1 SV=2	+	Cluster 4	2.943	0.001	Q15942	ZYX	Zyxin OS=Homo sapiens GN=ZYX PE=1 SV=1	+	Cluster 4	2.947	0.001
P60709	ACTB	Actin, cytoplasmic 1 OS=Homo sapiens GN=ACTB PE=1 SV=1	+	Cluster 4	7.000	< 0.001	P00915	CA1	Carbonic anhydrase 1 OS=Homo sapiens GN=CA1 PE=1 SV=2	+	Cluster 4	5.114	< 0.001	P21333	FLNA	Filamin-A OS=Homo sapiens GN=FLNA PE=1 SV=4	+	Cluster 4	2.940	0.001
P11216	PYGB	Glycogen phosphorylase, brain form OS=Homo sapiens GN=PYGB PE=1 SV=5	+	Cluster 4	4.780	< 0.001	Q05682	CALD1	Caldesmon OS=Homo sapiens GN=CALD1 PE=1 SV=3	+	Cluster 4	3.996	< 0.001	P68032	ACTC1	Actin, alpha cardiac muscle 1 OS=Homo sapiens GN=ACTC1 PE=1 SV=1	+	Cluster 4	2.658	0.002
P60981	DSTN	Destrin OS=Homo sapiens GN=DSTN PE=1 SV=3	+	Cluster 4	3.994	< 0.001	P30043	BLVRB	Flavin reductase (NADPH) OS=Homo sapiens GN=BLVRB PE=1 SV=3	+	Cluster 4	3.831	< 0.001	P23284	PPIB	Peptidyl-prolyl cis-trans isomerase B OS=Homo sapiens GN=PPIB PE=1 SV=2	+	Cluster 4	3.374	< 0.001
P61981	YWHAG	14-3-3 protein gamma OS=Homo sapiens GN=YWHAG PE=1 SV=2	+	Cluster 4	3.197	< 0.001	P06753-2	TPM3	Isoform 2 of Tropomyosin alpha-3 chain OS=Homo sapiens GN=TPM3	+	Cluster 4	3.223	< 0.001	A0A075B6J8	IGLV3-19	Protein IGLV3-19 (Fragment) OS=Homo sapiens GN=IGLV3-19 PE=4 SV=1	+	Cluster 4	1.581	0.026
P62937	PPIA	Peptidyl-prolyl cis-trans isomerase A OS=Homo sapiens GN=PPIA PE=1 SV=2	+	Cluster 4	5.331	< 0.001	P68366	TUBA4A	Tubulin alpha-4A chain OS=Homo sapiens GN=TUBA4A PE=1 SV=1	+	Cluster 4	4.676	< 0.001	Q9BR76	CORO1B	Coronin-1B OS=Homo sapiens GN=CORO1B PE=1 SV=1	+	Cluster 4	1.791	0.016
P07737	PFN1	Profilin-1 OS=Homo sapiens GN=PFN1 PE=1 SV=2	+	Cluster 4	7.060	< 0.001	P02042	HBD	Hemoglobin subunit delta OS=Homo sapiens GN=HBD PE=1 SV=2	+	Cluster 4	3.746	< 0.001	Q7L9L4-2	MOB1B	Isoform 2 of MOB kinase activator 1B OS=Homo sapiens GN=MOB1B	+	Cluster 4	2.046	0.009
P68871	HBB	Hemoglobin subunit beta OS=Homo sapiens GN=HBB PE=1 SV=2	+	Cluster 4	7.783	< 0.001	P58546	MTPN	Myotrophin OS=Homo sapiens GN=MTPN PE=1 SV=2	+	Cluster 4	4.171	< 0.001	Q8N392	ARHGAP18	Rho GTPase-activating protein 18 OS=Homo sapiens GN=ARHGAP18 PE=1 SV=3	+	Cluster 4	3.070	< 0.001
P69905	HBA1	Hemoglobin subunit alpha OS=Homo sapiens GN=HBA1 PE=1 SV=2	+	Cluster 4	6.902	< 0.001	P30041	PRDX6	Peroxiredoxin-6 OS=Homo sapiens GN=PRDX6 PE=1 SV=3	+	Cluster 4	5.250	< 0.001	J3KQ32	OLA1	Obg-like ATPase 1 OS=Homo sapiens GN=OLA1 PE=1 SV=1	+	Cluster 4	3.462	< 0.001
P37802-2	TAGLN2	Isoform 2 of Transgelin-2 OS=Homo sapiens GN=TAGLN2	+	Cluster 4	5.571	< 0.001	P02741	CRP	C-reactive protein OS=Homo sapiens GN=CRP PE=1 SV=1	+	Cluster 4	4.917	< 0.001	P02753	RBP4	Retinol-binding protein 4 OS=Homo sapiens GN=RBP4 PE=1 SV=3	+	Cluster 4	2.452	0.004
P11142	HSPA8	Heat shock cognate 71 kDa protein OS=Homo sapiens GN=HSPA8 PE=1 SV=1	+	Cluster 4	5.189	< 0.001	P07384	CAPN1	Calpain-1 catalytic subunit OS=Homo sapiens GN=CAPN1 PE=1 SV=1	+	Cluster 4	4.281	< 0.001	O75915	ARL6IP5	PRA1 family protein 3 OS=Homo sapiens GN=ARL6IP5 PE=1 SV=1	+	Cluster 4	1.841	0.014
Q3ZCW2	LGALSL	Galectin-related protein OS=Homo sapiens GN=LGALSL PE=1 SV=2	+	Cluster 4	4.342	< 0.001	P36871	PGM1	Phosphoglucomutase-1 OS=Homo sapiens GN=PGM1 PE=1 SV=3	+	Cluster 4	3.863	< 0.001	P08758	ANXA5	Annexin A5 OS=Homo sapiens GN=ANXA5 PE=1 SV=2	+	Cluster 4	2.576	0.003
Q8NBP7	PCSK9	Proprotein convertase subtilisin/kexin type 9 OS=Homo sapiens GN=PCSK9 PE=1 SV=3	+	Cluster 4	4.124	< 0.001	J3KQ32	OLA1	Obg-like ATPase 1 OS=Homo sapiens GN=OLA1 PE=1 SV=1	+	Cluster 4	3.192	< 0.001	P0DJI8	SAA1	Serum amyloid A-1 protein OS=Homo sapiens GN=SAA1 PE=1 SV=1	+	Cluster 4	2.727	0.002
P62258	YWHAE	14-3-3 protein epsilon OS=Homo sapiens GN=YWHAE PE=1 SV=1	+	Cluster 4	4.205	< 0.001	P54578	USP14	Ubiquitin carboxyl-terminal hydrolase 14 OS=Homo sapiens GN=USP14 PE=1 SV=3	+	Cluster 4	3.813	< 0.001	Q01518	CAP1	Adenylyl cyclase-associated protein 1 OS=Homo sapiens GN=CAP1 PE=1 SV=5	+	Cluster 4	1.414	0.039
O00533-2	CHL1	Isoform 2 of Neural cell adhesion molecule L1-like protein OS=Homo sapiens GN=CHL1	+	Cluster 4	1.432	0.037	Q9NVA2-2	SEPT11	Isoform 2 of Septin-11 OS=Homo sapiens GN=SEPT11	+	Cluster 4	3.217	< 0.001	Q14019	COTL1	Coactosin-like protein OS=Homo sapiens GN=COTL1 PE=1 SV=3	+	Cluster 4	1.775	0.017
P07996	THBS1	Thrombospondin-1 OS=Homo sapiens GN=THBS1 PE=1 SV=2	+	Cluster 4	1.955	0.011	P52566	ARHGDIB	Rho GDP-dissociation inhibitor 2 OS=Homo sapiens GN=ARHGDIB PE=1 SV=3	+	Cluster 4	3.839	< 0.001	O14818	PSMA7	Proteasome subunit alpha type-7 OS=Homo sapiens GN=PSMA7 PE=1 SV=1	+	Cluster 4	1.869	0.014
P07237	P4HB	Protein disulfide-isomerase OS=Homo sapiens GN=P4HB PE=1 SV=3	+	Cluster 4	1.532	0.029	P61981	YWHAG	14-3-3 protein gamma OS=Homo sapiens GN=YWHAG PE=1 SV=2	+	Cluster 4	5.011	< 0.001	Q14520	HABP2	Hyaluronan-binding protein 2 OS=Homo sapiens GN=HABP2 PE=1 SV=1	+	Cluster 4	1.769	0.017
O14818	PSMA7	Proteasome subunit alpha type-7 OS=Homo sapiens GN=PSMA7 PE=1 SV=1	+	Cluster 4	1.520	0.030	P11021	HSPA5	78 kDa glucose-regulated protein OS=Homo sapiens GN=HSPA5 PE=1 SV=2	+	Cluster 4	2.791	0.002	P16109	SELP	P-selectin OS=Homo sapiens GN=SELP PE=1 SV=3	+	Cluster 4	1.394	0.040
P15085	CPA1	Carboxypeptidase A1 OS=Homo sapiens GN=CPA1 PE=1 SV=2	+	Cluster 4	1.885	0.013	P62979	RPS27A	Ubiquitin-40S ribosomal protein S27a OS=Homo sapiens GN=RPS27A PE=1 SV=2	+	Cluster 4	3.229	< 0.001	P28070	PSMB4	Proteasome subunit beta type-4 OS=Homo sapiens GN=PSMB4 PE=1 SV=4	+	Cluster 4	1.412	0.039
P09972	ALDOC	Fructose-bisphosphate aldolase C OS=Homo sapiens GN=ALDOC PE=1 SV=2	+	Cluster 4	3.366	< 0.001	P32119	PRDX2	Peroxiredoxin-2 OS=Homo sapiens GN=PRDX2 PE=1 SV=5	+	Cluster 4	3.098	< 0.001	P00918	CA2	Carbonic anhydrase 2 OS=Homo sapiens GN=CA2 PE=1 SV=2	+	Cluster 4	2.197	0.006
P21333	FLNA	Filamin-A OS=Homo sapiens GN=FLNA PE=1 SV=4	+	Cluster 4	2.794	0.002	P02775	PPBP	Platelet basic protein OS=Homo sapiens GN=PPBP PE=1 SV=3	+	Cluster 4	4.212	< 0.001	P07996	THBS1	Thrombospondin-1 OS=Homo sapiens GN=THBS1 PE=1 SV=2	+	Cluster 4	1.854	0.014
O95897	OLFM2	Noelin-2 OS=Homo sapiens GN=OLFM2 PE=2 SV=2	+	Cluster 4	3.081	< 0.001	O00151	PDLIM1	PDZ and LIM domain protein 1 OS=Homo sapiens GN=PDLIM1 PE=1 SV=4	+	Cluster 4	3.889	< 0.001	Q96KP4	CNDP2	Cytosolic non-specific dipeptidase OS=Homo sapiens GN=CNDP2 PE=1 SV=2	+	Cluster 4	1.635	0.023
P61160-2	ACTR2	Isoform 2 of Actin-related protein 2 OS=Homo sapiens GN=ACTR2	+	Cluster 4	2.131	0.007	P06733	ENO1	Alpha-enolase OS=Homo sapiens GN=ENO1 PE=1 SV=2	+	Cluster 4	3.854	< 0.001	Q14697-2	GANAB	Isoform 2 of Neutral alpha-glucosidase AB OS=Homo sapiens GN=GANAB	+	Cluster 4	1.422	0.038
Q9BS26	ERP44	Endoplasmic reticulum resident protein 44 OS=Homo sapiens GN=ERP44 PE=1 SV=1	+	Cluster 4	2.320	0.005	Q13418	ILK	Integrin-linked protein kinase OS=Homo sapiens GN=ILK PE=1 SV=2	+	Cluster 4	3.424	< 0.001	F5H2F4	MTHFD1	C-1-tetrahydrofolate synthase, cytoplasmic OS=Homo sapiens GN=MTHFD1 PE=1 SV=1	+	Cluster 4	1.897	0.013
Q13642-4	FHL1	Isoform 4 of Four and a half LIM domains protein 1 OS=Homo sapiens GN=FHL1	+	Cluster 4	1.981	0.010	P26038	MSN	Moesin OS=Homo sapiens GN=MSN PE=1 SV=3	+	Cluster 4	5.123	< 0.001	Q8WZ75	ROBO4	Roundabout homolog 4 OS=Homo sapiens GN=ROBO4 PE=1 SV=1	+	Cluster 4	1.575	0.027
P41226	UBA7	Ubiquitin-like modifier-activating enzyme 7 OS=Homo sapiens GN=UBA7 PE=1 SV=2	+	Cluster 4	1.502	0.032	P11142	HSPA8	Heat shock cognate 71 kDa protein OS=Homo sapiens GN=HSPA8 PE=1 SV=1	+	Cluster 4	4.804	< 0.001	P40189	IL6ST	Interleukin-6 receptor subunit beta OS=Homo sapiens GN=IL6ST PE=1 SV=2	+	Cluster 4	1.666	0.022
Q9ULV4-3	CORO1C	Isoform 3 of Coronin-1C OS=Homo sapiens GN=CORO1C	+	Cluster 4	1.903	0.013	Q86YW5	TREML1	Trem-like transcript 1 protein OS=Homo sapiens GN=TREML1 PE=1 SV=2	+	Cluster 4	4.657	< 0.001	Q9ULV4-3	CORO1C	Isoform 3 of Coronin-1C OS=Homo sapiens GN=CORO1C	+	Cluster 4	2.168	0.007
P67936	TPM4	Tropomyosin alpha-4 chain OS=Homo sapiens GN=TPM4 PE=1 SV=3	+	Cluster 4	1.915	0.012	P07195	LDHB	L-lactate dehydrogenase B chain OS=Homo sapiens GN=LDHB PE=1 SV=2	+	Cluster 4	3.640	< 0.001	O00533-2	CHL1	Isoform 2 of Neural cell adhesion molecule L1-like protein OS=Homo sapiens GN=CHL1	+	Cluster 4	1.818	0.015
P31946	YWHAB	14-3-3 protein beta/alpha OS=Homo sapiens GN=YWHAB PE=1 SV=3	+	Cluster 4	1.739	0.018	P30740	SERPINB1	Leukocyte elastase inhibitor OS=Homo sapiens GN=SERPINB1 PE=1 SV=1	+	Cluster 4	5.088	< 0.001	Q9BS26	ERP44	Endoplasmic reticulum resident protein 44 OS=Homo sapiens GN=ERP44 PE=1 SV=1	+	Cluster 4	2.606	0.002
							P61204	ARF3	ADP-ribosylation factor 3 OS=Homo sapiens GN=ARF3 PE=1 SV=2	+	Cluster 4	3.838	< 0.001	P23526	AHCY	Adenosylhomocysteinase OS=Homo sapiens GN=AHCY PE=1 SV=4	+	Cluster 4	2.263	0.005
							P00338-3	LDHA	Isoform 3 of L-lactate dehydrogenase A chain OS=Homo sapiens GN=LDHA	+	Cluster 4	4.996	< 0.001	Q9H4A9	DPEP2	Dipeptidase 2 OS=Homo sapiens GN=DPEP2 PE=1 SV=2	+	Cluster 4	1.316	0.048
							P62258	YWHAE	14-3-3 protein epsilon OS=Homo sapiens GN=YWHAE PE=1 SV=1	+	Cluster 4	4.351	< 0.001	P68036	UBE2L3	Ubiquitin-conjugating enzyme E2 L3 OS=Homo sapiens GN=UBE2L3 PE=1 SV=1	+	Cluster 4	2.322	0.005
							Q9Y490	TLN1	Talin-1 OS=Homo sapiens GN=TLN1 PE=1 SV=3	+	Cluster 4	4.338	< 0.001	P37837	TALDO1	Transaldolase OS=Homo sapiens GN=TALDO1 PE=1 SV=2	+	Cluster 4	2.241	0.006
							O15143	ARPC1B	Actin-related protein 2/3 complex subunit 1B OS=Homo sapiens GN=ARPC1B PE=1 SV=3	+	Cluster 4	4.091	< 0.001	P05362	ICAM1	Intercellular adhesion molecule 1 OS=Homo sapiens GN=ICAM1 PE=1 SV=2	+	Cluster 4	2.156	0.007
							P18085	ARF4	ADP-ribosylation factor 4 OS=Homo sapiens GN=ARF4 PE=1 SV=3	+	Cluster 4	5.504	< 0.001	A6NC48	BST1	ADP-ribosyl cyclase/cyclic ADP-ribose hydrolase 2 OS=Homo sapiens GN=BST1 PE=4 SV=1	+	Cluster 4	2.542	0.003
							Q86UX7	FERMT3	Fermitin family homolog 3 OS=Homo sapiens GN=FERMT3 PE=1 SV=1	+	Cluster 4	7.861	< 0.001	P27918	CFP	Properdin OS=Homo sapiens GN=CFP PE=1 SV=2	+	Cluster 4	2.366	0.004
							P37802-2	TAGLN2	Isoform 2 of Transgelin-2 OS=Homo sapiens GN=TAGLN2	+	Cluster 4	7.666	< 0.001	O95810	SDPR	Serum deprivation-response protein OS=Homo sapiens GN=SDPR PE=1 SV=3	+	Cluster 4	2.396	0.004
							P07737	PFN1	Profilin-1 OS=Homo sapiens GN=PFN1 PE=1 SV=2	+	Cluster 4	7.526	< 0.001	P61088	UBE2N	Ubiquitin-conjugating enzyme E2 N OS=Homo sapiens GN=UBE2N PE=1 SV=1	+	Cluster 4	2.185	0.007
							P62937	PPIA	Peptidyl-prolyl cis-trans isomerase A OS=Homo sapiens GN=PPIA PE=1 SV=2	+	Cluster 4	5.233	< 0.001	P49721	PSMB2	Proteasome subunit beta type-2 OS=Homo sapiens GN=PSMB2 PE=1 SV=1	+	Cluster 4	2.869	0.001
							Q15404	RSU1	Ras suppressor protein 1 OS=Homo sapiens GN=RSU1 PE=1 SV=3	+	Cluster 4	5.137	< 0.001	O75023-3	LILRB5	Isoform 3 of Leukocyte immunoglobulin-like receptor subfamily B member 5 OS=Homo sapiens GN=LILRB5	+	Cluster 4	2.139	0.007

P78417	GSTO1	Glutathione S-transferase omega-1 OS=Homo sapiens GN=GSTO1 PE=1 SV=2	+	Cluster 4	5.544	< 0.001	P01033	TIMP1	Metalloproteinase inhibitor 1 OS=Homo sapiens GN=TIMP1 PE=1 SV=1	+	Cluster 4	2.706	0.002
P48059-3	LIMS1	Isoform 3 of LIM and senescent cell antigen-like-containing domain protein 1 OS=Homo sapiens GN=LIMS1	+	Cluster 4	5.297	< 0.001	P62979	RPS27A	Ubiquitin-40S ribosomal protein S27a OS=Homo sapiens GN=RPS27A PE=1 SV=2	+	Cluster 4	3.725	< 0.001
P08567	PLEK	Pleckstrin OS=Homo sapiens GN=PLEK PE=1 SV=3	+	Cluster 4	5.812	< 0.001	P10599	TXN	Thioredoxin OS=Homo sapiens GN=TXN PE=1 SV=3	+	Cluster 4	3.690	< 0.001
P60709	ACTB	Actin, cytoplasmic 1 OS=Homo sapiens GN=ACTB PE=1 SV=1	+	Cluster 4	7.179	< 0.001	P11021	HSPA5	78 kDa glucose-regulated protein OS=Homo sapiens GN=HSPA5 PE=1 SV=2	+	Cluster 4	2.919	0.001
P69905	HBA1	Hemoglobin subunit alpha OS=Homo sapiens GN=HBA1 PE=1 SV=2	+	Cluster 4	7.554	< 0.001	P18085	ARF4	ADP-ribosylation factor 4 OS=Homo sapiens GN=ARF4 PE=1 SV=3	+	Cluster 4	3.356	< 0.001
P68871	HBB	Hemoglobin subunit beta OS=Homo sapiens GN=HBB PE=1 SV=2	+	Cluster 4	8.897	< 0.001	A8MU27	SUMO3	Small ubiquitin-related modifier 3 OS=Homo sapiens GN=SUMO3 PE=1 SV=1	+	Cluster 4	4.183	< 0.001
P60981	DSTN	Destrin OS=Homo sapiens GN=DSTN PE=1 SV=3	+	Cluster 4	4.879	< 0.001	P58546	MTPN	Myotrophin OS=Homo sapiens GN=MTPN PE=1 SV=2	+	Cluster 4	3.709	< 0.001
P11216	PYGB	Glycogen phosphorylase, brain form OS=Homo sapiens GN=PYGB PE=1 SV=5	+	Cluster 4	5.525	< 0.001	A0A087WV23	SH3BGRL3	SH3 domain-binding glutamic acid-rich-like protein 3 OS=Homo sapiens GN=SH3BGRL3 PE=1 SV=1	+	Cluster 4	5.172	< 0.001
P14324	FDPS	Farnesyl pyrophosphate synthase OS=Homo sapiens GN=FDPS PE=1 SV=4	+	Cluster 4	4.265	< 0.001	P69905	HBA1	Hemoglobin subunit alpha OS=Homo sapiens GN=HBA1 PE=1 SV=2	+	Cluster 4	7.497	< 0.001
Q9HBI1-2	PARVB	Isoform 2 of Beta-parvin OS=Homo sapiens GN=PARVB	+	Cluster 4	5.377	< 0.001	P68871	HBB	Hemoglobin subunit beta OS=Homo sapiens GN=HBB PE=1 SV=2	+	Cluster 4	8.114	< 0.001
P02776	PF4	Platelet factor 4 OS=Homo sapiens GN=PF4 PE=1 SV=2	+	Cluster 4	2.649	0.002	P02741	CRP	C-reactive protein OS=Homo sapiens GN=CRP PE=1 SV=1	+	Cluster 4	6.190	< 0.001
P45974	USP5	Ubiquitin carboxyl-terminal hydrolase 5 OS=Homo sapiens GN=USP5 PE=1 SV=2	+	Cluster 4	2.817	0.002	A0A075B6L0	IGLC3	Ig lambda-3 chain C regions (Fragment) OS=Homo sapiens GN=IGLC3 PE=4 SV=2	+	Cluster 4	4.305	< 0.001
P23284	PPIB	Peptidyl-prolyl cis-trans isomerase B OS=Homo sapiens GN=PPIB PE=1 SV=2	+	Cluster 4	2.854	0.001	P00915	CA1	Carbonic anhydrase 1 OS=Homo sapiens GN=CA1 PE=1 SV=2	+	Cluster 4	5.038	< 0.001
P59998-3	ARPC4	Isoform 3 of Actin-related protein 2/3 complex subunit 4 OS=Homo sapiens GN=ARPC4	+	Cluster 4	2.924	0.001	P32119	PRDX2	Peroxiredoxin-2 OS=Homo sapiens GN=PRDX2 PE=1 SV=5	+	Cluster 4	5.520	< 0.001
O00299	CLIC1	Chloride intracellular channel protein 1 OS=Homo sapiens GN=CLIC1 PE=1 SV=4	+	Cluster 4	3.474	< 0.001	P36871	PGM1	Phosphoglucomutase-1 OS=Homo sapiens GN=PGM1 PE=1 SV=3	+	Cluster 4	4.064	< 0.001
P40925-3	MDH1	Isoform 3 of Malate dehydrogenase, cytoplasmic OS=Homo sapiens GN=MDH1	+	Cluster 4	3.006	< 0.001	Q15404	RSU1	Ras suppressor protein 1 OS=Homo sapiens GN=RSU1 PE=1 SV=3	+	Cluster 4	5.596	< 0.001
Q15942	ZYX	Zyxin OS=Homo sapiens GN=ZYX PE=1 SV=1	+	Cluster 4	3.209	< 0.001	P00338-3	LDHA	Isoform 3 of L-lactate dehydrogenase A chain OS=Homo sapiens GN=LDHA	+	Cluster 4	4.269	< 0.001
P09972	ALDOC	Fructose-bisphosphate aldolase C OS=Homo sapiens GN=ALDOC PE=1 SV=2	+	Cluster 4	3.188	< 0.001	P48059-3	LIMS1	Isoform 3 of LIM and senescent cell antigen-like-containing domain protein 1 OS=Homo sapiens GN=LIMS1	+	Cluster 4	4.804	< 0.001
P16520	GNB3	Guanine nucleotide-binding protein G(I)/G(S)/G(T) subunit beta-3 OS=Homo sapiens GN=GNB3 PE=1 SV=1	+	Cluster 4	2.641	0.002	Q13642-4	FHL1	Isoform 4 of Four and a half LIM domains protein 1 OS=Homo sapiens GN=FHL1	+	Cluster 4	2.236	0.006
P30626	SRI	Sorcin OS=Homo sapiens GN=SRI PE=1 SV=1	+	Cluster 4	3.206	< 0.001	O00299	CLIC1	Chloride intracellular channel protein 1 OS=Homo sapiens GN=CLIC1 PE=1 SV=4	+	Cluster 4	3.850	< 0.001
P54577	YARS	Tyrosine--tRNA ligase, cytoplasmic OS=Homo sapiens GN=YARS PE=1 SV=4	+	Cluster 4	2.776	0.002	Q9Y490	TLN1	Talin-1 OS=Homo sapiens GN=TLN1 PE=1 SV=3	+	Cluster 4	4.034	< 0.001
P25786-2	PSMA1	Isoform Long of Proteasome subunit alpha type-1 OS=Homo sapiens GN=PSMA1	+	Cluster 4	1.352	0.045	P59998-3	ARPC4	Isoform 3 of Actin-related protein 2/3 complex subunit 4 OS=Homo sapiens GN=ARPC4	+	Cluster 4	3.459	< 0.001
E7EUC7	UGP2	UTP--glucose-1-phosphate uridylyltransferase OS=Homo sapiens GN=UGP2 PE=1 SV=1	+	Cluster 4	1.372	0.042	P40925-3	MDH1	Isoform 3 of Malate dehydrogenase, cytoplasmic OS=Homo sapiens GN=MDH1	+	Cluster 4	3.121	< 0.001
P14780	MMP9	Matrix metalloproteinase-9 OS=Homo sapiens GN=MMP9 PE=1 SV=3	+	Cluster 4	2.269	0.005	P30626	SRI	Sorcin OS=Homo sapiens GN=SRI PE=1 SV=1	+	Cluster 4	2.928	0.001
P02753	RBP4	Retinol-binding protein 4 OS=Homo sapiens GN=RBP4 PE=1 SV=3	+	Cluster 4	3.396	< 0.001	P02776	PF4	Platelet factor 4 OS=Homo sapiens GN=PF4 PE=1 SV=2	+	Cluster 4	4.494	< 0.001
P31150	GDI1	Rab GDP dissociation inhibitor alpha OS=Homo sapiens GN=GDI1 PE=1 SV=2	+	Cluster 4	1.644	0.023	Q9NVA2-2	SEPT11	Isoform 2 of Septin-11 OS=Homo sapiens GN=SEPT11	+	Cluster 4	3.330	< 0.001
P07339	CTSD	Cathepsin D OS=Homo sapiens GN=CTSD PE=1 SV=1	+	Cluster 4	1.942	0.011	P30044	PRDX5	Peroxiredoxin-5, mitochondrial OS=Homo sapiens GN=PRDX5 PE=1 SV=4	+	Cluster 4	3.103	< 0.001
P29966	MARCKS	Myristoylated alanine-rich C-kinase substrate OS=Homo sapiens GN=MARCKS PE=1 SV=4	+	Cluster 4	2.072	0.008	P54578	USP14	Ubiquitin carboxyl-terminal hydrolase 14 OS=Homo sapiens GN=USP14 PE=1 SV=3	+	Cluster 4	3.403	< 0.001
P20851	C4BPB	C4b-binding protein beta chain OS=Homo sapiens GN=C4BPB PE=1 SV=1	+	Cluster 4	2.335	0.005	P52209	PGD	6-phosphogluconate dehydrogenase, decarboxylating OS=Homo sapiens GN=PGD PE=1 SV=3	+	Cluster 4	2.823	0.002
P0C0L5	C4B	Complement C4-B OS=Homo sapiens GN=C4B PE=1 SV=2	+	Cluster 4	1.813	0.015	P69891	HBG1	Hemoglobin subunit gamma-1 OS=Homo sapiens GN=HBG1 PE=1 SV=2	+	Cluster 4	4.074	< 0.001
P15531-2	NME1	Isoform 2 of Nucleoside diphosphate kinase A OS=Homo sapiens GN=NME1	+	Cluster 4	3.077	< 0.001	P61981	YWHAG	14-3-3 protein gamma OS=Homo sapiens GN=YWHAG PE=1 SV=2	+	Cluster 4	3.158	< 0.001
O15144	ARPC2	Actin-related protein 2/3 complex subunit 2 OS=Homo sapiens GN=ARPC2 PE=1 SV=1	+	Cluster 4	1.909	0.012	P11216	PYGB	Glycogen phosphorylase, brain form OS=Homo sapiens GN=PYGB PE=1 SV=5	+	Cluster 4	4.339	< 0.001
Q9H0U4	RAB1B	Ras-related protein Rab-1B OS=Homo sapiens GN=RAB1B PE=1 SV=1	+	Cluster 4	3.675	< 0.001	P60981	DSTN	Destrin OS=Homo sapiens GN=DSTN PE=1 SV=3	+	Cluster 4	3.623	< 0.001
Q13228-4	SELENBP1	Isoform 4 of Selenium-binding protein 1 OS=Homo sapiens GN=SELENBP1	+	Cluster 4	2.551	0.003	P29350-4	PTPN6	Isoform 4 of Tyrosine-protein phosphatase non-receptor type 6 OS=Homo sapiens GN=PTPN6	+	Cluster 4	3.385	< 0.001
P61158	ACTR3	Actin-related protein 3 OS=Homo sapiens GN=ACTR3 PE=1 SV=3	+	Cluster 4	3.436	< 0.001	P11142	HSPA8	Heat shock cognate 71 kDa protein OS=Homo sapiens GN=HSPA8 PE=1 SV=1	+	Cluster 4	4.259	< 0.001
P52209	PGD	6-phosphogluconate dehydrogenase, decarboxylating OS=Homo sapiens GN=PGD PE=1 SV=3	+	Cluster 4	4.167	< 0.001	P60709	ACTB	Actin, cytoplasmic 1 OS=Homo sapiens GN=ACTB PE=1 SV=1	+	Cluster 4	6.765	< 0.001
P11766	ADH5	Alcohol dehydrogenase class-3 OS=Homo sapiens GN=ADH5 PE=1 SV=4	+	Cluster 4	3.393	< 0.001	Q86UX7	FERMT3	Fermitin family homolog 3 OS=Homo sapiens GN=FERMT3 PE=1 SV=1	+	Cluster 4	8.555	< 0.001
O14745	SLC9A3R1	Na(+)/H(+) exchange regulatory cofactor NHE-RF1 OS=Homo sapiens GN=SLC9A3R1 PE=1 SV=4	+	Cluster 4	2.987	0.001	P06733	ENO1	Alpha-enolase OS=Homo sapiens GN=ENO1 PE=1 SV=2	+	Cluster 4	5.449	< 0.001
K7ELL7	PRKCSH	Glucosidase 2 subunit beta OS=Homo sapiens GN=PRKCSH PE=1 SV=1	+	Cluster 4	2.611	0.002	P37802-2	TAGLN2	Isoform 2 of Transgelin-2 OS=Homo sapiens GN=TAGLN2	+	Cluster 4	5.530	< 0.001
E9PK25	CFL1	Cofilin-1 OS=Homo sapiens GN=CFL1 PE=1 SV=1	+	Cluster 4	4.338	< 0.001	Q9HBI1-2	PARVB	Isoform 2 of Beta-parvin OS=Homo sapiens GN=PARVB	+	Cluster 4	5.077	< 0.001
P68032	ACTC1	Actin, alpha cardiac muscle 1 OS=Homo sapiens GN=ACTC1 PE=1 SV=1	+	Cluster 4	3.402	< 0.001	P26038	MSN	Moesin OS=Homo sapiens GN=MSN PE=1 SV=3	+	Cluster 4	4.643	< 0.001

P61088	UBE2N	Ubiquitin-conjugating enzyme E2 N OS=Homo sapiens GN=UBE2N PE=1 SV=1	+	Cluster 4	3.731	< 0.001	P07195	LDHB	L-lactate dehydrogenase B chain OS=Homo sapiens GN=LDHB PE=1 SV=2	+	Cluster 4	4.418	< 0.001
P0DJI8	SAA1	Serum amyloid A-1 protein OS=Homo sapiens GN=SAA1 PE=1 SV=1	+	Cluster 4	3.799	< 0.001	P07384	CAPN1	Calpain-1 catalytic subunit OS=Homo sapiens GN=CAPN1 PE=1 SV=1	+	Cluster 4	4.297	< 0.001
Q562R1	ACTBL2	Beta-actin-like protein 2 OS=Homo sapiens GN=ACTBL2 PE=1 SV=2	+	Cluster 4	2.567	0.003	P78417	GSTO1	Glutathione S-transferase omega-1 OS=Homo sapiens GN=GSTO1 PE=1 SV=2	+	Cluster 4	4.795	< 0.001
Q96KP4	CNDP2	Cytosolic non-specific dipeptidase OS=Homo sapiens GN=CNDP2 PE=1 SV=2	+	Cluster 4	2.697	0.002	P62937	PPIA	Peptidyl-prolyl cis-trans isomerase A OS=Homo sapiens GN=PPIA PE=1 SV=2	+	Cluster 4	5.816	< 0.001
P61077-3	UBE2D3	Isoform 3 of Ubiquitin-conjugating enzyme E2 D3 OS=Homo sapiens GN=UBE2D3	+	Cluster 4	2.826	0.001	P08567	PLEK	Pleckstrin OS=Homo sapiens GN=PLEK PE=1 SV=3	+	Cluster 4	6.239	< 0.001
P68036	UBE2L3	Ubiquitin-conjugating enzyme E2 L3 OS=Homo sapiens GN=UBE2L3 PE=1 SV=1	+	Cluster 4	3.955	< 0.001	P31946	YWHAB	14-3-3 protein beta/alpha OS=Homo sapiens GN=YWHAB PE=1 SV=3	+	Cluster 4	2.070	0.009
Q3ZCW2	LGALSL	Galectin-related protein OS=Homo sapiens GN=LGALSL PE=1 SV=2	+	Cluster 4	5.110	< 0.001	E7EMB3	CALM2	Calmodulin OS=Homo sapiens GN=CALM2 PE=1 SV=1	+	Cluster 4	1.985	0.010
P18206	VCL	Vinculin OS=Homo sapiens GN=VCL PE=1 SV=4	+	Cluster 5	4.777	< 0.001	P67936	TPM4	Tropomyosin alpha-4 chain OS=Homo sapiens GN=TPM4 PE=1 SV=3	+	Cluster 4	2.342	0.005
P07357	C8A	Complement component C8 alpha chain OS=Homo sapiens GN=C8A PE=1 SV=2	+	Cluster 5	1.813	0.015	P52566	ARHGDIB	Rho GDP-dissociation inhibitor 2 OS=Homo sapiens GN=ARHGDIB PE=1 SV=3	+	Cluster 4	4.406	< 0.001
Q9Y251	HPSE	Heparanase OS=Homo sapiens GN=HPSE PE=1 SV=2	+	Cluster 5	2.736	0.002	P01137	TGFB1	Transforming growth factor beta-1 OS=Homo sapiens GN=TGFB1 PE=1 SV=2	+	Cluster 4	3.859	< 0.001
P01620		Ig kappa chain V-III region SIE OS=Homo sapiens PE=1 SV=1	+	Cluster 5	1.302	0.050	P02042	HBD	Hemoglobin subunit delta OS=Homo sapiens GN=HBD PE=1 SV=2	+	Cluster 4	3.601	< 0.001
P02749	APOH	Beta-2-glycoprotein 1 OS=Homo sapiens GN=APOH PE=1 SV=3	+	Cluster 5	3.017	< 0.001	P00352	ALDH1A1	Retinal dehydrogenase 1 OS=Homo sapiens GN=ALDH1A1 PE=1 SV=2	+	Cluster 4	3.773	< 0.001
P13645	KRT10	Keratin, type I cytoskeletal 10 OS=Homo sapiens GN=KRT10 PE=1 SV=6	+	Cluster 5	4.061	< 0.001	P06753-2	TPM3	Isoform 2 of Tropomyosin alpha-3 chain OS=Homo sapiens GN=TPM3	+	Cluster 4	3.218	< 0.001
P02747	C1QC	Complement C1q subcomponent subunit C OS=Homo sapiens GN=C1QC PE=1 SV=3	+	Cluster 5	2.329	0.005	P07737	PFN1	Profilin-1 OS=Homo sapiens GN=PFN1 PE=1 SV=2	+	Cluster 4	6.710	< 0.001
P05019	IGF1	Insulin-like growth factor I OS=Homo sapiens GN=IGF1 PE=1 SV=1	+	Cluster 5	2.827	0.001	P68366	TUBA4A	Tubulin alpha-4A chain OS=Homo sapiens GN=TUBA4A PE=1 SV=1	+	Cluster 4	4.553	< 0.001
P53396	ACLY	ATP-citrate synthase OS=Homo sapiens GN=ACLY PE=1 SV=3	+	Cluster 5	2.297	0.005	P18206	VCL	Vinculin OS=Homo sapiens GN=VCL PE=1 SV=4	+	Cluster 4	4.436	< 0.001
P08123	COL1A2	Collagen alpha-2(I) chain OS=Homo sapiens GN=COL1A2 PE=1 SV=7	+	Cluster 5	1.388	0.041	P30043	BLVRB	Flavin reductase (NADPH) OS=Homo sapiens GN=BLVRB PE=1 SV=3	+	Cluster 4	4.139	< 0.001
P01889	HLA-B	HLA class I histocompatibility antigen, B-7 alpha chain OS=Homo sapiens GN=HLA-B PE=1 SV=3	+	Cluster 5	1.637	0.023	P62258	YWHAE	14-3-3 protein epsilon OS=Homo sapiens GN=YWHAE PE=1 SV=1	+	Cluster 4	4.121	< 0.001
P12273	PIP	Prolactin-inducible protein OS=Homo sapiens GN=PIP PE=1 SV=1	+	Cluster 5	1.372	0.042	P30041	PRDX6	Peroxiredoxin-6 OS=Homo sapiens GN=PRDX6 PE=1 SV=3	+	Cluster 4	4.157	< 0.001
P35908	KRT2	Keratin, type II cytoskeletal 2 epidermal OS=Homo sapiens GN=KRT2 PE=1 SV=2	+	Cluster 5	1.342	0.045	A0A087WX49	IGLL5	Immunoglobulin lambda-like polypeptide 5 OS=Homo sapiens GN=IGLL5 PE=4 SV=1	+	Cluster 4	3.666	< 0.001
Q6UX71	PLXDC2	Plexin domain-containing protein 2 OS=Homo sapiens GN=PLXDC2 PE=1 SV=1	+	Cluster 5	2.222	0.006							
A0A087WVE2	FCN1	Ficolin-1 OS=Homo sapiens GN=FCN1 PE=4 SV=1	+	Cluster 5	2.548	0.003							
Q13790	APOF	Apolipoprotein F OS=Homo sapiens GN=APOF PE=1 SV=2	+	Cluster 5	1.918	0.012							
D6RAR4	HGFAC	Hepatocyte growth factor activator OS=Homo sapiens GN=HGFAC PE=3 SV=1	+	Cluster 5	1.818	0.015							
P55268	LAMB2	Laminin subunit beta-2 OS=Homo sapiens GN=LAMB2 PE=1 SV=2	+	Cluster 5	2.047	0.009							
V9GYM3	APOA2	Apolipoprotein A-II OS=Homo sapiens GN=APOA2 PE=1 SV=1	+	Cluster 5	3.228	< 0.001							
P16035	TIMP2	Metalloproteinase inhibitor 2 OS=Homo sapiens GN=TIMP2 PE=1 SV=2	+	Cluster 5	2.940	0.001							
P04264	KRT1	Keratin, type II cytoskeletal 1 OS=Homo sapiens GN=KRT1 PE=1 SV=6	+		3.529	< 0.001							

† Differentially expressed proteins (DEPs) for three comparison of groups (MDD vs BD vs HC, MDD vs SCZ vs HC, and BD vs SCZ vs HC) were determined by one-way ANOVA. *P-value* < 0.05 is considered as statistically significant, and -$\log_{10}$ transformed *P-value* and *P-value* for each protein are listed. Uniprot accession, gene symbol, protein name of DEPs are listed. In addition, cluster corresponding to each protein and statistical significance of each protein are signified.

Abbreviations

MDD: major depressive disorder, BD: bipolar disorder, SCZ: schizophrenia, HC: healthy control

Table S14. Proteins of MPM models overlapping with DEPs of proteomic profiling data for each pairwise comparison of groups†

MDD vs BD

Protein	Gene symbol	ANOVA Significant (MDD vs BD vs HC)	Clusters	Significance of post-hoc analysis (MDD vs BD)	P-value (Tukey's HSD)	Log_2(Fold-change)	Expression pattern	Consistency of statistical significance and expression pattern for disease types
ALDOC	ALDOC	+	cluster 4		0.727	0.46	BD up	N
**ITIH2**	**ITIH2**	**+**	**cluster 1**	**+**	**0.001**	**-0.19**	**MDD up**	**Y**
TRFE	TF	+	cluster 2	+	0.002	0.26	BD up	N
SAA1	SAA1	+	cluster 4	+	0.004	-1.03	MDD up	N

MDD vs SCZ

Protein	Gene symbol	ANOVA Significant (MDD vs SCZ vs HC)	Clusters	Significance of post-hoc analysis (MDD vs SCZ)	P-value (Tukey's HSD)	Log_2(Fold-change)	Expression pattern	Consistency of statistical significance and expression pattern for disease types
**ITIH2**	**ITIH2**	**+**	**cluster 2**	**+**	**0.003**	**-0.13**	**MDD up**	**Y**
ALDOC	ALDOC	+	cluster 4		0.993	0.05	SCZ up	N
PROS	PROS1	+	cluster 1	+	0.006	-0.14	MDD up	N
CBG	SERPINA6	+	cluster 2		0.236	-0.59	MDD up	N
**TFPI1**	**TFPI**	**+**	**cluster 1**	**+**	**0.043**	**-0.86**	**MDD up**	**Y**
SAA1	SAA1	+	cluster 4	+	0.024	-0.23	MDD up	N

BD vs SCZ

Protein	Gene symbol	ANOVA Significant (BD vs SCZ vs HC)	Clusters	Significance of post-hoc analysis (BD vs SCZ)	P-value (Tukey's HSD)	Log_2(Fold-change)	Expression pattern	Consistency of statistical significance and expression pattern for disease types
**C1RL**	**C1RL**	**+**	**cluster 1**	**+**	**0.003**	**-0.21**	**BD up**	**Y**

† Proteins of MPM models overlapping with DEPs of proteomic profiling data are listed for each pairwise comparison of groups. For each pairwise comparison, ANOVA significance, designated clusters, significance of post-hoc analysis, Log_2 (Fold-change) values calculated from proteomics profiling data, and corresponding expression pattern are signified. In addition, consistency of statistical significance and expression pattern between targeted and proteomic profiling for disease types are denoted as Y or N. Proteins satisfying the consistency are represented as a bold font.

Abbreviations
MDD: major depressive disorder, BD: bipolar disorder, SCZ: schizophrenia, HC: healthy control, MPM: multiprotein marker, HSD: honestly significant difference, Y: yes, N: no
